# Prevalence of comorbidities and their impact on survival among older adults with the five most common cancers in Taiwan: a population study

**DOI:** 10.1038/s41598-023-29582-0

**Published:** 2023-04-25

**Authors:** Li-Hsin Chien, Tzu-Jui Tseng, Tzu-Yu Chen, Chung-Hsing Chen, Chia-Yu Chen, Hsin-Fang Jiang, Fang-Yu Tsai, Hsiu-Ying Ku, Shih Sheng Jiang, Chao A. Hsiung, Tsang-Wu Liu, I-Shou Chang

**Affiliations:** 1 Institute of Population Health Sciences, National Health Research Institutes, 35 Keyan Road, Zhunan, 35053 Miaoli County Taiwan; 2 Department of Applied Mathematics, Chung-Yuan Christian University, Chong-Li, Taiwan; 3 Center of Biomedical Resources, National Health Research Institutes, 35 Keyan Road, Zhunan, 35053 Miaoli County Taiwan; 4 Department of Bachelor’s Degree Program for Indigenous Peoples in Senior Health and Care Management, National Taitung University, 369 Section 2, University Road, Taitung City, Taitung County, 95092 Taiwan; 5 Master Program in Biomedicine, National Taitung University, 369 Section 2, University Road, Taitung City, 95092 Taitung County Taiwan; 6 National Institute of Cancer Research, National Health Research Institutes, 35 Keyan Road, Zhunan, 35053 Miaoli County Taiwan

**Keywords:** Cancer epidemiology, Cancer epidemiology, Geriatrics

## Abstract

Because of the cancer incidence increase and population aging in Taiwan, we aimed to assess the cancer prevalence, to summarize the comorbidities of older patients with the five most common cancers (i.e., breast, colorectal, liver, lung, and oral), and to develop a Taiwan cancer comorbidity index (TCCI) for studying their actual prognosis. The linkage of the Taiwan Cancer Registry, Cause of Death Database, and National Health Insurance Research Database was used. We followed the standard statistical learning steps to obtain a survival model with good discriminatory accuracy in predicting death due to noncancer causes, from which we obtained the TCCI and defined comorbidity levels. We reported the actual prognosis by age, stage, and comorbidity level. In Taiwan, cancer prevalence nearly doubled in 2004–2014, and comorbidities were common among older patients. Stage was the major predictor of patients' actual prognoses. For localized and regional breast, colorectal, and oral cancers, comorbidities correlated with noncancer-related deaths. Compared with the US, the chances of dying from comorbidities in Taiwan were lower and the chances of dying from cancer were higher for breast, colorectal, and male lung cancers. These actual prognoses could help clinicians and patients in treatment decision-making and help policymakers in resource planning.

## Introduction

Because of worldwide population aging, the increasing number of older patients with cancer, the high prevalence of comorbidity among older patients with cancer, and the low inclusion rates of older patients in clinical trials, oncological practice among older patients is challenging and needs improvement^1–4^. Comorbidities can affect treatment decisions and outcomes. Clinical management and treatment decision-making must be improved for older adults with cancer because of their comorbidities. To help physicians select the best cancer treatment, comorbidity assessment prior to initiation of oncological treatment is recommended. One critical strategy is to develop standardized comorbidity measurements to assess the impact of specific combinations of comorbidities on older adults with cancer^5–8^.

Important advances in this direction include determining the 5-year chances of dying from cancer and from noncancer by age, stage, and comorbidity levels for older patients with breast, prostate, colorectal, or lung cancers in the US^9–11^. These measures of patients' actual prognoses provide important information for clinicians and patients to determine their treatment options and point out the importance of coordinating both their cancer-related care and noncancer-related care. All these help policymakers allocate healthcare resources and researchers design trials for cancer treatments in older adults with cancer and preexisting comorbidities^12–17^.

The comorbidity level in these studies was determined jointly by clinical judgment and the National Cancer Institute combined comorbidity index (NCICI), which reflects the hazard ratio associated with the time from cancer diagnosis to noncancer-related death^18–20^.

It has been reported that in Taiwan, cancer incidence has increased over the past forty years^21–23^, and the population is aging, with the percentage of residents aged ≥ 65 years increasing from 2.5% in 1955 to 13.9% in 2017^24^. Although the association between cancer incidence and industrialization in Taiwan starting in the 1960s has been discussed^25^, it is important to note that these observations, together with an improved net survival for patients with cancer in Taiwan^19^, suggest that cancer survivors are likely to become more prevalent, and studies of comorbidities among patients with cancer may be timely for better clinical management and surveillance^26–28^. The aforementioned advances in developed countries are relevant in Taiwan.

This study aimed to report the prevalence of cancer survivors in Taiwan and their comorbidities before cancer diagnosis, to develop a Taiwan cancer comorbidity index (TCCI) for older patients with the five cancers having the highest incidence and mortality in 2014 (i.e., breast, colorectal, liver, lung, and oral)^29^, and to use it to study their actual prognoses by age, stage, and comorbidity levels. Because the prevalence of comorbidities and their effects on cancer patients in Taiwan may be different from those in developed countries, we followed the NCICI to develop the TCCI by slightly modifying the coding of a few comorbidities. We expect to provide information useful for health professionals to improve caring quality and to researchers to conduct further studies.

## Methods

### Study population

This study was based on the linkage of the Taiwan Cancer Registry (TCR), National Health Insurance Research Database (NHIRD), and Cause of Death Database (TCOD); they were used in our earlier studies^30–32^.

The TCR collects information on patients with primary cancers at all hospitals in Taiwan with 50 or more beds. The quality of the TCR is improving and was reviewed previously^22,33^. The TCR included 1,934,198 records for 1979–2014, with one record for each primary cancer. After basic data checks and cleaning using birth date and sex, 1,852,694 cancer cases involving 1,699,907 patients were included.

Taiwan's National Health Insurance (NHI) program (implemented in 1995 by the NHI Administration) provides compulsory universal health insurance and covers all health care services for more than 99% of Taiwan's population. It is characterized by good accessibility, short waiting times, and low cost, among others; however, problems with the system include poor gatekeeping of specialist services; patients can self-schedule hospital visits without a general practitioner’s referral^34^. The NHIRD is built on data from this program, and we used the 2000–2015 data in this study. While data in the NHIRD have proven to be valuable resources for health science research, there are limitations^35,36^.

TCOD has included the unique underlying cause, not the multiple cause, of death for individuals in Taiwan since 1971 and used the national identification card number (NICN) since 1985^37^. The original TCOD contained 4,191,373 individual records for 1985–2016. Eliminating inconsistencies in the NICN, sex, birth date, death date, and cause of death yielded 4,054,632 unique death records for this period.

Using the linked datasets, we studied the prevalence of cancer survivors, including patients with invasive or noninvasive cancers. We considered an individual a cancer survivor at the end of 2014 if he/she was included in the TCR for the period 1979–2014, not included in the TCOD for the period 1985–2014, but included in the NHIRD for the period 2000–2014; thus, there might be some minor underestimation of cancer prevalence due to cancers diagnosed before 1979. For each cancer survivor, the time interval from his/her cancer diagnosis to the end of 2014 was his/her survival time. Similarly, we considered cancer prevalence in 2004 and 2009 in this study.

For comorbidity, we studied patients whose first invasive primary cancer was breast, colorectal, liver, lung, or oral cancer and was diagnosed between 2004 and 2014. Table S1 presents the ICD9 codes for these cancers. From 2004, 27 hospitals, and from 2007, all participating hospitals were required to use the TCR long form to collect more information on patients with these cancers, including the sage. Table S2 reports the numbers of patients with these five cancers in the TCR. The restriction of the linkage of the TCR from 2004–2014, NHIRD from 2000 to 2014, and TCOD from 2004 to 2016 to those whose first primary cancer was the five cancers is referred to as the Linked Dataset.

**Figure 1:**
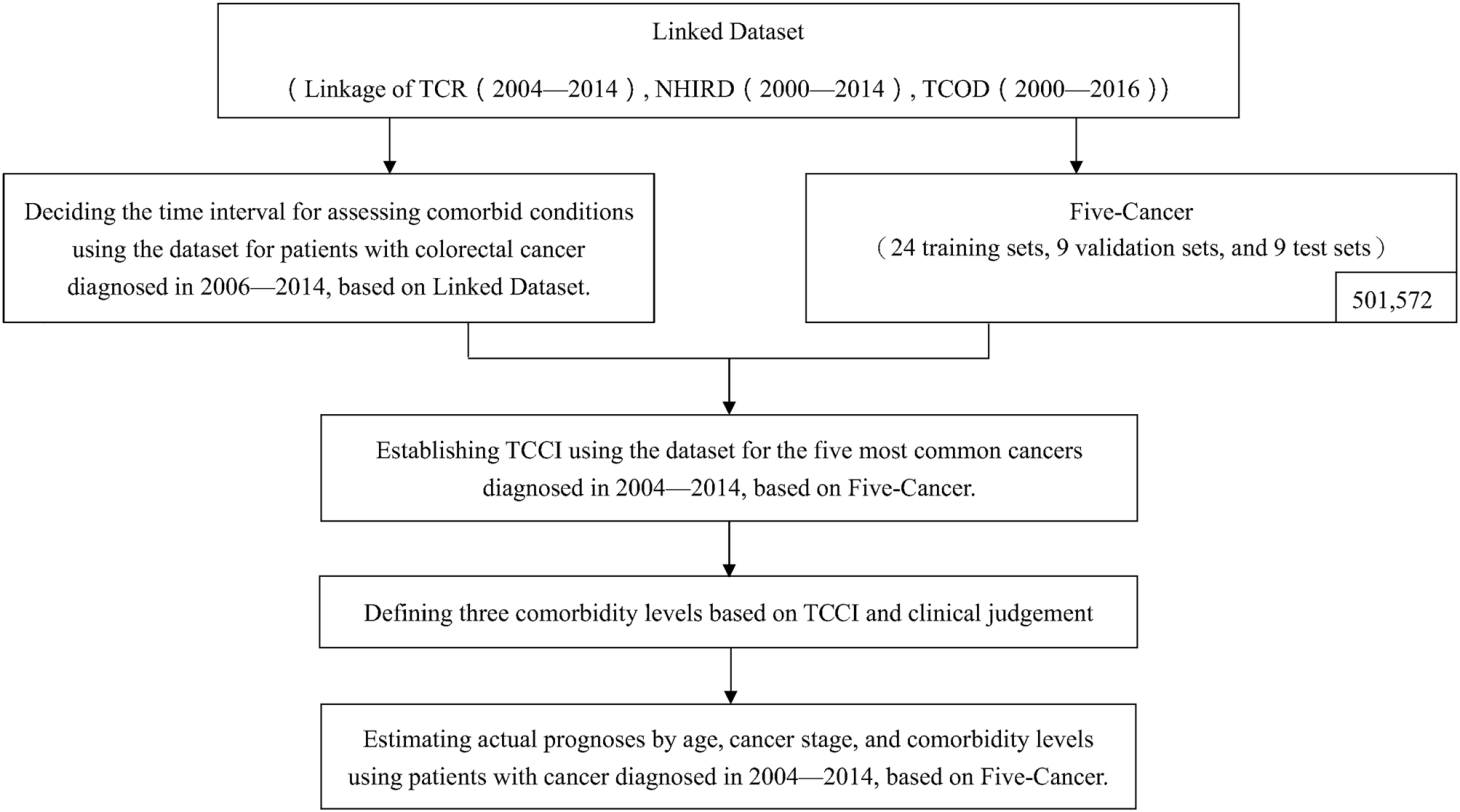
Flow chart of this study.

**Figure 2:**
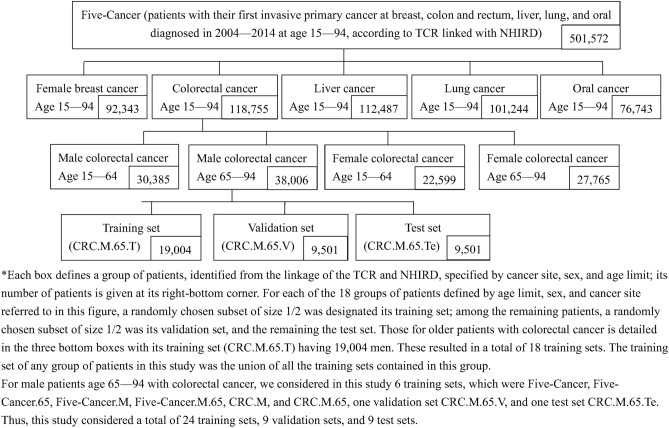
The forming of the 24 training sets, 9 validation sets, and 9 test sets in this study.

Figure 1 outlines the work flow of this comorbidity study. After forming the Linked Dataset, we decided the time interval prior to cancer diagnosis for comorbidity assessment and formed training sets, validation sets, and test sets for TCCI development, validation, and evaluation, which are described in detail in Fig. 2. Details are given below.

### Noncancer death

This study used the Surveillance, Epidemiology, and End Results Program (SEER) cause-specific death classification algorithm to define noncancer-related death^38^. This algorithm has been essential for cancer survivorship studies^9–11,20^. Our earlier publication used this algorithm and showed that cause-specific survival and relative survival for common cancers in Taiwan are comparable, thus suggesting the validity of this SEER algorithm in Taiwan^32^.

### Comorbidity definition

The comorbidities considered in this study were mainly adopted from those in NCICI except modifying mild liver diseases by incorporating viral hepatitis B and C to reflect their high prevalence in Taiwan and their roles in cancer development^39^^,^^40^ and including hypertension without and with complications, which was mentioned in the discussion of Stedman and colleagues on their study limitation^20^ and whose associations with cancer have been widely studied; see, for example, Seretis and colleagues and Dima and colleagues^41,42^. We considered 18 comorbidities in this study; they are shown in Table 1, and their ICD 9 codes are shown in Table S3. They included 16 of the 19 comorbidities defining the Charlson Comorbidity Index but excluded solid cancer, leukemia, and lymphoma because of our study focus^43^.

**Table 1:** The prevalence of selected comorbidities for the 5 most common cancers in Taiwan diagnosed in 2004–2014 and for individuals without cancer sampled in 2004–2014, ages 65–94.

Ages 65–94	Breast		Colorectum		Liver		Lung		Oral		Noncancer cohort	
**Number of individuals**	16,734		65,771		57,201		64,196		14,351		268,379	
**Status**	N	%	N	%	N	%	N	%	N	%	N	%
Number alive*	11,231	67.1	27,239	41.4	9416	16.5	6759	10.5	5022	35.0	NA	NA
Cancer deaths	3454	20.6	28,723	43.7	43,164	75.5	53,193	82.9	7043	49.1	NA	NA
Other-cause deaths	2049	12.2	9809	14.9	4621	8.1	4244	6.6	2286	15.9	NA	NA
Number of comorbid conditions**	N	%	N	%	N	%	N	%	N	%	N	%
0	4466	26.7	17,696	26.9	9848	17.2	17,633	27.5	4664	32.5	90,435	33.7
1	4405	26.3	16,241	24.7	11,088	19.4	15,767	24.6	3410	23.8	62,370	23.2
2	3902	23.3	14,554	22.1	13,227	23.1	14,092	22.0	2907	20.3	52,817	19.7
3	2177	13.0	8805	13.4	10,505	18.4	8539	13.3	1770	12.3	31,694	11.8
4+	1784	10.7	8475	12.9	12,533	21.9	8165	12.7	1600	11.1	31,063	11.6
**HT UC** (Hypertension, uncomplicated**)**	7993	47.8	29,984	45.6	26,850	46.9	27,797	43.3	5835	40.7	112,034	41.7
**DM W/O CC** (Diabetes without chronic complication)	4211	25.2	15,080	22.9	16,482	28.8	11,851	18.5	2993	20.9	52,033	19.4
**HT C** (Hypertension, complicated)	3619	21.6	13,783	21	11,555	20.2	12,387	19.3	2379	16.6	49,735	18.5
**Ulcer**	2384	14.3	10,565	16.1	13,707	24	10,632	16.6	2024	14.1	38,874	14.5
**COPD** (Chronic obstructive pulmonary disease)	1758	10.5	10,554	16	9651	16.9	16,045	25	2500	17.4	38,696	14.4
**CVD** (Cerebrovascular disease**)**	1909	11.4	9360	14.2	7341	12.8	9159	14.3	1865	13	35,611	13.3
**DM W CC** (Diabetes with chronic complication)	1399	8.4	5045	7.7	5734	10	3901	6.1	955	6.7	17,267	6.4
**Mild LD** (Cirrhosis/chronic hepatitis, HBV, HCV)	1194	7.1	4021	6.1	23,934	41.8	3767	5.9	1084	7.6	16,412	6.1
**CHF** (Congestive heart failure)	689	4.1	3780	5.7	3358	5.9	3682	5.7	607	4.2	13,666	5.1
**CRF** (Chronic renal failure)	679	4.1	3847	5.8	4146	7.2	3206	5	691	4.8	12,902	4.8
**Dementia**	558	3.3	2533	3.9	1865	3.3	2206	3.4	364	2.5	10,563	3.9
**PVD** (Peripheral vascular disease)	213	1.3	962	1.5	873	1.5	1053	1.6	208	1.4	3799	1.4
**RD** (Rheumatic disease)	191	1.1	551	0.8	587	1	600	0.9	126	0.9	2653	1
**Paralysis**	116	0.7	603	0.9	440	0.8	546	0.9	146	1	2456	0.9
**AMI** (Acute myocardial infraction)	58	0.4	567	0.9	389	0.7	578	0.9	98	0.7	2199	0.8
**OLD MI** (Old myocardial infraction)	48	0.3	483	0.7	374	0.7	611	1	109	0.8	1793	0.7
**MS LD** (Moderate-server liver disease)	32	0.2	142	0.2	2564	4.5	85	0.1	65	0.5	584	0.2
**AIDS**	0	0	0	0	13	0	5	0	0	0	22	0

*Death due to the cancer or other causes was decided by the SEER (NCI) classification algorithm using the TCOD and TCR from 2004 until Dec. 31, 2016; survival information of patients not included in the TCOD were obtained from the beneficiary registry of NHIRD at Dec. 31, 2015; the latter were all considered alive.

**The percentage is calculated by deleting the cells whose case number < 5. The 11 most common comorbidities for these 5 cancers in the order of their numbers are: HT UC (98,458), DM W/O CC (50,617), HT C (43,723), COPD (40,508), Ulcer (39,312), Mild LD (34,000), CVD (29,634), DM W CC (17,034), CRF (12,569), CHF (12,116) and Dementia (7526).

### Intervals defining comorbidity

Because comorbidity assessment depends on the time interval before cancer diagnosis, we followed Maringe and colleagues to determine the interval for comorbidity assessment for this study^44^. We know that longer intervals for comorbidity assessment provide more information for each patient but include fewer patients for the study because the NHIRD started in 1995 and adopted ICD9 exclusively in 2000. With these in mind, we present in Table S4 the hazard ratios from fitting Cox regression models, including only a single comorbidity as the covariate of interest and using time from cancer diagnosis to noncancer-related death as the outcome, based on all patients with colorectal cancer in the TCR from 2006–2014. Tables S4-1 and 4–2 regard patients aged 15–64 and 65–94, respectively. We considered three comorbidity assessment time intervals, 30 months, 54 months, and 78 months, before the cancer diagnosis and explored the sex-specific comorbidity effect. In fact, we followed Maringe and colleagues to exclude comorbidities that appeared only in the six months immediately before the cancer diagnosis to reduce the comorbidities caused by the cancers. A patient was said to have a specific comorbidity if their inpatient files contained a diagnosis of this comorbidity within the earlier 24, 48, or 72 months or if their outpatient files contained two diagnoses of this comorbidity in these periods with a gap > 1 month. The Supplementary Materials and Table S4 give more details in this regard.

For the age group 65–94, Table S4-2 shows that for the vast majority of the comorbidities, the differences in hazard ratios were small among these three assessment periods but not small between sexes; thus, we decided to consider sex-specific assessment with a 30-month period to include more patients in the study.

### Taiwan cancer comorbidity index

We followed the standard three steps in statistical learning (i.e., model training, model selection, and model assessment) to obtain a survival model with good discriminatory accuracy in predicting death due to noncancer causes; see, for example, Chapter 7 of Hastie, Tibshirani, and Friedman^45^. We acquired data from the NHIRD and TCOD for each patient with the five studied cancers in the TCR during 2004–2014, aged between 15 and 94 years. This dataset was called "Five-Cancer". We report in Tables S5-1, for those aged 15–64, and S5-2, for those aged 65–94, the numbers and percentages of these patients who had any of the 18 comorbidities and who were alive at the end of 2016. Five-Cancer was randomly divided into three disjoint parts: one half was the training set, one quarter was the validation set, and the remaining quarter was the test set. Five-Cancer had a total of 501,572 patients, as shown in Table S2.

Using the Five-Cancer training set, we fitted Cox regression models with time from diagnosis to noncancer-related death as the outcome. Censoring events included cancer-associated deaths or loss to follow-up as per the linkage of the TCR, TCOD, and NHIRD. Table S6-1 presents the estimated coefficients of the Cox model, including all 18 comorbidities and the interactions of any two of the 11 most common comorbidities ("Main18&11") as covariates. Three of the estimated main effects of the comorbidities were negative. We deleted the comorbidities with negative coefficients altogether and refitted the model until all the main effects were positive; whenever a comorbidity was deleted, interaction terms involving it were also deleted. The resulting model was termed "Main18&11.ND", for whom Table S6-2 presents the hazard ratios and coefficients. We note that the negative coefficients of the main effects may result from interactions or residual confounders. The motivation to delete them was to increase the interpretability, although it does not address the possible issue of bias.

A patient's TCCI in this study was defined to be the sum of the coefficients in the Cox model Main18&11.ND corresponding to the patient's comorbid conditions and interaction terms. We chose this for its excellent performance and simplicity.

In fact, we systematically considered 24 subsets of Five-Cancer defined by age, sex, and cancer site and divided each of them randomly into a training set, a validation set, and a test set. These divisions were compatible among these 24 subsets in the sense that if one subset was included in another subset, the training set, validation set, and test set of the former were included in the counterparts of the latter. The formation of these sets is detailed in Fig. 2. We fitted several Cox’s regression models to each of the 24 training sets and computed the time-dependent area under the operating characteristic curve (AUC) at 1 year, 2 years, and 5 years from diagnosis in each validation set. This AUC, a predictive accuracy measure, is the time-dependent extension of the analysis by Heagerty and Zheng^46^. Table S7 presents the 5-year AUCs evaluated in each of the sex- and site-specific validation sets of cancer patients aged 65–94. According to Tables S7-1–S7-9, the more intuitive Cox model Main18&11.ND trained by Five-Cancer generally performed very well across all these validation sets. The Supplementary Methods detail the construction of the training sets, validation sets, and test sets and the Cox models considered and assessed.

### TCCI and comorbidity levels

Based on the TCCI and clinical judgment, we followed Cho and colleagues and Edwards and colleagues to consider three comorbidity levels^9,11^. Patients with none of the 18 comorbidities were coded as 0. Patients were considered to have a severe comorbidity and coded as 2 if their TCCIs were > 0.66 or they had severe illnesses, such as COPD, liver dysfunction, chronic renal failure, dementia, or congestive heart failure, which frequently lead to organ failure or systemic dysfunction and usually require adjusting the cancer treatment. We note that according to NCICI, patients with exactly one comorbidity are coded 2 if and only if they have NCI index weights > 0.66^9^; this statement also held true in this study, except for those with COPD only. Patients coded as neither 0 nor 2 were coded as 1 and said to have a low/moderate comorbidity. Note that the cutoff of 0.66 was coincidentally the same as that of Edwards et al.^9^. Note also that AMI is usually not excluded from cancer clinical trials unless it is within 12 months prior to randomization; see, for example, the protocol in Krop and colleagues^47^. This may be the reason that patients were not coded 2 if AMI was the only comorbidity. In evaluating the effect of targeted therapy on lung cancer patients, we studied patients aged 30–94 years with distant stage by histology, although TCCI was not evaluated for younger patients.

### Noncancer cohort comorbidity

We constructed a cohort representing the 2004–2014 Taiwan population without cancer diagnoses using the TCR, TCOD, NHIRD, and Monthly Bulletin of Interior Statistics. Details are given in the Supplementary Materials.

### Statistical analysis

In this study, all the fitting of Cox's models, for choosing the time interval for comorbidity assessment and for constructing TCCI based on the training sets, were carried out using the R package ‘survival’. The time-dependent AUCs for the Cox models based on the validation datasets were obtained using the R package ‘risksetROC’, studied by Heagerty and Zheng^46^. The actual prognoses were computed using the R package ‘cmprsk’^48^, which estimates the subdistributions of a competing risk.

All methods were performed in accordance with the relevant guidelines and regulations of Scientific Reports. This study used only datasets for which all personal information had been deidentified by the Health and Welfare Data Science Center, Ministry of Health and Welfare of Taiwan. There was no patient contact for the study; therefore, there was no patient consent process. The Institutional Review Board of National Health Research Institutes, Taiwan approved this study (EC1030707-E) and waived the need for informed consent for this study as part of the study approval. Indeed, all the analyses were conducted in a secured area administered by the Health and Welfare Data Science Center, Ministry of Health and Welfare of Taiwan (https://dep.mohw.gov.tw/DOS/sp-GS-113.html? Query and https://dep.mohw.gov.tw/DOS/sp-GS-113.html?Query). Only summary tables could be brought out after verification by the officials.

### Ethical approval

This study was approved by Institutional Review Board of National Health Research Institutes, Taiwan (EC1030707-E), which conforms to the STROBE GUIDELINE for observation studies. All methods were performed in accordance with the relevant guidelines and regulations.

## Results

### A rapid increase in the number of cancer survivors

**Figure 3:**
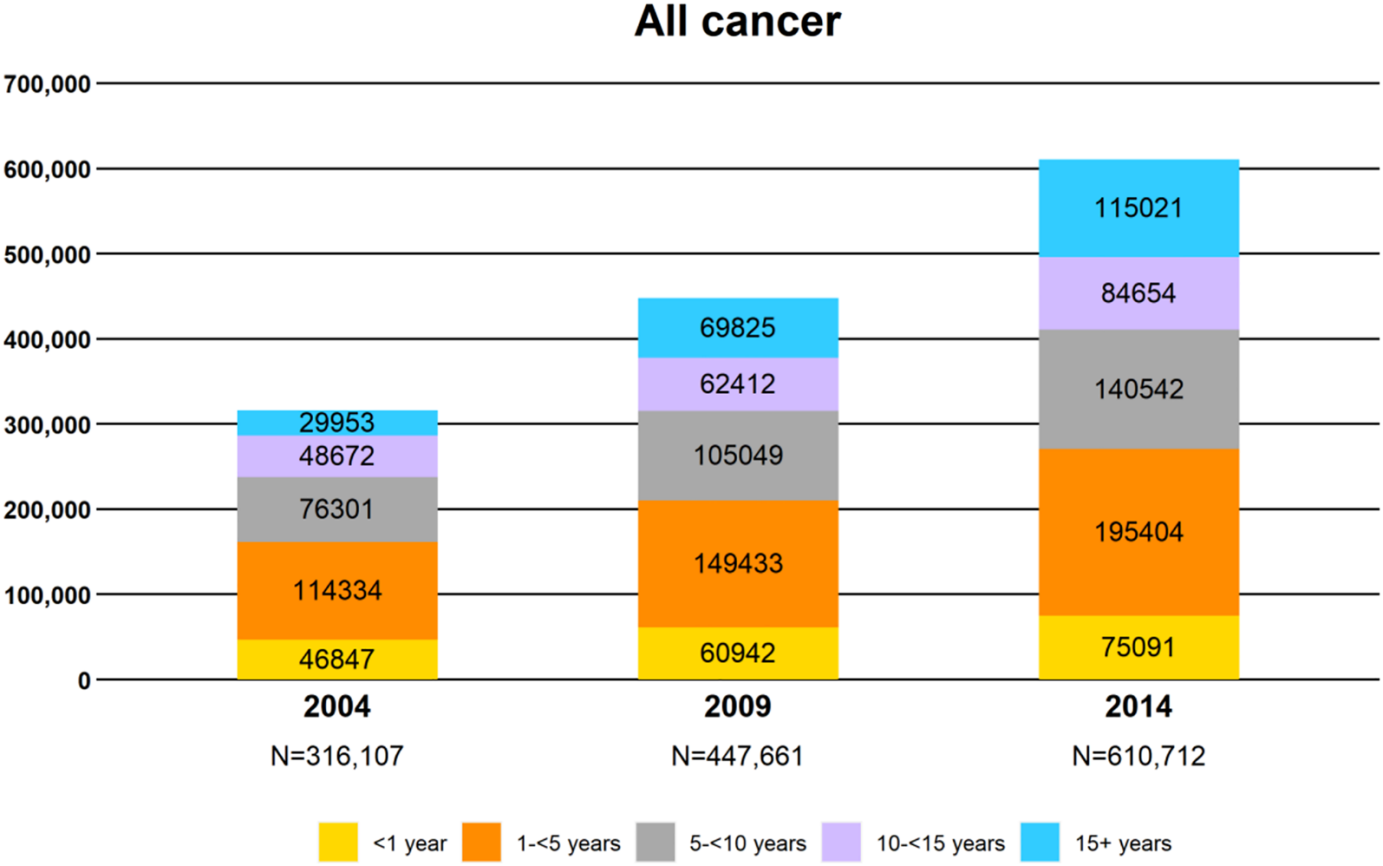
Prevalence of cancer survivors by calendar year and number of years from diagnosis: All cancer.

Figure 3 shows that the total number of cancer survivors increased drastically, from 314,107 in 2004 to 610,712 in 2014, and the number of long-term survivors who survived > 15 years increased from 29,953 in 2004 to 115,021 in 2014, a fourfold increase, which was much faster than that in the US^49^. Figure S1 presents the corresponding numbers for each of the five most common cancers, indicating that breast cancer in women had more long-term survivors than the other four cancers. Thus, cancer survivorship warrants immediate attention in Taiwan.

### Prevalence of comorbidities

Table 1 reports the prevalence of the 18 comorbidities in the cancer patient cohorts and the noncancer cohort aged 65–94 years; Tables S5-1 and S5-2 show that a much higher comorbidity prevalence existed among those aged 65–94 than among those aged 15–64 years. For example, 73.3% of breast cancer patients aged 65–94 had at least one comorbidity, while 26.5% of those aged 15–64 had at least one comorbidity. Among the elderly patients in Taiwan, hypertension, diabetes, ulcer disease, COPD, and CVD were the most common comorbidities, with a prevalence higher than 10%; liver disease, CHF, and CRF were the next most common comorbidities, with a prevalence of 5–10%. There were differences in comorbidity prevalence compared to those in the US and UK^9,20,50^. For example, CHF and PVD had higher ranks in the US and UK, and liver disease and ulcer disease had higher ranks in Taiwan. In Taiwan, the noncancer and oral cancer cohorts had the fewest comorbidities; patients with liver cancers had the most comorbidities, and 25%–28% of patients with breast, colorectal and lung cancer had no comorbidities. In the US, breast cancer had a similar comorbidity prevalence to the noncancer cohort, and lung cancer had a much higher comorbidity prevalence^9^. However, COPD was most prevalent in patients with lung cancer in both the US and Taiwan.

We present the weights for computing the TCCI for each patient in Table 2, which is the same as Table S6-2 and was used to define three levels of comorbidity. Based on these, Table 3 reports the numbers and percentages of patients by stage, age, and comorbidity levels for each cancer. Here, the cancer stage follows the SEER summary stage described in Table S8, which converts the stage at diagnosis from the tumor, node, metastasis (TNM) staging system to the SEER summary stage. Tables S9–S11 provide additional information about Table 3. Table 3 shows that comorbidity prevalence increased with age; breast cancer, colorectal cancer, and liver cancer had more patients diagnosed with early stages, oral cancer had more in regional stage, and lung cancer had the majority in late stage.

**Table 2:** Hazard ratios and the coefficients from Negative Deleted Model Main18 & 11.ND based on the training set of Five-Cancer.

Comorbid condition	Coef.	HR	Comorbid condition	Coef.	HR
Age	0.06	1.07	CHF*HT UC	−0.17	0.85
Sex	0.49	1.63	CVD*COPD	0.01	1.01
AMI	0.27	1.3	CVD*Dementia	−0.07	0.93
Old MI	0.08	1.08	CVD* DM W/O CC	0.12	1.13
CHF^†^	0.75	2.11	CVD* DM W CC	−0.08	0.92
PVD	0.24	1.27	CVD*CRF	−0.16	0.85
CVD	0.37	1.45	CVD*HT UC	0.06	1.06
COPD	0.26	1.3	COPD*Dementia	0.05	1.05
Dementia^†^	0.66	1.94	COPD* DM W/O CC	−0.1	0.9
Paralysis	0.34	1.4	COPD* DM W CC	−0.02	0.98
DM W/O CC	0.28	1.32	COPD*CRF	−0.17	0.84
DM W CC	0.38	1.46	COPD*HT UC	0.01	1.01
CRF^†^	0.80	2.23	Dementia* DM W/O CC	−0.02	0.98
MS LD^†^	0.81	2.24	Dementia* DM W CC	−0.05	0.95
RD	0.29	1.33	Dementia*CRF	−0.35	0.71
AIDS^†^	1.65	5.22	Dementia*HT UC	−0.04	0.96
HT UC	0.02	1.02	DM W/O CC * DM W CC	−0.11	0.89
CHF*CVD	−0.12	0.88	DM W/O CC *CRF	0.07	1.07
CHF*COPD	−0.11	0.9	DM W/O CC *HT UC	0.05	1.05
CHF*Dementia	−0.31	0.74	DM W CC *CRF	0.11	1.12
CHF* DM W/O CC	0.02	1.02	DM W CC *HT UC	0	1
CHF* DM W CC	−0.08	0.93	CRF*HT UC	−0.05	0.95
CHF*CRF	0.03	1.03			

^†^The coefficients are at least 0.66.

**Table 3:** Number and percentage of cancer patients by age, stage, and comorbidity level for each of the five cancers.

Age		15–64						65–74					
Comorbidity level		0		1		2		0		1		2	
		N	%	N	%	N	%	N	%	N	%	N	%
Breast cancer	Localized	27,105	48.8	8104	51.2	2092	49.7	1592	46.0	2688	49.4	1043	45.4
	Regional	18,393	33.1	5180	32.7	1353	32.1	1059	30.6	1704	31.3	722	31.4
	Distant	2846	5.1	542	3.4	147	3.5	268	7.7	268	4.9	119	5.2
	Others^+^	7216	13.0	2013	12.7	618	14.7	540	15.6	776	14.3	414	18.0
Colorectal cancer	Localized	10,569	32.1	5676	37.5	1791	36.8	3232	33.1	4678	36.7	2627	36.4
	Regional	10,507	31.9	4529	29.9	1316	27.0	2967	30.4	3687	28.9	1858	25.7
	Distant	6369	19.3	2303	15.2	729	15.0	1635	16.8	1898	14.9	1018	14.1
	Others^+^	5527	16.8	2636	17.4	1032	21.2	1926	19.7	2497	19.6	1715	23.8
Liver cancer	Localized	10,849	48.1	15,472	69.0	6950	67.5	2944	48.8	9884	66.3	6337	64.4
	Regional	4581	20.3	2670	11.9	996	9.7	1042	17.3	1701	11.4	989	10.1
	Distant	3956	17.5	1645	7.3	625	6.1	934	15.5	1211	8.1	753	7.7
	Others^+^	3184	14.1	2627	11.7	1731	16.8	1113	18.4	2103	14.1	1758	17.9
Lung cancer	Localized	3493	15.2	1987	20.3	815	18.9	1052	11.2	1622	15.7	1153	15.2
	Regional	3813	16.6	1731	17.7	887	20.6	1776	18.9	1846	17.9	1591	20.9
	Distant	13,593	59.2	5301	54.1	2103	48.8	5302	56.3	5728	55.6	3621	47.6
	Others^+^	2048	8.9	771	7.9	506	11.7	1283	13.6	1107	10.7	1240	16.3
Oral cancer	Localized	11,639	27.3	5008	34.4	1712	32.9	974	28.6	1252	35.3	744	31.7
	Regional	21,345	50.1	6782	46.6	2476	47.6	1624	47.7	1562	44.0	1016	43.3
	Distant	970	2.3	277	1.9	124	2.4	116	3.4	82	2.3	72	3.1
	Others^+^	8689	20.4	2480	17.0	886	17.0	690	20.3	650	18.3	512	21.8

Age		75–84						85–94					
Comorbidity level		0		1		2		0		1		2	
		N	%	N	%	N	%	N	%	N	%	N	%
Breast cancer	Localized	356	42.5	1034	46.5	696	46.0	58	36.0	161	40.3	172	44.3
	Regional	244	29.1	665	29.9	435	28.8	40	24.8	116	29.0	105	27.1
	Distant	84	10.0	136	6.1	104	6.9	14	8.7	45	11.3	20	5.2
	Others^+^	154	18.4	390	17.5	278	18.4	49	30.4	78	19.5	91	23.5
Colorectal cancer	Localized	1879	30.5	3790	33.6	3197	32.2	476	26.8	904	29.5	1128	29.6
	Regional	1819	29.5	3208	28.4	2558	25.7	435	24.5	850	27.8	888	23.3
	Distant	1073	17.4	1886	16.7	1525	15.3	312	17.5	503	16.4	582	15.3
	Others^+^	1386	22.5	2408	21.3	2656	26.7	556	31.3	804	26.3	1210	31.8
liver cancer	Localized	1388	45.6	5676	59.7	5167	57.3	275	35.7	846	46.0	998	44.3
	Regional	509	16.7	1177	12.4	993	11.0	105	13.6	282	15.3	290	12.9
	Distant	425	14.0	866	9.1	765	8.5	113	14.7	197	10.7	208	9.2
	Others^+^	722	23.7	1788	18.8	2090	23.2	278	36.1	515	28.0	759	33.7
Lung cancer	Localized	541	8.3	1017	10.2	1354	11.3	77	4.6	167	6.2	312	7.8
	Regional	1167	17.8	1747	17.6	2280	19.0	250	15.0	346	12.9	627	15.6
	Distant	3531	53.9	5603	56.3	5756	47.9	868	52.1	1545	57.6	1922	47.9
	Others^+^	1312	20.0	1582	15.9	2618	21.8	471	28.3	625	23.3	1150	28.7
Oral cancer	Localized	233	22.0	498	33.2	445	27.9	45	23.7	75	25.8	104	26.3
	Regional	507	48.0	623	41.6	729	45.7	103	54.2	135	46.4	193	48.9
	Distant	41	3.9	58	3.9	52	3.3	7	3.7	8	2.7	16	4.1
	Others^+^	276	26.1	320	21.3	368	23.1	35	18.4	73	25.1	82	20.8

^+^TCR cancer patients with no stage information; see Table S9 for details.

### Survival measures considering competing risks of death by comorbidity level

**Figure 4:**
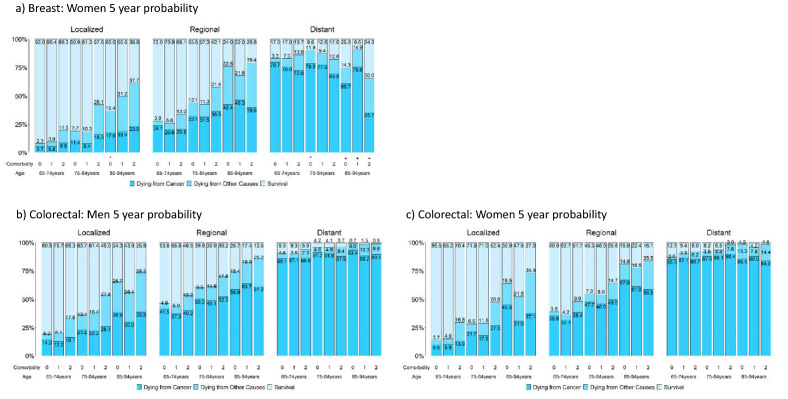

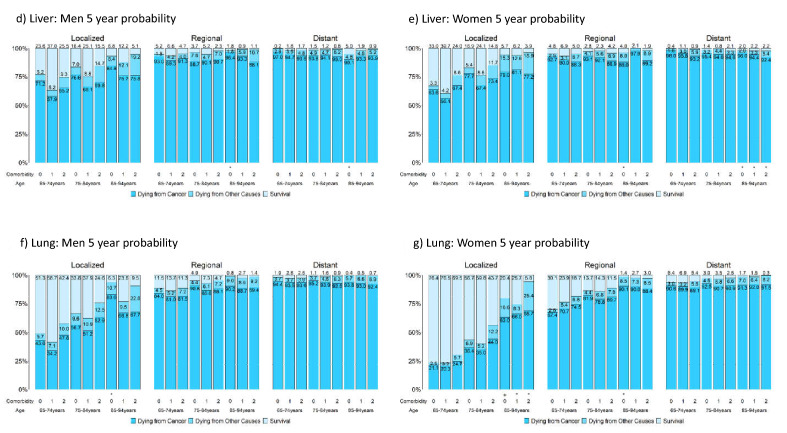

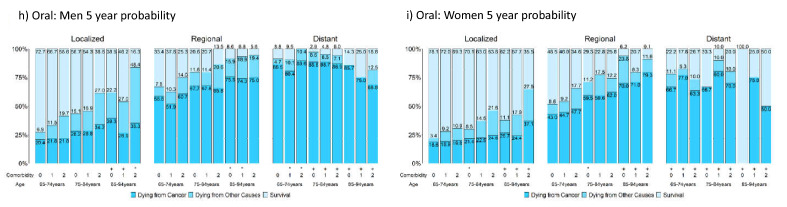
Five-year probabilities of dying from cancer, dying from other causes, and survival stratified by stage, age, comorbidity level, and sex for breast, colorectal, liver, lung, and oral cancer; strata having patients less than 100/50 are marked */+. A total of 9 panels are presented: panel a, b, c, d, e, f, g, h, and i are respectively for breast cancer, male colorectal cancer, female colorectal cancer, male liver cancer, female liver cancer, male lung cancer, female lung cancer, male oral cancer, and female oral cancer.

Figure 4 presents the 5-year probabilities of dying from cancer, dying from competing causes, and survival stratified by sex, stage, age, and comorbidity level for the five cancers. Table S12 reports their actual values and the corresponding 1-year and 2-year probabilities; Figure S2 presents the corresponding figures. Strata with fewer than 100/50 patients are marked with */+.

Among patients with localized and regional stage cancers, those with older age or severe comorbidity had lower survival rates, mainly due to increased deaths from competing causes. For patients with distant-stage cancers, age and comorbidities had a reduced effect, and the chances of dying from cancer were high. Although comorbidities affected both cancer-related and noncancer-related deaths, the effect was larger for noncancer-related deaths; this observation was in line with an Australia study of colorectal cancer, which included patients aged 18–80+^51^. Stage had a much larger effect on survival than age or comorbidity. Thus, the impact of age, comorbidity, and stage on the actual prognosis was generally similar to that reported in the US^10^.

Despite these similarities, there were considerable differences between Taiwan and the US. In Taiwan, patients with local or regional breast, colorectal, and lung cancers had lower chances of dying from competing causes and higher chances of dying from cancer, except for women with lung cancer.

Figure 4 shows that patients with liver and lung cancer had the highest probabilities of cancer-related death, and their comorbidities had smaller influences on death. Figure 4 also shows that patients with oral cancers had a better prognosis than those with liver and lung cancers when there were enough patients in the strata.

### Lung cancer subtypes

**Figure 5:**
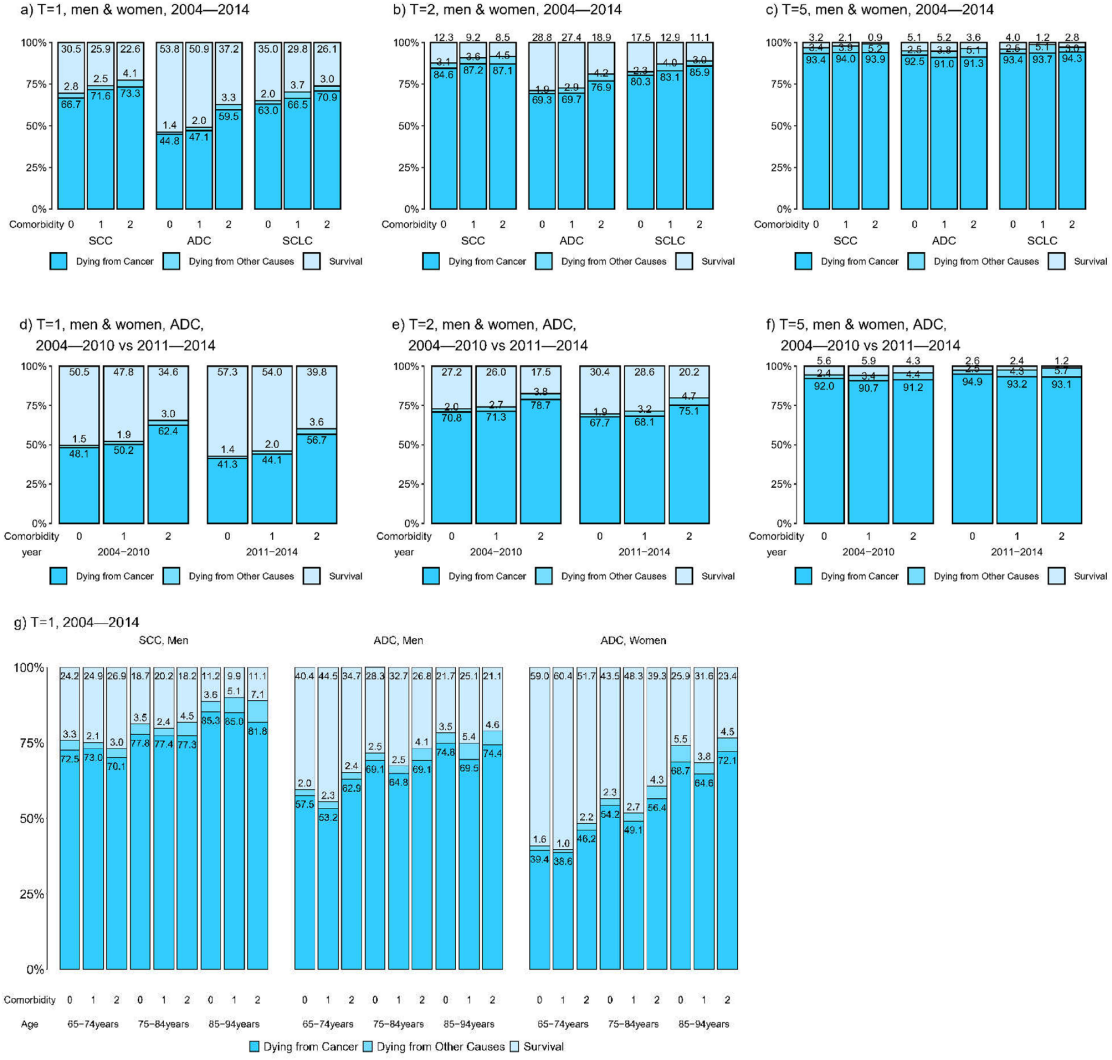
T-year probabilities of dying from cancer, dying from other causes, and survival stratified by comorbidity level, subtype and year at diagnosis for patients with distant lung cancer aged 30–94. Panels a, b, and c compares the one-, two- and five-year survival among subtypes. Panels d, e, f compares the one-, two, and five-year survival between calendar year periods. Panel g compares one-year survival between male SCC, male ADC, and female ADC.

For patients with distant lung cancer and aged 30–94, Fig. 5 shows that the overall survival for lung adenocarcinoma (ADC) was better than that for squamous cell carcinoma (SCC) of the lung and that for small cell lung cancer (SCLC); the difference was most obvious for one-year overall survival. It also shows that for lung ADC, the overall survival was better in 2011–2014 than in 2004–2010, and the difference was also most obvious for one-year overall survival. It also shows that the one-year overall survival was best for women with lung ADC, next for men with lung ADC, and worst for men with lung SCC. Supplementary Figures S3–S5 include other prognoses. Tables S13–S14 present the corresponding point estimates, confidence intervals, and other related statistics.

All of these findings are consistent with the 2011 Taiwan NHI Program policy that reimburses patients with late-stage lung ADC who have *EGFR* mutations for tyrosine kinase inhibitors (TKIs); *EGFR* mutations are common among never-smoking female lung ADC patients in Taiwan^52^.

The above observations from the 1-year probabilities became less prominent for the 2-year probabilities and nearly vanished for the 5-year probabilities (Figures S2–S3). This may reflect the palliative nature of the TKIs.

### Model performance

Table S7 reports the AUCs regarding the 5-year survival of noncancer deaths for indices based on different models and training sets. It is interesting to see from Tables S7-1–S7-9 that the AUCs did not change much by deleting the comorbidities with negative coefficients but did decrease clearly by backward stepwise variable selection, where one of the coefficients was still negative; see Table S6-3. They also varied little with training sets. Table S7-10 reports the 5-year AUCs using the test sets of those aged 65–94: 0.73 (breast), 0.71 (male colorectal), 0.75 (female colorectal), 0.68 (male liver), 0.69 (female liver), 0.64 (male lung), 0.72 (female lung), 0.65 (male oral), and 0.71 (female oral). These AUCs for breast, colorectal and lung cancer are 2% to 9% higher than those in the US^20^. The observation that male cancer patients had smaller AUCs might be caused by more deaths due to lung cancer.

## Discussion

This study reports that in Taiwan, the number of cancer survivors increased rapidly, comorbidities among older patients with cancer were common, and the comorbidity profile among Taiwanese older patients differed from those in the US and UK. Using the three comorbidity levels defined by the TCCI and clinical judgment, we reported the actual prognoses of patients with the five most common cancers, indicating that stage was the major predictor of patients' actual prognoses but for localized and regional breast, colorectal, and oral cancers, comorbidities correlated with noncancer-related deaths. Compared with the US, the chances of dying from comorbidities in Taiwan were lower, and the chances of dying from cancer were higher for breast, colorectal, and male lung cancers. These findings highlight the challenge of coordinating multidisciplinary cancer treatment and survivorship care and prompt future studies to determine whether cancer patients in Taiwan receive similar treatments for their comorbidities as their noncancer counterparts and whether their cancer treatments are unnecessarily modified.

Here are some remarks on the methodology of the TCCI. Although we modified the condition of mild liver disease by adding viral hepatitis B and C and included hypertension to reflect the high prevalence of these diseases in Taiwan, all the remaining comorbidities were adopted from the CCI and NCICI. While all these comorbidities are well established, we found that some of them had negative coefficients for their main effects in the resulting Cox models, suggesting the existence of correlation, interaction, or residual confounders among them. To make the model more intuitive and facilitate communication, we considered the procedure to eliminate the comorbidities with negative coefficients. Tables S7-1–S7-9 show that this procedure resulted in a more intuitive model without sacrificing performance. These tables also suggest that stepwise variable selection may suffer severe disadvantages. All the above are in line with those discussed in Steyerberg^53^ and Harrel^54^. Because we followed standard model development, selection, and assessment procedures strictly, the AUCs reported in Table S7-10 were based on the test sets, and the test sets were held back until the final assessment, the performance of the TCCI is likely reliable. Finally, we note that in the model selection step, we chose Main18&11, instead of Main11, because the former performed better in 7 of the 9 cancers, although only slightly.

Compared with the SEER studies, the chances of dying from competing causes are lower and those of dying from cancer are higher in Taiwan for local and regional breast, colorectal and male lung cancers^9^. This seems to be in line with the results based on net survival. Indeed, a comparison between the 5-year cancer cause-specific survival in Taiwan during 2000–2010, based on Table 3 in Chien and colleagues^32^, and that in the US SEER study during 1992–2004, based on Table 3 in Howlader and colleagues^38^, suggests that cancer survival of the breast and the colon and rectum in Taiwan seemed to be poorer than those in the US.

Comparing Table 2 with Stedman et al.^20^ suggests that COPD and chronic renal failure (CRF) exhibited the largest difference in hazard ratios. While the large hazard ratio for CRF might reflect the serious renal disease problem in Taiwan^55^, further studies are needed to understand the low hazard ratios for COPD in Taiwan. Because tobacco smoking is an important risk factor for both lung cancer and COPD and a large proportion of lung cancer patients are never-smokers in Taiwan^52,56^, it might be worthwhile to study the prognosis of lung cancer by smoking status.

A recent study suggested that targeted therapies may have contributed to the reduced mortality from non-small-cell lung cancer in the US population^57,58^. Our results on the actual prognoses for patients with distant-stage disease provide additional population-level support for the positive effects of recent advances in lung cancer treatment on patient outcomes, reflecting the 2011 reimbursement policy of the Taiwan NHI program.

Figure 4 indicates that for localized liver cancer, 5-year overall survival rates were better for those at comorbidity level 1 than for those without comorbidities. This might be related to the 2003 NHI policy that reimburses antiviral medications^59^ and suggests a future study that considers the actual prognoses of patients with liver cancer separately for those with and without hepatitis viral infections.

A major strength of this study is that the TCCI was developed and evaluated in a large dataset by following standard statistical learning methods; in addition, the comorbid conditions were selected from a literature review, and the comorbidity assessment period was decided empirically. Table S7 exemplifies the advantage of a large training set in terms of predictive performance.

There are some limitations to this study. The comorbid conditions, assessed by the administrative dataset NHIRD, do not reflect their severity. Another limitation is that including only comorbidities with positive main effects in defining TCCI promotes communication but may cause some bias.

Effects of comorbidities on actual prognoses have been studied in Australia, England, and the US^51,60,61^. Although there are underlying similarities between our study and theirs, comparisons suggest that we should take into account additional risk factors, such as treatment and exposures, to obtain more precise prognoses. In particular, the role of socioeconomic status could be explored^50^. It is also desirable to improve the TCCI by including more comorbid conditions and based on cohorts of more cancer sites, for other uses in geriatric oncology^62^.

## Conclusions

The rapid increase in long-term cancer survivors and the widespread comorbidities among older cancer patients in Taiwan demand attention to their actual prognoses. In addition to providing information for patients and clinicians regarding treatment decisions and for policymakers regarding resource allocation, this study proposed TCCI and suggested important future research topics, which may also be relevant to geriatric oncology in other parts of the world.

## Supplementary Information

Supplementary Information 1.

Supplementary Information 2.

## Data Availability

All the datasets used in this study were provided by and all the analyses were carried out in one of the secure labs of the Health and Welfare Data Science Center, Ministry of Health and Welfare, Taiwan. All the data are de-identified. For information on how to submit an application for gaining access to these datasets, please follow the instructions at https://www.apre.mohw.gov.tw/ If some one wants to request the data from this study, please contact the corresponding author Dr. I-Shou Chang (ischang@nhri.org.tw) for more detailed information.
