# Supplementary material for: Prevalence of comorbidities and their impact on survival among older adults with the five most common cancers in Taiwan: a population study
Source: Sci Rep. 2023 Apr 25;13:6727. doi: 10.1038/s41598-023-29582-0 (PMC10130096; doi:10.1038/s41598-023-29582-0)
Supplement: Supplementary file 1 — Supplementary Information 1. [file 41598_2023_29582_MOESM1_ESM.docx]

**Supplementary Materials**

**Prevalence of comorbidities and their impact on survival among older adults with the five most common cancers in Taiwan: A population study**

**Authors**: Li-Hsin Chien, PhD+, Tzu-Jui Tseng, Dr.PH+, Tzu-Yu Chen, PhD, Chung-Hsing Chen, PhD, Chia-Yu Chen, MS, Hsin-Fang Jiang, MS, Fang-Yu Tsai, MS, Hsiu-Ying Ku, PhD, Shih Sheng Jiang, PhD, Chao A. Hsiung, PhD, Tsang-Wu Liu, MD*, I-Shou Chang, PhD*

+These authors contributed equally to this work and are considered co-first authors.

*These authors contributed equally and are both corresponding authors

**Comparison of intervals defining the comorbidity**

Assessing comorbid conditions using population-based administrative data requires a specification of the time interval for the assessment. To evaluate the effect of the interval for comorbidity assessment and to avoid the possibility of comorbid conditions due to cancer complications, we followed Maringe and colleagues to decide the interval for comorbidity assessment in Taiwan.(Maringe et al. 2017)

Given a cancer patient, we considered the disjoint and consecutive six-month periods from the cancer diagnosis date backward to 2000. We ordered these 6-month periods as follows; the most recent 6-month period was called period 1, the second most recent period was called period 2, …, etc. Given a comorbidity, we claim that this patient had this comorbidity in periods 2—K if there was a diagnosis from his/her impatient file in periods 2—K or if there were two diagnoses from his/her outpatient file in periods 2—K with the gap between them longer than one month.

Given any one of the 18 comorbidities, for K=5, 9, and 13, we obtained the hazard ratios of the comorbidity among the colorectal cancer patients, diagnosed between 2006 and 2014, by fitting three Cox regression models with the comorbidity as the only covariate of interest and with time from cancer diagnosis to noncancer death as the event. The first model considered all colorectal cancer patients and adjusted for age and sex; the second (third) model considered all the male (female) patients and adjusted for age only. Because the hazard ratios for K=9 often lie between those for K=5 and those for K=13, we report only the hazard ratios for K=5 and K=13 in Tables S4-1 and S4-2 with Table S4-1 for the age group 15—64 and Table S4-2 for 65—94. It follows from Table S4-2 that the hazard ratios from K=5 and those from K=13 were similar; hence, we decided to consider K=5 in this study. Because Table S4-2 also shows that the hazard ratios for diabetes with chronic complications, chronic renal failure, and moderate/severe liver disease were different between males and females, we decided to explore sex-specific comorbidity indexes in this study.

Together, Tables S4-1 and S4-2 show that sex-specific hazard ratios for younger adults were generally higher than those for older adults.

**Datasets for training, validation, and test**

In developing comorbidity indexes, we considered all the cancer patients collected in the TCR diagnosed from 2004—2014 and at age between 15 and 94. Tables S5-1 and S5-2 report the numbers and percentages of patients who had any of the 18 comorbidities and the number of patients alive before the end of 2016. Model development, selection and assessment all used this dataset. Six subsets of this dataset were considered: the dataset consisting of all the patients of any of the five cancers, referred to as Five-Cancer; Five-Cancer restricted to those ages 65—94 (Five-Cancer.65); Five-Cancer restricted to male (Five-Cancer.M); Five-Cancer.M restricted to 65—94 (Five-Cancer.M.65); Five-Cancer restricted to female (Five-Cancer.F); Five-Cancer.F restricted to 65—94 (Five-Cancer.F.65). We also considered 4 datasets of patients for each cancer of the oral, colon and rectum, liver, and lung. For example, all male oral cancer patients (Oral.M) and Oral.M restricted to ages 65—94 (Oral.M.65); similarly, Oral.F, Oral.F.65, CRC.M, CRC.M.65, CRC.F, CRC.F.65, Liver.M, Liver.M.65, Liver.F, Liver.F.65, Lung.M, Lung.M.65, Lung.F, Lung.F.65. We also considered 2 datasets of breast cancer patients: all female breast cancer patients (Breast.F), and Breast.F restricted to ages 65—94 (Breast.F.65).

We followed the suggestion from Hastie and colleagues (Hastie 2009) to divide each of the above 24 datasets randomly into 3 disjoint parts: one-half as a training set, one quarter as a validation set, and the remaining one quarter as a test set. This helps in dealing with the overfitting issue. This division was carried out for groups specified by cancer site, age at diagnosis (less than 65 or not), year of diagnosis, and gender and then merged them properly for each of the 24 datasets. The training sets were used to train the models; the validation sets were used to estimate prediction error for model selection; and the test sets were used for assessment of the generalization error of the selected models.

**Training the models**

For each of the 24 training sets, we fitted Cox’s regression models with time from diagnosis to noncancer death as the outcome. Censoring events includes death due to the cancer or loss to follow-up based on the linkage of the TCR, TCOD, and NHIRD.

Because some of the training sets had few patients with HIV, we considered 17 main effects without HIV in this situation. Three sets of comorbidities were used in fitting the Cox models. The first considered only the main effects (Main17 or Main18), the second the main effects together with the interactions of the most common 6 comorbidities (Main17&6 or Main18&6)), the third the main effects together with the interactions of the most common 11 comorbidities (Main17&11 or Main18&11). In fitting the models, we adjusted for age and sex when the dataset included both male and female patients and for age only when consisting of patients of the same sex. Thus, we obtained 3 Cox’s regression models for each training set. These models are summarily referred to as original models (OMs).

**More intuitive models and others**

Table S6-1 presents the estimated coefficients of the original model Main18&11 using the dataset Five-Cancer. Table S6-1 shows that some of the estimated coefficients of the comorbidities were negative, which is unintuitive. Thus, we deleted all the comorbidities with negative coefficients and fitted the model again until all the main effects were positive. The resulting model is termed Main18&11.ND and the hazard ratios are presented in Table S6-2. In fact, for each original model, we obtained the corresponding model with no negative coefficient; denote them accordingly by Main17.ND, Main18.ND, Main17&6.ND, Main18&6.ND, Main17&11.ND, or Main18&11.ND. Note that we did not mind if the coefficient of an interaction term was negative and that when a comorbidity was deleted due to its negative coefficient, we deleted all the interaction terms involving this comorbidity. These models are referred to as negative deletion models (NDMs).

Based on the original models, we also applied backward variable selection to eliminate variables with p-values larger than 0.05. These models are denoted by Main17.VS, Main18.VS, Main17&6.VS, Main18&6.VS, Main17&11.VS, or Main18&11.VS. They are referred to as variable selection models (VSM). Table S6-3 presents the estimated coefficients of Main18&11.VS using Five-Cancer.

**Comorbidity indexes for each cancer patients cohort**

For each of the 24 training sets, we obtained 9 Cox’s regression models as described in the last 3 paragraphs, where the coefficients of the comorbidities and the interaction terms provided the weights for defining the comorbidity indexes. For each of these Cox’s regression models, we defined its comorbidity index for a cancer patient to be the sum of the coefficients in the model corresponding to the comorbid conditions and the interaction terms this patient had.

Considering older breast cancer patients, for example, we could use the comorbidity weights estimated in any of the 9 Cox’s models trained by any of the training sets in Five-Cancer, Five-Cancer.65, Five-Cancer.F, Five-Cancer.F.65, Breast.F, or Breast.F.65. Thus, we had 54 sets of weights to consider and each of them defines a comorbidity index. Table S7-1 reports the AUCs, regarding 5-year survival, for these 54 indexes based on the validation set in Breast.F.65. Those based on other cancer validation sets from the older patients are shown in Table S7-2 through Table S7-9.

**Model selection and assessment: performance of negative deletion models**

Based on the validation sets from each of Breast.F.65, CRC.M.65, CRC.F.65, Liver.M.65, Liver.F.65, Lung.M.65, Lung.F.65, Oral.M.65, Oral.F.65, Table S7 reports the AUCs using original models, negative deletion models, and variable selection models and their differences. Table S7 shows that negative deletion models perform similarly to original models and better than variable selection models, suggesting the use of NDMs to define the TCI in this study. For each of the 9 validation sets, we marked in red the largest AUC among the 18 AUCs from OM, NDM and VSM. It follows from these tables that the index defined by the Cox’s model Main18&11.ND trained by Five-Cancer had a high AUC in each of the 9 validation sets. Because of this excellent performance and also because of simplicity, we chose this index in this study and referred to it as the Taiwan Comorbidity Index. The performance of the Taiwan Comorbidity Index was evaluated in the respective test sets in Table S7-10.

**Comorbidity in noncancer cohort**

We constructed a cohort that represents the 2004—2014 Taiwan population without any cancer diagnosis, using the TCR, TCOD, NHIRD, and Monthly Bulletin of Interior Statistics (MBIS). The NHIRD has information only on the birth year of each beneficiary; we randomly assigned a birth month and date to each beneficiary. For each calendar year from 2004 to 2014, we took samples from the NHIRD so that the sample size was 1% of the population of that year, its age distribution was similar to that of the population of that same year, and each individual sampled in that year had no cancer diagnosis up to his/her “assigned” birth day of that year, which is referred to as the enrollment time, and was not included in the samples from any earlier year. Based on the NHIRD, we report the comorbidity of each individual at the enrollment date in the same way as we did for cancer patients; specifically, we considered the 24-months period for assessment without skipping the first 6-month period.

References

Hastie, T., Tibshirani, R.,, Friedman, J. (2009), *The Elements of Statistical Learning* (Second ed.): Springer.

Maringe, C., Fowler, H., Rachet, B., and Luque-Fernandez, M. A. (2017), "Reproducibility, reliability and validity of population-based administrative health data for the assessment of cancer non-related comorbidities," *PLoS One*, 12 (3), e0172814. DOI: 10.1371/journal.pone.0172814.
