# Supplementary material for: Prevalence of comorbidities and their impact on survival among older adults with the five most common cancers in Taiwan: a population study
Source: Sci Rep. 2023 Apr 25;13:6727. doi: 10.1038/s41598-023-29582-0 (PMC10130096; doi:10.1038/s41598-023-29582-0)

**Supplementary Tables and Figures**

**Prevalence of comorbidity and impact on survival among older persons with the five most common cancers in Taiwan: A population study**

**Authors**: Li-Hsin Chien, PhD+, Tzu-Jui Tseng, Dr.PH+, Tzu-Yu Chen, PhD, Chung-Hsing Chen, PhD, Chia-Yu Chen, MS, Fang-Yu Tsai, MS, Hsiu-Ying Ku, PhD, Shih Sheng Jiang, PhD, Chao A. Hsiung, PhD, Tsang-Wu Liu, MD*, I-Shou Chang, PhD*

**Table of contents**

[Table S1. Definitions of the cancers in this study 1](#_Toc100567640)

[Table S2. Numbers of patients diagnosed with cancer of the breast, colon and rectum, liver, lung, and oral reported in the TCR from 2004 to 2014 by gender and age of diagnosis. 2](#_Toc100567641)

[Table S3. Definitions of comorbidities+ 3](#_Toc100567642)

[Table S4-1. The hazard ratio of each comorbidity with respect to death due to other causes, among colorectal cancer patients, ages 15-64. 4](#_Toc100567643)

[Table S4-2. The hazard ratio of each comorbidity with respect to death due to other causes, among colorectal cancer patients, ages 65-94 5](#_Toc100567644)

[Table S5-1. The number and percentage of patients with comorbid conditions for each of the 5 cancer cohorts and non-cancer cohorts, ages 15-64 6](#_Toc100567645)

[Table S5-2. The number and percentage of patients with comorbid conditions for each of the 5 cancer cohorts and non-cancer cohorts, ages 65-94 8](#_Toc100567646)

[Table S6-1. Hazard Ratios from Original Model Main18&11 using Five-Cancer. 10](#_Toc100567647)

[Table S6-2. Hazard ratios from Negative Deleted Model Main18&11.ND using Five-Cancer. 11](#_Toc100567648)

[Table S6-3. Hazard ratios from Variable Selected Model Main18&11.VS using Five-Cancer. 12](#_Toc100567649)

[Table S7-1. The AUCs using OM, NDM, and VSM and the differences based on the **Breast.F.65** validation set 13](#_Toc100567650)

[Table S7-2. The AUCs using OM, NDM, and VSM and the differences based on the **CRC.M.65** validation set 14](#_Toc100567651)

[Table S7-3. The AUCs using OM, NDM, and VSM the differences based on the **CRC.F.65** validation set 15](#_Toc100567652)

[Table S7-4. The AUCs using OM, NDM, and VSM and the differences based on the **Liver.M.65** validation set 16](#_Toc100567653)

[Table S7-5. The AUCs using OM, NDM, and VSM and the differences based on the **Liver.F.65** validation set 17](#_Toc100567654)

[Table S7-6. The AUCs using OM, NDM, and VSM and the differences based on the **Lung.M.65** validation set 18](#_Toc100567655)

[Table S7-7. The AUCs using OM, NDM, and VSM and the differences based on the **Lung.F.65** validation set 19](#_Toc100567656)

[Table S7-8. The AUCs using OM, NDM, and VSM and the differences based on the **Oral.M.65** validation set 20](#_Toc100567657)

[Table S7-9. The AUCs using OM, NDM, and VSM and the differences based on the **Oral.F.65** validation set 21](#_Toc100567658)

[Table S7-10. Comparison of the AUCs based on the validation and test sets for the model Main18&11.ND. 22](#_Toc100567659)

[Table S8. Algorithm for mapping stage at diagnosis from TNM to SEER Summary Stage (localized, regional, and distant): 23](#_Toc100567660)

[Table S9-1. Number of patients by age, stage, and comorbidity level: **Breast Cancer** 25](#_Toc100567661)

[Table S9-2. Number of patients by age, stage, and comorbidity level: **Colorectal Cancer** 26](#_Toc100567662)

[Table S9-3. Number of patients by age, stage, and comorbidity level: **Liver Cancer** 27](#_Toc100567663)

[Table S9-4. Number of patients by age, stage, and comorbidity level: **Lung Cancer** 28](#_Toc100567664)

[Table S9-5. Number of patients by age, stage, and comorbidity level: **Oral Cancer** 29](#_Toc100567665)

[Table S10-1. Numbers of cancer patients in TCR by year of diagnosis and age of diagnosis and the number and percentage of them in TCRLF: **breast cancer** 30](#_Toc100567666)

[Table S10-2. Numbers of cancer patients in TCR by year of diagnosis, age of diagnosis and sex and the number and percentage of them in TCRLF: **colorectal cancer** 31](#_Toc100567667)

[Table S10-3. Numbers of cancer patients in TCR by year of diagnosis, age of diagnosis and sex and the number and percentage of them in TCRLF: **liver cancer** 32](#_Toc100567668)

[Table S10-4. Numbers of cancer patients in TCR by year of diagnosis, age of diagnosis and sex and the number and percentage of them in TCRLF: **lung cancer** 33](#_Toc100567669)

[Table S10-5. Numbers of cancer patients in TCR by year of diagnosis, age of diagnosis and sex and the number and percentage of them in TCRLF: **oral cancer** 34](#_Toc100567670)

[Table S11-1. Number and percentage of patients by stage and year of diagnosis: **breast cancer.** 35](#_Toc100567671)

[Table S11-2. Number and percentage of patients by stage and year of diagnosis: **colorectal cancer**. 35](#_Toc100567672)

[Table S11-3. Number and percentage of patients by stage and year of diagnosis: **liver cancer** 36](#_Toc100567673)

[Table S11-4. Number and percentage of patients by stage and year of diagnosis: **lung cancer.** 36](#_Toc100567674)

[Table S11-5. Number and percentage of patients by stage and year of diagnosis: **oral cancer.** 37](#_Toc100567675)

[Table S12-1-1. Number of patients and five-year probabilities and the confidence intervals of dying from cancer, dying from other-causes, and survival by stage, age and comorbidity level: **female breast cancer** 38](#_Toc100567676)

[Table S12-1-2. One and two-year probabilities and their confidence intervals of dying from cancer, dying from other-causes, and survival by stage, age, and comorbidity level: **female breast cancer** 39](#_Toc100567677)

[Table S12-2-1. Number of patients and five-year probabilities of dying from cancer, dying from other-causes, and survival by stage, age and comorbidity level: **male colorectal cancer** 41](#_Toc100567678)

[Table S12-2-2. One and Two-year probabilities of dying from cancer, dying from other-causes, and survival by stage, age and comorbidity level: **male colorectal cancer** 42](#_Toc100567679)

[Table S12-3-1. Number of patients and five-year probabilities of dying from cancer, dying from other-causes, and survival by stage, age and comorbidity level: **female colorectal cancer** 44](#_Toc100567680)

[Table S12-3-2. One and two-year probabilities of dying from cancer, dying from other-causes, and survival by stage, age and comorbidity level: **female colorectal cancer** 45](#_Toc100567681)

[Table S12-4-1. Number of patients and five-year probabilities of dying from cancer, dying from other-causes, and survival by stage, age and comorbidity level: **male liver cancer** 47](#_Toc100567682)

[Table S12-4-2. One and two-year probabilities of dying from cancer, dying from other-causes, and survival by stage, age and comorbidity level for **male liver cancer** 48](#_Toc100567683)

[Table S12-5-1. Number of patients and five-year probabilities of dying from cancer, dying from other-causes, and survival by stage, age and comorbidity level: **female liver cancer** 50](#_Toc100567684)

[Table S12-5-2. One and two-year probabilities of dying from cancer, dying from other-causes, and survival by stage, age and comorbidity level: **female liver cancer** 51](#_Toc100567685)

[Table S12-6-1. Number of patients and five-year probabilities of dying from cancer, dying from other-causes, and survival by stage, age and comorbidity level: **male lung cancer** 53](#_Toc100567686)

[Table S12-6-2. One and two-year probabilities of dying from cancer, dying from other-causes, and survival by stage, age and comorbidity level: **male lung cancer** 54](#_Toc100567687)

[Table S12-7-1. Number of patients and five-year probabilities of dying from cancer, dying from other-causes, and survival by stage, age and comorbidity level: **female lung cancer** 56](#_Toc100567688)

[Table S12-7-2. One and Two -year probabilities of dying from cancer, dying from other-causes, and survival by stage, age and comorbidity level:  **female lung cancer** 57](#_Toc100567689)

[Table S12-8-1. Number of patients and five-year probabilities of dying from cancer, dying from other-causes, and survival by stage, age and comorbidity level: **male oral cancer** 59](#_Toc100567690)

[Table S12-8-2. One and two-year probabilities of dying from cancer, dying from other-causes, and survival by stage, age and comorbidity level: **male oral cancer** 60](#_Toc100567691)

[Table S12-9-1. Number pf patients and five-year probabilities of dying from cancer, dying from other-causes, and survival by stage, age and comorbidity level: **female oral cancer** 62](#_Toc100567692)

[Table S12-9-2. One and two-year probabilities of dying from cancer, dying from other-causes, and survival by stage, age and comorbidity level: **female oral cancer** 63](#_Toc100567693)

[Table S13-1. Number of patients and probabilities of dying from cancer, dying from other-causes, and survival among patients who were diagnosed with distant lung cancer at ages 30—94 in 2004—2014 by subtypes and comorbidity level 65](#_Toc100567694)

[Table S13-2. Number of patients and probabilities of dying from cancer, dying from other-causes, and survival among patients who were diagnosed with distant lung ADC at ages 30—94 by comorbidity level for period 2004—2010 and 2011—2014 67](#_Toc100567695)

[Table S14-1. Number of patients and **one**-year probabilities of dying from cancer, dying from other-causes, and survival among **males** who were diagnosed with squamous cell carcinoma lung cancer **(SCC)** by stage, age and comorbidity level (n>100) 69](#_Toc100567696)

[Table S14-2. Number of patients and **two**-year probabilities of dying from cancer, dying from other-causes, and survival among **males** who were diagnosed with squamous cell carcinoma lung cancer **(SCC)** by stage, age and comorbidity level (n>100) 70](#_Toc100567697)

[Table S14-3: Number of patients and **five**-year probabilities of dying from cancer, dying from other-causes, and survival among **males** who were diagnosed with squamous cell carcinoma lung cancer **(SCC)** by stage, age and comorbidity level (n>100) 72](#_Toc100567698)

[Table S14-4: Number of patients and **one**-year probabilities of dying from cancer, dying from other-causes, and survival among **males** who were diagnosed with lung adenocarcinoma **(ADC)** by stage, age and comorbidity level (n>100) 73](#_Toc100567699)

[Table S14-5: Number of patients and **two**-year probabilities of dying from cancer, dying from other-causes, and survival among **males** who were diagnosed with lung adenocarcinoma **(ADC)** by stage, age and comorbidity level (n>100) 75](#_Toc100567700)

[Table S14-6: Number of patients and **five**-year probabilities of dying from cancer, dying from other-causes, and survival among **males** who were diagnosed with lung adenocarcinoma **(ADC)** by stage, age and comorbidity level (n>100) 76](#_Toc100567701)

[Table S14-7. Number of patients and **one**-year probabilities of dying from cancer, dying from other-causes, and survival among **females** who were diagnosed with lung Adenocarcinoma **(ADC)** by stage, age and comorbidity level (n>100) 78](#_Toc100567702)

[Table S14-8: Number of patients and **two**-year probabilities of dying from cancer, dying from other-causes, and survival among **females** who were diagnosed with lung Adenocarcinoma **(ADC)** by stage, age and comorbidity level (n>100) 79](#_Toc100567703)

[Table S14-9. Number of patients and **five**-year probabilities of dying from cancer, dying from other-causes, and survival among **females** who were diagnosed with lung Adenocarcinoma **(ADC)** by stage, age and comorbidity level (n>100) 81](#_Toc100567704)

[Figure S1-1. Prevalence of cancer survivors by calendar year and years from diagnosis: **All cancer** 83](#_Toc100567705)

[Figure S1-2. Prevalence of cancer survivors by calendar year and years from diagnosis: **Breast Cancer** 83](#_Toc100567706)

[Figure S1-3. Prevalence of cancer survivors by calendar year and years from diagnosis: **Colorectal Cancer** 84](#_Toc100567707)

[Figure S1-4. Prevalence of cancer survivors by calendar year and years from diagnosis: **Liver Cancer** 84](#_Toc100567708)

[Figure S1-5. Prevalence of cancer survivors by calendar year and years from diagnosis: **Lung Cancer** 85](#_Toc100567709)

[Figure S1-6. Prevalence of cancer survivors by calendar year and years from diagnosis: **Oral Cancer** 85](#_Toc100567710)

[Figure S2-1. Probabilities of dying from cancer, dying from other causes, and survival stratified by stage, comorbidity level, and age for **breast cancer** 86](#_Toc100567711)

[Figure S2-2. Probabilities of dying from cancer, dying from other causes, and survival stratified by stage, comorbidity level, and age for **colorectal cancer** 87](#_Toc100567712)

[Figure S2-3. Probabilities of dying from cancer, dying from other causes, and survival stratified by stage, comorbidity level, and age for **liver cancer** 89](#_Toc100567713)

[Figure S2-4. Probabilities of dying from cancer, dying from other causes, and survival stratified by stage, comorbidity level, and age for **lung cancer** 91](#_Toc100567714)

[Figure S2-5. Probabilities of dying from cancer, dying from other causes, and survival stratified by stage, comorbidity level, and age for **oral cancer** 93](#_Toc100567715)

[Figure S3-1. One-year probabilities of dying from cancer, dying from other causes, and survival stratified by comorbidity level and subtype for distant **lung cancer** patients ages 30—94. 95](#_Toc100567716)

[Figure S3-2. Two-year probabilities of dying from cancer, dying from other causes, and survival stratified by comorbidity level and subtype for distant **lung** **cancer** patients ages 30—94. 95](#_Toc100567717)

[Figure S3-3. Five-year probabilities of dying from cancer, dying from other causes, and survival stratified by comorbidity level and subtype for distant **lung cancer** patients ages 30—94. 96](#_Toc100567718)

[Figure S4-1. One-year probabilities of dying from cancer, dying from other causes, and survival stratified by comorbidity level and year of diagnosis for distant **lung ADC** patients ages 30—94. 97](#_Toc100567719)

[Figure S4-2. Two-year probabilities of dying from cancer, dying from other causes, and survival stratified by comorbidity level and year of diagnosis for distant **lung ADC** patients ages 30—94. 97](#_Toc100567720)

[Figure S4-3. Five-year probabilities of dying from cancer, dying from other causes, and survival are stratified by comorbidity level and year of diagnosis for distant **lung ADC** patients ages 30—94. 98](#_Toc100567721)

[Figure S5. Probabilities of dying from cancer, dying from other causes and survival stratified by stage, comorbidity level, age, and certain specific sex-subtypes for **lung cancer** 99](#_Toc100567722)

## Table S1. Definitions of the cancers in this study

| Cancer | ICD-9 | ICD-10 |
| --- | --- | --- |
| All cancer | 140-199 | C00-C97 |
| Breast | 174,175 | C50 |
| Colorectal | 153,154.0-154.1,159.0 | C18-C20 |
| Lung | 162 | C33, C34 |
| Oral | 140-149 | C00-C14 |
| Liver | 155 | C22 |

In Taiwan Cancer Registry, cancer sites were supplied with the anatomical site coded to the ninth revision of the International Classification of Disease (ICD-9) and in Taiwan Cancer Registry Long Form, they were coded to the tenth revision of the International Classification of Disease (ICD-10).

## Table S2. Numbers of patients diagnosed with cancer of the breast, colon and rectum, liver, lung, and oral reported in the TCR from 2004 to 2014 by gender and age of diagnosis.

|  | Breast | Colorectal | Liver | Lung | Oral |
| --- | --- | --- | --- | --- | --- |
| All-TCR^1^ | 92615 | 119315 | 113028 | 101760 | 76941 |
| All-NHIRD^2^ | 92343 | 118755 | 112487 | 101244 | 76743 |
| NHIRD/TCR (%) | 99.7 | 99.5 | 99.5 | 99.5 | 99.7 |
| Gender |  |  |  |  |  |
| Male |  | 68391 | 79563 | 64273 | 66998 |
| Female | 92343 | 50364 | 32924 | 36971 | 9745 |
| Age |  |  |  |  |  |
| 15-64 |  |  |  |  |  |
| Male |  | 30385 | 44505 | 21089 | 55393 |
| Female | 75609 | 22599 | 10781 | 15959 | 6999 |
| 65-94 |  |  |  |  |  |
| Male |  | 38006 | 35058 | 43184 | 11605 |
| Female | 16734 | 27765 | 22143 | 21012 | 2746 |

^1^All-TCR means the total number of cases in the 2004-2014 TCR ages 15-94; in this paper, only the first primary cancer of each patient was considered.

^2^All-NHIRD is the number of cases in All-TCR and linked to Taiwan NHIRD.

## Table S3. Definitions of comorbidities+

| No. | Disease | ICD-9-CM |
| --- | --- | --- |
| 1 | Acute Myocardial Infraction, AMI | 410.x |
| 2 | Old Myocardial Infraction, Old MI | 412.x |
| 3 | Congestive heart failure, CHF | 428.x |
| 4 | Peripheral vascular disease, PVD | 441.x,443.9,785.4,V43.4,  Procedure 38.48 |
| 5 | Cerebrovascular disease, CVD | 430.x-438.x |
| 6 | Chronic obstructive pulmonary disease, COPD | 490.x-505x,506.4 |
| 7 | Dementia | 290.x |
| 8 | Paralysis | 342.x,344.1 |
| 9 | Diabetes without chronic complication, DM W/O CC | 250.0-250.3,250.7 |
| 10 | Diabetes with chronic complication, DM W CC | 250.4-250.6,250.8-250.9 |
| 11 | Chronic renal failure (renal disease), CRF | 582.x, 583.x, 585.x, 586.x, 588.x |
| 12* | Cirrhosis/chronic hepatitis (Mild liver disease), HBV, HCV, Mild LD | 571.2,571.4,571.5,571.6  070.2-070.7(excluded 070.41,070.51),070.9, V026.1 |
| 13 | Moderate-Server Liver Disease, M S LD | 572.2-572.8,456.0-456.21 |
| 14 | Ulcer (Peptic ulcer disease) | 531.x-534.x |
| 15 | Rheumatic disease, RD | 710.0,710.1,710.4,714.0-714.2,714.81, 725.x |
| 16 | AIDS | 042.x-044x |
| 17** | Hypertension, uncomplicated, HT UC | 401.1, 401.9, 642.0 |
| 18** | Hypertension, complicated, HT C | 401.0, 402.x-405.x, 642.1, 642.2, 642.7, 642.9 |

+Deyo RA, Cherkin DC, Ciol MA. Adapting a clinical comorbidity index for use with ICD-8-CM administrative databases. J Clin Epidemiol.1992; 45:613-9

Quan H, Sundararajan V, Halfon P, et al. Coding algorithms for defining Comorbidities in ICD-9-CM and ICD-10 administrative data. Med Care. 2005 Nov; 43(11): 1130-9.

*The definition of the Cirrhosis/chronic hepatitis (Mild liver disease) comorbidity in this study includes viral hepatitis B and viral hepatitis C, which differs from the one that used by Charlson in 1987.

**Elixhauser AHRQ-Web ICD-9-CＭ

## Table S4-1. The hazard ratio of each comorbidity with respect to death due to other causes, among colorectal cancer patients, ages 15-64.

|  | Age 15-64 | | | | | | | | | | | | | | | | |
| --- | --- | --- | --- | --- | --- | --- | --- | --- | --- | --- | --- | --- | --- | --- | --- | --- | --- |
|  |  |  |  |  |  |  |  |  |  | K=5 | | | | | | | |
|  | K^+^=5 | | |  | K=13 | | |  |  | Male | | |  | Female | | |  |
| Comorbidity | HR* | 95CI | |  | HR | 95CI | | Comparison^#^ | | HR | 95CI | |  | HR | 95CI | | Comparison |
| Condition1 | 2.37 | 1.37 | 4.10 |  | 2.18 | 1.43 | 3.33 | 4 |  | 1.73 | 0.90 | 3.34 |  | 15.14 | 5.64 | 40.65 | 1 |
| Condition2 | 2.71 | 1.60 | 4.59 |  | 2.24 | 1.39 | 3.62 | 4 |  | 2.28 | 1.26 | 4.14 |  | 9.53 | 3.05 | 29.73 | 2 |
| Condition3 | 5.16 | 4.07 | 6.53 |  | 5.01 | 4.14 | 6.07 | 4 |  | 4.34 | 3.22 | 5.84 |  | 7.64 | 5.15 | 11.33 | 2 |
| Condition4 | 3.23 | 1.97 | 5.29 |  | 2.96 | 2.11 | 4.14 | 4 |  | 2.75 | 1.48 | 5.14 |  | 4.59 | 2.05 | 10.28 | 4 |
| Condition5 | 2.99 | 2.49 | 3.59 |  | 2.82 | 2.42 | 3.30 | 4 |  | 2.83 | 2.29 | 3.50 |  | 3.58 | 2.50 | 5.13 | 3 |
| Condition6 | 1.63 | 1.33 | 1.98 |  | 1.39 | 1.20 | 1.62 | 3 |  | 1.70 | 1.35 | 2.14 |  | 1.45 | 0.99 | 2.13 | 4 |
| Condition7 | 2.65 | 0.37 | 18.86 |  | 1.19 | 0.17 | 8.48 | 4 |  | 3.79 | 0.53 | 26.94 |  | 0.00 | 0.00 | Inf | 3 |
| Condition8 | 4.47 | 2.77 | 7.22 |  | 3.92 | 2.80 | 5.49 | 4 |  | 4.89 | 2.94 | 8.15 |  | 2.74 | 0.68 | 11.00 | 3 |
| Condition9 | 2.33 | 2.06 | 2.63 |  | 2.37 | 2.11 | 2.66 | 4 |  | 2.08 | 1.80 | 2.40 |  | 3.08 | 2.46 | 3.84 | 1 |
| Condition10 | 3.36 | 2.84 | 3.98 |  | 3.00 | 2.59 | 3.48 | 4 |  | 2.89 | 2.34 | 3.55 |  | 4.79 | 3.58 | 6.40 | 1 |
| Condition11 | 5.07 | 4.22 | 6.09 |  | 4.16 | 3.51 | 4.93 | 2 |  | 4.34 | 3.46 | 5.44 |  | 7.29 | 5.33 | 9.98 | 2 |
| Condition12 | 1.74 | 1.49 | 2.04 |  | 1.62 | 1.42 | 1.84 | 4 |  | 1.73 | 1.44 | 2.08 |  | 1.75 | 1.27 | 2.40 | 4 |
| Condition13 | 12.76 | 8.77 | 18.56 |  | 11.03 | 7.92 | 15.38 | 4 |  | 14.36 | 9.86 | 20.92 |  | 0.00 | 0.00 | Inf | 3 |
| Condition14 | 1.56 | 1.33 | 1.83 |  | 1.41 | 1.24 | 1.60 | 4 |  | 1.68 | 1.40 | 2.02 |  | 1.27 | 0.92 | 1.75 | 3 |
| Condition15 | 2.64 | 1.61 | 4.32 |  | 2.10 | 1.41 | 3.13 | 4 |  | 1.46 | 0.47 | 4.54 |  | 3.24 | 1.86 | 5.63 | 3 |
| Condition16 | 6.23 | 1.56 | 24.93 |  | 5.19 | 1.30 | 20.77 | 4 |  | 7.77 | 1.94 | 31.12 |  | 0.00 | 0.00 | Inf | 3 |
| Condition17 | 1.47 | 1.31 | 1.65 |  | 1.70 | 1.52 | 1.89 | 2 |  | 1.41 | 1.23 | 1.62 |  | 1.63 | 1.31 | 2.03 | 3 |
| Condition18 | 2.22 | 1.92 | 2.56 |  | 2.08 | 1.83 | 2.36 | 4 |  | 1.99 | 1.67 | 2.36 |  | 2.85 | 2.22 | 3.67 | 2 |

^*^Obtained by fitting a Cox’s regression model with only one comorbidity as the covariate of interest, adjusted for age and sex.

^#^1 if there was no overlap between the two confidence intervals; 2 if there was overlap but neither of the two point estimates was in the confidence interval of the other; 3 if exactly one of the point estimates was in the confidence interval of the other; 4 if each point estimate was in the confidence interval of the other.

^+^The number of half-years used in assessing the comorbidities.

## Table S4-2. The hazard ratio of each comorbidity with respect to death due to other causes, among colorectal cancer patients, ages 65-94

|  | Age 65-94 | | | | | | | | | | | | | | | | |
| --- | --- | --- | --- | --- | --- | --- | --- | --- | --- | --- | --- | --- | --- | --- | --- | --- | --- |
|  |  |  |  |  |  |  |  |  |  | K=5 | | | | | | | |
|  | K^+^=5 | | |  | K=13 | | |  |  | Male | | |  | Female | | |  |
| Comorbidity | HR* | 95CI | |  | HR | 95CI | | Comparison^#^ | | HR | 95CI | |  | HR | 95CI | | Comparison^#^ |
| Condition1 | 2.16 | 1.82 | 2.58 |  | 1.99 | 1.76 | 2.25 | 4 |  | 2.05 | 1.66 | 2.52 |  | 2.50 | 1.81 | 3.46 | 4 |
| Condition2 | 1.56 | 1.27 | 1.93 |  | 1.67 | 1.43 | 1.96 | 4 |  | 1.64 | 1.29 | 2.09 |  | 1.36 | 0.89 | 2.07 | 4 |
| Condition3 | 2.23 | 2.07 | 2.39 |  | 2.10 | 1.97 | 2.23 | 3 |  | 2.22 | 2.02 | 2.44 |  | 2.22 | 1.99 | 2.47 | 4 |
| Condition4 | 1.65 | 1.43 | 1.91 |  | 1.61 | 1.45 | 1.78 | 4 |  | 1.45 | 1.19 | 1.75 |  | 2.09 | 1.66 | 2.62 | 2 |
| Condition5 | 1.80 | 1.70 | 1.90 |  | 1.74 | 1.66 | 1.83 | 4 |  | 1.73 | 1.62 | 1.86 |  | 1.92 | 1.76 | 2.10 | 2 |
| Condition6 | 1.47 | 1.39 | 1.55 |  | 1.37 | 1.30 | 1.43 | 2 |  | 1.54 | 1.44 | 1.64 |  | 1.36 | 1.24 | 1.50 | 2 |
| Condition7 | 2.24 | 2.05 | 2.44 |  | 2.15 | 1.98 | 2.32 | 4 |  | 2.35 | 2.09 | 2.65 |  | 2.08 | 1.83 | 2.36 | 3 |
| Condition8 | 2.23 | 1.86 | 2.68 |  | 2.55 | 2.26 | 2.89 | 3 |  | 2.14 | 1.70 | 2.70 |  | 2.40 | 1.79 | 3.23 | 4 |
| Condition9 | 1.73 | 1.64 | 1.81 |  | 1.73 | 1.65 | 1.81 | 4 |  | 1.66 | 1.56 | 1.77 |  | 1.84 | 1.70 | 1.99 | 2 |
| Condition10 | 2.07 | 1.93 | 2.21 |  | 2.08 | 1.96 | 2.20 | 4 |  | 1.89 | 1.72 | 2.07 |  | 2.35 | 2.12 | 2.61 | 1 |
| Condition11 | 2.60 | 2.42 | 2.79 |  | 2.16 | 2.02 | 2.29 | 1 |  | 2.26 | 2.07 | 2.47 |  | 3.44 | 3.07 | 3.87 | 1 |
| Condition12 | 1.25 | 1.15 | 1.37 |  | 1.14 | 1.06 | 1.22 | 2 |  | 1.16 | 1.03 | 1.30 |  | 1.41 | 1.23 | 1.63 | 2 |
| Condition13 | 5.93 | 4.50 | 7.81 |  | 3.88 | 3.08 | 4.87 | 2 |  | 4.12 | 2.78 | 6.11 |  | 10.19 | 6.92 | 15.02 | 1 |
| Condition14 | 1.26 | 1.19 | 1.33 |  | 1.19 | 1.14 | 1.25 | 3 |  | 1.30 | 1.21 | 1.39 |  | 1.20 | 1.09 | 1.31 | 3 |
| Condition15 | 1.48 | 1.19 | 1.83 |  | 1.30 | 1.11 | 1.52 | 4 |  | 1.40 | 1.01 | 1.93 |  | 1.57 | 1.17 | 2.10 | 4 |
| Condition16 | 1.63 | 0.23 | 11.55 |  | 1.54 | 0.22 | 10.94 | 4 |  | 1.67 | 0.23 | 11.83 |  | - | - | - | - |
| Condition17 | 1.26 | 1.21 | 1.32 |  | 1.38 | 1.31 | 1.44 | 2 |  | 1.28 | 1.21 | 1.36 |  | 1.23 | 1.14 | 1.32 | 4 |
| Condition18 | 1.30 | 1.24 | 1.37 |  | 1.38 | 1.32 | 1.45 | 2 |  | 1.25 | 1.17 | 1.33 |  | 1.40 | 1.29 | 1.51 | 2 |

^*^Obtained by fitting a Cox’s regression model with only one comorbidity as the covariate of interest, adjusted for age and sex.

^#^1 if there was no overlap between the two confidence intervals; 2 if there was overlap but neither of the two point estimates was in the confidence interval of the other; 3 if exactly one of the point estimates was in the confidence interval of the other; 4 if each point estimate was in the confidence interval of the other.

^+^The number of half-years used in assessing the comorbidities.

## Table S5-1. The number and percentage of patients with comorbid conditions for each of the 5 cancer cohorts and non-cancer cohorts, ages 15-64

| Ages 15-64 | **Breast** | | **Colorectal** | | | | **Liver** | | | | | **Lung** | | | | | | | | | | | **Oral** | | | | | | | | | | | | **Noncancer cohort** | | | | | | | | | | | |
| --- | --- | --- | --- | --- | --- | --- | --- | --- | --- | --- | --- | --- | --- | --- | --- | --- | --- | --- | --- | --- | --- | --- | --- | --- | --- | --- | --- | --- | --- | --- | --- | --- | --- | --- | --- | --- | --- | --- | --- | --- | --- | --- | --- | --- | --- | --- |
|  | Female | | Male | | Female | | Male | | Female | | | Male | | | | | Female | | | | | | Male | | | | | | Female | | | | | | Male | | | | | Female | | | | | | |
|  | N | % | N | % | N | % | N | % | N | % | | N | | % | | | N | | | % | | | N | | | % | | | N | | | % | | | N | | | % | | N | | | % | | | |
| **Number Alive^*^** | 63551 | 84.1 | 19069 | 62.8 | 14947 | 66.1 | 11528 | 25.9 | 3444 | 32.0 | | 4201 | | 19.9 | | | 5229 | | | 32.8 | | | 30012 | | | 54.2 | | | 5137 | | | 73.4 | | | NA  NA  NA | | | NA  NA  NA | | NA  NA  NA | | | NA  NA  NA | | | |
| **Cancer Deaths** | 10729 | 14.2 | 10057 | 33.1 | 7114 | 31.5 | 31294 | 70.3 | 6879 | 63.8 | | 16191 | | 76.8 | | | 10407 | | | 65.2 | | | 22299 | | | 40.3 | | | 1619 | | | 23.1 | | |  |  |  |  |  |  |  |  |  |  |  |  |
| **Other-Cause Deaths** | 1329 | 1.8 | 1259 | 4.1 | 538 | 2.4 | 1683 | 3.8 | 458 | 4.3 | | 697 | | 3.3 | | | 323 | | | 2.0 | | | 3082 | | | 5.6 | | | 243 | | | 3.5 | | |  |  |  |  |  |  |  |  |  |  |  |  |
| **Number of Comorbid conditions (%)** | | | | | | | | | | | | | | | | | | | | | | | | | | | | | | | | | | | | | | | | | | | | | | |
| **Comorbid conditions (%)** | N | % | N | % | N | % | N | % | N | % | N | | % | | N | | | % | | | N | | | % | | | N | | | % | | | N | | | % | | | N | | % | | |  |  |  |
| 0 | 55560 | 73.5 | 18146 | 59.7 | 14826 | 65.6 | 19057 | 42.8 | 3513 | 32.6 | 12852 | | 60.9 | | 10095 | | | 63.3 | | | 37733 | | | 68.1 | | | 4910 | | | 70.2 | | | 785729  90586  35578  12988  6986 | | | 84.3  9.7  3.8  1.4  0.7 | | | 798789  80235  27788  9705  4896 | | 86.7  8.7  3.0  1.1  0.5 | | |  |  |  |
| 1 | 12363 | 16.4 | 6179 | 20.3 | 4358 | 19.3 | 9185 | 20.6 | 2459 | 22.8 | 4094 | | 19.4 | | 3362 | | | 21.1 | | | 8699 | | | 15.7 | | | 1182 | | | 16.9 | | |  |  |  |  |  |  |  |  |  |  |  |  |  |  |
| 2 | 5050 | 6.7 | 3585 | 11.8 | 2079 | 9.2 | 7611 | 17.1 | 2156 | 20.0 | 2326 | | 11.0 | | 1535 | | | 9.6 | | | 5144 | | | 9.3 | | | 579 | | | 8.3 | | |  |  |  |  |  |  |  |  |  |  |  |  |  |  |
| 3 | 1726 | 2.3 | 1484 | 4.9 | 838 | 3.7 | 4787 | 10.8 | 1389 | 12.9 | 1130 | | 5.4 | | 632 | | | 4.0 | | | 2371 | | | 4.3 | | | 213 | | | 3.0 | | |  |  |  |  |  |  |  |  |  |  |  |  |  |  |
| 4+ | 910 | 1.2 | 991 | 3.3 | 498 | 2.2 | 3865 | 8.7 | 1264 | 11.7 | 687 | | 3.3 | | 335 | | | 2.1 | | | 1446 | | | 2.6 | | | 115 | | | 1.6 | | |  |  |  |  |  |  |  |  |  |  |  |  |  |  |
| **Comorbid Conditions** | | | | | | | | | | | | | | | | | | | | | | | | | | | | | | | | | | | | | | | | | | | | | | |
| **AMI** | 44 | 0.06 | 131 | 0.43 | 17 | 0.08 | 138 | 0.31 | 7 | 0.06 | 104 | | 0.49 | | | 16 | | | 0.1 | | | 165 | | | 0.30 | | | <5 | | | NA | | | 1469 | | | 0.16 | | 266 | | | 0.03 | | | |  |
| **OLD MI** | 27 | 0.04 | 110 | 0.36 | 19 | 0.08 | 91 | 0.2 | 7 | 0.06 | 92 | | 0.44 | | | 9 | | | 0.06 | | | 129 | | | 0.23 | | | <5 | | | NA | | | 986 | | | 0.11 | | 162 | | | 0.02 | | | |  |
| **CHF** | 296 | 0.39 | 347 | 1.14 | 173 | 0.77 | 500 | 1.12 | 194 | 1.8 | 243 | | 1.15 | | | 103 | | | 0.65 | | | 470 | | | 0.85 | | | 29 | | | 0.41 | | | 2968 | | | 0.32 | | 1886 | | | 0.20 | | | |  |
| **PVD** | 208 | 0.28 | 115 | 0.38 | 73 | 0.32 | 244 | 0.55 | 65 | 0.6 | 108 | | 0.51 | | | 73 | | | 0.46 | | | 196 | | | 0.35 | | | 28 | | | 0.4 | | | 1412 | | | 0.15 | | 1264 | | | 0.14 | | | |  |
| **CVD** | 928 | 1.23 | 1071 | 3.52 | 486 | 2.15 | 1400 | 3.15 | 344 | 3.19 | 736 | | 3.49 | | | 335 | | | 2.1 | | | 1581 | | | 2.85 | | | 123 | | | 1.76 | | | 9963 | | | 1.07 | | 6062 | | | 0.66 | | | |  |
| **COPD** | 2585 | 3.42 | 1297 | 4.27 | 954 | 4.22 | 2213 | 4.97 | 682 | 6.33 | 1702 | | 8.07 | | | 1098 | | | 6.88 | | | 2057 | | | 3.71 | | | 322 | | | 4.6 | | | 18761 | | | 2.01 | | 18842 | | | 2.04 | | | |  |
| **Dementia** | 11 | 0.01 | 20 | 0.07 | <5 | NA | 23 | 0.05 | 5 | 0.05 | 15 | | 0.07 | | | <5 | | | NA | | | 16 | | | 0.03 | | | 0 | | | 0 | | | 131 | | | 0.01 | | 73 | | | 0.01 | | | |  |
| **Paralysis** | 59 | 0.08 | 106 | 0.35 | 40 | 0.18 | 151 | 0.34 | 24 | 0.22 | 83 | | 0.39 | | | 20 | | | 0.13 | | | 202 | | | 0.36 | | | 12 | | | 0.17 | | | 1326 | | | 0.14 | | 489 | | | 0.05 | | | |  |
| **DM W/O CC** | 4322 | 5.72 | 3847 | 12.66 | 2226 | 9.85 | 8176 | 18.37 | 2197 | 20.38 | 2270 | | 10.76 | | | 1289 | | | 8.08 | | | 5330 | | | 9.62 | | | 520 | | | 7.43 | | | 31686 | | | 3.40 | | 24493 | | | 2.66 | | | |  |
| **DM W CC** | 1160 | 1.53 | 1119 | 3.68 | 621 | 2.75 | 2408 | 5.41 | 767 | 7.11 | 660 | | 3.13 | | | 338 | | | 2.12 | | | 1493 | | | 2.7 | | | 140 | | | 2.00 | | | 8510 | | | 0.91 | | 6383 | | | 0.69 | | | |  |
| **CRF** | 729 | 0.96 | 648 | 2.13 | 362 | 1.60 | 1464 | 3.29 | 460 | 4.27 | 404 | | 1.92 | | | 179 | | | 1.12 | | | 714 | | | 1.29 | | | 162 | | | 2.31 | | | 5217 | | | 0.56 | | 3897 | | | 0.42 | | | |  |
| **Mild LD** | 3512 | 4.64 | 2308 | 7.6 | 1188 | 5.26 | 17960 | 40.36 | 5182 | 48.07 | 1619 | | 7.68 | | | 1023 | | | 6.41 | | | 4713 | | | 8.51 | | | 365 | | | 5.22 | | | 36265 | | | 3.89 | | 21749 | | | 2.36 | | | |  |
| **M S LD** | 34 | 0.04 | 98 | 0.32 | 11 | 0.05 | 3091 | 6.95 | 762 | 7.07 | 74 | | 0.35 | | | 11 | | | 0.07 | | | 615 | | | 1.11 | | | 11 | | | 0.16 | | | 1130 | | | 0.12 | | 228 | | | 0.02 | | | |  |
| **Ulcer** | 4513 | 5.97 | 2234 | 7.35 | 1608 | 7.12 | 6978 | 15.68 | 2291 | 21.25 | 1751 | | 8.3 | | | 1414 | | | 8.86 | | | 3967 | | | 7.16 | | | 444 | | | 6.34 | | | 30792 | | | 3.3 | | 30716 | | 3.33 | | | |  |  |
| **RD** | 628 | 0.83 | 63 | 0.21 | 219 | 0.97 | 134 | 0.3 | 171 | 1.59 | 83 | | 0.39 | | | 211 | | | 1.32 | | | 150 | | | 0.27 | | | 85 | | | 1.21 | | | 1157 | | | 0.12 | | 4244 | | 0.46 | | | |  |  |
| **AIDS** | <5** | NA | 15 | 0.05 | <5 | NA | 53 | 0.12 | <5 | NA | 18 | | 0.09 | | | <5 | | | NA | | | 53 | | | 0.1 | | | 0 | | | 0 | | | 1155 | | | 0.12 | | 101 | | 0.01 | | | |  |  |
| **HT UC** | 9303 | 12.3 | 6523 | 21.47 | 3874 | 17.14 | 8636 | 19.4 | 2666 | 24.73 | 3902 | | 18.5 | | | 2775 | | | 17.39 | | | 8198 | | | 14.8 | | | 1033 | | | 14.76 | | | 59287 | | | 6.36 | | 49400 | | 5.36 | | | |  |  |
| **HT C** | 3323 | 4.39 | 2286 | 7.52 | 1463 | 6.47 | 3071 | 6.9 | 1028 | 9.54 | 1402 | | 6.65 | | | 938 | | | 5.88 | | | 2657 | | | 4.8 | | | 330 | | | 4.71 | | | 20263 | | | 2.17 | | 16833 | | 1.83 | | | |  |  |

* Death due to the cancer or other causes was decided by the NCI classification algorithm using the TCOD and TCR from 2004 until Dec. 31, 2016; survival information of patients not included in the TCOD were obtained from the beneficiary registry of NHIRD at Dec. 31, 2015; the latter were all considered alive.

**The percentage is calculated by deleting the cells whose case number <5.

|  |
| --- |

|  |
| --- |

## Table S5-2. The number and percentage of patients with comorbid conditions for each of the 5 cancer cohorts and non-cancer cohorts, ages 65-94

| Age:65-94 | **Breast** | | **Colorectal** | | | | **Liver** | | | | **Lung** | | | | | **Oral** | | | | **Noncancer cohort** | | | |
| --- | --- | --- | --- | --- | --- | --- | --- | --- | --- | --- | --- | --- | --- | --- | --- | --- | --- | --- | --- | --- | --- | --- | --- |
|  | Female | | Male | | Female | | Male | | Female | | Male | | Female | | | Male | | Female | | Male | | Female | |
|  | N | % | N | % | N | % | N | % | N | % | N | % | N | | % | N | % | N | % | N | % | N | % |
| **Number Alive*^*^** | 11231 | 67.1 | 15083 | 39.7 | 12156 | 43.8 | 5638 | 16.1 | 3778 | 17.1 | 3370 | 7.8 | 3389 | | 16.1 | 3867 | 33.3 | 1155 | 42.1 | NA | NA | NA | NA |
| **Cancer Deaths** | 3454 | 20.6 | 16801 | 44.2 | 11922 | 42.9 | 26484 | 75.5 | 16680 | 75.3 | 36896 | 85.4 | 16297 | | 77.6 | 5860 | 50.5 | 1183 | 43.1 | NA | NA | NA | NA |
| **Other-Cause Deaths** | 2049 | 12.2 | 6122 | 16.1 | 3687 | 13.3 | 2936 | 8.4 | 1685 | 7.6 | 2918 | 6.8 | 1326 | | 6.3 | 1878 | 16.2 | 408 | 14.9 | NA | NA | NA | NA |
| **Number of Comorbid conditions (%)** | | | | | | | | | | | | | | | | | | | | | | | |
| **Comorbid conditions (%)** | N | % | N | % | N | % | N | % | N | % | N | % | N | % | | N | % | N | % | N | % | N | % |
| 0 | 4466 | 26.7 | 10664 | 28.1 | 7032 | 25.3 | 6986 | 19.9 | 2862 | 12.9 | 12241 | 28.3 | 5392 | 25.7 | | 3957 | 34.1 | 707 | 25.7 | 45773 | 35.4 | 44662 | 32.1 |
| 1 | 4405 | 26.3 | 9246 | 24.3 | 6995 | 25.2 | 6979 | 19.9 | 4109 | 18.6 | 10277 | 23.8 | 5490 | 26.1 | | 2671 | 23 | 739 | 26.9 | 28781 | 22.3 | 33589 | 24.2 |
| 2 | 3902 | 23.3 | 8304 | 21.8 | 6250 | 22.5 | 8004 | 22.8 | 5223 | 23.6 | 9304 | 21.5 | 4788 | 22.8 | | 2303 | 19.8 | 604 | 22 | 24380 | 18.9 | 28437 | 20.5 |
| 3 | 2177 | 13 | 4966 | 13.1 | 3839 | 13.8 | 6143 | 17.5 | 4362 | 19.7 | 5721 | 13.2 | 2818 | 13.4 | | 1391 | 12 | 379 | 13.8 | 15186 | 11.7 | 16508 | 11.9 |
| 4+ | 1784 | 10.7 | 4826 | 12.7 | 3649 | 13.1 | 6946 | 19.8 | 5587 | 25.2 | 5641 | 13.1 | 2524 | 12 | | 1283 | 11.1 | 317 | 11.5 | 15212 | 11.8 | 15851 | 11.4 |
| **Comorbid Conditions** | | | | | | | | | | | | | | | | | | | | | | | |
| **AMI** | 58 | 0.35 | 400 | 1.05 | 167 | 0.60 | 277 | 0.79 | 112 | 0.51 | 446 | 1.03 | 132 | 0.63 | | 85 | 0.73 | 13 | 0.47 | 1386 | 1.07 | 813 | 0.58 |
| **OLD MI** | 48 | 0.29 | 345 | 0.91 | 138 | 0.50 | 290 | 0.83 | 84 | 0.38 | 512 | 1.19 | 99 | 0.47 | | 94 | 0.81 | 15 | 0.55 | 1248 | 0.96 | 545 | 0.39 |
| **CHF** | 689 | 4.12 | 2004 | 5.27 | 1776 | 6.40 | 1785 | 5.09 | 1573 | 7.10 | 2502 | 5.79 | 1180 | 5.62 | | 464 | 4.00 | 143 | 5.21 | 6477 | 5.01 | 7189 | 5.17 |
| **PVD** | 213 | 1.27 | 594 | 1.56 | 368 | 1.33 | 539 | 1.54 | 334 | 1.51 | 749 | 1.73 | 304 | 1.45 | | 176 | 1.52 | 32 | 1.17 | 1903 | 1.47 | 1896 | 1.36 |
| **CVD** | 1909 | 11.41 | 5699 | 15.00 | 3661 | 13.19 | 4729 | 13.49 | 2612 | 11.80 | 6542 | 15.15 | 2617 | 12.45 | | 1550 | 13.36 | 315 | 11.47 | 18669 | 14.43 | 16942 | 12.18 |
| **COPD** | 1758 | 10.51 | 7056 | 18.57 | 3498 | 12.60 | 6574 | 18.75 | 3077 | 13.90 | 12483 | 28.91 | 3562 | 16.95 | | 2108 | 18.16 | 392 | 14.28 | 22515 | 17.41 | 16181 | 11.64 |
| **Dementia** | 558 | 3.33 | 1246 | 3.28 | 1287 | 4.64 | 967 | 2.76 | 898 | 4.06 | 1356 | 3.14 | 850 | 4.05 | | 269 | 2.32 | 95 | 3.46 | 4661 | 3.60 | 5902 | 4.24 |
| **Paralysis** | 116 | 0.69 | 365 | 0.96 | 238 | 0.86 | 298 | 0.85 | 142 | 0.64 | 390 | 0.90 | 156 | 0.74 | | 125 | 1.08 | 21 | 0.76 | 1342 | 1.04 | 1114 | 0.80 |
| **DM W/O CC** | 4211 | 25.16 | 8092 | 21.29 | 6988 | 25.17 | 9323 | 26.59 | 7159 | 32.33 | 7403 | 17.14 | 4448 | 21.17 | | 2311 | 19.91 | 682 | 24.84 | 22686 | 17.54 | 29347 | 21.11 |
| **DM W CC** | 1399 | 8.36 | 2744 | 7.22 | 2301 | 8.29 | 3224 | 9.20 | 2510 | 11.34 | 2495 | 5.78 | 1406 | 6.69 | | 745 | 6.42 | 210 | 7.65 | 7708 | 5.96 | 9559 | 6.87 |
| **CRF** | 679 | 4.06 | 2430 | 6.39 | 1417 | 5.10 | 2647 | 7.55 | 1499 | 6.77 | 2392 | 5.54 | 814 | 3.87 | | 575 | 4.95 | 116 | 4.22 | 7245 | 5.60 | 5657 | 4.07 |
| **Mild LD** | 1194 | 7.14 | 2281 | 6.00 | 1740 | 6.27 | 13314 | 37.98 | 10620 | 47.96 | 2435 | 5.64 | 1332 | 6.34 | | 893 | 7.69 | 191 | 6.96 | 7774 | 6.01 | 8638 | 6.21 |
| **M S LD** | 32 | 0.19 | 80 | 0.21 | 62 | 0.22 | 1256 | 3.58 | 1308 | 5.91 | 60 | 0.14 | 25 | 0.12 | | 58 | 0.50 | 7 | 0.25 | 279 | 0.22 | 305 | 0.22 |
| **Ulcer** | 2384 | 14.25 | 5978 | 15.73 | 4587 | 16.52 | 7666 | 21.87 | 6041 | 27.28 | 7224 | 16.73 | 3408 | 16.22 | | 1616 | 13.93 | 408 | 14.86 | 18517 | 14.32 | 20357 | 14.64 |
| **RD** | 191 | 1.14 | 205 | 0.54 | 346 | 1.25 | 221 | 0.63 | 366 | 1.65 | 321 | 0.74 | 279 | 1.33 | | 67 | 0.58 | 59 | 2.15 | 743 | 0.57 | 1910 | 1.37 |
| **AIDS** | 0 | 0.00 | <5** | NA | 0 | 0.00 | 13 | 0.04 | <5 | NA | 5 | 0.01 | <5 | NA | | <5 | NA | 0 | 0.00 | 16 | 0.01 | 6 | 0.00 |
| **HT UC** | 7993 | 47.77 | 16559 | 43.57 | 13425 | 48.35 | 15393 | 43.91 | 11457 | 51.74 | 17588 | 40.73 | 10209 | 48.59 | | 4509 | 38.85 | 1326 | 48.29 | 50294 | 38.89 | 61740 | 44.40 |
| **HT C** | 3619 | 21.63 | 7499 | 19.73 | 6284 | 22.63 | 6437 | 18.36 | 5118 | 23.11 | 7847 | 18.17 | 4540 | 21.61 | | 1811 | 15.61 | 568 | 20.68 | 22414 | 17.33 | 27321 | 19.65 |

*Death due to the cancer or other causes was decided by the NCI classification algorithm using the TCOD and TCR from 2004 until Dec. 31, 2016; survival information of patients not included in the TCOD were obtained from the beneficiary registry of NHIRD at Dec. 31, 2015; the latter were all considered alive.

**The percentage is calculated by deleting the cells whose case number <5.

## Table S6-1. Hazard Ratios from Original Model Main18&11 using Five-Cancer.

| Comorbid condition | Coef | HR |  | Comorbid condition | Coef | HR |
| --- | --- | --- | --- | --- | --- | --- |
| Age | 0.06 | 1.07 |  | CVD*Mild LD | -0.13 | 0.88 |
| Sex | 0.49 | 1.63 |  | COPD*Dementia | 0.06 | 1.07 |
| AMI | 0.23 | 1.26 |  | COPD*DM W/O CC | -0.11 | 0.90 |
| Old MI | 0.07 | 1.08 |  | COPD*DM W CC | -0.03 | 0.97 |
| CHF | 0.77 | 2.16 |  | COPD*CRF | -0.19 | 0.83 |
| PVD | 0.23 | 1.26 |  | COPD*Ulcer | -0.02 | 0.98 |
| CVD | 0.40 | 1.49 |  | COPD*HT UC | 0.00 | 1.00 |
| COPD | 0.28 | 1.33 |  | COPD*HT C | 0.09 | 1.09 |
| Dementia | 0.71 | 2.03 |  | COPD*Mild LD | -0.14 | 0.87 |
| Paralysis | 0.34 | 1.41 |  | Dementia*DM W/O CC | -0.03 | 0.97 |
| DM W/O CC | 0.24 | 1.27 |  | Dementia*DM W CC | -0.05 | 0.95 |
| DM W CC | 0.39 | 1.48 |  | Dementia*CRF | -0.33 | 0.72 |
| CRF | 0.78 | 2.18 |  | Dementia*Ulcer | -0.09 | 0.91 |
| Mild LD | -0.07 | 0.94 |  | Dementia*HT UC | -0.04 | 0.96 |
| M S LD | 0.93 | 2.52 |  | Dementia*HT C | -0.05 | 0.95 |
| Ulcer | -0.01 | 0.99 |  | Dementia*Mild LD | -0.17 | 0.85 |
| RD | 0.30 | 1.35 |  | DM W/O CC*DM W CC | -0.12 | 0.88 |
| AIDS | 1.66 | 5.28 |  | DM W/O CC*CRF | 0.03 | 1.03 |
| HT UC | 0.03 | 1.03 |  | DM W/O CC*Ulcer | 0.09 | 1.09 |
| HT C | -0.01 | 0.99 |  | DM W/O CC*HT UC | 0.04 | 1.04 |
| CHF*CVD | -0.11 | 0.90 |  | DM W/O CC*HT C | 0.12 | 1.12 |
| CHF*COPD | -0.12 | 0.89 |  | DM W/O CC*Mild LD | 0.02 | 1.03 |
| CHF*Dementia | -0.31 | 0.73 |  | DM W CC*CRF | 0.03 | 1.03 |
| CHF*DM W/O CC | -0.02 | 0.98 |  | DM W CC*Ulcer | -0.01 | 0.99 |
| CHF*DM W CC | -0.09 | 0.91 |  | DM W CC*HT UC | -0.01 | 0.99 |
| CHF*CRF | -0.05 | 0.95 |  | DM W CC*HT C | 0.03 | 1.03 |
| CHF*Ulcer | -0.03 | 0.97 |  | DM W CC*Mild LD | -0.09 | 0.92 |
| CHF*HT UC | -0.17 | 0.85 |  | CRF*Ulcer | -0.02 | 0.98 |
| CHF*HT C | 0.03 | 1.03 |  | CRF*HT UC | -0.07 | 0.93 |
| CHF*Mild LD | -0.04 | 0.96 |  | CRF*HT C | 0.30 | 1.34 |
| CVD*COPD | 0.00 | 1.00 |  | CRF*Mild LD | -0.19 | 0.83 |
| CVD*Dementia | -0.06 | 0.94 |  | Ulcer*HT UC | 0.01 | 1.01 |
| CVD*DM W/O CC | 0.11 | 1.12 |  | Ulcer*HT C | -0.08 | 0.93 |
| CVD*DM W CC | -0.09 | 0.91 |  | Ulcer*Mild LD | -0.01 | 0.99 |
| CVD*CRF | -0.16 | 0.85 |  | HT UC*HT C | 0.00 | 1.00 |
| CVD*Ulcer | 0.06 | 1.06 |  | HT UC*Mild LD | -0.01 | 0.99 |
| CVD*HT UC | 0.05 | 1.06 |  | HT C*Mild LD | 0.03 | 1.03 |
| CVD*HT C | -0.08 | 0.92 |  |  |  |  |

## Table S6-2. Hazard ratios from Negative Deleted Model Main18&11.ND using Five-Cancer.

| Comorbid condition | Coef | HR |  | Comorbid condition | Coef | HR |
| --- | --- | --- | --- | --- | --- | --- |
| Age | 0.06 | 1.07 |  | CHF*HT UC | -0.17 | 0.85 |
| Sex | 0.49 | 1.63 |  | CVD*COPD | 0.01 | 1.01 |
| AMI | 0.27 | 1.3 |  | CVD*Dementia | -0.07 | 0.93 |
| Old MI | 0.08 | 1.08 |  | CVD* DM W/O CC | 0.12 | 1.13 |
| CHF | 0.75 | 2.11 |  | CVD* DM W CC | -0.08 | 0.92 |
| PVD | 0.24 | 1.27 |  | CVD*CRF | -0.16 | 0.85 |
| CVD | 0.37 | 1.45 |  | CVD*HT UC | 0.06 | 1.06 |
| COPD | 0.26 | 1.3 |  | COPD*Dementia | 0.05 | 1.05 |
| Dementia | 0.66 | 1.94 |  | COPD* DM W/O CC | -0.1 | 0.9 |
| Paralysis | 0.34 | 1.4 |  | COPD* DM W CC | -0.02 | 0.98 |
| DM W/O CC | 0.28 | 1.32 |  | COPD*CRF | -0.17 | 0.84 |
| DM W CC | 0.38 | 1.46 |  | COPD*HT UC | 0.01 | 1.01 |
| CRF | 0.8 | 2.23 |  | Dementia* DM W/O CC | -0.02 | 0.98 |
| M S LD | 0.81 | 2.24 |  | Dementia* DM W CC | -0.05 | 0.95 |
| RD | 0.29 | 1.33 |  | Dementia*CRF | -0.35 | 0.71 |
| AIDS | 1.65 | 5.22 |  | Dementia*HT UC | -0.04 | 0.96 |
| HT UC | 0.02 | 1.02 |  | DM W/O CC * DM W CC | -0.11 | 0.89 |
| CHF*CVD | -0.12 | 0.88 |  | DM W/O CC *CRF | 0.07 | 1.07 |
| CHF*COPD | -0.11 | 0.9 |  | DM W/O CC *HT UC | 0.05 | 1.05 |
| CHF*Dementia | -0.31 | 0.74 |  | DM W CC *CRF | 0.11 | 1.12 |
| CHF* DM W/O CC | 0.02 | 1.02 |  | DM W CC *HT UC | 0 | 1 |
| CHF* DM W CC | -0.08 | 0.93 |  | CRF*HT UC | -0.05 | 0.95 |
| CHF*CRF | 0.03 | 1.03 |  |  |  |  |

## Table S6-3. Hazard ratios from Variable Selected Model Main18&11.VS using Five-Cancer.

| Comorbid condition | Coef | HR |  | Comorbid condition | Coef | HR |
| --- | --- | --- | --- | --- | --- | --- |
| Diagnosis age | 0.06 | 1.07 |  | HT C | -0.07 | 0.93 |
| Sex | 0.49 | 1.64 |  | CHF*Dementia | -0.38 | 0.69 |
| AMI | 0.25 | 1.28 |  | CHF*HT UC | -0.20 | 0.82 |
| CHF | 0.68 | 1.98 |  | CVD*DM W/O CC | 0.10 | 1.10 |
| PVD | 0.23 | 1.26 |  | CVD*CRF | -0.19 | 0.82 |
| CVD | 0.43 | 1.54 |  | CVD*HT C | -0.09 | 0.91 |
| COPD | 0.29 | 1.34 |  | CVD*Mild LD | -0.13 | 0.87 |
| Dementia | 0.60 | 1.83 |  | COPD*DM W/O CC | -0.11 | 0.90 |
| Paralysis | 0.35 | 1.42 |  | COPD*CRF | -0.21 | 0.81 |
| DM W/O CC | 0.29 | 1.34 |  | COPD*Mild LD | -0.15 | 0.86 |
| DM W CC | 0.36 | 1.43 |  | Dementia*CRF | -0.37 | 0.69 |
| CRF | 0.76 | 2.13 |  | DM W/O CC*DM W CC | -0.14 | 0.87 |
| M S LD | 0.93 | 2.52 |  | DM W/O CC*Mild LD | 0.13 | 1.14 |
| RD | 0.30 | 1.35 |  | CRF*HT C | 0.30 | 1.35 |
| AIDS | 1.66 | 5.26 |  | CRF*Mild LD | -0.20 | 0.82 |
| HT UC | 0.04 | 1.04 |  |  |  |  |

## Table S7-1. The AUCs using OM, NDM, and VSM and the differences based on the **Breast.F.65** validation set

| Training set | Covariates | OM | NDM | VSM | Difference OM-NDM | Difference NDM-VSM |
| --- | --- | --- | --- | --- | --- | --- |
| Five-Cancer | Main18 | 0.7593 | 0.7598 | 0.7133 | -0.0004 | 0.0465 |
| Five-Cancer.65 | Main18 | 0.7595 | 0.7601 | 0.7126 | -0.0007 | 0.0476 |
| Five-Cancer.F | Main18 | 0.7585 | 0.7584 | 0.7133 | 0.0000 | 0.0451 |
| Five-Cancer.F.65 | Main18 | 0.7592 | 0.7592 | 0.7126 | 0.0000 | 0.0466 |
| Breast.F | Main17 | 0.7608 | 0.7617 | 0.7135 | -0.0009 | 0.0482 |
| Breast.F.65 | Main17 | 0.7623 | 0.7628 | 0.7237 | -0.0005 | 0.0390 |
| Five-Cancer | Main18&6 | 0.7590 | 0.7592 | 0.7137 | -0.0002 | 0.0455 |
| Five-Cancer.65 | Main18&6 | 0.7586 | 0.7593 | 0.7183 | -0.0007 | 0.0410 |
| Five-Cancer.F | Main18&6 | 0.7565 | 0.7574 | 0.7158 | -0.0009 | 0.0416 |
| Five-Cancer.F.65 | Main18&6 | 0.7563 | 0.7579 | 0.7126 | -0.0015 | 0.0453 |
| Breast.F | Main17&6 | 0.7578 | 0.7605 | 0.7154 | -0.0027 | 0.0451 |
| Breast.F.65 | Main17&6 | 0.7579 | 0.7616 | 0.7248 | -0.0037 | 0.0368 |
| Five-Cancer | Main18&11 | **0.7594** | **0.7609** | **0.7261** | -0.0015 | 0.0348 |
| Five-Cancer.65 | Main18&11 | 0.7579 | 0.7609 | 0.7243 | -0.0030 | 0.0366 |
| Five-Cancer.F | Main18&11 | 0.7563 | 0.7568 | 0.7261 | -0.0005 | 0.0307 |
| Five-Cancer.F.65 | Main18&11 | 0.7550 | 0.7588 | 0.7241 | -0.0038 | 0.0347 |
| Breast.F | Main17&11 | 0.7490 | 0.7514 | 0.7273 | -0.0024 | 0.0241 |
| Breast.F.65 | Main17&11 | 0.7445 | 0.7480 | 0.7197 | -0.0035 | 0.0283 |
| Difference |  | 0.0178 | 0.0148 | 0.0147 |  |  |
|  |  |  |  | Average | -0.0015 | 0.0399 |
|  |  |  |  | SD | 0.0013 | 0.0072 |

## Table S7-2. The AUCs using OM, NDM, and VSM and the differences based on the **CRC.M.65** validation set

| Training set | Covariates | OM | NDM | VSM | Difference OM-NDM | Difference NDM-VSM |
| --- | --- | --- | --- | --- | --- | --- |
| Five-Cancer | Main18 | 0.7122 | 0.7125 | 0.6794 | -0.0003 | 0.0330 |
| Five-Cancer.65 | Main18 | 0.7118 | 0.7123 | 0.6786 | -0.0004 | 0.0336 |
| Five-Cancer.M | Main18 | 0.7110 | 0.7114 | 0.6791 | -0.0004 | 0.0323 |
| Five-Cancer.M.65 | Main18 | 0.7106 | 0.7115 | 0.6788 | -0.0009 | 0.0327 |
| CRC.M | Main17 | 0.7126 | 0.7126 | 0.6786 | 0.0000 | 0.0340 |
| CRC.M.65 | Main17 | 0.7121 | 0.7120 | 0.6852 | 0.0000 | 0.0268 |
| Five-Cancer | Main18&6 | 0.7117 | 0.7116 | 0.6816 | 0.0001 | 0.0300 |
| Five-Cancer.65 | Main18&6 | 0.7111 | 0.7112 | 0.6837 | -0.0001 | 0.0274 |
| Five-Cancer.M | Main18&6 | 0.7100 | 0.7119 | 0.6799 | -0.0019 | 0.0320 |
| Five-Cancer.M.65 | Main18&6 | 0.7093 | 0.7112 | 0.6790 | -0.0019 | 0.0322 |
| CRC.M | Main17&6 | 0.7111 | 0.7113 | 0.6792 | -0.0002 | 0.0321 |
| CRC.M.65 | Main17&6 | 0.7105 | 0.7108 | 0.6849 | -0.0003 | 0.0259 |
| Five-Cancer | Main18&11 | **0.7128** | **0.7139** | **0.6907** | -0.0011 | 0.0232 |
| Five-Cancer.65 | Main18&11 | 0.7123 | 0.7136 | 0.6864 | -0.0013 | 0.0272 |
| Five-Cancer.M | Main18&11 | 0.7097 | 0.7123 | 0.6813 | -0.0026 | 0.0310 |
| Five-Cancer.M.65 | Main18&11 | 0.7093 | 0.7112 | 0.6805 | -0.0020 | 0.0307 |
| CRC.M | Main17&11 | 0.7098 | 0.7108 | 0.6851 | -0.0010 | 0.0258 |
| CRC.M.65 | Main17&11 | 0.7093 | 0.7111 | 0.6844 | -0.0018 | 0.0267 |
| Difference |  | 0.0035 | 0.0031 | 0.0121 |  |  |
|  |  |  |  | Average | -0.0009 | 0.0298 |
|  |  |  |  | SD | 0.0009 | 0.0033 |

## Table S7-3. The AUCs using OM, NDM, and VSM the differences based on the **CRC.F.65** validation set

| Training set | Covariates | OM | NDM | VSM | Difference OM-NDM | Difference NDM-VSM |
| --- | --- | --- | --- | --- | --- | --- |
| Five-Cancer | Main18 | 0.7433 | 0.7436 | 0.7125 | -0.0003 | 0.0311 |
| Five-Cancer.65 | Main18 | 0.7431 | 0.7436 | 0.7096 | -0.0005 | 0.0340 |
| Five-Cancer.F | Main18 | 0.7441 | 0.7441 | 0.7110 | 0.0000 | 0.0331 |
| Five-Cancer.F.65 | Main18 | 0.7442 | 0.7443 | 0.7103 | 0.0000 | 0.0340 |
| CRC.F | Main17 | 0.7429 | 0.7428 | 0.7090 | 0.0001 | 0.0338 |
| CRC.F.65 | Main17 | 0.7430 | 0.7429 | 0.7102 | 0.0001 | 0.0327 |
| Five-Cancer | Main18&6 | 0.7430 | 0.7434 | 0.7096 | -0.0004 | 0.0338 |
| Five-Cancer.65 | Main18&6 | 0.7426 | 0.7432 | 0.7110 | -0.0006 | 0.0322 |
| Five-Cancer.F | Main18&6 | 0.7430 | 0.7433 | 0.7133 | -0.0003 | 0.0300 |
| Five-Cancer.F.65 | Main18&6 | 0.7429 | 0.7434 | 0.7088 | -0.0005 | 0.0346 |
| CRC.F | Main17&6 | 0.7412 | 0.7416 | 0.7121 | -0.0003 | 0.0295 |
| CRC.F.65 | Main17&6 | 0.7408 | 0.7417 | 0.7082 | -0.0009 | 0.0335 |
| Five-Cancer | Main18&11 | **0.7450** | **0.7460** | **0.7228** | -0.0010 | 0.0232 |
| Five-Cancer.65 | Main18&11 | 0.7445 | 0.7458 | 0.7195 | -0.0014 | 0.0263 |
| Five-Cancer.F | Main18&11 | 0.7436 | 0.7443 | 0.7198 | -0.0006 | 0.0244 |
| Five-Cancer.F.65 | Main18&11 | 0.7428 | 0.7453 | 0.7185 | -0.0025 | 0.0268 |
| CRC.F | Main17&11 | 0.7412 | 0.7423 | 0.7115 | -0.0011 | 0.0308 |
| CRC.F.65 | Main17&11 | 0.7403 | 0.7420 | 0.7094 | -0.0017 | 0.0326 |
| Difference |  | 0.0047 | 0.0044 | 0.0146 |  |  |
|  |  |  |  | Average | -0.0007 | 0.0309 |
|  |  |  |  | SD | 0.0007 | 0.0035 |

## Table S7-4. The AUCs using OM, NDM, and VSM and the differences based on the **Liver.M.65** validation set

| Training set | Covariates | OM | NDM | VSM | Difference OM-NDM | Difference NDM-VSM |
| --- | --- | --- | --- | --- | --- | --- |
| Five-Cancer | Main18 | 0.6636 | 0.6601 | 0.6415 | 0.0035 | 0.0186 |
| Five-Cancer.65 | Main18 | 0.6654 | 0.6608 | 0.6376 | 0.0046 | 0.0232 |
| Five-Cancer.M | Main18 | 0.6662 | 0.6611 | 0.6405 | 0.0052 | 0.0206 |
| Five-Cancer.M.65 | Main18 | 0.6677 | 0.6615 | 0.6380 | 0.0062 | 0.0235 |
| Liver.M | Main17 | 0.6697 | 0.6604 | 0.6398 | 0.0093 | 0.0206 |
| Liver.M.65 | Main17 | 0.6709 | 0.6617 | 0.6418 | 0.0092 | 0.0199 |
| Five-Cancer | Main18&6 | 0.6621 | 0.6602 | 0.6346 | 0.0019 | 0.0256 |
| Five-Cancer.65 | Main18&6 | 0.6639 | 0.6607 | 0.6360 | 0.0032 | 0.0247 |
| Five-Cancer.M | Main18&6 | 0.6651 | 0.6609 | 0.6401 | 0.0042 | 0.0208 |
| Five-Cancer.M.65 | Main18&6 | 0.6670 | 0.6606 | 0.6383 | 0.0064 | 0.0223 |
| Liver.M | Main17&6 | 0.6687 | 0.6581 | 0.6357 | 0.0106 | 0.0224 |
| Liver.M.65 | Main17&6 | 0.6690 | 0.6597 | 0.6416 | 0.0093 | 0.0181 |
| Five-Cancer | Main18&11 | **0.6652** | **0.6634** | **0.6364** | 0.0017 | 0.0270 |
| Five-Cancer.65 | Main18&11 | 0.6660 | 0.6639 | 0.6357 | 0.0020 | 0.0282 |
| Five-Cancer.M | Main18&11 | 0.6684 | 0.6633 | 0.6394 | 0.0051 | 0.0239 |
| Five-Cancer.M.65 | Main18&11 | 0.6691 | 0.6631 | 0.6324 | 0.0060 | 0.0307 |
| Liver.M | Main17&11 | 0.6676 | 0.6577 | 0.6359 | 0.0099 | 0.0218 |
| Liver.M.65 | Main17&11 | 0.6664 | 0.6576 | 0.6376 | 0.0089 | 0.0199 |
| Difference |  | 0.0088 | 0.0063 | 0.0094 |  |  |
|  |  |  |  | Average | 0.0060 | 0.0229 |
|  |  |  |  | SD | 0.0029 | 0.0034 |

## Table S7-5. The AUCs using OM, NDM, and VSM and the differences based on the **Liver.F.65** validation set

| Training set | Covariates | OM | NDM | VSM | Difference OM-NDM | Difference NDM-VSM |
| --- | --- | --- | --- | --- | --- | --- |
| Five-Cancer | Main18 | 0.6926 | 0.6901 | 0.6688 | 0.0025 | 0.0212 |
| Five-Cancer.65 | Main18 | 0.6918 | 0.6886 | 0.6698 | 0.0032 | 0.0188 |
| Five-Cancer.F | Main18 | 0.6933 | 0.6925 | 0.6700 | 0.0008 | 0.0225 |
| Five-Cancer.F.65 | Main18 | 0.6937 | 0.6927 | 0.6697 | 0.0010 | 0.0230 |
| Liver.F | Main17 | 0.7001 | 0.6922 | 0.6715 | 0.0079 | 0.0208 |
| Liver.F.65 | Main17 | 0.7005 | 0.6927 | 0.6704 | 0.0077 | 0.0223 |
| Five-Cancer | Main18&6 | 0.6923 | 0.6901 | 0.6677 | 0.0022 | 0.0224 |
| Five-Cancer.65 | Main18&6 | 0.6926 | 0.6887 | 0.6685 | 0.0039 | 0.0202 |
| Five-Cancer.F | Main18&6 | 0.6918 | 0.6920 | 0.6698 | -0.0002 | 0.0222 |
| Five-Cancer.F.65 | Main18&6 | 0.6933 | 0.6925 | 0.6684 | 0.0008 | 0.0241 |
| Liver.F | Main17&6 | 0.6994 | 0.6734 | 0.6714 | 0.0260 | 0.0020 |
| Liver.F.65 | Main17&6 | 0.6997 | 0.6735 | 0.6717 | 0.0262 | 0.0018 |
| Five-Cancer | Main18&11 | **0.6985** | **0.6946** | **0.6727** | 0.0039 | 0.0219 |
| Five-Cancer.65 | Main18&11 | 0.6974 | 0.6928 | 0.6717 | 0.0046 | 0.0211 |
| Five-Cancer.F | Main18&11 | 0.6950 | 0.6957 | 0.6735 | -0.0007 | 0.0222 |
| Five-Cancer.F.65 | Main18&11 | 0.6954 | 0.6981 | 0.6722 | -0.0027 | 0.0259 |
| Liver.F | Main17&11 | 0.7003 | 0.6767 | 0.6744 | 0.0236 | 0.0023 |
| Liver.F.65 | Main17&11 | 0.6995 | 0.6739 | 0.6744 | 0.0255 | -0.0005 |
| Difference |  | 0.0087 | 0.0247 | 0.0067 |  |  |
|  |  |  |  | Average | 0.0076 | 0.0175 |
|  |  |  |  | SD | 0.0101 | 0.0090 |

## Table S7-6. The AUCs using OM, NDM, and VSM and the differences based on the **Lung.M.65** validation set

| Training set | Covariates | OM | NDM | VSM | Difference OM-NDM | Difference NDM-VSM |
| --- | --- | --- | --- | --- | --- | --- |
| Five-Cancer | Main18 | 0.6527 | 0.6527 | 0.6351 | 0.0000 | 0.0176 |
| Five-Cancer.65 | Main18 | 0.6525 | 0.6525 | 0.6339 | 0.0000 | 0.0186 |
| Five-Cancer.M | Main18 | 0.6532 | 0.6533 | 0.6353 | -0.0001 | 0.0180 |
| Five-Cancer.M.65 | Main18 | 0.6528 | 0.6532 | 0.6337 | -0.0005 | 0.0195 |
| Lung.M | Main17 | 0.6519 | 0.6520 | 0.6346 | -0.0001 | 0.0174 |
| Lung.M.65 | Main17 | 0.6508 | 0.6510 | 0.6337 | -0.0002 | 0.0173 |
| Five-Cancer | Main18&6 | 0.6524 | 0.6525 | 0.6343 | -0.0001 | 0.0182 |
| Five-Cancer.65 | Main18&6 | 0.6521 | 0.6524 | 0.6366 | -0.0003 | 0.0158 |
| Five-Cancer.M | Main18&6 | 0.6531 | 0.6532 | 0.6356 | -0.0001 | 0.0176 |
| Five-Cancer.M.65 | Main18&6 | 0.6525 | 0.6532 | 0.6339 | -0.0007 | 0.0193 |
| Lung.M | Main17&6 | 0.6510 | 0.6510 | 0.6346 | 0.0001 | 0.0164 |
| Lung.M.65 | Main17&6 | 0.6499 | 0.6523 | 0.6396 | -0.0024 | 0.0127 |
| Five-Cancer | Main18&11 | **0.6547** | **0.6561** | **0.6390** | -0.0014 | 0.0171 |
| Five-Cancer.65 | Main18&11 | 0.6540 | 0.6540 | 0.6389 | 0.0000 | 0.0151 |
| Five-Cancer.M | Main18&11 | 0.6551 | 0.6539 | 0.6359 | 0.0012 | 0.0180 |
| Five-Cancer.M.65 | Main18&11 | 0.6542 | 0.6561 | 0.6340 | -0.0019 | 0.0220 |
| Lung.M | Main17&11 | 0.6477 | 0.6495 | 0.6340 | -0.0018 | 0.0155 |
| Lung.M.65 | Main17&11 | 0.6461 | 0.6479 | 0.6410 | -0.0018 | 0.0069 |
| Difference |  | 0.0090 | 0.0082 | 0.0073 |  |  |
|  |  |  |  | Average | -0.0006 | 0.0168 |
|  |  |  |  | SD | 0.0009 | 0.0032 |

## Table S7-7. The AUCs using OM, NDM, and VSM and the differences based on the **Lung.F.65** validation set

| Training set | Covariates | OM | NDM | VSM | Difference OM-NDM | Difference NDM-VSM |
| --- | --- | --- | --- | --- | --- | --- |
| Five-Cancer | Main18 | 0.6914 | 0.6914 | 0.6782 | 0.0001 | 0.0132 |
| Five-Cancer.65 | Main18 | 0.6914 | 0.6914 | 0.6784 | 0.0000 | 0.0131 |
| Five-Cancer.F | Main18 | 0.6918 | 0.6918 | 0.6784 | 0.0000 | 0.0134 |
| Five-Cancer.F.65 | Main18 | 0.6919 | 0.6919 | 0.6784 | 0.0000 | 0.0135 |
| Lung.F | Main17 | 0.6916 | 0.6917 | 0.6829 | 0.0000 | 0.0088 |
| Lung.F.65 | Main17 | 0.6925 | 0.6919 | 0.6850 | 0.0006 | 0.0069 |
| Five-Cancer | Main18&6 | 0.6915 | 0.6915 | 0.6786 | 0.0000 | 0.0128 |
| Five-Cancer.65 | Main18&6 | 0.6913 | 0.6914 | 0.6789 | -0.0001 | 0.0125 |
| Five-Cancer.F | Main18&6 | 0.6920 | 0.6921 | 0.6796 | -0.0001 | 0.0125 |
| Five-Cancer.F.65 | Main18&6 | 0.6920 | 0.6918 | 0.6783 | 0.0002 | 0.0136 |
| Lung.F | Main17&6 | 0.6895 | 0.6915 | 0.6829 | -0.0020 | 0.0086 |
| Lung.F.65 | Main17&6 | 0.6907 | 0.6894 | 0.6846 | 0.0013 | 0.0048 |
| Five-Cancer | Main18&11 | **0.6905** | **0.6903** | **0.6826** | 0.0002 | 0.0077 |
| Five-Cancer.65 | Main18&11 | 0.6901 | 0.6904 | 0.6812 | -0.0003 | 0.0091 |
| Five-Cancer.F | Main18&11 | 0.6903 | 0.6908 | 0.6820 | -0.0005 | 0.0088 |
| Five-Cancer.F.65 | Main18&11 | 0.6900 | 0.6908 | 0.6809 | -0.0008 | 0.0099 |
| Lung.F | Main17&11 | 0.6851 | 0.6886 | 0.6783 | -0.0035 | 0.0103 |
| Lung.F.65 | Main17&11 | 0.6864 | 0.6880 | 0.6800 | -0.0016 | 0.0080 |
| Difference |  | 0.0074 | 0.0041 | 0.0068 |  |  |
|  |  |  |  | Average | -0.0004 | 0.0104 |
|  |  |  |  | SD | 0.0011 | 0.0027 |

## Table S7-8. The AUCs using OM, NDM, and VSM and the differences based on the **Oral.M.65** validation set

| Training set | Covariates | OM | NDM | VSM | Difference OM-NDM | Difference NDM-VSM |
| --- | --- | --- | --- | --- | --- | --- |
| Five-Cancer | Main18 | 0.6607 | 0.6614 | 0.6306 | -0.0007 | 0.0307 |
| Five-Cancer.65 | Main18 | 0.6585 | 0.6594 | 0.6296 | -0.0009 | 0.0299 |
| Five-Cancer.M | Main18 | 0.6578 | 0.6587 | 0.6306 | -0.0009 | 0.0281 |
| Five-Cancer.M.65 | Main18 | 0.6548 | 0.6568 | 0.6260 | -0.0020 | 0.0308 |
| Oral.M | Main17 | 0.6638 | 0.6641 | 0.6262 | -0.0003 | 0.0379 |
| Oral.M.65 | Main17 | 0.6582 | 0.6636 | 0.6343 | -0.0053 | 0.0293 |
| Five-Cancer | Main18&6 | 0.6602 | 0.6611 | 0.6266 | -0.0009 | 0.0344 |
| Five-Cancer.65 | Main18&6 | 0.6576 | 0.6593 | 0.6339 | -0.0017 | 0.0255 |
| Five-Cancer.M | Main18&6 | 0.6573 | 0.6582 | 0.6329 | -0.0008 | 0.0253 |
| Five-Cancer.M.65 | Main18&6 | 0.6545 | 0.6569 | 0.6264 | -0.0025 | 0.0306 |
| Oral.M | Main17&6 | 0.6622 | 0.6639 | 0.6282 | -0.0017 | 0.0357 |
| Oral.M.65 | Main17&6 | 0.6521 | 0.6621 | 0.6445 | -0.0100 | 0.0176 |
| Five-Cancer | Main18&11 | **0.6626** | **0.6627** | **0.6387** | -0.0001 | 0.0240 |
| Five-Cancer.65 | Main18&11 | 0.6590 | 0.6593 | 0.6346 | -0.0003 | 0.0247 |
| Five-Cancer.M | Main18&11 | 0.6588 | 0.6595 | 0.6282 | -0.0007 | 0.0313 |
| Five-Cancer.M.65 | Main18&11 | 0.6548 | 0.6563 | 0.6270 | -0.0016 | 0.0294 |
| Oral.M | Main17&11 | 0.6531 | 0.6490 | 0.6293 | 0.0041 | 0.0197 |
| Oral.M.65 | Main17&11 | 0.6400 | 0.6531 | 0.6498 | -0.0130 | 0.0033 |
| Difference |  | 0.0238 | 0.0151 | 0.0238 |  |  |
|  |  |  |  | Average | -0.0022 | 0.0271 |
|  |  |  |  | SD | 0.0039 | 0.0079 |

## Table S7-9. The AUCs using OM, NDM, and VSM and the differences based on the **Oral.F.65** validation set

| Training set | Covariates | OM | NDM | VSM | Difference OM-NDM | Difference NDM-VSM |
| --- | --- | --- | --- | --- | --- | --- |
| Five-Cancer | Main18 | 0.6952 | 0.6975 | 0.6846 | -0.0022 | 0.0128 |
| Five-Cancer.65 | Main18 | 0.6949 | 0.6978 | 0.6848 | -0.0029 | 0.0131 |
| Five-Cancer.F | Main18 | 0.6962 | 0.6971 | 0.6870 | -0.0009 | 0.0102 |
| Five-Cancer.F.65 | Main18 | 0.6957 | 0.6965 | 0.6853 | -0.0008 | 0.0112 |
| Oral.F | Main17 | 0.6892 | 0.6957 | 0.6925 | -0.0065 | 0.0031 |
| Oral.F.65 | Main17 | 0.6893 | 0.6924 | 0.6915 | -0.0030 | 0.0009 |
| Five-Cancer | Main18&6 | 0.6957 | 0.6976 | 0.6851 | -0.0020 | 0.0125 |
| Five-Cancer.65 | Main18&6 | 0.6954 | 0.6981 | 0.6910 | -0.0027 | 0.0071 |
| Five-Cancer.F | Main18&6 | 0.6952 | 0.6968 | 0.6903 | -0.0016 | 0.0064 |
| Five-Cancer.F.65 | Main18&6 | 0.6953 | 0.6974 | 0.6849 | -0.0021 | 0.0124 |
| Oral.F | Main17&6 | 0.6866 | 0.6882 | 0.6923 | -0.0015 | -0.0041 |
| Oral.F.65 | Main17&6 | 0.6887 | 0.6913 | 0.6879 | -0.0026 | 0.0033 |
| Five-Cancer | Main18&11 | **0.6961** | **0.6970** | **0.6857** | -0.0009 | 0.0113 |
| Five-Cancer.65 | Main18&11 | 0.6966 | 0.7003 | 0.6837 | -0.0037 | 0.0166 |
| Five-Cancer.F | Main18&11 | 0.6936 | 0.6953 | 0.6893 | -0.0017 | 0.0060 |
| Five-Cancer.F.65 | Main18&11 | 0.6939 | 0.6938 | 0.6844 | 0.0001 | 0.0094 |
| Oral.F | Main17&11 | 0.6846 | 0.6924 | 0.6939 | -0.0078 | -0.0015 |
| Oral.F.65 | Main17&11 | 0.6843 | 0.6842 | 0.7132 | 0.0002 | -0.0291 |
| Difference |  | 0.0123 | 0.0161 | 0.0295 |  |  |
|  |  |  |  | Average | -0.0024 | 0.0057 |
|  |  |  |  | SD | 0.0020 | 0.0103 |

## Table S7-10. Comparison of the AUCs based on the validation and test sets for the model Main18&11.ND.

| Five-Cancer | Main18&11 | NDM | Validation set | Test set |
| --- | --- | --- | --- | --- |
|  |  | Breast.F.65 | 0.7609 | 0.7316 |
|  |  | CRC.M.65 | 0.7139 | 0.7129 |
|  |  | CRC.F.65 | 0.7460 | 0.7512 |
|  |  | Liver.M.65 | 0.6634 | 0.6818 |
|  |  | Liver.F.65 | 0.6946 | 0.6907 |
|  |  | Lung.M.65 | 0.6561 | 0.6440 |
|  |  | Lung.F.65 | 0.6903 | 0.7156 |
|  |  | Oral.M.65 | 0.6627 | 0.6511 |
|  |  | Oral.F.65 | 0.6970 | 0.7133 |

## Table S8. Algorithm for mapping stage at diagnosis from TNM to SEER Summary Stage (localized, regional, and distant):

|  | 6^th^ edition | 7^th^ edition |
| --- | --- | --- |
| Cancer /SEER stages |  |  |
| **Breast** |  |  |
| Localized | T1, T2, T3 | T1mi-1c, T2, T3 |
| Regional | N1-3, T4 | N1-3b, T4 |
| Distant | N3c, M1 | N3c, M1, MC |
| Non-Distant* | M0, MB | M0, MB |
| **Colorectal** |  |  |
| Localized | T1, T2, T3 | T1, T2, T3 |
| Regional | N1, N2, T4 | N1, N2, T4 |
| Distant | M1 | M1, MC |
| Non-Distant* | M0, MB | M0, MB |
| **Lung**, |  |  |
| Localized | T1, T2 | T1, T2 |
| Regional | N1, N2, T3, T4 | N1, N2, T3, T4 |
| Distant | M1 | N3, M1, MC |
| Non-Distant* | M0, MB | M0, MB |
| **Oral-** |  |  |
| Localized | T1, T2 | T1, T2 |
| Regional | N1-3, T3, T4 | N1-3, T3, T4 |
| Distant | M1 | M1, MC |
| Non-Distant* | M0, MB | M0, MB |
| **Liver-**Liver cell Carcinoma |  |  |
| Localized | T1, T2, T3 | T1, T2, T3(a)**_** |
| Regional | N1, T4 | N1, T3b, T4 |
| Distant | M1 | M1, MC |
| Non-Distant* | M0, MB | M0, MB |
| **Liver**-Intrahepatic bile duct carcinoma |  |  |
| Localized | T1, T2, T3 | T1, T2 |
| Regional | N1, T4 | N1, T3, T4 |
| Distant | M1 | M1, MC |
| Non-Distant* | M0, MB | M0, MB |

The cancer stage information for cancer cases was obtained from The Taiwan Cancer Registry Long Form Database, which are based on the TNM stage information derived from the American Joint Committee on Cancer (AJCC) and the International Union Against Cancer (UICC). AJCC 6^th^ edition was used from 2004 to 2009, 7^th^ was used since 2010. The algorithms for mapping stage at diagnosis from TNM to SEER Summary Stage in Table S8 follows that of Japan National Cancer Control and Information Service (<https://ganjoho.jp/data/reg_stat/cancer_reg/hospital/info/toroku08.pdf>) and clinical advice.

* For patients whose cancer stage cannot be classified into localized, regional and distant stages, we categorize their cancer stage as non-distant stage if the metastatic information can be obtained from TCR-LF.

## Table S9-1. Number of patients by age, stage, and comorbidity level: **Breast Cancer**

| Breast cancer | | | | | | | | | | | | | |
| --- | --- | --- | --- | --- | --- | --- | --- | --- | --- | --- | --- | --- | --- |
| Age |  | 15-64 | | | 65-74 | | | 75-84 | | | 85-94 | | |
| Comorbidity level* | | 0 | 1 | 2 | 0 | 1 | 2 | 0 | 1 | 2 | 0 | 1 | 2 |
| Stage** | NA^+^ | 6113 | 1790 | 543 | 459 | 680 | 376 | 144 | 333 | 237 | 40 | 78 | 86 |
|  | Localized | 27105 | 8104 | 2092 | 1592 | 2688 | 1043 | 356 | 1034 | 696 | 58 | 161 | 172 |
|  | Regional | 18393 | 5180 | 1353 | 1059 | 1704 | 722 | 244 | 665 | 435 | 40 | 116 | 105 |
|  | Distant | 2846 | 542 | 147 | 268 | 268 | 119 | 84 | 136 | 104 | 14 | 45 | 20 |
|  |  | X-squared = 103.86,  p-value < 2.2e-16 | | | X-squared = 36.705, p-value = 2.072e-07 | | | X-squared = 15.865, p-value = 0.003206 | | | X-squared = 9.7462, p-value = 0.04493 | | |
|  | Non-distant | 423 | 101 | 29 | 35 | 42 | 15 | <5 | 29 | 21 | <5 | <5 | <5 |
|  | Unknown^++^ | 680 | 122 | 46 | 46 | 54 | 23 | 10 | 28 | 20 | 9 | <5 | 5 |

*comorbidity level: 0 refers to individuals with no comorbid conditions. 2 refers to conditions that include comorbid conditions 3, 6, 7, 11, 13, 16 or the comorbid index exceeded 0.66. 1 refers to individuals not classified into 0 nor 2 of comorbidity level.

** For patients whose cancer stage cannot be classified into localized, regional and distant stages, we categorize their cancer stage as non-distant stage if the metastatic information can be obtained from TCR-LF. Patients whose cancer stage cannot be classified into localized, regional, distant and non-distant stage is categorized as unknown stage.

^+^ Cancer patients in TCR but not in TCRLF.

^++^ Cancer patients in TCRLF stage information missing.

## Table S9-2. Number of patients by age, stage, and comorbidity level: **Colorectal Cancer**

| Colorectal cancer | | | | | | | | | | | | | | | | | | | | | | | | | |
| --- | --- | --- | --- | --- | --- | --- | --- | --- | --- | --- | --- | --- | --- | --- | --- | --- | --- | --- | --- | --- | --- | --- | --- | --- | --- |
| Age | | 15-64 | | | | | | 65-74 | | | | | | 75-84 | | | | | | 85-94 | | | | | |
| Sex | | Male | | | Female | | | Male | | | Female | | | Male | | | Female | | | Male | | | Female | | |
| Comorbidity level* | | 0 | 1 | 2 | 0 | 1 | 2 | 0 | 1 | 2 | 0 | 1 | 2 | 0 | 1 | 2 | 0 | 1 | 2 | 0 | 1 | 2 | 0 | 1 | 2 |
| Stage** | NA^+^ | 2134 | 1097 | 426 | 1568 | 674 | 274 | 908 | 1067 | 834 | 560 | 805 | 471 | 712 | 1071 | 1242 | 404 | 853 | 800 | 222 | 265 | 451 | 203 | 309 | 417 |
|  | Localized | 5896 | 3492 | 1117 | 4673 | 2184 | 674 | 1975 | 2657 | 1629 | 1257 | 2021 | 998 | 1181 | 2058 | 2025 | 698 | 1732 | 1172 | 254 | 446 | 628 | 222 | 458 | 500 |
|  | Regional | 5689 | 2713 | 817 | 4818 | 1816 | 499 | 1777 | 2036 | 1149 | 1190 | 1651 | 709 | 1073 | 1671 | 1489 | 746 | 1537 | 1069 | 236 | 384 | 494 | 199 | 466 | 394 |
|  | Distant | 3398 | 1382 | 439 | 2971 | 921 | 290 | 1023 | 1095 | 626 | 612 | 803 | 392 | 625 | 1032 | 925 | 448 | 854 | 600 | 152 | 222 | 322 | 160 | 281 | 260 |
|  |  | X-squared = 135.1, p-value <  2.2e-16 | | | X-squared =  111.15,  p-value <  2.2e-16 | | | X-squared =  40.276, p-value =  3.795e-08 | | | X-squared =  25.809,  p-value =  3.457e-05 | | | X-squared =  16.571, p-value =  0.002341 | | | X-squared =  15.891,  p-value =  0.003169 | | | X-squared =  4.0896,  p-value =  0.394 | | | X-squared =  13.113, p-value =  0.01074 | | |
|  | Non-distant | 457 | 284 | 125 | 353 | 164 | 54 | 118 | 193 | 124 | 68 | 114 | 71 | 61 | 90 | 161 | 24 | 101 | 118 | 26 | 49 | 85 | 28 | 63 | 91 |
|  | Unknown^++^ | 572 | 249 | 98 | 443 | 168 | 55 | 169 | 181 | 137 | 103 | 137 | 78 | 118 | 145 | 226 | 67 | 148 | 109 | 34 | 56 | 77 | 43 | 62 | 89 |

*comorbidity level: 0 refers to individuals with no comorbid conditions. 2 refers to conditions that include comorbid conditions 3, 6, 7, 11, 13, 16 or the comorbid score exceeded 0.66. 1 refers to individuals not classified into 0 nor 2 of comorbidity level.

** For patients whose cancer stage cannot be classified into localized, regional and distant stages, we categorize their cancer stage as non-distant stage if the metastatic information can be obtained from TCR-LF. Patients whose cancer stage cannot be classified into localized, regional, distant and non-distant stage is categorized as unknown stage.

^+^ Cancer patients in TCR but not in TCRLF.

^++^ Cancer patients in TCRLF with stage information missing.

## Table S9-3. Number of patients by age, stage, and comorbidity level: **Liver Cancer**

| Liver cancer | | | | | | | | | | | | | | | | | | | | | | | | | |
| --- | --- | --- | --- | --- | --- | --- | --- | --- | --- | --- | --- | --- | --- | --- | --- | --- | --- | --- | --- | --- | --- | --- | --- | --- | --- |
| Age | | 15-64 | | | | | | 65-74 | | | | | | 75-84 | | | | | | 85-94 | | | | | |
| Sex | | Male | | | Female | | | Male | | | Female | | | Male | | | Female | | | Male | | | Female | | |
| Comorbidity level* | | 0 | 1 | 2 | 0 | 1 | 2 | 0 | 1 | 2 | 0 | 1 | 2 | 0 | 1 | 2 | 0 | 1 | 2 | 0 | 1 | 2 | 0 | 1 | 2 |
| Stage** | NA^+^ | 1627 | 1289 | 928 | 306 | 354 | 266 | 536 | 800 | 799 | 191 | 542 | 493 | 337 | 720 | 953 | 183 | 530 | 618 | 125 | 173 | 295 | 83 | 194 | 266 |
|  | Localized | 9176 | 11912 | 5352 | 1673 | 3560 | 1598 | 2180 | 5852 | 3942 | 764 | 4032 | 2395 | 984 | 3095 | 3107 | 404 | 2581 | 2060 | 167 | 465 | 628 | 108 | 381 | 370 |
|  | Regional | 3940 | 2209 | 833 | 641 | 461 | 163 | 801 | 1110 | 646 | 241 | 591 | 343 | 356 | 645 | 608 | 153 | 532 | 385 | 55 | 137 | 158 | 50 | 145 | 132 |
|  | Distant | 3320 | 1293 | 478 | 636 | 352 | 147 | 674 | 750 | 509 | 260 | 461 | 244 | 269 | 472 | 488 | 156 | 394 | 277 | 63 | 107 | 115 | 50 | 90 | 93 |
|  |  | X-squared =  2303.7, p-value <  2.2e-16 | | | X-squared =  686.46,  p-value <  2.2e-16 | | | X-squared =  420.4, p-value <  2.2e-16 | | | X-squared =  250.5,  p-value <  2.2e-16 | | | X-squared =  106.06, p-value <  2.2e-16 | | | X-squared =  116.53, p-value <  2.2e-16 | | | X-squared =  17.53, p-value =  0.001524 | | | X-squared =  12.144, p-value =  0.01631 | | |
|  | Non-distant | 113 | 83 | 39 | 27 | 17 | 11 | 25 | 60 | 41 | 15 | 42 | 21 | 16 | 36 | 54 | 11 | 44 | 57 | 7 | 23 | 40 | 6 | 29 | 32 |
|  | Unknown^++^ | 881 | 661 | 371 | 230 | 223 | 116 | 248 | 395 | 248 | 98 | 264 | 156 | 115 | 245 | 246 | 60 | 213 | 162 | 28 | 44 | 66 | 29 | 52 | 60 |

*comorbidity level: 0 refers to individuals with no comorbid conditions. 2 refers to conditions that include comorbid conditions 3, 6, 7, 11, 13, 16 or the comorbid score exceeded 0.66. 1 refers to individuals not classified into 0 nor 2 of comorbidity level.

** For patients whose cancer stage cannot be classified into localized, regional and distant stages, we categorize their cancer stage as non-distant stage if the metastatic information can be obtained from TCR-LF. Patients whose cancer stage cannot be classified into localized, regional, distant and non-distant stage is categorized as unknown stage.

^+^ Cancer patient in TCR but not in TCRLF.

^++^ Cancer patient in TCRLF stage information missing.

## Table S9-4. Number of patients by age, stage, and comorbidity level: **Lung Cancer**

| Lung cancer | | | | | | | | | | | | | | | | | | | | | | | | | |
| --- | --- | --- | --- | --- | --- | --- | --- | --- | --- | --- | --- | --- | --- | --- | --- | --- | --- | --- | --- | --- | --- | --- | --- | --- | --- |
| Age | | 15-64 | | | | | | 65-74 | | | | | | 75-84 | | | | | | 85-94 | | | | | |
| Sex | | Male | | | Female | | | Male | | | Female | | | Male | | | Female | | | Male | | | Female | | |
| Comorbidity level* | | 0 | 1 | 2 | 0 | 1 | 2 | 0 | 1 | 2 | 0 | 1 | 2 | 0 | 1 | 2 | 0 | 1 | 2 | 0 | 1 | 2 | 0 | 1 | 2 |
| Stage** | NA^+^ | 945 | 328 | 258 | 521 | 201 | 91 | 694 | 504 | 686 | 276 | 326 | 241 | 791 | 797 | 1558 | 252 | 447 | 539 | 235 | 279 | 641 | 151 | 232 | 300 |
|  | Localized | 1458 | 908 | 420 | 2035 | 1079 | 395 | 584 | 834 | 723 | 468 | 788 | 430 | 385 | 577 | 1005 | 156 | 440 | 349 | 58 | 103 | 242 | 19 | 64 | 70 |
|  | Regional | 2393 | 1068 | 612 | 1420 | 663 | 275 | 1322 | 1208 | 1228 | 454 | 638 | 363 | 955 | 1227 | 1862 | 212 | 520 | 418 | 178 | 221 | 470 | 72 | 125 | 157 |
|  | Distant | 7678 | 3027 | 1350 | 5915 | 2274 | 753 | 3463 | 3302 | 2494 | 1839 | 2426 | 1127 | 2489 | 3342 | 4174 | 1042 | 2261 | 1582 | 566 | 865 | 1330 | 302 | 680 | 592 |
|  |  | X-squared =  151.4, p-value <  2.2e-16 | | | X-squared =  91.334, p-value <  2.2e-16 | | | X-squared =  116.3, p-value <  2.2e-16 | | | X-squared =  34.998, p-value =  4.649e-07 | | | X-squared =  71.589, p-value =  1.048e-14 | | | X-squared =  19.346, p-value =  0.0006719 | | | X-squared =  29.34,  p-value =  6.666e-06 | | | X-squared =  13.221, p-value =  0.01024 | | |
|  | Non-distant | 46 | 18 | 13 | 28 | 10 | 6 | 14 | 21 | 30 | 7 | <5 | 11 | 27 | 23 | 65 | 11 | 18 | 31 | 18 | 20 | 38 | <5 | 17 | 19 |
|  | Unknown^++^ | 332 | 145 | 90 | 176 | 69 | 48 | 238 | 181 | 205 | 54 | 75 | 67 | 180 | 196 | 344 | 51 | 101 | 81 | 44 | 44 | 104 | 23 | 33 | 48 |

*comorbidity level: 0 refers to individuals with no comorbid conditions. 2 refers to conditions that include comorbid conditions 3, 6, 7, 11, 13, 16 or the comorbid score exceeded 0.66. 1 refers to individuals not classified into 0 nor 2 of comorbidity level.

** For patients whose cancer stage cannot be classified into localized, regional and distant stages, we categorize their cancer stage as non-distant stage if the metastatic information can be obtained from TCR-LF. Patients whose cancer stage cannot be classified into localized, regional, distant and non-distant stage is categorized as unknown stage.

^+^ Cancer patient in TCR but not in TCRLF.

^++^ Cancer patient in TCRLF stage information missing.

## Table S9-5. Number of patients by age, stage, and comorbidity level: **Oral Cancer**

| Oral cancer | | | | | | | | | | | | | | | | | | | | | | | | | |
| --- | --- | --- | --- | --- | --- | --- | --- | --- | --- | --- | --- | --- | --- | --- | --- | --- | --- | --- | --- | --- | --- | --- | --- | --- | --- |
| Age | | 15-64 | | | | | | 65-74 | | | | | | 75-84 | | | | | | 85-94 | | | | | |
| Sex | | Male | | | Female | | | Male | | | Female | | | Male | | | Female | | | Male | | | Female | | |
| Comorbidity level* | | 0 | 1 | 2 | 0 | 1 | 2 | 0 | 1 | 2 | 0 | 1 | 2 | 0 | 1 | 2 | 0 | 1 | 2 | 0 | 1 | 2 | 0 | 1 | 2 |
| Stage** | NA^+^ | 6013 | 1722 | 624 | 1589 | 424 | 122 | 473 | 424 | 353 | 113 | 145 | 77 | 170 | 172 | 237 | 56 | 77 | 66 | 20 | 32 | 35 | 9 | 19 | 21 |
|  | Localized | 10510 | 4528 | 1534 | 1129 | 480 | 178 | 839 | 996 | 619 | 135 | 256 | 125 | 180 | 339 | 327 | 53 | 159 | 118 | 36 | 43 | 72 | 9 | 32 | 32 |
|  | Regional | 19379 | 6168 | 2284 | 1966 | 614 | 192 | 1461 | 1298 | 875 | 163 | 264 | 141 | 412 | 467 | 611 | 95 | 156 | 118 | 77 | 93 | 146 | 26 | 42 | 47 |
|  | Distant | 878 | 254 | 115 | 92 | 23 | 9 | 107 | 63 | 62 | 9 | 19 | 10 | 35 | 48 | 42 | 6 | 10 | 10 | 7 | 8 | 16 | <5 | <5 | <5 |
|  |  | X-squared =  197.34, p-value <  2.2e-16 | | | X-squared =  34.364, p-value =  6.274e-07 | | | X-squared =  35.159,  p-value =  4.308e-07 | | | X-squared =  1.4998, p-value =  0.8267 | | | X-squared =  23.333, p-value =  0.0001086 | | | X-squared =  10.23, p-value =  0.03672 | | | X-squared = 0.38399, p-value =  0.9838 | | | X-squared = NaN,  p-value = NA | | |
|  | Non-distant | 254 | 77 | 41 | 43 | 11 | <5 | 16 | 10 | 15 | <5 | <5 | <5 | 13 | 20 | 10 | <5 | 7 | 8 | <5 | 5 | 7 | 0 | 5 | <5 |
|  | Unknown^++^ | 699 | 220 | 93 | 91 | 26 | 6 | 70 | 53 | 53 | 18 | 18 | 14 | 31 | 34 | 39 | 6 | 10 | 8 | 6 | 12 | 12 | <5 | <5 | 7 |

*comorbidity level: 0 refers to individuals with no comorbid conditions. 2 refers to conditions that include comorbid conditions 3, 6, 7, 11, 13, 16 or the comorbid score exceeded 0.66. 1 refers to individuals not classified into 0 nor 2 of comorbidity level.

** For patients whose cancer stage cannot be classified into localized, regional and distant stages, we categorize their cancer stage as non-distant stage if the metastatic information can be obtained from TCR-LF. Patients whose cancer stage cannot be classified into localized, regional, distant and non-distant stage is categorized as unknown stage.

^+^ Cancer patient in TCR but not in TCRLF.

^++^ Cancer patient in TCRLF stage information missing.

## Table S10-1. Numbers of cancer patients in TCR by year of diagnosis and age of diagnosis and the number and percentage of them in TCRLF: **breast cancer**

| Year of diagnosis | TCR | TCRLF | | TCRLF/TCR | 15-64 | | 65-94 | |  |
| --- | --- | --- | --- | --- | --- | --- | --- | --- | --- |
| sex | Female | | Female | (%) | | Female | | Female | |
| 2004 | 6066 | | 4337 | 71.5 | | 5123 | | 943 | |
| 2005 | 6465 | | 4911 | 76.0 | | 5447 | | 1018 | |
| 2006 | 6762 | | 5271 | 78.0 | | 5679 | | 1083 | |
| 2007 | 7372 | | 6344 | 86.1 | | 6089 | | 1283 | |
| 2008 | 7825 | | 6660 | 85.1 | | 6501 | | 1324 | |
| 2009 | 8429 | | 7406 | 87.9 | | 6884 | | 1545 | |
| 2010 | 9115 | | 8586 | 94.2 | | 7466 | | 1649 | |
| 2011 | 9394 | | 8840 | 94.1 | | 7639 | | 1755 | |
| 2012 | 9789 | | 9298 | 95.0 | | 7955 | | 1834 | |
| 2013 | 10377 | | 9936 | 95.8 | | 8295 | | 2082 | |
| 2014 | 10749 | | 10398 | 96.7 | | 8531 | | 2218 | |
| Age of diagnosis |  | |  |  | |  | |  | |
| 15-34 | 3555 | |  |  | |  | |  | |
| 35-49 | 33565 | |  |  | |  | |  | |
| 50-64 | 38489 | |  |  | |  | |  | |
| 65-74 | 11193 | |  |  | |  | |  | |
| 75-84 | 4580 | |  |  | |  | |  | |
| 85-94 | 961 | |  |  | |  | |  | |

## Table S10-2. Numbers of cancer patients in TCR by year of diagnosis, age of diagnosis and sex and the number and percentage of them in TCRLF: **colorectal cancer**

| Year of diagnosis | TCR | | | TCRLF | TCRLF/TCR | 15-64 | | 65-94 | | | |  |
| --- | --- | --- | --- | --- | --- | --- | --- | --- | --- | --- | --- | --- |
| sex | All | Male | Female | All | (%) | Male | Female | | Male | Female | | |
| 2004 | 8455 | 4839 | 3616 | 5693 | 67.3 | 1993 | 1570 | | 2846 | 2046 | | |
| 2005 | 8638 | 4960 | 3678 | 6087 | 70.5 | 1958 | 1625 | | 3002 | 2053 | | |
| 2006 | 9094 | 5169 | 3925 | 6548 | 72.0 | 2160 | 1789 | | 3009 | 2136 | | |
| 2007 | 9634 | 5557 | 4077 | 7937 | 82.4 | 2276 | 1806 | | 3281 | 2271 | | |
| 2008 | 9826 | 5656 | 4170 | 8217 | 83.6 | 2354 | 1776 | | 3302 | 2394 | | |
| 2009 | 10633 | 6105 | 4528 | 9242 | 86.9 | 2626 | 1923 | | 3479 | 2605 | | |
| 2010 | 11936 | 6944 | 4992 | 11052 | 92.6 | 3273 | 2260 | | 3671 | 2732 | | |
| 2011 | 11987 | 6972 | 5015 | 11236 | 93.7 | 3259 | 2317 | | 3713 | 2698 | | |
| 2012 | 12626 | 7264 | 5362 | 11855 | 93.9 | 3503 | 2495 | | 3761 | 2867 | | |
| 2013 | 12783 | 7343 | 5440 | 12005 | 93.9 | 3403 | 2482 | | 3940 | 2958 | | |
| 2014 | 13143 | 7582 | 5561 | 12474 | 94.9 | 3580 | 2556 | | 4002 | 3005 | | |
| Age of diagnosis |  |  |  |  |  |  |  | |  | |  |  |
| 15-34 | 2025 | 1085 | 940 |  |  |  |  | |  | |  |  |
| 35-49 | 12362 | 6521 | 5841 |  |  |  |  | |  | |  |  |
| 50-64 | 38597 | 22779 | 15818 |  |  |  |  | |  | |  |  |
| 65-74 | 29738 | 17698 | 12040 |  |  |  |  | |  | |  |  |
| 75-84 | 27385 | 15905 | 11480 |  |  |  |  | |  | |  |  |
| 85-94 | 8648 | 4403 | 4245 |  |  |  |  | |  | |  |  |

## Table S10-3. Numbers of cancer patients in TCR by year of diagnosis, age of diagnosis and sex and the number and percentage of them in TCRLF: **liver cancer**

| Year of diagnosis | TCR | | | TCRLF | TCRLF/TCR | 15-64 | | 65-94 | |
| --- | --- | --- | --- | --- | --- | --- | --- | --- | --- |
| sex | All | Male | Female | All | % | Male | Female | Male | Female |
| 2004 | 9597 | 6929 | 2668 | 7118 | 74.2 | 3944 | 975 | 2985 | 1693 |
| 2005 | 9772 | 7064 | 2708 | 7476 | 76.5 | 4027 | 974 | 3037 | 1734 |
| 2006 | 9985 | 7107 | 2878 | 7956 | 79.7 | 3876 | 1003 | 3231 | 1875 |
| 2007 | 10285 | 7353 | 2932 | 9099 | 88.5 | 4088 | 1015 | 3265 | 1917 |
| 2008 | 10245 | 7194 | 3051 | 9163 | 89.4 | 4019 | 989 | 3175 | 2062 |
| 2009 | 10538 | 7414 | 3124 | 9706 | 92.1 | 4104 | 989 | 3310 | 2135 |
| 2010 | 10424 | 7375 | 3049 | 9839 | 94.4 | 4182 | 964 | 3193 | 2085 |
| 2011 | 10480 | 7403 | 3077 | 9987 | 95.3 | 4152 | 953 | 3251 | 2124 |
| 2012 | 10483 | 7331 | 3152 | 9999 | 95.4 | 4107 | 966 | 3224 | 2186 |
| 2013 | 10542 | 7348 | 3194 | 10246 | 97.2 | 4051 | 1001 | 3297 | 2193 |
| 2014 | 10136 | 7045 | 3091 | 9889 | 97.6 | 3955 | 952 | 3090 | 2139 |
| Age of diagnosis |  |  |  |  |  |  |  |  |  |
| 15-34 | 1682 | 1323 | 359 |  |  |  |  |  |  |
| 35-49 | 13794 | 12105 | 1689 |  |  |  |  |  |  |
| 50-64 | 39810 | 31077 | 8733 |  |  |  |  |  |  |
| 65-74 | 30769 | 19616 | 11153 |  |  |  |  |  |  |
| 75-84 | 21566 | 12746 | 8820 |  |  |  |  |  |  |
| 85-94 | 4866 | 2696 | 2170 |  |  |  |  |  |  |

## Table S10-4. Numbers of cancer patients in TCR by year of diagnosis, age of diagnosis and sex and the number and percentage of them in TCRLF: **lung cancer**

| Year of diagnosis | TCR | | | TCRLF | TCRLF/TCR | 15-64 | | 65-94 | |
| --- | --- | --- | --- | --- | --- | --- | --- | --- | --- |
| sex | All | Male | Female | All | % | Male | Female | Male | Female |
| 2004 | 7587 | 5147 | 2440 | 5666 | 74.7 | 1443 | 971 | 3704 | 1469 |
| 2005 | 7929 | 5318 | 2611 | 6047 | 76.3 | 1575 | 1035 | 3743 | 1576 |
| 2006 | 8266 | 5451 | 2815 | 6456 | 78.1 | 1568 | 1129 | 3883 | 1686 |
| 2007 | 8639 | 5629 | 3010 | 7534 | 87.2 | 1701 | 1190 | 3928 | 1820 |
| 2008 | 8690 | 5673 | 3017 | 7721 | 88.8 | 1720 | 1273 | 3953 | 1744 |
| 2009 | 9533 | 6068 | 3465 | 8657 | 90.8 | 1932 | 1460 | 4136 | 2005 |
| 2010 | 9474 | 5963 | 3511 | 8787 | 92.7 | 1954 | 1496 | 4009 | 2015 |
| 2011 | 9818 | 6178 | 3640 | 9263 | 94.3 | 2070 | 1635 | 4108 | 2005 |
| 2012 | 10284 | 6247 | 4037 | 9865 | 95.9 | 2279 | 1805 | 3968 | 2232 |
| 2013 | 10300 | 6248 | 4052 | 9998 | 97.1 | 2433 | 1875 | 3815 | 2177 |
| 2014 | 10724 | 6351 | 4373 | 10487 | 97.8 | 2414 | 2090 | 3937 | 2283 |
| Age of diagnosis |  |  |  |  |  |  |  |  |  |
| 15-34 | 656 | 333 | 323 |  |  |  |  |  |  |
| 35-49 | 8182 | 4302 | 3880 |  |  |  |  |  |  |
| 50-64 | 28210 | 16454 | 11756 |  |  |  |  |  |  |
| 65-74 | 27325 | 17731 | 9594 |  |  |  |  |  |  |
| 75-84 | 28508 | 19997 | 8511 |  |  |  |  |  |  |
| 85-94 | 8363 | 5456 | 2907 |  |  |  |  |  |  |

## Table S10-5. Numbers of cancer patients in TCR by year of diagnosis, age of diagnosis and sex and the number and percentage of them in TCRLF: **oral cancer**

| Year of diagnosis | TCR | | | TCRLF | TCRLF/TCR | 15-64 | | | | 65-94 | | | | |  |
| --- | --- | --- | --- | --- | --- | --- | --- | --- | --- | --- | --- | --- | --- | --- | --- |
| sex | All | Male | Female | All | % | Male | Female | | Male | | | Female | |  |  |
| 2004 | 5845 | 5070 | 775 | 3452 | 59.1 | 4191 | 582 | | 879 | | | 193 | |  |  |
| 2005 | 5862 | 5055 | 807 | 3562 | 60.8 | 4250 | 578 | | 805 | | | 229 | |  |  |
| 2006 | 6316 | 5508 | 808 | 4028 | 63.8 | 4516 | 591 | | 992 | | | 217 | |  |  |
| 2007 | 6700 | 5820 | 880 | 4629 | 69.1 | 4837 | 628 | | 983 | | | 252 | |  |  |
| 2008 | 6778 | 5935 | 843 | 4755 | 70.2 | 4876 | 617 | | 1059 | | | 226 | |  |  |
| 2009 | 7261 | 6355 | 906 | 6766 | 93.2 | 5263 | 633 | | 1092 | | | 273 | |  |  |
| 2010 | 7387 | 6481 | 906 | 6973 | 94.4 | 5314 | 645 | | 1167 | | | 261 | |  |  |
| 2011 | 7522 | 6571 | 951 | 7333 | 97.5 | 5495 | 686 | | 1076 | | | 265 | |  |  |
| 2012 | 7635 | 6688 | 947 | 7437 | 97.4 | 5546 | 690 | | 1142 | | | 257 | |  |  |
| 2013 | 7581 | 6677 | 904 | 7463 | 98.4 | 5509 | 619 | | 1168 | | | 285 | |  |  |
| 2014 | 7856 | 6838 | 1018 | 7760 | 98.8 | 5596 | 730 | | 1242 | | | 288 | |  |  |
| Age of diagnosis |  |  |  |  |  |  | |  | | |  | |  | | |
| 15-34 |  | 2592 | 823 |  |  |  |  | |  | | |  | |  |  |
| 35-49 |  | 23569 | 2761 |  |  |  |  | |  | | |  | |  |  |
| 50-64 |  | 29232 | 3415 |  |  |  |  | |  | | |  | |  |  |
| 65-74 |  | 7787 | 1516 |  |  |  |  | |  | | |  | |  |  |
| 75-84 |  | 3187 | 966 |  |  |  |  | |  | | |  | |  |  |
| 85-94 |  | 631 | 264 |  |  |  |  | |  | | |  | |  |  |

## Table S11-1. Number and percentage of patients by stage and year of diagnosis: **breast cancer.**

| Stage | 2004 | | 2005 | | 2006 | | 2007 | | 2008 | | 2009 | | 2010 | | 2011 | | 2012 | | 2013 | | 2014 | |
| --- | --- | --- | --- | --- | --- | --- | --- | --- | --- | --- | --- | --- | --- | --- | --- | --- | --- | --- | --- | --- | --- | --- |
|  | N | % | N | % | N | % | N | % | N | % | N | % | N | % | N | % | N | % | N | % | N | % |
| Localized | 2313 | 53.3 | 2658 | 54.1 | 2741 | 52.0 | 3409 | 53.7 | 3556 | 53.4 | 4050 | 54.7 | 4786 | 55.7 | 4844 | 54.8 | 5267 | 56.6 | 5704 | 57.4 | 6009 | 57.8 |
| regional | 1686 | 38.9 | 1910 | 38.9 | 2073 | 39.3 | 2392 | 37.7 | 2494 | 37.4 | 2716 | 36.7 | 3184 | 37.1 | 3317 | 37.5 | 3300 | 35.5 | 3490 | 35.1 | 3600 | 34.6 |
| Distant | 199 | 4.6 | 210 | 4.3 | 312 | 5.9 | 414 | 6.5 | 478 | 7.2 | 480 | 6.5 | 461 | 5.4 | 475 | 5.4 | 501 | 5.4 | 514 | 5.2 | 587 | 5.6 |
| Non-distant | 22 | 0.5 | 30 | 0.6 | 24 | 0.5 | 22 | 0.3 | 22 | 0.3 | 43 | 0.6 | 92 | 1.1 | 95 | 1.1 | 106 | 1.1 | 138 | 1.4 | 132 | 1.3 |
| Unknown | 117 | 2.7 | 103 | 2.1 | 121 | 2.3 | 107 | 1.7 | 110 | 1.7 | 117 | 1.6 | 63 | 0.7 | 109 | 1.2 | 124 | 1.3 | 90 | 0.9 | 70 | 0.7 |

For patients whose cancer stage cannot be classified into localized, regional and distant stages, we categorize their cancer stage as non-distant stage if the metastatic information can be obtained from TCR-LF. Patients whose cancer stage cannot be classified into localized, regional, distant and non-distant stage is categorized as unknown stage.

## Table S11-2. Number and percentage of patients by stage and year of diagnosis: **colorectal cancer**.

| Stage | 2004 | | 2005 | | 2006 | | 2007 | | 2008 | | 2009 | | 2010 | | 2011 | | 2012 | | 2013 | | 2014 | |
| --- | --- | --- | --- | --- | --- | --- | --- | --- | --- | --- | --- | --- | --- | --- | --- | --- | --- | --- | --- | --- | --- | --- |
|  | N | % | N | % | N | % | N | % | N | % | N | % | N | % | N | % | N | % | N | % | N | % |
| Localized | 2082 | 36.6 | 2283 | 37.5 | 2484 | 37.9 | 3031 | 38.2 | 3031 | 36.9 | 3476 | 37.6 | 4521 | 40.9 | 4557 | 40.6 | 4904 | 41.4 | 4917 | 41.0 | 5136 | 41.2 |
| regional | 1918 | 33.7 | 2077 | 34.1 | 2129 | 32.5 | 2490 | 31.4 | 2725 | 33.2 | 3080 | 33.3 | 3946 | 35.7 | 4085 | 36.4 | 4099 | 34.6 | 4130 | 34.4 | 4351 | 34.9 |
| Distant | 1143 | 20.1 | 1228 | 20.2 | 1445 | 22.1 | 1905 | 24.0 | 1993 | 24.3 | 2113 | 22.9 | 1949 | 17.6 | 1964 | 17.5 | 2088 | 17.6 | 2113 | 17.6 | 2128 | 17.1 |
| Non-distant | 38 | 0.7 | 14 | 0.2 | 28 | 0.4 | 43 | 0.5 | 33 | 0.4 | 43 | 0.5 | 524 | 4.7 | 468 | 4.2 | 592 | 5.0 | 671 | 5.6 | 705 | 5.7 |
| Unknown | 512 | 9.0 | 485 | 8.0 | 462 | 7.1 | 468 | 5.9 | 435 | 5.3 | 530 | 5.7 | 112 | 1.0 | 162 | 1.4 | 172 | 1.5 | 174 | 1.4 | 154 | 1.2 |

For patients whose cancer stage cannot be classified into localized, regional and distant stages, we categorize their cancer stage as non-distant stage if the metastatic information can be obtained from TCR-LF. Patients whose cancer stage cannot be classified into localized, regional, distant and non-distant stage is categorized as unknown stage. The somewhat large differences in the percentages between 2009 and 2010 might be caused by the difference between AJCC 6^th^ edition and 7^th^ edition; see Table S8.

## Table S11-3. Number and percentage of patients by stage and year of diagnosis: **liver cancer**

| Stage | 2004 | | 2005 | | 2006 | | 2007 | | 2008 | | 2009 | | 2010 | | 2011 | | 2012 | | 2013 | | 2014 | |
| --- | --- | --- | --- | --- | --- | --- | --- | --- | --- | --- | --- | --- | --- | --- | --- | --- | --- | --- | --- | --- | --- | --- |
|  | N | % | N | % | N | % | N | % | N | % | N | % | N | % | N | % | N | % | N | % | N | % |
| Localized | 5071 | 71.2 | 5580 | 74.6 | 5707 | 71.7 | 6556 | 72.1 | 6785 | 74.0 | 7275 | 75.0 | 5898 | 59.9 | 6007 | 60.1 | 6046 | 60.5 | 6120 | 59.7 | 6001 | 60.7 |
| regional | 585 | 8.2 | 548 | 7.3 | 667 | 8.4 | 847 | 9.3 | 805 | 8.8 | 924 | 9.5 | 2268 | 23.1 | 2233 | 22.4 | 2214 | 22.1 | 2201 | 21.5 | 2110 | 21.3 |
| Distant | 737 | 10.4 | 735 | 9.8 | 910 | 11.4 | 1167 | 12.8 | 1149 | 12.5 | 1160 | 12.0 | 1205 | 12.2 | 1160 | 11.6 | 1101 | 11.0 | 1215 | 11.9 | 1216 | 12.3 |
| Non-distant | 5 | 0.1 | <5 | 0.0 | - | - | 5 | 0.1 | <5 | 0.0 | <5 | 0.0 | 145 | 1.5 | 150 | 1.5 | 164 | 1.6 | 209 | 2.0 | 187 | 1.9 |
| Unknown | 720 | 10.1 | 610 | 8.2 | 672 | 8.4 | 524 | 5.8 | 422 | 4.6 | 343 | 3.5 | 323 | 3.3 | 437 | 4.4 | 474 | 4.7 | 501 | 4.9 | 375 | 3.8 |

For patients whose cancer stage cannot be classified into localized, regional and distant stages, we categorize their cancer stage as non-distant stage if the metastatic information can be obtained from TCR-LF. Patients whose cancer stage cannot be classified into localized, regional, distant and non-distant stage is categorized as unknown stage. The somewhat large differences in the percentages between 2009 and 2010 might be caused by the difference between AJCC 6^th^ edition and 7^th^ edition; see Table S8.

## Table S11-4. Number and percentage of patients by stage and year of diagnosis: **lung cancer.**

| Stage | 2004 | | 2005 | | 2006 | | 2007 | | 2008 | | 2009 | | 2010 | | 2011 | | 2012 | | 2013 | | 2014 | |
| --- | --- | --- | --- | --- | --- | --- | --- | --- | --- | --- | --- | --- | --- | --- | --- | --- | --- | --- | --- | --- | --- | --- |
|  | N | % | N | % | N | % | N | % | N | % | N | % | N | % | N | % | N | % | N | % | N | % |
| Localized | 628 | 11.1 | 736 | 12.2 | 776 | 12.0 | 983 | 13.0 | 1070 | 13.9 | 1408 | 16.3 | 1287 | 14.6 | 1302 | 14.1 | 1656 | 16.8 | 1788 | 17.9 | 2012 | 19.2 |
| regional | 1665 | 29.4 | 1631 | 27.0 | 1758 | 27.2 | 1971 | 26.2 | 1990 | 25.8 | 2099 | 24.2 | 1410 | 16.0 | 1384 | 14.9 | 1432 | 14.5 | 1383 | 13.8 | 1436 | 13.7 |
| Distant | 3030 | 53.5 | 3223 | 53.3 | 3521 | 54.5 | 4259 | 56.5 | 4381 | 56.7 | 4925 | 56.9 | 5851 | 66.6 | 6287 | 67.9 | 6475 | 65.6 | 6467 | 64.7 | 6743 | 64.3 |
| Non-distant | <5 | 0.0 |  | 0.0 | <5 | - | <5 | 0.0 | <5 | 0.0 |  | 0.0 | 103 | 1.2 | 86 | 0.9 | 101 | 1.0 | 110 | 1.1 | 113 | 1.1 |
| Unknown | 341 | 6.0 | 457 | 7.6 | 400 | 6.2 | 320 | 4.2 | 278 | 3.6 | 225 | 2.6 | 136 | 1.5 | 204 | 2.2 | 201 | 2.0 | 250 | 2.5 | 183 | 1.7 |

For patients whose cancer stage cannot be classified into localized, regional and distant stages, we categorize their cancer stage as non-distant stage if the metastatic information can be obtained from TCR-LF. Patients whose cancer stage cannot be classified into localized, regional, distant and non-distant stage is categorized as unknown stage. The somewhat large differences in the percentages between 2009 and 2010 might have something to do with the difference between AJCC 6^th^ edition and 7^th^ edition; see Table S8.

## Table S11-5. Number and percentage of patients by stage and year of diagnosis: **oral cancer.**

| Stage | 2004 | | 2005 | | 2006 | | 2007 | | 2008 | | 2009 | | 2010 | | 2011 | | 2012 | | 2013 | | 2014 | |
| --- | --- | --- | --- | --- | --- | --- | --- | --- | --- | --- | --- | --- | --- | --- | --- | --- | --- | --- | --- | --- | --- | --- |
|  | N | % | N | % | N | % | N | % | N | % | N | % | N | % | N | % | N | % | N | % | N | % |
| Localized | 1312 | 38.0 | 1382 | 38.8 | 1484 | 36.8 | 1779 | 38.4 | 1804 | 37.9 | 2396 | 35.4 | 2394 | 34.3 | 2514 | 34.3 | 2581 | 34.7 | 2573 | 34.5 | 2649 | 34.1 |
| regional | 1886 | 54.6 | 1938 | 54.4 | 2293 | 56.9 | 2592 | 56.0 | 2707 | 56.9 | 3961 | 58.5 | 4146 | 59.5 | 4387 | 59.8 | 4362 | 58.7 | 4402 | 59.0 | 4638 | 59.8 |
| Distant | 83 | 2.4 | 64 | 1.8 | 78 | 1.9 | 117 | 2.5 | 112 | 2.4 | 223 | 3.3 | 242 | 3.5 | 207 | 2.8 | 227 | 3.1 | 243 | 3.3 | 240 | 3.1 |
| Non-distant | <5 | 0.1 | <5 | 0.1 | <5 | - | <5 | 0.0 | <5 | 0.1 | <5 | 0.1 | 80 | 1.1 | 106 | 1.4 | 113 | 1.5 | 132 | 1.8 | 132 | 1.7 |
| Unknown | 167 | 4.8 | 175 | 4.9 | 170 | 4.2 | 140 | 3.0 | 129 | 2.7 | 182 | 2.7 | 111 | 1.6 | 119 | 1.6 | 154 | 2.1 | 113 | 1.5 | 101 | 1.3 |

For patients whose cancer stage cannot be classified into localized, regional and distant stages, we categorize their cancer stage as non-distant stage if the metastatic information can be obtained from TCR-LF. Patients whose cancer stage cannot be classified into localized, regional, distant and non-distant stage is categorized as unknown stage.

## Table S12-1-1. Number of patients and five-year probabilities and the confidence intervals of dying from cancer, dying from other-causes, and survival by stage, age and comorbidity level: **female breast cancer**

| Stage | Age at diagnosis | Comorbidity | N | Survival (%) | | | Cancer deaths (%) | | | Other-cause deaths (%) | | |
| --- | --- | --- | --- | --- | --- | --- | --- | --- | --- | --- | --- | --- |
|  |  |  |  | Estimate | 95%CI. | | Estimate | 95%CI. | | Estimate | 95%CI. | |
| Localized | 65-74 | 0 | 1592 | 92.0 | 89.5 | 94.0 | 5.7 | 4.4 | 7.2 | 2.3 | 1.6 | 3.3 |
|  |  | 1 | 2688 | 90.4 | 88.2 | 92.2 | 5.8 | 4.8 | 6.9 | 3.9 | 3.0 | 4.8 |
|  |  | 2 | 1043 | 80.3 | 75.7 | 84.4 | 8.6 | 6.8 | 10.8 | 11.0 | 8.9 | 13.5 |
|  | 75-84 | 0 | 356 | 80.9 | 72.7 | 87.4 | 11.4 | 7.8 | 15.8 | 7.7 | 4.8 | 11.5 |
|  |  | 1 | 1034 | 81.3 | 76.8 | 85.3 | 8.4 | 6.5 | 10.6 | 10.3 | 8.2 | 12.7 |
|  |  | 2 | 696 | 57.6 | 50.3 | 64.5 | 16.3 | 13.2 | 19.6 | 26.1 | 22.3 | 30.1 |
|  | 85-94 | 0 | 58 | 63.0 | 37.2 | 82.9 | 17.6 | 7.9 | 30.4 | 19.4 | 9.2 | 32.4 |
|  |  | 1 | 161 | 50.5 | 34.4 | 65.1 | 18.4 | 12.0 | 25.9 | 31.2 | 23.0 | 39.7 |
|  |  | 2 | 172 | 38.8 | 23.2 | 53.7 | 23.5 | 16.7 | 31.0 | 37.7 | 29.6 | 45.8 |
| Regional | 65-74 | 0 | 1059 | 72.0 | 67.4 | 76.1 | 24.1 | 21.2 | 27.2 | 3.9 | 2.7 | 5.4 |
|  |  | 1 | 1704 | 73.9 | 70.2 | 77.2 | 20.6 | 18.4 | 22.8 | 5.6 | 4.4 | 6.9 |
|  |  | 2 | 722 | 66.1 | 59.8 | 71.9 | 20.9 | 17.7 | 24.3 | 13.0 | 10.4 | 15.9 |
|  | 75-84 | 0 | 244 | 55.8 | 43.6 | 66.7 | 32.1 | 25.5 | 38.9 | 12.1 | 7.8 | 17.4 |
|  |  | 1 | 665 | 57.3 | 50.1 | 64.0 | 31.5 | 27.4 | 35.7 | 11.3 | 8.6 | 14.3 |
|  |  | 2 | 435 | 42.1 | 32.0 | 51.9 | 36.5 | 31.2 | 41.7 | 21.4 | 16.9 | 26.3 |
|  | 85-94 | 0 | 40 | 24.0 | -8.6 | 56.7 | 42.4 | 25.4 | 58.4 | 33.6 | 17.8 | 50.3 |
|  |  | 1 | 116 | 32.0 | 12.7 | 50.8 | 46.3 | 35.7 | 56.3 | 21.6 | 13.5 | 31.0 |
|  |  | 2 | 105 | 20.8 | -0.8 | 42.8 | 39.9 | 29.1 | 50.4 | 39.4 | 28.1 | 50.4 |
| Distant | 65-74 | 0 | 268 | 17.0 | 9.0 | 25.1 | 79.7 | 73.5 | 84.6 | 3.3 | 1.4 | 6.4 |
|  |  | 1 | 268 | 17.0 | 8.0 | 26.2 | 76.0 | 69.7 | 81.2 | 7.0 | 4.1 | 10.9 |
|  |  | 2 | 119 | 13.7 | -1.4 | 29.5 | 72.6 | 62.5 | 80.4 | 13.8 | 8.0 | 21.0 |
|  | 75-84 | 0 | 84 | 9.6 | -7.0 | 27.7 | 78.5 | 66.6 | 86.6 | 11.8 | 5.7 | 20.4 |
|  |  | 1 | 136 | 12.6 | -0.9 | 27.1 | 77.9 | 68.0 | 85.1 | 9.4 | 4.9 | 15.8 |
|  |  | 2 | 104 | 17.6 | 0.8 | 34.7 | 69.8 | 58.9 | 78.4 | 12.6 | 6.4 | 20.8 |
|  | 85-94 | 0 | 14 | 25.0 | -20.1 | 70.8 | 60.7 | 27.1 | 82.6 | 14.3 | 2.1 | 37.5 |
|  |  | 1 | 45 | 9.6 | -14.9 | 36.7 | 75.6 | 57.6 | 86.7 | 14.9 | 5.7 | 28.2 |
|  |  | 2 | 20 | 34.3 | -7.7 | 73.0 | 35.7 | 15.2 | 56.9 | 30.0 | 11.8 | 50.8 |

## Table S12-1-2. One and two-year probabilities and their confidence intervals of dying from cancer, dying from other-causes, and survival by stage, age, and comorbidity level: **female breast cancer**

| Stage | Age at diagnosis | Comorbidity | One-year (%) | | | | | | | | | Two-year (%) | | | | | | | | |
| --- | --- | --- | --- | --- | --- | --- | --- | --- | --- | --- | --- | --- | --- | --- | --- | --- | --- | --- | --- | --- |
|  |  |  | Survival | | | Cancer deaths | | | Other-cause deaths | | | Survival | | | Cancer deaths | | | Other-cause deaths | | |
|  |  |  | Est. | 95%CI. | | Est. | 95%CI. | | Est. | 95%CI. | | Est. | 95%CI. | | Est. | 95%CI. | | Est. | 95%CI. | |
| Localized | 65-74 | 0 | 98.9 | 97.9 | 99.4 | 0.9 | 0.5 | 1.4 | 0.3 | 0.1 | 0.6 | 97.8 | 96.6 | 98.7 | 1.5 | 1.0 | 2.2 | 0.7 | 0.3 | 1.2 |
|  |  | 1 | 98.8 | 98.1 | 99.3 | 0.6 | 0.3 | 0.9 | 0.6 | 0.4 | 1.0 | 97.3 | 96.3 | 98.1 | 1.3 | 0.9 | 1.8 | 1.4 | 1.0 | 1.9 |
|  |  | 2 | 97.5 | 95.9 | 98.6 | 1.0 | 0.5 | 1.7 | 1.5 | 0.9 | 2.4 | 94.0 | 91.6 | 95.9 | 2.5 | 1.7 | 3.7 | 3.4 | 2.4 | 4.7 |
|  | 75-84 | 0 | 97.5 | 94.2 | 99.1 | 1.7 | 0.7 | 3.5 | 0.8 | 0.2 | 2.3 | 94.5 | 90.2 | 97.2 | 3.2 | 1.7 | 5.5 | 2.3 | 1.1 | 4.3 |
|  |  | 1 | 98.1 | 96.6 | 99.0 | 0.5 | 0.2 | 1.1 | 1.5 | 0.9 | 2.3 | 95.3 | 93.1 | 96.9 | 2.2 | 1.4 | 3.3 | 2.5 | 1.7 | 3.6 |
|  |  | 2 | 94.0 | 91.0 | 96.1 | 3.4 | 2.3 | 5.0 | 2.6 | 1.6 | 4.0 | 87.3 | 83.2 | 90.6 | 5.8 | 4.2 | 7.7 | 6.9 | 5.2 | 9.1 |
|  | 85-94 | 0 | 93.1 | 78.7 | 98.7 | 3.4 | 0.6 | 10.7 | 3.4 | 0.6 | 10.7 | 85.4 | 67.7 | 95.3 | 5.2 | 1.4 | 13.2 | 9.4 | 3.4 | 19.1 |
|  |  | 1 | 91.9 | 84.2 | 96.5 | 3.7 | 1.5 | 7.5 | 4.4 | 1.9 | 8.3 | 86.7 | 77.5 | 93.1 | 5.7 | 2.8 | 10.1 | 7.6 | 4.1 | 12.4 |
|  |  | 2 | 83.7 | 74.6 | 90.4 | 4.1 | 1.8 | 7.8 | 12.2 | 7.8 | 17.6 | 69.4 | 57.9 | 79.0 | 8.4 | 4.8 | 13.3 | 22.2 | 16.2 | 28.8 |
| Regional | 65-74 | 0 | 97.1 | 95.4 | 98.2 | 2.3 | 1.5 | 3.3 | 0.7 | 0.3 | 1.3 | 91.5 | 88.9 | 93.6 | 7.1 | 5.7 | 8.8 | 1.4 | 0.8 | 2.2 |
|  |  | 1 | 97.5 | 96.3 | 98.3 | 1.8 | 1.3 | 2.5 | 0.7 | 0.4 | 1.2 | 92.8 | 91.0 | 94.4 | 5.9 | 4.8 | 7.1 | 1.3 | 0.8 | 1.9 |
|  |  | 2 | 94.5 | 91.7 | 96.5 | 3.2 | 2.1 | 4.7 | 2.4 | 1.4 | 3.7 | 86.8 | 82.8 | 90.1 | 8.6 | 6.7 | 10.9 | 4.6 | 3.2 | 6.3 |
|  | 75-84 | 0 | 93.0 | 87.7 | 96.1 | 6.6 | 3.9 | 10.1 | 0.4 | 0.0 | 2.1 | 81.9 | 74.0 | 87.8 | 15.1 | 10.8 | 20.0 | 3.1 | 1.4 | 6.0 |
|  |  | 1 | 94.9 | 92.1 | 96.8 | 3.8 | 2.5 | 5.4 | 1.4 | 0.7 | 2.5 | 85.5 | 81.2 | 89.0 | 10.7 | 8.5 | 13.3 | 3.8 | 2.5 | 5.5 |
|  |  | 2 | 87.4 | 82.4 | 91.2 | 9.7 | 7.1 | 12.7 | 3.0 | 1.7 | 4.9 | 73.2 | 66.5 | 79.1 | 19.7 | 16.0 | 23.7 | 7.1 | 4.8 | 9.8 |
|  | 85-94 | 0 | 87.4 | 66.6 | 96.7 | 10.1 | 3.2 | 21.9 | 2.5 | 0.2 | 11.4 | 65.2 | 38.4 | 84.3 | 26.7 | 13.6 | 41.6 | 8.2 | 2.0 | 20.0 |
|  |  | 1 | 88.8 | 78.4 | 95.1 | 7.8 | 3.8 | 13.6 | 3.4 | 1.1 | 8.0 | 74.5 | 61.2 | 84.7 | 20.1 | 13.2 | 28.2 | 5.3 | 2.2 | 10.5 |
|  |  | 2 | 74.3 | 60.0 | 85.3 | 16.2 | 9.9 | 23.9 | 9.5 | 4.9 | 16.1 | 54.2 | 37.3 | 69.3 | 28.2 | 19.8 | 37.1 | 17.6 | 10.9 | 25.6 |
| Distant | 65-74 | 0 | 74.2 | 68.8 | 79.3 | 25.8 | 20.7 | 31.2 | 0.0 | NA | NA | 53.0 | 47.0 | 59.3 | 47.0 | 40.7 | 53.0 | 0.0 | NA | NA |
|  |  | 1 | 75.3 | 68.2 | 81.0 | 23.6 | 18.7 | 28.8 | 1.1 | 0.3 | 3.0 | 54.2 | 45.9 | 61.6 | 43.5 | 37.5 | 49.4 | 2.3 | 0.9 | 4.7 |
|  |  | 2 | 63.7 | 49.9 | 75.0 | 30.4 | 22.4 | 38.8 | 5.9 | 2.6 | 11.2 | 38.0 | 22.9 | 52.2 | 52.5 | 42.7 | 61.3 | 9.5 | 5.0 | 15.8 |
|  | 75-84 | 0 | 59.2 | 42.8 | 72.7 | 36.0 | 25.8 | 46.3 | 4.8 | 1.5 | 10.9 | 29.7 | 12.5 | 46.3 | 61.6 | 49.9 | 71.4 | 8.7 | 3.8 | 16.2 |
|  |  | 1 | 52.6 | 40.0 | 63.7 | 42.9 | 34.5 | 51.1 | 4.4 | 1.8 | 8.9 | 40.6 | 27.5 | 52.8 | 53.4 | 44.4 | 61.5 | 6.0 | 2.8 | 11.0 |
|  |  | 2 | 64.4 | 50.7 | 75.4 | 32.7 | 23.9 | 41.8 | 2.9 | 0.8 | 7.6 | 36.3 | 20.6 | 51.0 | 55.7 | 45.2 | 64.9 | 8.1 | 3.7 | 14.6 |
|  | 85-94 | 0 | 50.0 | 1.9 | 85.8 | 35.7 | 12.0 | 60.6 | 14.3 | 2.1 | 37.5 | 25.0 | -20.1 | 70.8 | 60.7 | 27.1 | 82.6 | 14.3 | 2.1 | 37.5 |
|  |  | 1 | 42.2 | 19.8 | 61.6 | 53.3 | 37.6 | 66.7 | 4.4 | 0.8 | 13.5 | 30.6 | 6.8 | 53.0 | 60.3 | 44.1 | 73.1 | 9.2 | 2.8 | 20.1 |
|  |  | 2 | 40.0 | -1.5 | 76.4 | 30.0 | 11.8 | 50.7 | 30.0 | 11.8 | 50.8 | 34.3 | -7.7 | 73.0 | 35.7 | 15.2 | 56.9 | 30.0 | 11.8 | 50.8 |

## Table S12-2-1. Number of patients and five-year probabilities of dying from cancer, dying from other-causes, and survival by stage, age and comorbidity level: **male colorectal cancer**

| Stage | Age at diagnosis | Comorbidity | N | Survival (%) | | | Cancer deaths (%) | | | Other-cause deaths (%) | | |
| --- | --- | --- | --- | --- | --- | --- | --- | --- | --- | --- | --- | --- |
|  |  |  |  | Estimate | 95%CI. | | Estimate | 95%CI. | | Estimate | 95%CI. | |
| Localized | 65-74 | 0 | 1975 | 80.8 | 77.8 | 83.5 | 14.0 | 12.3 | 15.8 | 5.2 | 4.2 | 6.4 |
|  |  | 1 | 2657 | 79.7 | 76.9 | 82.2 | 12.2 | 10.8 | 13.7 | 8.1 | 6.9 | 9.4 |
|  |  | 2 | 1629 | 66.3 | 62.1 | 70.3 | 16.1 | 14.2 | 18.2 | 17.6 | 15.5 | 19.7 |
|  | 75-84 | 0 | 1181 | 63.7 | 58.8 | 68.4 | 23.2 | 20.6 | 25.9 | 13.1 | 11.0 | 15.3 |
|  |  | 1 | 2058 | 61.4 | 57.5 | 65.2 | 22.2 | 20.3 | 24.3 | 16.4 | 14.6 | 18.2 |
|  |  | 2 | 2025 | 46.0 | 41.7 | 50.2 | 26.1 | 24.1 | 28.2 | 27.8 | 25.7 | 30.0 |
|  | 85-94 | 0 | 254 | 34.3 | 21.6 | 46.8 | 38.9 | 32.4 | 45.4 | 26.7 | 20.8 | 33.0 |
|  |  | 1 | 446 | 43.9 | 34.3 | 53.2 | 30.0 | 25.3 | 34.8 | 26.1 | 21.5 | 30.9 |
|  |  | 2 | 628 | 25.9 | 17.7 | 34.1 | 38.8 | 34.7 | 42.9 | 35.3 | 31.2 | 39.4 |
| Regional | 65-74 | 0 | 1777 | 53.6 | 49.8 | 57.2 | 41.5 | 39.0 | 44.1 | 4.9 | 3.9 | 6.1 |
|  |  | 1 | 2036 | 55.8 | 52.2 | 59.3 | 37.3 | 34.9 | 39.6 | 6.9 | 5.8 | 8.2 |
|  |  | 2 | 1149 | 46.5 | 41.1 | 51.8 | 40.2 | 37.0 | 43.4 | 13.2 | 11.1 | 15.5 |
|  | 75-84 | 0 | 1073 | 39.9 | 34.7 | 45.0 | 50.2 | 46.9 | 53.4 | 9.9 | 8.0 | 11.9 |
|  |  | 1 | 1671 | 39.0 | 34.6 | 43.3 | 49.1 | 46.4 | 51.7 | 11.9 | 10.3 | 13.7 |
|  |  | 2 | 1489 | 30.2 | 25.3 | 35.0 | 52.3 | 49.5 | 55.0 | 17.6 | 15.5 | 19.7 |
|  | 85-94 | 0 | 236 | 25.7 | 13.9 | 37.4 | 58.9 | 51.7 | 65.3 | 15.4 | 10.8 | 20.8 |
|  |  | 1 | 384 | 17.4 | 7.8 | 27.1 | 63.7 | 58.0 | 68.8 | 18.9 | 14.8 | 23.4 |
|  |  | 2 | 494 | 13.5 | 4.8 | 22.4 | 61.2 | 56.4 | 65.7 | 25.2 | 21.2 | 29.5 |
| Distant | 65-74 | 0 | 1023 | 9.3 | 5.9 | 12.9 | 86.1 | 83.7 | 88.2 | 4.6 | 3.4 | 6.0 |
|  |  | 1 | 1095 | 9.3 | 6.0 | 12.6 | 87.1 | 84.8 | 89.1 | 3.6 | 2.6 | 4.9 |
|  |  | 2 | 626 | 5.9 | 1.2 | 10.8 | 86.8 | 83.8 | 89.3 | 7.3 | 5.4 | 9.5 |
|  | 75-84 | 0 | 625 | 4.2 | 0.2 | 8.3 | 91.2 | 88.6 | 93.3 | 4.6 | 3.1 | 6.5 |
|  |  | 1 | 1032 | 4.1 | 1.0 | 7.3 | 91.0 | 89.0 | 92.6 | 4.9 | 3.7 | 6.4 |
|  |  | 2 | 925 | 3.7 | -0.2 | 7.8 | 87.9 | 85.6 | 89.9 | 8.4 | 6.7 | 10.3 |
|  | 85-94 | 0 | 152 | 0.7 | -7.0 | 9.3 | 93.4 | 87.8 | 96.5 | 6.0 | 2.9 | 10.5 |
|  |  | 1 | 222 | 1.5 | -6.7 | 10.3 | 88.2 | 83.0 | 91.9 | 10.3 | 6.6 | 14.8 |
|  |  | 2 | 322 | 0.9 | -5.7 | 7.8 | 89.5 | 85.6 | 92.4 | 9.6 | 6.7 | 13.3 |

## Table S12-2-2. One and Two-year probabilities of dying from cancer, dying from other-causes, and survival by stage, age and comorbidity level: **male colorectal cancer**

| Stage | Age at diagnosis | Comorbidity | One-year (%) | | | | | | | | | | Two-year (%) | | | | | | | | | |
| --- | --- | --- | --- | --- | --- | --- | --- | --- | --- | --- | --- | --- | --- | --- | --- | --- | --- | --- | --- | --- | --- | --- |
|  |  |  | Survival | | | Cancer deaths | | | Other-cause deaths | | | | Survival | | | Cancer deaths | | | Other-cause deaths | | | |
|  |  |  | Est. | 95%CI. | | Est. | 95%CI. | | Est. | 95%CI. | | Est. | | 95%CI. | | Est. | 95%CI. | | Est. | 95%CI. | |  |
| Localized | 65-74 | 0 | 97.1 | 95.9 | 98.0 | 2.1 | 1.6 | 2.8 | 0.8 | 0.4 | 1.2 | 93.3 | | 91.6 | 94.7 | 4.9 | 4.0 | 6.0 | 1.8 | 1.3 | 2.5 |  |
|  |  | 1 | 96.3 | 95.2 | 97.3 | 2.2 | 1.7 | 2.8 | 1.4 | 1.0 | 1.9 | 93.1 | | 91.6 | 94.4 | 4.3 | 3.5 | 5.1 | 2.6 | 2.1 | 3.3 |  |
|  |  | 2 | 91.3 | 89.1 | 93.1 | 5.1 | 4.1 | 6.2 | 3.6 | 2.8 | 4.6 | 85.1 | | 82.4 | 87.6 | 8.4 | 7.1 | 9.8 | 6.5 | 5.3 | 7.8 |  |
|  | 75-84 | 0 | 91.9 | 89.5 | 93.8 | 6.1 | 4.8 | 7.6 | 2.0 | 1.3 | 3.0 | 85.2 | | 82.0 | 88.0 | 10.6 | 8.9 | 12.5 | 4.2 | 3.2 | 5.5 |  |
|  |  | 1 | 91.4 | 89.6 | 93.0 | 5.8 | 4.9 | 6.9 | 2.7 | 2.1 | 3.5 | 85.8 | | 83.4 | 87.9 | 9.6 | 8.4 | 11.0 | 4.6 | 3.7 | 5.6 |  |
|  |  | 2 | 83.1 | 80.6 | 85.4 | 10.2 | 8.9 | 11.5 | 6.7 | 5.7 | 7.9 | 72.9 | | 69.8 | 75.8 | 15.2 | 13.6 | 16.8 | 11.9 | 10.5 | 13.4 |  |
|  | 85-94 | 0 | 81.1 | 73.2 | 87.4 | 12.6 | 8.9 | 17.0 | 6.3 | 3.8 | 9.7 | 69.3 | | 60.0 | 77.4 | 21.5 | 16.6 | 26.8 | 9.2 | 6.0 | 13.3 |  |
|  |  | 1 | 82.7 | 77.2 | 87.3 | 12.8 | 9.9 | 16.1 | 4.5 | 2.8 | 6.7 | 73.9 | | 67.2 | 79.8 | 16.7 | 13.4 | 20.4 | 9.4 | 6.9 | 12.4 |  |
|  |  | 2 | 73.1 | 67.5 | 78.1 | 16.9 | 14.1 | 19.9 | 10.0 | 7.8 | 12.5 | 54.6 | | 47.8 | 61.1 | 27.7 | 24.1 | 31.3 | 17.7 | 14.8 | 20.9 |  |
| Regional | 65-74 | 0 | 89.9 | 87.9 | 91.6 | 9.0 | 7.7 | 10.4 | 1.1 | 0.7 | 1.7 | 78.3 | | 75.7 | 80.7 | 19.8 | 17.9 | 21.7 | 1.9 | 1.3 | 2.6 |  |
|  |  | 1 | 90.5 | 88.7 | 92.1 | 8.0 | 6.8 | 9.2 | 1.5 | 1.1 | 2.1 | 79.7 | | 77.2 | 82.1 | 17.4 | 15.8 | 19.1 | 2.8 | 2.2 | 3.6 |  |
|  |  | 2 | 86.3 | 83.3 | 88.9 | 10.5 | 8.8 | 12.3 | 3.2 | 2.3 | 4.4 | 74.0 | | 70.1 | 77.5 | 20.5 | 18.2 | 22.9 | 5.6 | 4.3 | 7.0 |  |
|  | 75-84 | 0 | 81.8 | 78.6 | 84.7 | 16.0 | 13.9 | 18.3 | 2.1 | 1.4 | 3.1 | 67.3 | | 63.3 | 71.1 | 28.6 | 25.9 | 31.3 | 4.1 | 3.0 | 5.4 |  |
|  |  | 1 | 81.0 | 78.2 | 83.4 | 15.6 | 13.9 | 17.4 | 3.4 | 2.6 | 4.4 | 66.6 | | 63.2 | 69.8 | 27.9 | 25.7 | 30.1 | 5.5 | 4.5 | 6.7 |  |
|  |  | 2 | 74.5 | 71.2 | 77.5 | 20.6 | 18.6 | 22.7 | 4.8 | 3.8 | 6.0 | 58.2 | | 54.3 | 62.0 | 33.8 | 31.3 | 36.2 | 8.0 | 6.7 | 9.5 |  |
|  | 85-94 | 0 | 66.5 | 57.4 | 74.3 | 28.8 | 23.2 | 34.7 | 4.7 | 2.5 | 7.9 | 53.5 | | 43.5 | 62.5 | 39.7 | 33.4 | 45.9 | 6.9 | 4.1 | 10.6 |  |
|  |  | 1 | 65.6 | 58.3 | 72.1 | 28.1 | 23.7 | 32.7 | 6.3 | 4.1 | 9.0 | 48.5 | | 40.2 | 56.3 | 41.2 | 36.2 | 46.2 | 10.3 | 7.5 | 13.6 |  |
|  |  | 2 | 57.3 | 50.3 | 63.8 | 32.9 | 28.8 | 37.1 | 9.8 | 7.3 | 12.6 | 40.2 | | 32.5 | 47.7 | 44.8 | 40.3 | 49.2 | 14.9 | 11.9 | 18.3 |  |
| Distant | 65-74 | 0 | 56.2 | 52.1 | 60.0 | 41.4 | 38.4 | 44.5 | 2.4 | 1.5 | 3.4 | 32.7 | | 28.6 | 36.8 | 63.7 | 60.7 | 66.6 | 3.5 | 2.5 | 4.8 |  |
|  |  | 1 | 54.3 | 50.5 | 58.0 | 43.7 | 40.7 | 46.6 | 2.0 | 1.3 | 3.0 | 30.4 | | 26.5 | 34.2 | 66.6 | 63.7 | 69.3 | 3.1 | 2.2 | 4.2 |  |
|  |  | 2 | 44.2 | 38.5 | 49.7 | 51.0 | 47.0 | 54.8 | 4.8 | 3.3 | 6.7 | 22.1 | | 16.6 | 27.6 | 71.3 | 67.6 | 74.7 | 6.6 | 4.8 | 8.7 |  |
|  | 75-84 | 0 | 38.5 | 33.1 | 43.7 | 58.2 | 54.2 | 61.9 | 3.4 | 2.2 | 5.0 | 18.5 | | 13.8 | 23.3 | 77.8 | 74.3 | 80.9 | 3.7 | 2.4 | 5.4 |  |
|  |  | 1 | 41.0 | 36.9 | 45.1 | 55.8 | 52.7 | 58.7 | 3.2 | 2.3 | 4.4 | 20.2 | | 16.3 | 24.0 | 75.9 | 73.2 | 78.5 | 3.9 | 2.8 | 5.2 |  |
|  |  | 2 | 32.6 | 28.0 | 37.2 | 61.6 | 58.4 | 64.7 | 5.7 | 4.4 | 7.4 | 14.9 | | 10.6 | 19.3 | 78.1 | 75.3 | 80.6 | 7.0 | 5.4 | 8.7 |  |
|  | 85-94 | 0 | 21.9 | 11.4 | 32.3 | 73.5 | 65.6 | 79.8 | 4.6 | 2.0 | 8.8 | 9.3 | | 0.2 | 18.9 | 85.4 | 78.7 | 90.2 | 5.3 | 2.5 | 9.7 |  |
|  |  | 1 | 24.4 | 15.0 | 33.6 | 69.3 | 62.7 | 74.9 | 6.4 | 3.6 | 10.1 | 12.0 | | 3.3 | 21.1 | 80.2 | 74.2 | 84.9 | 7.8 | 4.7 | 11.8 |  |
|  |  | 2 | 21.7 | 14.0 | 29.4 | 71.4 | 66.2 | 76.0 | 6.9 | 4.5 | 10.0 | 7.6 | | 0.7 | 14.6 | 84.3 | 79.9 | 87.9 | 8.1 | 5.5 | 11.4 |  |

## Table S12-3-1. Number of patients and five-year probabilities of dying from cancer, dying from other-causes, and survival by stage, age and comorbidity level: **female colorectal cancer**

| Stage | Age at diagnosis | Comorbidity | N | Survival (%) | | | Cancer deaths (%) | | | Other-cause deaths (%) | | |
| --- | --- | --- | --- | --- | --- | --- | --- | --- | --- | --- | --- | --- |
|  |  |  |  | Estimate | 95%CI. | | Estimate | 95%CI. | | Estimate | 95%CI. | |
| Localized | 65-74 | 0 | 1257 | 86.5 | 83.3 | 89.3 | 9.8 | 8.0 | 11.8 | 3.7 | 2.6 | 5.0 |
|  |  | 1 | 2021 | 85.2 | 82.5 | 87.6 | 9.9 | 8.5 | 11.5 | 4.9 | 3.9 | 6.0 |
|  |  | 2 | 998 | 70.4 | 65.2 | 75.2 | 13.0 | 10.8 | 15.5 | 16.6 | 14.0 | 19.3 |
|  | 75-84 | 0 | 698 | 71.8 | 66.1 | 76.9 | 21.7 | 18.4 | 25.1 | 6.5 | 4.7 | 8.8 |
|  |  | 1 | 1732 | 71.0 | 67.1 | 74.6 | 17.5 | 15.5 | 19.6 | 11.5 | 9.9 | 13.3 |
|  |  | 2 | 1172 | 52.4 | 46.9 | 57.7 | 27.0 | 24.2 | 29.8 | 20.6 | 18.1 | 23.3 |
|  | 85-94 | 0 | 222 | 35.9 | 23.1 | 48.4 | 45.5 | 38.4 | 52.4 | 18.5 | 13.2 | 24.5 |
|  |  | 1 | 458 | 47.5 | 38.1 | 56.4 | 31.0 | 26.3 | 35.8 | 21.5 | 17.3 | 26.1 |
|  |  | 2 | 500 | 27.0 | 17.4 | 36.5 | 37.1 | 32.5 | 41.8 | 35.9 | 31.0 | 40.8 |
| Regional | 65-74 | 0 | 1190 | 60.9 | 56.6 | 64.9 | 35.6 | 32.6 | 38.6 | 3.5 | 2.5 | 4.8 |
|  |  | 1 | 1651 | 63.7 | 60.0 | 67.2 | 32.1 | 29.6 | 34.6 | 4.2 | 3.2 | 5.4 |
|  |  | 2 | 709 | 51.7 | 45.1 | 57.9 | 38.4 | 34.5 | 42.3 | 9.9 | 7.6 | 12.5 |
|  | 75-84 | 0 | 746 | 45.3 | 39.3 | 51.0 | 47.7 | 43.8 | 51.6 | 7.0 | 5.1 | 9.1 |
|  |  | 1 | 1537 | 46.0 | 41.6 | 50.2 | 46.0 | 43.2 | 48.7 | 8.0 | 6.6 | 9.7 |
|  |  | 2 | 1069 | 35.8 | 30.1 | 41.3 | 49.5 | 46.2 | 52.8 | 14.7 | 12.5 | 17.1 |
|  | 85-94 | 0 | 199 | 19.0 | 6.9 | 31.3 | 67.0 | 59.4 | 73.5 | 14.0 | 9.3 | 19.6 |
|  |  | 1 | 466 | 22.4 | 14.0 | 30.9 | 61.0 | 56.0 | 65.7 | 16.5 | 13.1 | 20.4 |
|  |  | 2 | 394 | 16.1 | 6.4 | 25.9 | 58.5 | 53.1 | 63.4 | 25.5 | 21.0 | 30.2 |
| Distant | 65-74 | 0 | 612 | 12.7 | 8.5 | 16.9 | 85.3 | 82.0 | 88.1 | 2.0 | 1.1 | 3.4 |
|  |  | 1 | 803 | 9.4 | 5.6 | 13.2 | 87.1 | 84.4 | 89.4 | 3.5 | 2.4 | 5.0 |
|  |  | 2 | 392 | 8.0 | 2.1 | 14.2 | 85.7 | 81.7 | 89.0 | 6.2 | 4.1 | 8.9 |
|  | 75-84 | 0 | 448 | 8.2 | 3.3 | 13.4 | 87.9 | 84.3 | 90.7 | 3.9 | 2.4 | 6.1 |
|  |  | 1 | 854 | 6.5 | 2.9 | 10.2 | 89.1 | 86.6 | 91.1 | 4.4 | 3.2 | 6.0 |
|  |  | 2 | 600 | 2.0 | -2.5 | 6.6 | 90.4 | 87.7 | 92.5 | 7.6 | 5.7 | 10.0 |
|  | 85-94 | 0 | 160 | 1.2 | -9.7 | 12.8 | 85.5 | 78.9 | 90.2 | 13.3 | 8.3 | 19.5 |
|  |  | 1 | 281 | 4.2 | -2.8 | 11.6 | 88.0 | 83.4 | 91.5 | 7.8 | 5.0 | 11.4 |
|  |  | 2 | 260 | 1.6 | -7.0 | 10.9 | 84.0 | 78.6 | 88.1 | 14.4 | 10.5 | 18.9 |

## Table S12-3-2. One and two-year probabilities of dying from cancer, dying from other-causes, and survival by stage, age and comorbidity level: **female colorectal cancer**

| Stage | Age at diagnosis | Comorbidity | One-year (%) | | | | | | | | | Two-year (%) | | | | | | | | | |
| --- | --- | --- | --- | --- | --- | --- | --- | --- | --- | --- | --- | --- | --- | --- | --- | --- | --- | --- | --- | --- | --- |
|  |  |  | Survival | | | Cancer deaths | | | Other-cause deaths | | | Survival | | | Cancer deaths | | | Other-cause deaths | | | |
|  |  |  | Est. | 95%CI. | | Est. | 95%CI. | | Est. | 95%CI. | | Est. | 95%CI. | | Est. | 95%CI. | | Est. | 95%CI. | |  |
| Localized | 65-74 | 0 | 97.9 | 96.6 | 98.8 | 1.3 | 0.8 | 2.0 | 0.8 | 0.4 | 1.4 | 95.2 | 93.3 | 96.7 | 3.1 | 2.3 | 4.2 | 1.6 | 1.0 | 2.5 |  |
|  |  | 1 | 97.7 | 96.7 | 98.5 | 1.5 | 1.0 | 2.1 | 0.8 | 0.5 | 1.3 | 95.4 | 94.0 | 96.6 | 3.1 | 2.4 | 3.9 | 1.5 | 1.0 | 2.1 |  |
|  |  | 2 | 91.9 | 89.2 | 94.1 | 4.8 | 3.6 | 6.3 | 3.3 | 2.3 | 4.6 | 87.8 | 84.6 | 90.6 | 6.9 | 5.4 | 8.6 | 5.3 | 4.0 | 6.9 |  |
|  | 75-84 | 0 | 93.2 | 90.3 | 95.4 | 5.3 | 3.8 | 7.2 | 1.4 | 0.7 | 2.5 | 88.4 | 84.7 | 91.3 | 9.4 | 7.4 | 11.8 | 2.2 | 1.3 | 3.5 |  |
|  |  | 1 | 94.2 | 92.5 | 95.6 | 4.0 | 3.1 | 5.0 | 1.8 | 1.2 | 2.5 | 88.2 | 85.8 | 90.3 | 7.4 | 6.3 | 8.8 | 4.3 | 3.4 | 5.4 |  |
|  |  | 2 | 86.1 | 83.0 | 88.8 | 8.2 | 6.7 | 9.9 | 5.7 | 4.5 | 7.1 | 76.8 | 73.0 | 80.3 | 14.1 | 12.2 | 16.2 | 9.1 | 7.5 | 10.8 |  |
|  | 85-94 | 0 | 73.3 | 64.3 | 80.8 | 22.1 | 16.9 | 27.8 | 4.5 | 2.3 | 7.8 | 58.4 | 47.9 | 67.9 | 32.8 | 26.6 | 39.1 | 8.8 | 5.5 | 13.0 |  |
|  |  | 1 | 83.4 | 78.0 | 87.9 | 11.6 | 8.8 | 14.7 | 5.0 | 3.3 | 7.3 | 73.8 | 67.3 | 79.6 | 17.6 | 14.3 | 21.3 | 8.5 | 6.2 | 11.4 |  |
|  |  | 2 | 69.5 | 63.0 | 75.4 | 19.0 | 15.7 | 22.6 | 11.4 | 8.8 | 14.4 | 57.0 | 49.5 | 64.0 | 26.4 | 22.6 | 30.4 | 16.6 | 13.4 | 20.0 |  |
| Regional | 65-74 | 0 | 92.9 | 90.8 | 94.6 | 6.5 | 5.2 | 8.0 | 0.6 | 0.3 | 1.2 | 82.2 | 79.1 | 84.9 | 16.2 | 14.1 | 18.4 | 1.6 | 1.0 | 2.5 |  |
|  |  | 1 | 92.9 | 91.1 | 94.4 | 6.3 | 5.2 | 7.6 | 0.8 | 0.4 | 1.3 | 83.1 | 80.5 | 85.3 | 15.3 | 13.6 | 17.1 | 1.6 | 1.1 | 2.3 |  |
|  |  | 2 | 86.4 | 82.7 | 89.6 | 11.2 | 9.0 | 13.6 | 2.4 | 1.5 | 3.7 | 74.7 | 69.9 | 78.9 | 21.9 | 18.9 | 25.1 | 3.5 | 2.3 | 5.0 |  |
|  | 75-84 | 0 | 84.2 | 80.5 | 87.4 | 14.0 | 11.6 | 16.6 | 1.7 | 1.0 | 2.9 | 69.1 | 64.4 | 73.5 | 27.4 | 24.2 | 30.7 | 3.4 | 2.3 | 4.9 |  |
|  |  | 1 | 83.2 | 80.6 | 85.5 | 14.9 | 13.2 | 16.7 | 1.9 | 1.3 | 2.7 | 68.2 | 64.9 | 71.3 | 28.5 | 26.2 | 30.8 | 3.3 | 2.5 | 4.3 |  |
|  |  | 2 | 76.4 | 72.7 | 79.8 | 19.1 | 16.8 | 21.5 | 4.5 | 3.4 | 5.9 | 60.7 | 56.1 | 65.0 | 31.9 | 29.0 | 34.7 | 7.4 | 6.0 | 9.1 |  |
|  | 85-94 | 0 | 58.8 | 48.5 | 67.8 | 36.7 | 30.0 | 43.4 | 4.5 | 2.2 | 8.1 | 39.5 | 28.5 | 49.9 | 52.9 | 45.6 | 59.6 | 7.7 | 4.5 | 11.9 |  |
|  |  | 1 | 63.9 | 57.3 | 70.0 | 29.8 | 25.7 | 34.0 | 6.2 | 4.3 | 8.7 | 42.6 | 35.1 | 49.9 | 46.6 | 42.0 | 51.1 | 10.7 | 8.1 | 13.8 |  |
|  |  | 2 | 55.0 | 47.1 | 62.4 | 35.1 | 30.4 | 39.8 | 9.9 | 7.2 | 13.1 | 38.1 | 29.4 | 46.6 | 46.0 | 41.0 | 50.9 | 15.9 | 12.4 | 19.7 |  |
| Distant | 65-74 | 0 | 56.1 | 51.4 | 60.5 | 43.4 | 39.4 | 47.3 | 0.5 | 0.1 | 1.4 | 32.6 | 27.9 | 37.1 | 66.4 | 62.5 | 70.0 | 1.0 | 0.4 | 2.1 |  |
|  |  | 1 | 52.7 | 48.0 | 57.1 | 44.7 | 41.2 | 48.1 | 2.6 | 1.7 | 3.9 | 29.0 | 24.5 | 33.5 | 67.7 | 64.3 | 70.9 | 3.3 | 2.2 | 4.7 |  |
|  |  | 2 | 49.2 | 42.2 | 55.8 | 47.2 | 42.2 | 52.0 | 3.6 | 2.0 | 5.8 | 23.3 | 16.5 | 30.0 | 71.3 | 66.6 | 75.6 | 5.4 | 3.4 | 7.9 |  |
|  | 75-84 | 0 | 40.6 | 34.5 | 46.4 | 57.2 | 52.5 | 61.6 | 2.2 | 1.1 | 3.9 | 21.8 | 16.1 | 27.3 | 75.3 | 71.0 | 79.1 | 2.9 | 1.6 | 4.8 |  |
|  |  | 1 | 37.7 | 33.2 | 42.1 | 59.5 | 56.1 | 62.7 | 2.8 | 1.9 | 4.1 | 19.6 | 15.4 | 23.7 | 76.6 | 73.5 | 79.3 | 3.9 | 2.7 | 5.3 |  |
|  |  | 2 | 28.7 | 23.0 | 34.2 | 65.8 | 61.9 | 69.5 | 5.5 | 3.9 | 7.5 | 13.0 | 7.9 | 18.1 | 80.9 | 77.5 | 83.8 | 6.2 | 4.4 | 8.3 |  |
|  | 85-94 | 0 | 21.4 | 9.5 | 33.4 | 67.9 | 60.1 | 74.5 | 10.7 | 6.5 | 16.0 | 9.1 | -1.8 | 20.6 | 79.5 | 72.4 | 85.0 | 11.3 | 7.0 | 16.8 |  |
|  |  | 1 | 23.0 | 15.3 | 30.7 | 72.3 | 66.7 | 77.2 | 4.7 | 2.6 | 7.6 | 9.2 | 2.0 | 16.6 | 84.3 | 79.4 | 88.1 | 6.5 | 4.0 | 9.9 |  |
|  |  | 2 | 17.5 | 8.2 | 27.0 | 70.5 | 64.6 | 75.6 | 12.0 | 8.4 | 16.2 | 5.2 | -3.6 | 14.5 | 80.4 | 75.1 | 84.7 | 14.4 | 10.5 | 18.9 |  |

## Table S12-4-1. Number of patients and five-year probabilities of dying from cancer, dying from other-causes, and survival by stage, age and comorbidity level: **male liver cancer**

| Stage | Age at diagnosis | Comorbidity | N | Survival (%) | | | Cancer deaths (%) | | | Other-cause deaths (%) | | |
| --- | --- | --- | --- | --- | --- | --- | --- | --- | --- | --- | --- | --- |
|  |  |  |  | Estimate | 95%CI. | | Estimate | 95%CI. | | Estimate | 95%CI. | |
| Localized | 65-74 | 0 | 2180 | 23.6 | 20.6 | 26.6 | 71.2 | 69.1 | 73.2 | 5.2 | 4.3 | 6.2 |
|  |  | 1 | 5852 | 37.0 | 35.0 | 39.0 | 57.9 | 56.5 | 59.3 | 5.2 | 4.6 | 5.8 |
|  |  | 2 | 3942 | 25.5 | 22.9 | 28.1 | 65.2 | 63.5 | 66.8 | 9.3 | 8.4 | 10.3 |
|  | 75-84 | 0 | 984 | 16.4 | 11.9 | 21.0 | 76.6 | 73.6 | 79.3 | 7.0 | 5.4 | 8.8 |
|  |  | 1 | 3095 | 25.1 | 22.2 | 28.0 | 66.1 | 64.3 | 67.9 | 8.8 | 7.8 | 9.9 |
|  |  | 2 | 3107 | 15.5 | 12.4 | 18.6 | 69.8 | 68.0 | 71.5 | 14.7 | 13.4 | 16.1 |
|  | 85-94 | 0 | 167 | 6.8 | -3.4 | 17.6 | 84.8 | 77.7 | 89.8 | 8.4 | 4.7 | 13.6 |
|  |  | 1 | 465 | 12.2 | 4.9 | 19.7 | 75.7 | 71.2 | 79.7 | 12.1 | 9.2 | 15.4 |
|  |  | 2 | 628 | 5.1 | -1.5 | 11.9 | 75.8 | 72.0 | 79.1 | 19.2 | 16.1 | 22.5 |
| Regional | 65-74 | 0 | 801 | 5.2 | 2.2 | 8.2 | 93.0 | 90.8 | 94.7 | 1.8 | 1.0 | 3.0 |
|  |  | 1 | 1110 | 6.6 | 3.4 | 9.8 | 89.3 | 87.1 | 91.1 | 4.2 | 3.1 | 5.5 |
|  |  | 2 | 646 | 4.7 | 0.8 | 8.9 | 91.3 | 88.5 | 93.4 | 4.0 | 2.7 | 5.8 |
|  | 75-84 | 0 | 356 | 3.7 | -2.4 | 10.1 | 88.7 | 84.8 | 91.7 | 7.6 | 5.1 | 10.8 |
|  |  | 1 | 645 | 5.2 | 1.2 | 9.5 | 90.1 | 87.3 | 92.3 | 4.7 | 3.2 | 6.5 |
|  |  | 2 | 608 | 2.3 | -2.2 | 6.9 | 90.7 | 87.9 | 92.9 | 7.0 | 5.1 | 9.3 |
|  | 85-94 | 0 | 55 | 1.8 | -7.8 | 16.7 | 96.4 | 83.2 | 99.3 | 1.8 | 0.1 | 8.6 |
|  |  | 1 | 137 | 0.9 | -7.2 | 10.2 | 93.3 | 87.1 | 96.5 | 5.9 | 2.7 | 10.7 |
|  |  | 2 | 158 | 1.1 | -9.1 | 12.5 | 88.1 | 81.2 | 92.7 | 10.7 | 6.3 | 16.4 |
| Distant | 65-74 | 0 | 674 | 0.2 | -2.4 | 2.9 | 97.0 | 95.3 | 98.0 | 2.9 | 1.8 | 4.3 |
|  |  | 1 | 750 | 1.8 | -1.1 | 4.9 | 94.7 | 92.8 | 96.1 | 3.5 | 2.4 | 5.0 |
|  |  | 2 | 509 | 1.7 | -2.2 | 5.9 | 93.5 | 90.9 | 95.4 | 4.8 | 3.1 | 6.8 |
|  | 75-84 | 0 | 269 | 1.5 | -3.9 | 7.4 | 93.6 | 89.8 | 96.0 | 4.9 | 2.7 | 7.9 |
|  |  | 1 | 472 | 1.2 | -2.9 | 5.5 | 94.1 | 91.4 | 96.0 | 4.7 | 3.1 | 6.9 |
|  |  | 2 | 488 | 0.8 | -3.6 | 5.5 | 93.0 | 90.2 | 95.0 | 6.2 | 4.3 | 8.6 |
|  | 85-94 | 0 | 63 | 5.0 | -8.2 | 19.8 | 90.1 | 79.0 | 95.5 | 4.8 | 1.2 | 12.6 |
|  |  | 1 | 107 | 1.9 | -7.3 | 12.2 | 93.3 | 86.1 | 96.8 | 4.8 | 1.7 | 10.5 |
|  |  | 2 | 115 | 0.9 | -7.5 | 10.6 | 93.9 | 87.3 | 97.1 | 5.2 | 2.1 | 10.4 |

## Table S12-4-2. One and two-year probabilities of dying from cancer, dying from other-causes, and survival by stage, age and comorbidity level for **male liver cancer**

| Stage | Age at diagnosis | Comorbidity | One-year (%) | | | | | | | | | Two-year (%) | | | | | | | | |
| --- | --- | --- | --- | --- | --- | --- | --- | --- | --- | --- | --- | --- | --- | --- | --- | --- | --- | --- | --- | --- |
|  |  |  | Survival | | | Cancer deaths | | | Other-cause deaths | | | Survival | | | Cancer deaths | | | Other-cause deaths | | |
|  |  |  | Est. | 95%CI. | | Est. | 95%CI. | | Est. | 95%CI. | | Est. | 95%CI. | | Est. | 95%CI. | | Est. | 95%CI. | |
| Localized | 65-74 | 0 | 59.9 | 57.2 | 62.5 | 38.1 | 36.0 | 40.1 | 2.0 | 1.5 | 2.7 | 45.7 | 42.8 | 48.5 | 51.0 | 48.9 | 53.1 | 3.3 | 2.6 | 4.1 |
|  |  | 1 | 77.5 | 76.1 | 78.9 | 20.9 | 19.8 | 21.9 | 1.6 | 1.3 | 1.9 | 64.4 | 62.7 | 66.0 | 33.1 | 31.9 | 34.3 | 2.6 | 2.2 | 3.0 |
|  |  | 2 | 71.2 | 69.3 | 73.1 | 25.9 | 24.6 | 27.3 | 2.9 | 2.4 | 3.4 | 54.8 | 52.5 | 57.0 | 40.3 | 38.8 | 41.9 | 4.9 | 4.3 | 5.6 |
|  | 75-84 | 0 | 50.9 | 46.6 | 55.1 | 45.9 | 42.7 | 49.0 | 3.2 | 2.2 | 4.4 | 37.8 | 33.4 | 42.1 | 58.0 | 54.8 | 61.0 | 4.2 | 3.1 | 5.6 |
|  |  | 1 | 68.6 | 66.4 | 70.7 | 28.7 | 27.1 | 30.3 | 2.7 | 2.2 | 3.4 | 53.0 | 50.5 | 55.5 | 42.7 | 40.9 | 44.4 | 4.3 | 3.6 | 5.1 |
|  |  | 2 | 59.6 | 57.0 | 62.1 | 34.0 | 32.3 | 35.6 | 6.4 | 5.6 | 7.3 | 42.7 | 39.9 | 45.4 | 48.4 | 46.6 | 50.1 | 8.9 | 8.0 | 10.0 |
|  | 85-94 | 0 | 35.0 | 23.8 | 45.7 | 59.5 | 51.6 | 66.6 | 5.4 | 2.7 | 9.6 | 23.4 | 12.8 | 34.0 | 70.5 | 62.9 | 76.9 | 6.0 | 3.1 | 10.4 |
|  |  | 1 | 52.9 | 46.2 | 59.2 | 41.7 | 37.2 | 46.2 | 5.4 | 3.6 | 7.7 | 34.1 | 27.0 | 41.1 | 57.8 | 53.1 | 62.2 | 8.1 | 5.8 | 10.8 |
|  |  | 2 | 47.4 | 41.1 | 53.4 | 43.3 | 39.4 | 47.1 | 9.3 | 7.2 | 11.7 | 26.9 | 20.4 | 33.4 | 59.8 | 55.8 | 63.5 | 13.3 | 10.8 | 16.1 |
| Regional | 65-74 | 0 | 24.2 | 20.4 | 27.9 | 74.7 | 71.5 | 77.6 | 1.1 | 0.6 | 2.1 | 14.0 | 10.6 | 17.3 | 84.8 | 82.1 | 87.1 | 1.3 | 0.6 | 2.2 |
|  |  | 1 | 28.8 | 25.2 | 32.4 | 68.8 | 66.0 | 71.5 | 2.3 | 1.6 | 3.4 | 15.7 | 12.3 | 19.1 | 80.9 | 78.5 | 83.1 | 3.4 | 2.4 | 4.6 |
|  |  | 2 | 26.6 | 21.8 | 31.2 | 71.0 | 67.3 | 74.3 | 2.5 | 1.5 | 3.9 | 13.8 | 9.4 | 18.3 | 82.5 | 79.3 | 85.3 | 3.6 | 2.4 | 5.3 |
|  | 75-84 | 0 | 21.9 | 15.0 | 28.8 | 72.8 | 67.8 | 77.0 | 5.3 | 3.3 | 8.0 | 10.2 | 4.0 | 16.7 | 83.9 | 79.5 | 87.3 | 5.9 | 3.8 | 8.7 |
|  |  | 1 | 25.6 | 20.6 | 30.5 | 70.8 | 67.1 | 74.1 | 3.6 | 2.3 | 5.2 | 12.6 | 8.2 | 17.1 | 83.3 | 80.2 | 86.0 | 4.1 | 2.7 | 5.8 |
|  |  | 2 | 19.5 | 14.5 | 24.4 | 76.3 | 72.7 | 79.4 | 4.3 | 2.9 | 6.1 | 10.6 | 6.0 | 15.3 | 84.2 | 81.1 | 86.9 | 5.2 | 3.6 | 7.1 |
|  | 85-94 | 0 | 18.2 | 2.9 | 33.4 | 80.0 | 66.5 | 88.5 | 1.8 | 0.1 | 8.6 | 7.3 | -4.9 | 21.2 | 90.9 | 78.7 | 96.3 | 1.8 | 0.1 | 8.6 |
|  |  | 1 | 15.9 | 5.1 | 26.9 | 78.2 | 70.3 | 84.2 | 5.9 | 2.7 | 10.7 | 7.8 | -1.9 | 18.2 | 86.3 | 79.1 | 91.2 | 5.9 | 2.7 | 10.7 |
|  |  | 2 | 14.7 | 3.8 | 25.8 | 76.4 | 69.1 | 82.2 | 8.9 | 5.1 | 14.0 | 5.5 | -4.5 | 16.3 | 84.9 | 78.1 | 89.8 | 9.6 | 5.6 | 14.7 |
| Distant | 65-74 | 0 | 9.7 | 6.1 | 13.5 | 87.7 | 85.0 | 90.0 | 2.5 | 1.5 | 3.9 | 2.7 | -0.3 | 5.8 | 94.5 | 92.4 | 96.0 | 2.9 | 1.8 | 4.3 |
|  |  | 1 | 14.1 | 10.2 | 17.9 | 83.1 | 80.3 | 85.6 | 2.8 | 1.8 | 4.2 | 5.4 | 2.1 | 8.7 | 91.4 | 89.2 | 93.2 | 3.2 | 2.1 | 4.7 |
|  |  | 2 | 14.9 | 9.8 | 20.1 | 80.9 | 77.3 | 84.1 | 4.2 | 2.7 | 6.1 | 7.3 | 2.6 | 12.0 | 88.0 | 84.8 | 90.5 | 4.8 | 3.1 | 6.8 |
|  | 75-84 | 0 | 6.6 | 0.6 | 12.9 | 89.3 | 85.0 | 92.5 | 4.1 | 2.2 | 7.0 | 3.5 | -2.2 | 9.5 | 92.0 | 88.0 | 94.7 | 4.5 | 2.5 | 7.4 |
|  |  | 1 | 10.6 | 5.8 | 15.5 | 85.8 | 82.4 | 88.6 | 3.6 | 2.2 | 5.6 | 4.1 | -0.2 | 8.5 | 91.7 | 88.8 | 93.9 | 4.2 | 2.7 | 6.3 |
|  |  | 2 | 8.7 | 3.9 | 13.6 | 86.6 | 83.3 | 89.3 | 4.7 | 3.1 | 6.8 | 3.2 | -1.2 | 7.8 | 91.4 | 88.5 | 93.6 | 5.4 | 3.6 | 7.6 |
|  | 85-94 | 0 | 5.0 | -8.2 | 19.8 | 90.1 | 79.0 | 95.5 | 4.8 | 1.2 | 12.6 | 5.0 | -8.2 | 19.8 | 90.1 | 79.0 | 95.5 | 4.8 | 1.2 | 12.6 |
|  |  | 1 | 5.8 | -3.1 | 15.4 | 91.4 | 83.9 | 95.5 | 2.9 | 0.7 | 7.6 | 1.9 | -7.3 | 12.2 | 93.3 | 86.1 | 96.8 | 4.8 | 1.7 | 10.5 |
|  |  | 2 | 5.2 | -4.3 | 15.5 | 89.6 | 82.3 | 93.9 | 5.2 | 2.1 | 10.4 | 0.9 | -7.5 | 10.6 | 93.9 | 87.3 | 97.1 | 5.2 | 2.1 | 10.4 |

## Table S12-5-1. Number of patients and five-year probabilities of dying from cancer, dying from other-causes, and survival by stage, age and comorbidity level: **female liver cancer**

| Stage | Age at diagnosis | Comorbidity | N | Survival (%) | | | Cancer deaths (%) | | | Other-cause deaths (%) | | |
| --- | --- | --- | --- | --- | --- | --- | --- | --- | --- | --- | --- | --- |
|  |  |  |  | Estimate | 95%CI. | | Estimate | 95%CI. | | Estimate | 95%CI. | |
| Localized | 65-74 | 0 | 764 | 33.0 | 27.9 | 38.0 | 63.6 | 59.8 | 67.2 | 3.3 | 2.2 | 4.9 |
|  |  | 1 | 4032 | 39.7 | 37.3 | 42.0 | 56.1 | 54.4 | 57.8 | 4.2 | 3.6 | 5.0 |
|  |  | 2 | 2395 | 24.0 | 20.7 | 27.3 | 67.4 | 65.2 | 69.4 | 8.6 | 7.5 | 9.9 |
|  | 75-84 | 0 | 404 | 16.9 | 10.2 | 23.7 | 77.7 | 72.9 | 81.7 | 5.4 | 3.4 | 8.1 |
|  |  | 1 | 2581 | 24.1 | 20.8 | 27.3 | 67.4 | 65.3 | 69.4 | 8.5 | 7.3 | 9.7 |
|  |  | 2 | 2060 | 14.8 | 11.3 | 18.5 | 73.4 | 71.3 | 75.5 | 11.7 | 10.3 | 13.3 |
|  | 85-94 | 0 | 108 | 5.7 | -8.8 | 21.4 | 79.0 | 69.5 | 85.8 | 15.3 | 9.1 | 23.1 |
|  |  | 1 | 381 | 6.2 | -1.5 | 14.4 | 81.1 | 76.3 | 85.1 | 12.6 | 9.3 | 16.5 |
|  |  | 2 | 370 | 3.9 | -4.6 | 12.8 | 77.2 | 72.3 | 81.4 | 18.9 | 14.9 | 23.2 |
| Regional | 65-74 | 0 | 241 | 4.8 | -0.7 | 10.8 | 92.7 | 88.1 | 95.6 | 2.5 | 1.0 | 5.1 |
|  |  | 1 | 591 | 6.9 | 2.8 | 11.2 | 90.0 | 86.9 | 92.3 | 3.1 | 1.9 | 4.9 |
|  |  | 2 | 343 | 5.0 | -1.4 | 11.7 | 88.3 | 84.0 | 91.5 | 6.7 | 4.2 | 9.9 |
|  | 75-84 | 0 | 153 | 2.8 | -4.6 | 11.4 | 93.1 | 87.0 | 96.4 | 4.1 | 1.6 | 8.3 |
|  |  | 1 | 532 | 2.3 | -2.2 | 7.1 | 92.1 | 89.1 | 94.2 | 5.6 | 3.8 | 7.9 |
|  |  | 2 | 385 | 4.2 | -1.5 | 10.2 | 88.9 | 85.1 | 91.7 | 6.9 | 4.7 | 9.8 |
|  | 85-94 | 0 | 50 | 4.0 | -12.5 | 23.1 | 88.0 | 74.5 | 94.6 | 8.0 | 2.4 | 17.9 |
|  |  | 1 | 145 | 0.0 | -5.1 | 8.7 | 97.9 | 90.7 | 99.5 | 2.1 | 0.6 | 5.6 |
|  |  | 2 | 132 | 1.9 | -9.0 | 14.7 | 89.2 | 80.7 | 94.1 | 8.9 | 4.6 | 14.9 |
| Distant | 65-74 | 0 | 260 | 0.4 | -2.9 | 4.4 | 98.0 | 95.1 | 99.2 | 1.6 | 0.5 | 3.7 |
|  |  | 1 | 461 | 1.1 | -2.3 | 4.8 | 95.8 | 93.4 | 97.4 | 3.0 | 1.7 | 4.9 |
|  |  | 2 | 244 | 0.9 | -5.2 | 7.6 | 93.2 | 89.0 | 95.8 | 5.9 | 3.4 | 9.4 |
|  | 75-84 | 0 | 156 | 1.4 | -4.7 | 8.6 | 95.4 | 90.2 | 97.8 | 3.2 | 1.2 | 6.9 |
|  |  | 1 | 394 | 0.8 | -3.4 | 5.3 | 94.8 | 92.0 | 96.6 | 4.4 | 2.6 | 6.7 |
|  |  | 2 | 277 | 2.1 | -2.7 | 7.2 | 94.6 | 91.2 | 96.7 | 3.3 | 1.6 | 6.0 |
|  | 85-94 | 0 | 50 | 2.0 | -10.6 | 19.1 | 96.0 | 80.8 | 99.2 | 2.0 | 0.1 | 11.3 |
|  |  | 1 | 90 | 2.2 | -6.3 | 12.3 | 94.4 | 86.8 | 97.7 | 3.3 | 0.9 | 8.6 |
|  |  | 2 | 93 | 2.2 | -7.7 | 13.6 | 92.4 | 84.4 | 96.4 | 5.4 | 2.0 | 11.4 |

## Table S12-5-2. One and two-year probabilities of dying from cancer, dying from other-causes, and survival by stage, age and comorbidity level: **female liver cancer**

| Stage | Age at diagnosis | Comorbidity | One-year (%) | | | | | | | | | Two-year (%) | | | | | | | | |
| --- | --- | --- | --- | --- | --- | --- | --- | --- | --- | --- | --- | --- | --- | --- | --- | --- | --- | --- | --- | --- |
|  |  |  | Survival | | | Cancer deaths | | | Other-cause deaths | | | Survival | | | Cancer deaths | | | Other-cause deaths | | |
|  |  |  | Est. | 95%CI. | | Est. | 95%CI. | | Est. | 95%CI. | | Est. | 95%CI. | | Est. | 95%CI. | | Est. | 95%CI. | |
| Localized | 65-74 | 0 | 67.4 | 63.1 | 71.4 | 31.1 | 27.9 | 34.4 | 1.4 | 0.8 | 2.5 | 54.7 | 50.0 | 59.1 | 43.4 | 39.9 | 47.0 | 1.8 | 1.1 | 3.0 |
|  |  | 1 | 81.7 | 80.1 | 83.1 | 17.2 | 16.0 | 18.4 | 1.2 | 0.9 | 1.5 | 69.2 | 67.3 | 71.0 | 28.7 | 27.3 | 30.2 | 2.1 | 1.6 | 2.5 |
|  |  | 2 | 72.8 | 70.4 | 75.1 | 24.7 | 23.0 | 26.4 | 2.5 | 1.9 | 3.2 | 56.2 | 53.3 | 58.9 | 39.4 | 37.4 | 41.4 | 4.4 | 3.7 | 5.3 |
|  | 75-84 | 0 | 51.4 | 44.7 | 57.5 | 46.2 | 41.2 | 50.9 | 2.5 | 1.3 | 4.4 | 37.7 | 30.8 | 44.2 | 58.8 | 53.8 | 63.5 | 3.5 | 2.0 | 5.7 |
|  |  | 1 | 72.2 | 69.9 | 74.4 | 25.6 | 23.9 | 27.3 | 2.2 | 1.7 | 2.9 | 55.8 | 53.1 | 58.5 | 40.1 | 38.2 | 42.0 | 4.1 | 3.3 | 4.9 |
|  |  | 2 | 62.0 | 59.0 | 64.9 | 33.4 | 31.4 | 35.4 | 4.6 | 3.7 | 5.5 | 43.7 | 40.4 | 47.0 | 49.5 | 47.3 | 51.7 | 6.8 | 5.7 | 7.9 |
|  | 85-94 | 0 | 29.6 | 14.8 | 44.0 | 61.1 | 51.2 | 69.6 | 9.3 | 4.7 | 15.6 | 15.7 | 0.9 | 31.1 | 70.4 | 60.8 | 78.0 | 13.9 | 8.2 | 21.1 |
|  |  | 1 | 50.9 | 43.4 | 57.9 | 43.6 | 38.5 | 48.5 | 5.5 | 3.5 | 8.1 | 32.4 | 24.6 | 39.9 | 60.2 | 55.0 | 65.0 | 7.5 | 5.1 | 10.4 |
|  |  | 2 | 45.7 | 37.5 | 53.4 | 45.4 | 40.3 | 50.4 | 8.9 | 6.3 | 12.1 | 25.1 | 16.4 | 33.7 | 60.4 | 55.1 | 65.2 | 14.6 | 11.2 | 18.4 |
| Regional | 65-74 | 0 | 21.8 | 14.2 | 29.2 | 75.7 | 69.8 | 80.7 | 2.5 | 1.0 | 5.1 | 10.2 | 3.9 | 16.6 | 87.3 | 82.3 | 91.0 | 2.5 | 1.0 | 5.1 |
|  |  | 1 | 30.4 | 25.3 | 35.4 | 67.2 | 63.2 | 70.8 | 2.4 | 1.4 | 3.9 | 17.2 | 12.7 | 21.8 | 80.4 | 76.9 | 83.4 | 2.4 | 1.4 | 3.9 |
|  |  | 2 | 27.4 | 20.1 | 34.5 | 67.7 | 62.5 | 72.3 | 5.0 | 3.0 | 7.6 | 15.4 | 8.8 | 22.0 | 79.7 | 75.0 | 83.6 | 5.0 | 3.0 | 7.6 |
|  | 75-84 | 0 | 15.3 | 6.6 | 24.2 | 82.0 | 74.9 | 87.3 | 2.6 | 0.9 | 6.1 | 8.6 | 0.6 | 17.1 | 88.1 | 81.7 | 92.4 | 3.3 | 1.2 | 7.1 |
|  |  | 1 | 20.9 | 15.7 | 26.0 | 75.8 | 71.9 | 79.2 | 3.4 | 2.1 | 5.2 | 10.3 | 5.5 | 15.3 | 84.9 | 81.6 | 87.7 | 4.7 | 3.2 | 6.8 |
|  |  | 2 | 18.3 | 11.8 | 24.7 | 76.5 | 72.0 | 80.4 | 5.2 | 3.3 | 7.7 | 8.9 | 2.9 | 15.1 | 84.8 | 80.8 | 88.0 | 6.3 | 4.2 | 9.0 |
|  | 85-94 | 0 | 8.0 | -8.4 | 26.0 | 86.0 | 72.5 | 93.2 | 6.0 | 1.5 | 15.2 | 4.0 | -12.5 | 23.1 | 88.0 | 74.5 | 94.6 | 8.0 | 2.4 | 17.9 |
|  |  | 1 | 10.1 | 2.2 | 18.4 | 87.8 | 81.0 | 92.2 | 2.1 | 0.6 | 5.6 | 5.6 | -1.3 | 13.3 | 92.3 | 86.2 | 95.7 | 2.1 | 0.6 | 5.6 |
|  |  | 2 | 15.2 | 4.2 | 26.3 | 78.8 | 70.8 | 84.8 | 6.1 | 2.8 | 11.0 | 8.0 | -2.6 | 19.2 | 84.3 | 76.9 | 89.6 | 7.7 | 3.9 | 13.0 |
| Distant | 65-74 | 0 | 11.3 | 5.6 | 17.1 | 87.2 | 82.4 | 90.7 | 1.6 | 0.5 | 3.7 | 5.3 | 0.6 | 10.3 | 93.1 | 89.2 | 95.6 | 1.6 | 0.5 | 3.7 |
|  |  | 1 | 11.5 | 6.9 | 16.2 | 85.9 | 82.4 | 88.8 | 2.6 | 1.4 | 4.4 | 4.1 | 0.3 | 8.1 | 93.1 | 90.3 | 95.1 | 2.8 | 1.6 | 4.6 |
|  |  | 2 | 13.2 | 5.7 | 20.9 | 81.8 | 76.4 | 86.1 | 4.9 | 2.7 | 8.2 | 6.2 | -0.4 | 13.1 | 88.9 | 84.2 | 92.2 | 4.9 | 2.7 | 8.2 |
|  | 75-84 | 0 | 6.4 | -1.0 | 14.3 | 90.4 | 84.5 | 94.1 | 3.2 | 1.2 | 6.9 | 1.4 | -4.7 | 8.6 | 95.4 | 90.2 | 97.8 | 3.2 | 1.2 | 6.9 |
|  |  | 1 | 11.6 | 6.2 | 17.1 | 84.6 | 80.7 | 87.8 | 3.8 | 2.2 | 6.0 | 3.6 | -1.0 | 8.4 | 92.3 | 89.2 | 94.6 | 4.1 | 2.4 | 6.4 |
|  |  | 2 | 6.5 | 1.1 | 12.1 | 90.6 | 86.5 | 93.5 | 2.9 | 1.4 | 5.4 | 2.1 | -2.7 | 7.2 | 94.6 | 91.2 | 96.7 | 3.3 | 1.6 | 6.0 |
|  | 85-94 | 0 | 6.0 | 1.8 | 19.1 | 94.0 | 80.9 | 98.2 | 0.0 | NA | NA | 2.0 | -10.6 | 19.1 | 96.0 | 80.8 | 99.2 | 2.0 | 0.1 | 11.3 |
|  |  | 1 | 2.2 | -6.3 | 12.3 | 94.4 | 86.8 | 97.7 | 3.3 | 0.9 | 8.6 | 2.2 | -6.3 | 12.3 | 94.4 | 86.8 | 97.7 | 3.3 | 0.9 | 8.6 |
|  |  | 2 | 6.5 | -4.6 | 18.5 | 88.0 | 79.4 | 93.2 | 5.4 | 2.0 | 11.4 | 2.2 | -7.7 | 13.6 | 92.4 | 84.4 | 96.4 | 5.4 | 2.0 | 11.4 |

## Table S12-6-1. Number of patients and five-year probabilities of dying from cancer, dying from other-causes, and survival by stage, age and comorbidity level: **male lung cancer**

| Stage | Age at diagnosis | Comorbidity | N | Survival (%) | | | Cancer deaths (%) | | | Other-cause deaths (%) | | |
| --- | --- | --- | --- | --- | --- | --- | --- | --- | --- | --- | --- | --- |
|  |  |  |  | Estimate | 95%CI. | | Estimate | 95%CI. | | Estimate | 95%CI. | |
| Localized | 65-74 | 0 | 584 | 51.3 | 44.5 | 57.7 | 43.0 | 38.5 | 47.5 | 5.7 | 3.9 | 8.0 |
|  |  | 1 | 834 | 58.7 | 52.8 | 64.2 | 34.2 | 30.5 | 37.9 | 7.1 | 5.3 | 9.3 |
|  |  | 2 | 723 | 42.4 | 35.8 | 48.8 | 47.6 | 43.4 | 51.6 | 10.0 | 7.7 | 12.6 |
|  | 75-84 | 0 | 385 | 33.8 | 25.2 | 42.1 | 56.7 | 51.2 | 61.8 | 9.6 | 6.7 | 13.0 |
|  |  | 1 | 577 | 37.9 | 30.4 | 45.3 | 51.2 | 46.5 | 55.7 | 10.9 | 8.2 | 13.9 |
|  |  | 2 | 1005 | 24.6 | 19.1 | 30.1 | 62.9 | 59.5 | 66.1 | 12.5 | 10.4 | 14.8 |
|  | 85-94 | 0 | 58 | 6.3 | -12.0 | 28.1 | 83.0 | 67.6 | 91.5 | 10.7 | 4.3 | 20.5 |
|  |  | 1 | 103 | 23.6 | 6.9 | 40.8 | 66.8 | 54.6 | 76.5 | 9.5 | 4.6 | 16.6 |
|  |  | 2 | 242 | 9.5 | -2.2 | 21.7 | 67.7 | 60.9 | 73.5 | 22.8 | 17.4 | 28.7 |
| Regional | 65-74 | 0 | 1322 | 11.5 | 8.3 | 14.8 | 84.0 | 81.8 | 86.0 | 4.5 | 3.5 | 5.7 |
|  |  | 1 | 1208 | 13.7 | 10.0 | 17.5 | 81.0 | 78.5 | 83.3 | 5.2 | 4.0 | 6.7 |
|  |  | 2 | 1228 | 11.3 | 7.6 | 15.1 | 81.5 | 79.0 | 83.7 | 7.2 | 5.8 | 8.8 |
|  | 75-84 | 0 | 955 | 4.9 | 1.7 | 8.2 | 90.8 | 88.7 | 92.5 | 4.4 | 3.2 | 5.8 |
|  |  | 1 | 1227 | 7.3 | 3.9 | 10.7 | 86.6 | 84.5 | 88.5 | 6.1 | 4.8 | 7.6 |
|  |  | 2 | 1862 | 4.7 | 2.0 | 7.5 | 88.1 | 86.5 | 89.6 | 7.2 | 6.0 | 8.4 |
|  | 85-94 | 0 | 178 | 0.8 | -7.6 | 10.2 | 90.2 | 84.4 | 93.9 | 9.0 | 5.4 | 13.7 |
|  |  | 1 | 221 | 2.7 | -5.3 | 11.2 | 88.7 | 83.5 | 92.4 | 8.6 | 5.3 | 13.0 |
|  |  | 2 | 470 | 1.4 | -4.0 | 7.1 | 89.4 | 86.1 | 91.9 | 9.2 | 6.8 | 12.1 |
| Distant | 65-74 | 0 | 3463 | 1.9 | 0.5 | 3.4 | 94.4 | 93.5 | 95.1 | 3.7 | 3.1 | 4.4 |
|  |  | 1 | 3302 | 2.8 | 1.2 | 4.4 | 93.5 | 92.5 | 94.3 | 3.7 | 3.1 | 4.4 |
|  |  | 2 | 2494 | 2.5 | 0.8 | 4.4 | 93.6 | 92.5 | 94.5 | 3.9 | 3.2 | 4.7 |
|  | 75-84 | 0 | 2489 | 1.1 | -0.5 | 2.7 | 95.2 | 94.3 | 96.0 | 3.7 | 3.0 | 4.6 |
|  |  | 1 | 3342 | 1.6 | 0.0 | 3.1 | 93.9 | 93.0 | 94.7 | 4.6 | 3.9 | 5.3 |
|  |  | 2 | 4174 | 0.9 | -0.6 | 2.5 | 92.8 | 92.0 | 93.6 | 6.3 | 5.6 | 7.0 |
|  | 85-94 | 0 | 566 | 0.4 | -3.4 | 4.5 | 93.8 | 91.5 | 95.6 | 5.7 | 4.0 | 7.9 |
|  |  | 1 | 865 | 0.5 | -2.8 | 4.0 | 93.0 | 91.0 | 94.5 | 6.6 | 5.0 | 8.3 |
|  |  | 2 | 1330 | 0.7 | -2.0 | 3.6 | 92.4 | 90.8 | 93.7 | 6.9 | 5.6 | 8.3 |

## Table S12-6-2. One and two-year probabilities of dying from cancer, dying from other-causes, and survival by stage, age and comorbidity level: **male lung cancer**

| Stage | Age at diagnosis | Comorbidity | One-year (%) | | | | | | | | | Two-year (%) | | | | | | | | |
| --- | --- | --- | --- | --- | --- | --- | --- | --- | --- | --- | --- | --- | --- | --- | --- | --- | --- | --- | --- | --- |
|  |  |  | Survival | | | Cancer deaths | | | Other-cause deaths | | | Survival | | | Cancer deaths | | | Other-cause deaths | | |
|  |  |  | Est. | 95%CI. | | Est. | 95%CI. | | Est. | 95%CI. | | Est. | 95%CI. | | Est. | 95%CI. | | Est. | 95%CI. | |
| Localized | 65-74 | 0 | 86.4 | 82.3 | 89.8 | 11.5 | 9.1 | 14.2 | 2.1 | 1.1 | 3.5 | 74.7 | 69.5 | 79.2 | 22.2 | 18.9 | 25.7 | 3.1 | 1.9 | 4.8 |
|  |  | 1 | 87.9 | 84.7 | 90.5 | 10.6 | 8.6 | 12.8 | 1.6 | 0.9 | 2.6 | 78.0 | 73.9 | 81.6 | 19.2 | 16.5 | 22.0 | 2.8 | 1.8 | 4.1 |
|  |  | 2 | 83.7 | 79.8 | 87.0 | 14.3 | 11.8 | 16.9 | 2.1 | 1.2 | 3.3 | 70.4 | 65.4 | 74.9 | 25.0 | 21.8 | 28.3 | 4.6 | 3.2 | 6.3 |
|  | 75-84 | 0 | 71.8 | 65.1 | 77.6 | 24.0 | 19.9 | 28.4 | 4.2 | 2.5 | 6.5 | 54.0 | 46.4 | 61.0 | 40.5 | 35.4 | 45.4 | 5.6 | 3.6 | 8.2 |
|  |  | 1 | 77.3 | 72.3 | 81.6 | 19.6 | 16.5 | 22.9 | 3.1 | 1.9 | 4.8 | 67.1 | 61.3 | 72.4 | 27.8 | 24.1 | 31.5 | 5.2 | 3.5 | 7.2 |
|  |  | 2 | 69.9 | 65.8 | 73.6 | 26.3 | 23.6 | 29.1 | 3.8 | 2.7 | 5.1 | 50.4 | 45.6 | 54.9 | 43.2 | 40.1 | 46.3 | 6.4 | 5.0 | 8.0 |
|  | 85-94 | 0 | 51.3 | 31.5 | 67.5 | 45.2 | 31.9 | 57.5 | 3.5 | 0.6 | 11.0 | 23.8 | 2.9 | 44.5 | 65.5 | 51.2 | 76.6 | 10.7 | 4.3 | 20.5 |
|  |  | 1 | 65.0 | 50.6 | 76.7 | 30.1 | 21.5 | 39.1 | 4.9 | 1.8 | 10.3 | 47.4 | 32.2 | 61.0 | 46.6 | 36.6 | 56.1 | 5.9 | 2.4 | 11.7 |
|  |  | 2 | 56.5 | 46.6 | 65.4 | 36.1 | 30.1 | 42.1 | 7.5 | 4.6 | 11.2 | 34.1 | 23.1 | 44.8 | 51.4 | 44.8 | 57.5 | 14.6 | 10.4 | 19.4 |
| Regional | 65-74 | 0 | 52.9 | 49.3 | 56.3 | 44.6 | 41.9 | 47.3 | 2.5 | 1.8 | 3.5 | 30.4 | 26.8 | 33.9 | 66.2 | 63.5 | 68.7 | 3.4 | 2.5 | 4.5 |
|  |  | 1 | 57.2 | 53.4 | 60.8 | 40.1 | 37.3 | 42.8 | 2.7 | 1.9 | 3.8 | 35.8 | 32.0 | 39.5 | 61.0 | 58.2 | 63.7 | 3.2 | 2.3 | 4.3 |
|  |  | 2 | 48.3 | 44.4 | 52.1 | 47.9 | 45.0 | 50.6 | 3.8 | 2.9 | 5.0 | 28.8 | 24.7 | 32.8 | 65.3 | 62.5 | 67.9 | 5.9 | 4.7 | 7.4 |
|  | 75-84 | 0 | 35.8 | 31.6 | 39.8 | 61.7 | 58.5 | 64.7 | 2.5 | 1.7 | 3.7 | 16.6 | 12.9 | 20.4 | 79.9 | 77.2 | 82.3 | 3.5 | 2.5 | 4.8 |
|  |  | 1 | 43.9 | 40.0 | 47.5 | 53.2 | 50.4 | 55.9 | 2.9 | 2.1 | 4.0 | 22.3 | 18.7 | 25.9 | 73.5 | 70.9 | 75.9 | 4.2 | 3.2 | 5.4 |
|  |  | 2 | 35.5 | 32.3 | 38.6 | 60.1 | 57.8 | 62.3 | 4.5 | 3.6 | 5.5 | 17.0 | 14.1 | 20.0 | 77.5 | 75.5 | 79.4 | 5.5 | 4.5 | 6.6 |
|  | 85-94 | 0 | 18.0 | 7.4 | 28.7 | 73.0 | 65.9 | 78.9 | 9.0 | 5.4 | 13.7 | 3.4 | -5.4 | 12.8 | 87.6 | 81.8 | 91.7 | 9.0 | 5.4 | 13.7 |
|  |  | 1 | 31.3 | 21.9 | 40.3 | 64.1 | 57.4 | 70.1 | 4.6 | 2.4 | 8.0 | 10.7 | 2.3 | 19.3 | 82.8 | 76.9 | 87.3 | 6.5 | 3.7 | 10.3 |
|  |  | 2 | 28.6 | 22.1 | 35.0 | 65.4 | 60.9 | 69.5 | 6.0 | 4.1 | 8.4 | 8.4 | 2.6 | 14.5 | 83.1 | 79.4 | 86.2 | 8.5 | 6.2 | 11.2 |
| Distant | 65-74 | 0 | 30.6 | 28.5 | 32.7 | 66.7 | 65.1 | 68.2 | 2.7 | 2.2 | 3.3 | 12.9 | 11.1 | 14.8 | 83.9 | 82.6 | 85.1 | 3.2 | 2.6 | 3.8 |
|  |  | 1 | 33.9 | 31.7 | 36.1 | 63.4 | 61.7 | 65.0 | 2.7 | 2.2 | 3.3 | 15.3 | 13.3 | 17.2 | 81.5 | 80.2 | 82.8 | 3.2 | 2.6 | 3.8 |
|  |  | 2 | 27.2 | 24.7 | 29.7 | 69.9 | 68.1 | 71.6 | 2.9 | 2.3 | 3.6 | 12.0 | 9.8 | 14.1 | 84.7 | 83.2 | 86.0 | 3.4 | 2.7 | 4.2 |
|  | 75-84 | 0 | 21.2 | 18.9 | 23.6 | 75.9 | 74.2 | 77.5 | 2.9 | 2.3 | 3.6 | 7.6 | 5.7 | 9.6 | 89.0 | 87.7 | 90.2 | 3.4 | 2.7 | 4.1 |
|  |  | 1 | 23.8 | 21.7 | 25.9 | 73.2 | 71.7 | 74.7 | 2.9 | 2.4 | 3.6 | 8.4 | 6.6 | 10.2 | 87.6 | 86.4 | 88.6 | 4.0 | 3.4 | 4.7 |
|  |  | 2 | 19.3 | 17.3 | 21.2 | 76.0 | 74.7 | 77.3 | 4.7 | 4.1 | 5.4 | 7.3 | 5.6 | 9.0 | 87.0 | 86.0 | 88.0 | 5.7 | 5.0 | 6.4 |
|  | 85-94 | 0 | 12.7 | 7.8 | 17.7 | 82.2 | 78.8 | 85.1 | 5.1 | 3.5 | 7.2 | 4.0 | -0.2 | 8.4 | 90.6 | 87.9 | 92.8 | 5.3 | 3.7 | 7.4 |
|  |  | 1 | 15.2 | 11.1 | 19.3 | 79.7 | 76.9 | 82.2 | 5.1 | 3.8 | 6.7 | 5.0 | 1.3 | 8.8 | 88.7 | 86.4 | 90.6 | 6.3 | 4.8 | 8.0 |
|  |  | 2 | 14.0 | 10.7 | 17.4 | 80.1 | 77.9 | 82.1 | 5.9 | 4.7 | 7.2 | 5.0 | 2.0 | 8.1 | 88.5 | 86.7 | 90.1 | 6.5 | 5.3 | 7.9 |

## Table S12-7-1. Number of patients and five-year probabilities of dying from cancer, dying from other-causes, and survival by stage, age and comorbidity level: **female lung cancer**

| Stage | Age at diagnosis | Comorbidity | N | Survival (%) | | | Cancer deaths (%) | | | Other-cause deaths (%) | | |
| --- | --- | --- | --- | --- | --- | --- | --- | --- | --- | --- | --- | --- |
|  |  |  |  | Estimate | 95%CI. | | Estimate | 95%CI. | | Estimate | 95%CI. | |
| Localized | 65-74 | 0 | 468 | 76.4 | 69.8 | 81.9 | 21.1 | 16.9 | 25.6 | 2.5 | 1.2 | 4.5 |
|  |  | 1 | 788 | 76.5 | 71.2 | 81.1 | 20.3 | 16.9 | 23.9 | 3.2 | 2.0 | 4.9 |
|  |  | 2 | 430 | 69.5 | 61.7 | 76.4 | 24.7 | 20.1 | 29.6 | 5.7 | 3.5 | 8.7 |
|  | 75-84 | 0 | 156 | 56.7 | 41.7 | 69.3 | 36.4 | 27.7 | 45.1 | 6.9 | 2.9 | 13.2 |
|  |  | 1 | 440 | 59.8 | 51.4 | 67.3 | 35.0 | 29.7 | 40.3 | 5.2 | 3.0 | 8.3 |
|  |  | 2 | 349 | 43.7 | 33.7 | 53.3 | 44.0 | 38.0 | 49.8 | 12.2 | 8.7 | 16.4 |
|  | 85-94 | 0 | 19 | 20.4 | -19.8 | 62.7 | 63.0 | 33.5 | 82.2 | 16.6 | 3.7 | 37.5 |
|  |  | 1 | 64 | 25.7 | 4.5 | 47.7 | 66.0 | 49.3 | 78.4 | 8.3 | 3.0 | 17.1 |
|  |  | 2 | 70 | 5.8 | -24.2 | 40.5 | 68.7 | 47.6 | 82.7 | 25.4 | 11.9 | 41.5 |
| Regional | 65-74 | 0 | 454 | 30.1 | 23.6 | 36.4 | 67.4 | 62.3 | 71.9 | 2.6 | 1.4 | 4.5 |
|  |  | 1 | 638 | 23.9 | 17.9 | 29.8 | 70.7 | 66.5 | 74.5 | 5.4 | 3.7 | 7.6 |
|  |  | 2 | 363 | 18.7 | 10.9 | 26.7 | 74.5 | 68.9 | 79.3 | 6.8 | 4.4 | 9.8 |
|  | 75-84 | 0 | 212 | 13.7 | 5.3 | 22.4 | 81.9 | 75.5 | 86.8 | 4.4 | 2.2 | 7.9 |
|  |  | 1 | 520 | 14.3 | 8.2 | 20.5 | 78.8 | 74.7 | 82.4 | 6.8 | 4.8 | 9.4 |
|  |  | 2 | 418 | 11.5 | 4.6 | 18.6 | 80.7 | 76.0 | 84.5 | 7.8 | 5.4 | 10.9 |
|  | 85-94 | 0 | 72 | 1.4 | -12.2 | 16.9 | 90.1 | 79.8 | 95.3 | 8.5 | 3.2 | 16.9 |
|  |  | 1 | 125 | 2.7 | -7.0 | 13.5 | 90.0 | 83.0 | 94.3 | 7.3 | 3.5 | 12.8 |
|  |  | 2 | 157 | 3.0 | -6.5 | 13.5 | 88.4 | 81.7 | 92.8 | 8.5 | 4.8 | 13.7 |
| Distant | 65-74 | 0 | 1839 | 6.4 | 4.1 | 8.8 | 90.6 | 89.0 | 92.0 | 3.0 | 2.3 | 3.9 |
|  |  | 1 | 2426 | 6.9 | 4.8 | 9.0 | 89.9 | 88.4 | 91.1 | 3.2 | 2.6 | 4.0 |
|  |  | 2 | 1127 | 5.4 | 2.1 | 8.8 | 89.1 | 86.9 | 90.9 | 5.5 | 4.3 | 7.0 |
|  | 75-84 | 0 | 1042 | 3.0 | 0.1 | 6.1 | 92.5 | 90.6 | 94.1 | 4.5 | 3.3 | 5.9 |
|  |  | 1 | 2261 | 3.5 | 1.2 | 5.8 | 90.7 | 89.3 | 91.9 | 5.8 | 4.9 | 6.9 |
|  |  | 2 | 1582 | 2.5 | -0.2 | 5.3 | 90.9 | 89.3 | 92.3 | 6.6 | 5.4 | 7.9 |
|  | 85-94 | 0 | 302 | 1.7 | -4.3 | 8.3 | 91.3 | 87.3 | 94.1 | 7.0 | 4.5 | 10.2 |
|  |  | 1 | 680 | 1.5 | -2.4 | 5.7 | 92.0 | 89.6 | 93.9 | 6.4 | 4.7 | 8.5 |
|  |  | 2 | 592 | 0.3 | -4.3 | 5.1 | 91.5 | 88.8 | 93.5 | 8.2 | 6.1 | 10.7 |

## Table S12-7-2. One and Two -year probabilities of dying from cancer, dying from other-causes, and survival by stage, age and comorbidity level: **female lung cancer**

| Stage | Age at diagnosis | Comorbidity | One-year (%) | | | | | | | | | Two-year (%) | | | | | | | | |
| --- | --- | --- | --- | --- | --- | --- | --- | --- | --- | --- | --- | --- | --- | --- | --- | --- | --- | --- | --- | --- |
|  |  |  | Survival | | | Cancer deaths | | | Other-cause deaths | | | Survival | | | Cancer deaths | | | Other-cause deaths | | |
|  |  |  | Est. | 95%CI. | | Est. | 95%CI. | | Est. | 95%CI. | | Est. | 95%CI. | | Est. | 95%CI. | | Est. | 95%CI. | |
| Localized | 65-74 | 0 | 97.4 | 94.7 | 98.9 | 1.7 | 0.8 | 3.2 | 0.9 | 0.3 | 2.1 | 92.3 | 88.5 | 95.0 | 6.8 | 4.7 | 9.4 | 0.9 | 0.3 | 2.1 |
|  |  | 1 | 97.1 | 95.1 | 98.3 | 2.3 | 1.4 | 3.5 | 0.6 | 0.2 | 1.4 | 93.9 | 91.2 | 95.9 | 4.9 | 3.5 | 6.6 | 1.2 | 0.6 | 2.2 |
|  |  | 2 | 92.8 | 88.9 | 95.4 | 6.3 | 4.2 | 8.8 | 0.9 | 0.3 | 2.2 | 86.7 | 81.6 | 90.7 | 10.9 | 8.1 | 14.1 | 2.4 | 1.2 | 4.3 |
|  | 75-84 | 0 | 90.4 | 85.1 | 94.4 | 9.6 | 5.6 | 14.9 | 0.0 | NA | NA | 79.7 | 69.8 | 86.8 | 18.8 | 13.0 | 25.6 | 1.4 | 0.3 | 4.7 |
|  |  | 1 | 93.0 | 89.3 | 95.4 | 6.6 | 4.5 | 9.2 | 0.5 | 0.1 | 1.5 | 83.0 | 77.8 | 87.2 | 15.3 | 12.1 | 18.9 | 1.7 | 0.7 | 3.3 |
|  |  | 2 | 83.7 | 77.4 | 88.7 | 11.5 | 8.4 | 15.1 | 4.9 | 2.9 | 7.5 | 69.9 | 62.4 | 76.5 | 24.3 | 19.8 | 29.1 | 5.8 | 3.6 | 8.6 |
|  | 85-94 | 0 | 62.3 | 23.8 | 87.0 | 32.4 | 12.7 | 54.0 | 5.3 | 0.3 | 22.1 | 45.3 | 4.6 | 78.0 | 43.7 | 20.3 | 65.1 | 10.9 | 1.6 | 30.4 |
|  |  | 1 | 70.3 | 52.0 | 83.6 | 25.0 | 15.1 | 36.1 | 4.7 | 1.2 | 11.9 | 54.9 | 34.8 | 71.5 | 38.6 | 26.4 | 50.6 | 6.5 | 2.0 | 14.5 |
|  |  | 2 | 69.8 | 51.5 | 83.8 | 21.5 | 12.7 | 31.8 | 8.6 | 3.5 | 16.7 | 49.3 | 28.4 | 67.8 | 37.2 | 25.6 | 48.8 | 13.4 | 6.5 | 22.8 |
| Regional | 65-74 | 0 | 74.1 | 68.7 | 78.7 | 24.8 | 20.9 | 28.9 | 1.1 | 0.4 | 2.4 | 58.8 | 52.6 | 64.4 | 39.2 | 34.6 | 43.7 | 2.0 | 1.0 | 3.7 |
|  |  | 1 | 75.5 | 70.9 | 79.5 | 22.6 | 19.4 | 25.9 | 1.9 | 1.0 | 3.2 | 59.4 | 54.2 | 64.3 | 38.0 | 34.2 | 41.9 | 2.5 | 1.5 | 4.0 |
|  |  | 2 | 63.3 | 56.0 | 69.9 | 32.2 | 27.5 | 37.1 | 4.4 | 2.6 | 6.9 | 49.7 | 41.9 | 56.9 | 45.1 | 39.8 | 50.2 | 5.3 | 3.3 | 7.9 |
|  | 75-84 | 0 | 53.8 | 44.6 | 61.8 | 44.3 | 37.6 | 50.9 | 1.9 | 0.6 | 4.5 | 33.2 | 23.7 | 42.2 | 63.0 | 56.0 | 69.2 | 3.8 | 1.8 | 7.1 |
|  |  | 1 | 60.1 | 54.3 | 65.5 | 36.8 | 32.6 | 40.9 | 3.1 | 1.8 | 4.8 | 38.8 | 32.7 | 44.7 | 57.1 | 52.7 | 61.3 | 4.1 | 2.6 | 6.0 |
|  |  | 2 | 52.6 | 45.8 | 58.9 | 43.8 | 39.0 | 48.5 | 3.6 | 2.1 | 5.7 | 30.9 | 23.9 | 37.7 | 63.3 | 58.4 | 67.8 | 5.8 | 3.8 | 8.3 |
|  | 85-94 | 0 | 24.0 | 7.0 | 40.7 | 69.0 | 56.7 | 78.4 | 7.0 | 2.6 | 14.6 | 11.3 | -3.6 | 27.1 | 81.7 | 70.3 | 89.0 | 7.0 | 2.6 | 14.6 |
|  |  | 1 | 35.6 | 23.0 | 47.4 | 60.4 | 51.1 | 68.4 | 4.0 | 1.5 | 8.6 | 15.4 | 4.1 | 26.9 | 79.0 | 70.6 | 85.2 | 5.6 | 2.5 | 10.7 |
|  |  | 2 | 38.9 | 27.4 | 49.6 | 56.6 | 48.4 | 64.0 | 4.5 | 2.0 | 8.6 | 12.2 | 1.9 | 22.9 | 80.6 | 73.3 | 86.0 | 7.2 | 3.8 | 12.0 |
| Distant | 65-74 | 0 | 54.8 | 51.9 | 57.5 | 43.7 | 41.4 | 45.9 | 1.6 | 1.1 | 2.2 | 29.7 | 26.8 | 32.5 | 67.9 | 65.7 | 70.0 | 2.4 | 1.8 | 3.2 |
|  |  | 1 | 54.9 | 52.4 | 57.3 | 43.8 | 41.8 | 45.8 | 1.3 | 0.9 | 1.8 | 31.2 | 28.7 | 33.7 | 66.4 | 64.5 | 68.3 | 2.3 | 1.8 | 3.0 |
|  |  | 2 | 46.6 | 42.6 | 50.5 | 50.0 | 47.0 | 52.9 | 3.4 | 2.4 | 4.5 | 24.1 | 20.3 | 28.0 | 71.4 | 68.7 | 74.0 | 4.5 | 3.4 | 5.8 |
|  | 75-84 | 0 | 38.3 | 34.3 | 42.3 | 59.0 | 55.9 | 61.9 | 2.7 | 1.8 | 3.8 | 18.4 | 14.7 | 22.1 | 78.1 | 75.4 | 80.5 | 3.5 | 2.5 | 4.8 |
|  |  | 1 | 42.7 | 39.9 | 45.4 | 54.2 | 52.1 | 56.2 | 3.1 | 2.5 | 3.9 | 20.8 | 18.1 | 23.4 | 74.6 | 72.7 | 76.4 | 4.6 | 3.8 | 5.6 |
|  |  | 2 | 32.9 | 29.4 | 36.3 | 62.4 | 60.0 | 64.7 | 4.8 | 3.8 | 5.9 | 16.3 | 13.1 | 19.5 | 78.0 | 75.9 | 80.0 | 5.7 | 4.6 | 6.9 |
|  | 85-94 | 0 | 19.9 | 12.4 | 27.4 | 74.5 | 69.2 | 79.0 | 5.6 | 3.4 | 8.6 | 7.2 | 0.6 | 14.0 | 86.2 | 81.8 | 89.6 | 6.6 | 4.2 | 9.8 |
|  |  | 1 | 26.6 | 21.5 | 31.5 | 69.0 | 65.4 | 72.3 | 4.4 | 3.1 | 6.2 | 10.8 | 6.3 | 15.3 | 83.7 | 80.7 | 86.3 | 5.5 | 3.9 | 7.4 |
|  |  | 2 | 20.7 | 15.4 | 25.9 | 74.2 | 70.5 | 77.6 | 5.1 | 3.5 | 7.0 | 8.2 | 3.4 | 13.1 | 85.4 | 82.2 | 88.0 | 6.5 | 4.7 | 8.6 |

## Table S12-8-1. Number of patients and five-year probabilities of dying from cancer, dying from other-causes, and survival by stage, age and comorbidity level: **male oral cancer**

| Stage | Age at diagnosis | Comorbidity | N | Survival (%) | | | Cancer deaths (%) | | | Other-cause deaths (%) | | |
| --- | --- | --- | --- | --- | --- | --- | --- | --- | --- | --- | --- | --- |
|  |  |  |  | Estimate | 95%CI. | | Estimate | 95%CI. | | Estimate | 95%CI. | |
| Localized | 65-74 | 0 | 839 | 72.7 | 67.4 | 77.4 | 20.4 | 17.5 | 23.5 | 6.9 | 5.1 | 9.0 |
|  |  | 1 | 996 | 66.7 | 61.3 | 71.7 | 21.8 | 19.0 | 24.7 | 11.5 | 9.3 | 14.0 |
|  |  | 2 | 619 | 58.6 | 51.2 | 65.5 | 21.8 | 18.2 | 25.5 | 19.7 | 16.2 | 23.3 |
|  | 75-84 | 0 | 180 | 56.7 | 43.5 | 68.5 | 28.2 | 21.5 | 35.3 | 15.1 | 10.0 | 21.2 |
|  |  | 1 | 339 | 54.3 | 44.1 | 63.7 | 28.8 | 23.6 | 34.2 | 16.9 | 12.6 | 21.8 |
|  |  | 2 | 327 | 38.8 | 27.6 | 49.7 | 34.2 | 28.6 | 39.9 | 27.0 | 21.7 | 32.6 |
|  | 85-94 | 0 | 36 | 38.5 | 6.0 | 68.1 | 39.3 | 22.4 | 55.8 | 22.2 | 9.5 | 38.2 |
|  |  | 1 | 43 | 46.2 | 16.2 | 72.4 | 26.9 | 14.2 | 41.4 | 27.0 | 13.5 | 42.5 |
|  |  | 2 | 72 | 16.3 | -11.0 | 45.0 | 35.3 | 22.2 | 48.6 | 48.4 | 32.8 | 62.4 |
| Regional | 65-74 | 0 | 1461 | 33.4 | 29.1 | 37.6 | 58.8 | 56.0 | 61.5 | 7.8 | 6.4 | 9.4 |
|  |  | 1 | 1298 | 37.8 | 33.0 | 42.6 | 51.9 | 48.9 | 54.8 | 10.3 | 8.6 | 12.3 |
|  |  | 2 | 875 | 25.3 | 19.3 | 31.2 | 60.7 | 57.1 | 64.1 | 14.0 | 11.7 | 16.6 |
|  | 75-84 | 0 | 412 | 20.6 | 12.5 | 28.8 | 67.7 | 62.6 | 72.3 | 11.6 | 8.6 | 15.2 |
|  |  | 1 | 467 | 20.7 | 13.0 | 28.5 | 67.8 | 63.0 | 72.1 | 11.4 | 8.5 | 14.8 |
|  |  | 2 | 611 | 13.5 | 6.4 | 20.9 | 65.8 | 61.8 | 69.6 | 20.6 | 17.4 | 24.1 |
|  | 85-94 | 0 | 77 | 8.6 | -9.0 | 27.4 | 75.5 | 63.9 | 83.8 | 15.9 | 8.7 | 25.1 |
|  |  | 1 | 93 | 6.8 | -12.1 | 27.2 | 74.3 | 62.3 | 82.9 | 18.9 | 10.5 | 29.2 |
|  |  | 2 | 146 | 5.6 | -8.3 | 20.3 | 75.0 | 66.6 | 81.5 | 19.4 | 13.1 | 26.8 |
| Distant | 65-74 | 0 | 107 | 8.8 | -1.7 | 20.2 | 86.5 | 78.1 | 91.9 | 4.7 | 1.7 | 9.9 |
|  |  | 1 | 63 | 9.5 | -8.3 | 29.0 | 80.4 | 67.0 | 88.8 | 10.1 | 4.0 | 19.5 |
|  |  | 2 | 62 | 0.0 | -16.5 | 21.3 | 89.6 | 74.8 | 96.0 | 10.4 | 3.9 | 20.5 |
|  | 75-84 | 0 | 35 | 2.9 | -17.1 | 27.7 | 88.6 | 70.2 | 95.9 | 8.6 | 2.1 | 21.2 |
|  |  | 1 | 48 | 4.8 | -12.5 | 24.6 | 88.7 | 74.0 | 95.3 | 6.5 | 1.5 | 17.1 |
|  |  | 2 | 42 | 6.0 | -12.5 | 28.2 | 86.9 | 70.1 | 94.6 | 7.1 | 1.7 | 17.9 |
|  | 85-94 | 0 | 7 | 14.3 | 1.5 | 80.1 | 85.7 | 19.9 | 98.5 | 0.0 | NA | NA |
|  |  | 1 | 8 | 25.0 | 5.5 | 76.7 | 75.0 | 23.3 | 94.5 | 0.0 | NA | NA |
|  |  | 2 | 16 | 18.8 | -20.1 | 59.5 | 68.8 | 38.7 | 86.3 | 12.5 | 1.8 | 33.9 |

## Table S12-8-2. One and two-year probabilities of dying from cancer, dying from other-causes, and survival by stage, age and comorbidity level: **male oral cancer**

| Stage | Age at diagnosis | Comorbidity | One-year (%) | | | | | | | | | Two-year (%) | | | | | | | | |
| --- | --- | --- | --- | --- | --- | --- | --- | --- | --- | --- | --- | --- | --- | --- | --- | --- | --- | --- | --- | --- |
|  |  |  | Survival | | | Cancer deaths | | | Other-cause deaths | | | Survival | | | Cancer deaths | | | Other-cause deaths | | |
|  |  |  | Est. | 95%CI. | | Est. | 95%CI. | | Est. | 95%CI. | | Est. | 95%CI. | | Est. | 95%CI. | | Est. | 95%CI. | |
| Localized | 65-74 | 0 | 95.0 | 92.6 | 96.7 | 3.9 | 2.8 | 5.4 | 1.1 | 0.5 | 2.0 | 88.9 | 85.6 | 91.6 | 8.9 | 7.1 | 11.0 | 2.2 | 1.4 | 3.4 |
|  |  | 1 | 94.2 | 91.9 | 95.9 | 4.6 | 3.4 | 6.1 | 1.2 | 0.7 | 2.0 | 85.3 | 81.9 | 88.2 | 11.1 | 9.3 | 13.2 | 3.6 | 2.5 | 4.9 |
|  |  | 2 | 91.0 | 87.3 | 93.8 | 5.3 | 3.8 | 7.3 | 3.7 | 2.4 | 5.4 | 82.7 | 77.9 | 86.9 | 9.9 | 7.7 | 12.5 | 7.4 | 5.5 | 9.7 |
|  | 75-84 | 0 | 87.8 | 79.7 | 93.2 | 9.4 | 5.7 | 14.3 | 2.8 | 1.0 | 6.0 | 76.9 | 67.0 | 84.8 | 18.0 | 12.7 | 24.0 | 5.1 | 2.5 | 9.0 |
|  |  | 1 | 85.0 | 78.9 | 89.7 | 11.2 | 8.1 | 14.8 | 3.8 | 2.1 | 6.3 | 74.5 | 66.9 | 81.1 | 17.9 | 14.0 | 22.3 | 7.5 | 5.0 | 10.8 |
|  |  | 2 | 79.5 | 72.5 | 85.4 | 13.8 | 10.3 | 17.7 | 6.7 | 4.4 | 9.8 | 68.6 | 60.3 | 76.1 | 19.9 | 15.7 | 24.5 | 11.4 | 8.2 | 15.2 |
|  | 85-94 | 0 | 77.6 | 52.5 | 92.3 | 16.8 | 6.7 | 30.8 | 5.7 | 1.0 | 16.8 | 62.5 | 34.5 | 83.0 | 28.8 | 14.8 | 44.5 | 8.6 | 2.2 | 21.0 |
|  |  | 1 | 81.4 | 60.4 | 92.8 | 16.3 | 7.1 | 28.8 | 2.3 | 0.2 | 10.7 | 64.5 | 39.0 | 83.7 | 23.6 | 12.1 | 37.2 | 11.9 | 4.3 | 23.8 |
|  |  | 2 | 76.4 | 59.3 | 88.5 | 15.3 | 8.1 | 24.6 | 8.3 | 3.4 | 16.2 | 56.3 | 35.2 | 74.2 | 24.5 | 15.0 | 35.2 | 19.3 | 10.8 | 29.6 |
| Regional | 65-74 | 0 | 67.1 | 63.7 | 70.2 | 29.8 | 27.4 | 32.1 | 3.2 | 2.3 | 4.1 | 50.8 | 47.0 | 54.4 | 44.4 | 41.8 | 47.0 | 4.8 | 3.8 | 6.0 |
|  |  | 1 | 72.1 | 68.7 | 75.2 | 24.9 | 22.6 | 27.3 | 3.0 | 2.2 | 4.0 | 55.9 | 51.9 | 59.8 | 38.6 | 36.0 | 41.3 | 5.4 | 4.3 | 6.8 |
|  |  | 2 | 61.8 | 57.0 | 66.3 | 32.8 | 29.7 | 35.9 | 5.4 | 4.0 | 7.0 | 44.3 | 39.0 | 49.4 | 47.7 | 44.3 | 51.0 | 8.0 | 6.4 | 10.0 |
|  | 75-84 | 0 | 56.4 | 49.3 | 62.9 | 38.7 | 34.0 | 43.4 | 4.9 | 3.1 | 7.3 | 38.6 | 31.0 | 46.0 | 53.6 | 48.6 | 58.4 | 7.7 | 5.4 | 10.6 |
|  |  | 1 | 57.6 | 51.2 | 63.5 | 38.5 | 34.1 | 42.9 | 3.9 | 2.4 | 5.9 | 37.0 | 30.1 | 43.7 | 56.6 | 51.9 | 61.0 | 6.3 | 4.4 | 8.8 |
|  |  | 2 | 49.1 | 42.7 | 55.2 | 41.0 | 37.1 | 44.9 | 9.9 | 7.7 | 12.4 | 30.0 | 23.2 | 36.7 | 55.8 | 51.8 | 59.7 | 14.2 | 11.6 | 17.1 |
|  | 85-94 | 0 | 35.8 | 17.7 | 52.9 | 55.0 | 43.1 | 65.4 | 9.2 | 4.0 | 17.0 | 21.2 | 3.1 | 39.6 | 65.6 | 53.7 | 75.1 | 13.2 | 6.7 | 21.8 |
|  |  | 1 | 48.4 | 32.7 | 62.2 | 46.2 | 35.8 | 56.0 | 5.4 | 2.0 | 11.3 | 25.6 | 9.3 | 41.6 | 64.4 | 53.5 | 73.4 | 10.0 | 4.9 | 17.3 |
|  |  | 2 | 35.6 | 22.2 | 48.4 | 53.5 | 45.1 | 61.2 | 11.0 | 6.6 | 16.6 | 19.0 | 5.8 | 32.4 | 67.1 | 58.8 | 74.2 | 13.8 | 8.8 | 20.0 |
| Distant | 65-74 | 0 | 39.3 | 25.6 | 51.7 | 57.0 | 47.0 | 65.8 | 3.7 | 1.2 | 8.6 | 17.8 | 5.7 | 29.9 | 77.6 | 68.3 | 84.4 | 4.7 | 1.7 | 9.9 |
|  |  | 1 | 46.0 | 27.0 | 62.5 | 49.2 | 36.3 | 60.9 | 4.8 | 1.2 | 12.1 | 24.8 | 5.6 | 43.8 | 67.0 | 53.2 | 77.6 | 8.2 | 2.9 | 16.8 |
|  |  | 2 | 32.3 | 13.3 | 50.1 | 61.3 | 47.9 | 72.2 | 6.5 | 2.0 | 14.5 | 12.9 | -4.0 | 30.7 | 79.0 | 66.4 | 87.4 | 8.1 | 2.9 | 16.6 |
|  | 75-84 | 0 | 22.9 | -1.0 | 46.2 | 71.4 | 52.8 | 83.7 | 5.7 | 1.0 | 17.2 | 2.9 | -17.1 | 27.7 | 88.6 | 70.2 | 95.9 | 8.6 | 2.1 | 21.2 |
|  |  | 1 | 39.6 | 18.2 | 58.3 | 56.3 | 40.9 | 69.0 | 4.2 | 0.7 | 12.7 | 16.7 | -1.1 | 35.0 | 79.2 | 64.3 | 88.4 | 4.2 | 0.7 | 12.7 |
|  |  | 2 | 14.3 | -4.7 | 34.2 | 81.0 | 65.0 | 90.2 | 4.8 | 0.8 | 14.5 | 11.9 | -8.1 | 33.3 | 81.0 | 65.0 | 90.2 | 7.1 | 1.7 | 17.9 |
|  | 85-94 | 0 | 14.3 | 1.5 | 80.1 | 85.7 | 19.9 | 98.5 | 0.0 | NA | NA | 14.3 | 1.5 | 80.1 | 85.7 | 19.9 | 98.5 | 0.0 | NA | NA |
|  |  | 1 | 25.0 | 5.5 | 76.7 | 75.0 | 23.3 | 94.5 | 0.0 | NA | NA | 25.0 | 5.5 | 76.7 | 75.0 | 23.3 | 94.5 | 0.0 | NA | NA |
|  |  | 2 | 18.8 | -20.1 | 59.5 | 68.8 | 38.7 | 86.3 | 12.5 | 1.8 | 33.9 | 18.8 | -20.1 | 59.5 | 68.8 | 38.7 | 86.3 | 12.5 | 1.8 | 33.9 |

## Table S12-9-1. Number pf patients and five-year probabilities of dying from cancer, dying from other-causes, and survival by stage, age and comorbidity level: **female oral cancer**

| Stage | Age at diagnosis | Comorbidity | N | Survival (%) | | | Cancer deaths (%) | | | Other-cause deaths (%) | | |
| --- | --- | --- | --- | --- | --- | --- | --- | --- | --- | --- | --- | --- |
|  |  |  |  | Estimate | 95%CI. | | Estimate | 95%CI. | | Estimate | 95%CI. | |
| Localized | 65-74 | 0 | 135 | 78.1 | 65.8 | 86.9 | 18.6 | 12.0 | 26.3 | 3.4 | 1.1 | 7.9 |
|  |  | 1 | 256 | 72.0 | 61.5 | 80.7 | 18.9 | 13.8 | 24.5 | 9.2 | 5.5 | 14.0 |
|  |  | 2 | 125 | 69.3 | 54.2 | 81.5 | 19.8 | 12.8 | 27.8 | 10.9 | 5.7 | 18.0 |
|  | 75-84 | 0 | 53 | 70.1 | 46.8 | 86.6 | 21.4 | 10.8 | 34.3 | 8.5 | 2.6 | 18.9 |
|  |  | 1 | 159 | 63.0 | 48.3 | 75.6 | 22.5 | 15.8 | 30.0 | 14.5 | 8.7 | 21.7 |
|  |  | 2 | 118 | 53.8 | 36.7 | 69.1 | 24.6 | 16.7 | 33.4 | 21.6 | 14.2 | 29.9 |
|  | 85-94 | 0 | 9 | 62.2 | -2.7 | 96.9 | 26.7 | 2.6 | 61.8 | 11.1 | 0.5 | 40.9 |
|  |  | 1 | 32 | 57.7 | 23.5 | 83.7 | 24.4 | 10.1 | 42.1 | 17.9 | 6.2 | 34.4 |
|  |  | 2 | 32 | 35.5 | -2.6 | 70.0 | 37.1 | 19.7 | 54.5 | 27.5 | 10.3 | 48.1 |
| Regional | 65-74 | 0 | 163 | 48.5 | 34.4 | 61.1 | 43.0 | 34.4 | 51.2 | 8.6 | 4.4 | 14.4 |
|  |  | 1 | 264 | 46.0 | 34.9 | 56.5 | 44.7 | 37.8 | 51.4 | 9.2 | 5.7 | 13.7 |
|  |  | 2 | 141 | 34.6 | 18.6 | 50.0 | 47.7 | 38.7 | 56.2 | 17.7 | 11.4 | 25.2 |
|  | 75-84 | 0 | 95 | 29.3 | 11.3 | 46.6 | 59.5 | 48.1 | 69.2 | 11.2 | 5.4 | 19.4 |
|  |  | 1 | 156 | 22.8 | 8.3 | 37.5 | 59.6 | 50.9 | 67.2 | 17.5 | 11.6 | 24.5 |
|  |  | 2 | 118 | 25.8 | 10.6 | 40.9 | 62.0 | 52.2 | 70.5 | 12.2 | 7.0 | 18.9 |
|  | 85-94 | 0 | 26 | 6.2 | -31.1 | 49.2 | 70.0 | 43.3 | 85.9 | 23.8 | 7.5 | 45.2 |
|  |  | 1 | 42 | 20.7 | -4.4 | 46.1 | 71.0 | 52.0 | 83.6 | 8.3 | 1.9 | 20.8 |
|  |  | 2 | 47 | 9.1 | -12.3 | 32.1 | 79.3 | 64.0 | 88.7 | 11.6 | 3.9 | 23.7 |
| Distant | 65-74 | 0 | 9 | 22.2 | -31.3 | 77.2 | 66.7 | 22.4 | 89.6 | 11.1 | 0.4 | 41.7 |
|  |  | 1 | 19 | 17.8 | -13.5 | 52.7 | 77.0 | 47.0 | 91.3 | 5.3 | 0.3 | 22.2 |
|  |  | 2 | 10 | 26.7 | -25.3 | 79.5 | 63.3 | 20.0 | 87.8 | 10.0 | 0.5 | 37.4 |
|  | 75-84 | 0 | 6 | 33.3 | 7.5 | 87.8 | 66.7 | 12.2 | 92.5 | 0.0 | NA | NA |
|  |  | 1 | 10 | 10.0 | -40.6 | 67.2 | 80.0 | 32.6 | 95.7 | 10.0 | 0.1 | 44.9 |
|  |  | 2 | 10 | 20.0 | -28.4 | 71.8 | 70.0 | 27.8 | 90.5 | 10.0 | 0.4 | 37.9 |
|  | 85-94 | 0 | <5 | 100.0 | 100.0 | 100.0 | 0.0 | NA | NA | 0.0 | NA | NA |
|  |  | 1 | <5 | 25.0 | 2.0 | 98.3 | 75.0 | 1.7 | 98.0 | 0.0 | NA | NA |
|  |  | 2 | <5 | 50.0 | 4.0 | 100.0 | 50.0 | 0.0 | 96.0 | 0.0 | NA | NA |

## Table S12-9-2. One and two-year probabilities of dying from cancer, dying from other-causes, and survival by stage, age and comorbidity level: **female oral cancer**

| Stage | Age at diagnosis | Comorbidity | One-year (%) | | | | | | | | | Two-year (%) | | | | | | | | |
| --- | --- | --- | --- | --- | --- | --- | --- | --- | --- | --- | --- | --- | --- | --- | --- | --- | --- | --- | --- | --- |
|  |  |  | Survival | | | Cancer deaths | | | Other-cause deaths | | | Survival | | | Cancer deaths | | | Other-cause deaths | | |
|  |  |  | Est. | 95%CI. | | Est. | 95%CI. | | Est. | 95%CI. | | Est. | 95%CI. | | Est. | 95%CI. | | Est. | 95%CI. | |
| Localized | 65-74 | 0 | 94.1 | 86.3 | 97.9 | 4.4 | 1.8 | 8.9 | 1.5 | 0.3 | 4.8 | 86.4 | 76.6 | 92.7 | 11.4 | 6.7 | 17.5 | 2.2 | 0.6 | 5.9 |
|  |  | 1 | 93.4 | 88.1 | 96.5 | 5.5 | 3.1 | 8.7 | 1.2 | 0.3 | 3.2 | 86.5 | 79.5 | 91.5 | 11.0 | 7.5 | 15.3 | 2.5 | 1.0 | 5.2 |
|  |  | 2 | 92.0 | 83.2 | 96.7 | 6.4 | 3.0 | 11.6 | 1.6 | 0.3 | 5.2 | 81.5 | 69.7 | 89.8 | 14.2 | 8.7 | 21.2 | 4.3 | 1.6 | 9.1 |
|  | 75-84 | 0 | 94.3 | 79.6 | 99.2 | 3.8 | 0.7 | 11.6 | 1.9 | 0.1 | 8.9 | 82.4 | 63.0 | 93.8 | 11.8 | 4.7 | 22.4 | 5.8 | 1.5 | 14.6 |
|  |  | 1 | 91.2 | 83.2 | 95.9 | 6.3 | 3.2 | 10.9 | 2.5 | 0.8 | 5.9 | 81.5 | 71.5 | 88.9 | 14.6 | 9.5 | 20.8 | 3.8 | 1.6 | 7.7 |
|  |  | 2 | 88.1 | 77.6 | 94.8 | 6.8 | 3.2 | 12.3 | 5.1 | 2.1 | 10.1 | 73.1 | 59.2 | 84.1 | 14.7 | 9.0 | 21.8 | 12.2 | 7.0 | 18.9 |
|  | 85-94 | 0 | 88.9 | 59.4 | 99.5 | 11.1 | 0.5 | 40.6 | 0.0 | NA | NA | 77.8 | 18.5 | 99.1 | 11.1 | 0.5 | 40.6 | 11.1 | 0.5 | 40.9 |
|  |  | 1 | 81.3 | 55.1 | 95.1 | 12.5 | 3.9 | 26.5 | 6.3 | 1.1 | 18.4 | 67.5 | 37.1 | 88.3 | 19.2 | 7.6 | 34.7 | 13.4 | 4.1 | 28.2 |
|  |  | 2 | 81.3 | 66.0 | 92.5 | 18.8 | 7.5 | 34.0 | 0.0 | NA | NA | 60.0 | 29.5 | 81.9 | 33.1 | 16.9 | 50.2 | 6.9 | 1.2 | 20.3 |
| Regional | 65-74 | 0 | 79.8 | 70.4 | 86.6 | 18.4 | 12.9 | 24.7 | 1.8 | 0.5 | 4.9 | 66.9 | 56.1 | 75.8 | 30.0 | 23.0 | 37.2 | 3.1 | 1.2 | 6.7 |
|  |  | 1 | 77.6 | 70.3 | 83.4 | 20.5 | 15.9 | 25.6 | 1.9 | 0.7 | 4.1 | 62.8 | 53.6 | 70.8 | 31.7 | 26.0 | 37.4 | 5.6 | 3.2 | 8.9 |
|  |  | 2 | 64.5 | 51.6 | 75.6 | 26.2 | 19.3 | 33.7 | 9.2 | 5.2 | 14.7 | 50.1 | 35.9 | 63.0 | 37.6 | 29.5 | 45.6 | 12.3 | 7.5 | 18.4 |
|  | 75-84 | 0 | 66.3 | 51.8 | 77.6 | 30.6 | 21.6 | 40.0 | 3.2 | 0.8 | 8.2 | 47.5 | 31.7 | 61.4 | 47.1 | 36.6 | 56.9 | 5.4 | 2.0 | 11.4 |
|  |  | 1 | 58.1 | 46.1 | 68.6 | 36.1 | 28.6 | 43.6 | 5.8 | 2.8 | 10.2 | 40.3 | 27.0 | 52.8 | 49.0 | 40.8 | 56.7 | 10.7 | 6.4 | 16.3 |
|  |  | 2 | 46.6 | 32.0 | 59.9 | 44.9 | 35.8 | 53.6 | 8.5 | 4.3 | 14.4 | 37.2 | 22.4 | 51.2 | 52.6 | 43.2 | 61.2 | 10.2 | 5.6 | 16.5 |
|  | 85-94 | 0 | 30.8 | -1.4 | 61.0 | 57.7 | 36.2 | 74.3 | 11.5 | 2.8 | 27.1 | 30.8 | -1.4 | 61.0 | 57.7 | 36.2 | 74.3 | 11.5 | 2.8 | 27.1 |
|  |  | 1 | 40.5 | 17.2 | 60.8 | 54.8 | 38.4 | 68.5 | 4.8 | 0.8 | 14.4 | 28.3 | 5.9 | 50.1 | 66.9 | 49.0 | 79.7 | 4.8 | 0.8 | 14.4 |
|  |  | 2 | 19.1 | -1.7 | 40.3 | 72.3 | 57.0 | 83.0 | 8.5 | 2.7 | 18.7 | 12.2 | -7.4 | 33.3 | 79.3 | 64.0 | 88.7 | 8.5 | 2.7 | 18.7 |
| Distant | 65-74 | 0 | 33.3 | -24.0 | 82.6 | 55.6 | 17.0 | 82.3 | 11.1 | 0.4 | 41.7 | 22.2 | -31.3 | 77.2 | 66.7 | 22.4 | 89.6 | 11.1 | 0.4 | 41.7 |
|  |  | 1 | 41.4 | 4.6 | 71.7 | 53.3 | 28.0 | 73.3 | 5.3 | 0.3 | 22.2 | 29.6 | -5.2 | 62.4 | 65.1 | 37.3 | 83.0 | 5.3 | 0.3 | 22.2 |
|  |  | 2 | 40.0 | -14.4 | 83.6 | 50.0 | 16.0 | 77.0 | 10.0 | 0.5 | 37.4 | 26.7 | -25.3 | 79.5 | 63.3 | 20.0 | 87.8 | 10.0 | 0.5 | 37.4 |
|  | 75-84 | 0 | 33.3 | 7.5 | 87.8 | 66.7 | 12.2 | 92.5 | 0.0 | NA | NA | 33.3 | 7.5 | 87.8 | 66.7 | 12.2 | 92.5 | 0.0 | NA | NA |
|  |  | 1 | 50.0 | 23.2 | 83.7 | 50.0 | 16.3 | 76.8 | 0.0 | NA | NA | 30.0 | 9.6 | 71.8 | 70.0 | 28.2 | 90.4 | 0.0 | NA | NA |
|  |  | 2 | 20.0 | -28.4 | 71.8 | 70.0 | 27.8 | 90.5 | 10.0 | 0.4 | 37.9 | 20.0 | -28.4 | 71.8 | 70.0 | 27.8 | 90.5 | 10.0 | 0.4 | 37.9 |
|  | 85-94 | 0 | 100.0 | 100.0 | 100.0 | 0.0 | NA | NA | 0.0 | NA | NA | 100.0 | 100.0 | 100.0 | 0.0 | NA | NA | 0.0 | NA | NA |
|  |  | 1 | 50.0 | 11.9 | 97.7 | 50.0 | 2.3 | 88.1 | 0.0 | NA | NA | 25.0 | 2.0 | 98.3 | 75.0 | 1.7 | 98.0 | 0.0 | NA | NA |
|  |  | 2 | 50.0 | 4.0 | 100.0 | 50.0 | 0.0 | 96.0 | 0.0 | NA | NA | 50.0 | 4.0 | 100.0 | 50.0 | 0.0 | 96.0 | 0.0 | NA | NA |

## Table S13-1. Number of patients and probabilities of dying from cancer, dying from other-causes, and survival among patients who were diagnosed with distant lung cancer at ages 30—94 in 2004—2014 by subtypes and comorbidity level

| Subtype | Comorbidity | N | One-year | | | | | | | | |
| --- | --- | --- | --- | --- | --- | --- | --- | --- | --- | --- | --- |
|  |  |  | Survival (%) | | | Cancer deaths (%) | | | Other-cause deaths (%) | | |
|  |  |  | Estimate | 95%CI | | Estimate | 95%CI | | Estimate | 95%%CI | |
| SCC | 0 | 2907 | 30.5 | 28.2 | 32.8 | 66.7 | 64.9 | 68.4 | 2.8 | 2.2 | 3.4 |
|  | 1 | 2285 | 25.9 | 23.4 | 28.3 | 71.6 | 69.7 | 73.4 | 2.5 | 1.9 | 3.2 |
|  | 2 | 2501 | 22.6 | 20.0 | 25.1 | 73.3 | 71.5 | 75.0 | 4.1 | 3.4 | 5.0 |
| ADC | 0 | 15277 | 53.8 | 52.8 | 54.7 | 44.8 | 44.0 | 45.6 | 1.4 | 1.2 | 1.6 |
|  | 1 | 11655 | 50.9 | 49.8 | 52.1 | 47.1 | 46.2 | 48.0 | 2.0 | 1.7 | 2.2 |
|  | 2 | 7001 | 37.2 | 35.6 | 38.8 | 59.5 | 58.4 | 60.6 | 3.3 | 2.9 | 3.7 |
| SCLC | 0 | 403 | 35.0 | 28.7 | 41.0 | 63.0 | 58.1 | 67.5 | 2.0 | 0.9 | 3.7 |
|  | 1 | 272 | 29.8 | 21.8 | 37.5 | 66.5 | 60.6 | 71.8 | 3.7 | 1.9 | 6.4 |
|  | 2 | 203 | 26.1 | 17.3 | 34.6 | 70.9 | 64.2 | 76.7 | 3.0 | 1.2 | 6.0 |
| Subtype | Comorbidity | N | Two-year | | | | | | | | |
| SCC | 0 | 2907 | 12.3 | 10.4 | 14.3 | 84.6 | 83.2 | 85.8 | 3.1 | 2.5 | 3.8 |
|  | 1 | 2285 | 9.2 | 7.1 | 11.4 | 87.2 | 85.8 | 88.5 | 3.6 | 2.8 | 4.4 |
|  | 2 | 2501 | 8.5 | 6.3 | 10.6 | 87.1 | 85.7 | 88.3 | 4.5 | 3.7 | 5.3 |
| ADC | 0 | 15277 | 28.8 | 27.8 | 29.7 | 69.3 | 68.5 | 70.0 | 1.9 | 1.7 | 2.2 |
|  | 1 | 11655 | 27.4 | 26.2 | 28.5 | 69.7 | 68.8 | 70.5 | 2.9 | 2.6 | 3.3 |
|  | 2 | 7001 | 18.9 | 17.4 | 20.4 | 76.9 | 75.8 | 77.8 | 4.2 | 3.8 | 4.7 |
| SCLC | 0 | 403 | 17.5 | 12.0 | 22.9 | 80.3 | 75.9 | 83.9 | 2.3 | 1.1 | 4.1 |
|  | 1 | 272 | 12.9 | 6.1 | 19.9 | 83.1 | 78.0 | 87.1 | 4.0 | 2.1 | 6.9 |
|  | 2 | 203 | 11.1 | 3.9 | 18.6 | 85.9 | 80.2 | 90.1 | 3.0 | 1.2 | 6.0 |
| Subtype | Comorbidity | N | Five-year | | | | | | | | |
| SCC | 0 | 2907 | 3.2 | 1.6 | 4.8 | 93.4 | 92.4 | 94.3 | 3.4 | 2.8 | 4.1 |
|  | 1 | 2285 | 2.1 | 0.3 | 4.0 | 94.0 | 92.9 | 95.0 | 3.9 | 3.1 | 4.7 |
|  | 2 | 2501 | 0.9 | -0.9 | 2.8 | 93.9 | 92.9 | 94.8 | 5.2 | 4.3 | 6.1 |
| ADC | 0 | 15277 | 5.1 | 4.3 | 5.8 | 92.5 | 92.0 | 92.9 | 2.5 | 2.2 | 2.7 |
|  | 1 | 11655 | 5.2 | 4.2 | 6.1 | 91.0 | 90.4 | 91.6 | 3.8 | 3.5 | 4.2 |
|  | 2 | 7001 | 3.6 | 2.4 | 4.9 | 91.3 | 90.6 | 92.0 | 5.1 | 4.5 | 5.6 |
| SCLC | 0 | 403 | 4.0 | -0.2 | 8.7 | 93.4 | 90.0 | 95.7 | 2.5 | 1.3 | 4.4 |
|  | 1 | 272 | 1.2 | -4.5 | 7.5 | 93.7 | 89.7 | 96.2 | 5.1 | 2.8 | 8.3 |
|  | 2 | 203 | 2.8 | -3.0 | 9.4 | 94.3 | 89.3 | 97.0 | 3.0 | 1.2 | 6.0 |

## Table S13-2. Number of patients and probabilities of dying from cancer, dying from other-causes, and survival among patients who were diagnosed with distant lung ADC at ages 30—94 by comorbidity level for period 2004—2010 and 2011—2014

| Period | Comorbidity | N | One-year | | | | | | | | |
| --- | --- | --- | --- | --- | --- | --- | --- | --- | --- | --- | --- |
|  |  |  | Survival (%) | | | Cancer deaths (%) | | | Other-cause deaths (%) | | |
|  |  |  | Estimate | 95%CI. | | Estimate | 95%CI. | | Estimate | 95%CI. | |
| 2004-2010 | 0 | 7994 | 50.5 | 49.1 | 51.8 | 48.1 | 47.0 | 49.2 | 1.5 | 1.2 | 1.7 |
|  | 1 | 5730 | 47.8 | 46.2 | 49.5 | 50.2 | 48.9 | 51.5 | 1.9 | 1.6 | 2.3 |
|  | 2 | 3481 | 34.6 | 32.4 | 36.8 | 62.4 | 60.8 | 64.0 | 3.0 | 2.5 | 3.6 |
| 2011-2014 | 0 | 7283 | 57.3 | 55.9 | 58.7 | 41.3 | 40.1 | 42.4 | 1.4 | 1.1 | 1.7 |
|  | 1 | 5925 | 54.0 | 52.3 | 55.6 | 44.1 | 42.8 | 45.3 | 2.0 | 1.7 | 2.4 |
|  | 2 | 3520 | 39.8 | 37.5 | 42.0 | 56.7 | 55.0 | 58.3 | 3.6 | 3.0 | 4.2 |
| Period | Comorbidity | N | Two-year | | | | | | | | |
| 2004-2010 | 0 | 7994 | 27.2 | 25.9 | 28.5 | 70.8 | 69.8 | 71.8 | 2.0 | 1.7 | 2.3 |
|  | 1 | 5730 | 26.0 | 24.4 | 27.6 | 71.3 | 70.1 | 72.5 | 2.7 | 2.3 | 3.1 |
|  | 2 | 3481 | 17.5 | 15.5 | 19.5 | 78.7 | 77.3 | 80.0 | 3.8 | 3.2 | 4.4 |
| 2011-2014 | 0 | 7283 | 30.4 | 29.0 | 31.9 | 67.7 | 66.5 | 68.8 | 1.9 | 1.6 | 2.2 |
|  | 1 | 5925 | 28.6 | 27.0 | 30.3 | 68.1 | 66.9 | 69.3 | 3.2 | 2.8 | 3.7 |
|  | 2 | 3520 | 20.2 | 18.1 | 22.4 | 75.1 | 73.6 | 76.5 | 4.7 | 4.0 | 5.4 |
| Period | Comorbidity | N | Five-year | | | | | | | | |
| 2004-2010 | 0 | 7994 | 5.6 | 4.7 | 6.5 | 92.0 | 91.4 | 92.6 | 2.4 | 2.1 | 2.8 |
|  | 1 | 5730 | 5.9 | 4.7 | 7.1 | 90.7 | 89.9 | 91.4 | 3.4 | 2.9 | 3.9 |
|  | 2 | 3481 | 4.3 | 2.7 | 6.0 | 91.2 | 90.2 | 92.1 | 4.4 | 3.8 | 5.2 |
| 2011-2014 | 0 | 7283 | 2.6 | 1.4 | 3.9 | 94.9 | 94.0 | 95.7 | 2.5 | 2.1 | 2.9 |
|  | 1 | 5925 | 2.4 | 0.8 | 4.2 | 93.2 | 92.0 | 94.2 | 4.3 | 3.8 | 4.9 |
|  | 2 | 3520 | 1.2 | -0.8 | 3.2 | 93.1 | 91.8 | 94.2 | 5.7 | 5.0 | 6.6 |

## Table S14-1. Number of patients and **one**-year probabilities of dying from cancer, dying from other-causes, and survival among **males** who were diagnosed with squamous cell carcinoma lung cancer **(SCC)** by stage, age and comorbidity level (n>100)

| Stage | Age | Comorbidity | N | Survival (%) | | | Cancer deaths (%) | | | Other-Cause Deaths (%) | | |
| --- | --- | --- | --- | --- | --- | --- | --- | --- | --- | --- | --- | --- |
|  |  |  |  | Estimate | 95%CI. | | Estimate | 95%CI. | | Estimate | 95%CI. | |
| Localized | 65-74 | 0 | 156 | 84.6 | 75.4 | 91.0 | 12.8 | 8.1 | 18.6 | 2.6 | 0.8 | 6.0 |
|  |  | 1 | 226 | 83.6 | 76.0 | 89.3 | 13.7 | 9.6 | 18.6 | 2.7 | 1.1 | 5.4 |
|  |  | 2 | 303 | 77.2 | 70.2 | 82.9 | 20.2 | 15.8 | 24.9 | 2.6 | 1.2 | 4.9 |
|  | 75-84 | 0 | 135 | 70.4 | 58.2 | 80.2 | 24.5 | 17.6 | 32.0 | 5.2 | 2.3 | 9.9 |
|  |  | 1 | 188 | 71.8 | 61.8 | 80.0 | 23.4 | 17.6 | 29.7 | 4.8 | 2.4 | 8.5 |
|  |  | 2 | 387 | 68.0 | 61.1 | 74.0 | 27.9 | 23.5 | 32.4 | 4.1 | 2.5 | 6.5 |
|  | 85-94 | 0 | 0 | 0.0 | 0.0 | 0.0 | 0.0 | 0.0 | 0.0 | 0.0 | 0.0 | 0.0 |
|  |  | 1 | 0 | 0.0 | 0.0 | 0.0 | 0.0 | 0.0 | 0.0 | 0.0 | 0.0 | 0.0 |
|  |  | 2 | 0 | 0.0 | 0.0 | 0.0 | 0.0 | 0.0 | 0.0 | 0.0 | 0.0 | 0.0 |
| Regional | 65-74 | 0 | 567 | 50.0 | 44.4 | 55.2 | 47.5 | 43.3 | 51.6 | 2.5 | 1.4 | 4.0 |
|  |  | 1 | 494 | 52.7 | 46.7 | 58.3 | 44.8 | 40.4 | 49.2 | 2.4 | 1.3 | 4.1 |
|  |  | 2 | 581 | 50.5 | 44.8 | 55.8 | 46.4 | 42.3 | 50.4 | 3.1 | 1.9 | 4.8 |
|  | 75-84 | 0 | 410 | 36.0 | 29.7 | 42.0 | 61.8 | 56.9 | 66.3 | 2.2 | 1.1 | 4.0 |
|  |  | 1 | 490 | 39.3 | 33.2 | 45.1 | 57.4 | 52.9 | 61.7 | 3.3 | 2.0 | 5.1 |
|  |  | 2 | 855 | 35.7 | 31.0 | 40.4 | 59.5 | 56.1 | 62.7 | 4.8 | 3.5 | 6.4 |
|  | 85-94 | 0 | 0 | 0.0 | 0.0 | 0.0 | 0.0 | 0.0 | 0.0 | 0.0 | 0.0 | 0.0 |
|  |  | 1 | 0 | 0.0 | 0.0 | 0.0 | 0.0 | 0.0 | 0.0 | 0.0 | 0.0 | 0.0 |
|  |  | 2 | 207 | 29.5 | 20.1 | 38.6 | 66.2 | 59.3 | 72.2 | 4.3 | 2.1 | 7.8 |
| Distant | 65-74 | 0 | 707 | 24.2 | 19.6 | 28.8 | 72.5 | 69.1 | 75.7 | 3.3 | 2.1 | 4.8 |
|  |  | 1 | 611 | 24.9 | 20.1 | 29.5 | 73.0 | 69.3 | 76.4 | 2.1 | 1.2 | 3.5 |
|  |  | 2 | 644 | 26.9 | 22.0 | 31.7 | 70.1 | 66.4 | 73.5 | 3.0 | 1.8 | 4.5 |
|  | 75-84 | 0 | 606 | 18.7 | 14.0 | 23.5 | 77.8 | 74.3 | 80.9 | 3.5 | 2.2 | 5.2 |
|  |  | 1 | 666 | 20.2 | 15.8 | 24.5 | 77.4 | 74.1 | 80.4 | 2.4 | 1.4 | 3.8 |
|  |  | 2 | 1036 | 18.2 | 14.4 | 22.0 | 77.3 | 74.6 | 79.7 | 4.5 | 3.3 | 5.9 |
|  | 85-94 | 0 | 115 | 11.2 | 1.1 | 21.7 | 85.3 | 77.1 | 90.7 | 3.6 | 1.2 | 8.3 |
|  |  | 1 | 178 | 9.9 | 1.5 | 18.7 | 85.0 | 78.8 | 89.5 | 5.1 | 2.5 | 9.0 |
|  |  | 2 | 280 | 11.1 | 3.7 | 18.6 | 81.8 | 76.8 | 85.8 | 7.1 | 4.5 | 10.5 |

## Table S14-2. Number of patients and **two**-year probabilities of dying from cancer, dying from other-causes, and survival among **males** who were diagnosed with squamous cell carcinoma lung cancer **(SCC)** by stage, age and comorbidity level (n>100)

| Stage | Age | Comorbidity | N | Survival (%) | | | Cancer deaths (%) | | | Other-Cause Deaths (%) | | |
| --- | --- | --- | --- | --- | --- | --- | --- | --- | --- | --- | --- | --- |
|  |  |  |  | Estimate | 95%CI. | | Estimate | 95%CI. | | Estimate | 95%CI. | |
| Localized | 65-74 | 0 | 156 | 68.3 | 57.2 | 77.3 | 28.5 | 21.5 | 35.9 | 3.2 | 1.2 | 6.9 |
|  |  | 1 | 226 | 72.4 | 63.3 | 79.9 | 23.1 | 17.8 | 28.9 | 4.5 | 2.3 | 7.8 |
|  |  | 2 | 303 | 65.6 | 57.6 | 72.6 | 30.3 | 25.2 | 35.6 | 4.0 | 2.2 | 6.7 |
|  | 75-84 | 0 | 135 | 48.0 | 34.4 | 60.3 | 44.5 | 35.8 | 52.7 | 7.5 | 3.8 | 12.9 |
|  |  | 1 | 188 | 57.3 | 45.9 | 67.4 | 35.1 | 28.3 | 42.0 | 7.6 | 4.4 | 12.0 |
|  |  | 2 | 387 | 47.6 | 39.9 | 54.9 | 45.9 | 40.8 | 50.8 | 6.5 | 4.3 | 9.3 |
|  | 85-94 | 0 | 0 | 0.0 | 0.0 | 0.0 | 0.0 | 0.0 | 0.0 | 0.0 | 0.0 | 0.0 |
|  |  | 1 | 0 | 0.0 | 0.0 | 0.0 | 0.0 | 0.0 | 0.0 | 0.0 | 0.0 | 0.0 |
|  |  | 2 | 0 | 0.0 | 0.0 | 0.0 | 0.0 | 0.0 | 0.0 | 0.0 | 0.0 | 0.0 |
| Regional | 65-74 | 0 | 567 | 26.6 | 21.2 | 31.8 | 70.0 | 66.1 | 73.7 | 3.4 | 2.1 | 5.1 |
|  |  | 1 | 494 | 30.1 | 24.4 | 35.6 | 67.5 | 63.1 | 71.5 | 2.4 | 1.3 | 4.1 |
|  |  | 2 | 581 | 28.7 | 23.0 | 34.3 | 66.4 | 62.3 | 70.1 | 4.9 | 3.3 | 6.9 |
|  | 75-84 | 0 | 410 | 14.7 | 9.3 | 20.1 | 82.4 | 78.3 | 85.8 | 2.9 | 1.6 | 4.9 |
|  |  | 1 | 490 | 18.0 | 12.6 | 23.5 | 78.1 | 74.1 | 81.5 | 3.9 | 2.4 | 5.9 |
|  |  | 2 | 855 | 14.8 | 10.5 | 19.0 | 79.7 | 76.8 | 82.3 | 5.5 | 4.1 | 7.2 |
|  | 85-94 | 0 | 0 | 0.0 | 0.0 | 0.0 | 0.0 | 0.0 | 0.0 | 0.0 | 0.0 | 0.0 |
|  |  | 1 | 0 | 0.0 | 0.0 | 0.0 | 0.0 | 0.0 | 0.0 | 0.0 | 0.0 | 0.0 |
|  |  | 2 | 207 | 8.7 | -0.3 | 18.1 | 82.4 | 76.5 | 87.0 | 8.9 | 5.5 | 13.3 |
| Distant | 65-74 | 0 | 707 | 8.9 | 5.2 | 12.8 | 87.7 | 85.0 | 89.9 | 3.4 | 2.2 | 4.9 |
|  |  | 1 | 611 | 8.8 | 5.0 | 12.7 | 88.7 | 85.9 | 91.0 | 2.5 | 1.4 | 3.9 |
|  |  | 2 | 644 | 9.7 | 5.7 | 13.8 | 87.0 | 84.1 | 89.4 | 3.3 | 2.1 | 4.9 |
|  | 75-84 | 0 | 606 | 5.5 | 1.7 | 9.5 | 90.6 | 88.0 | 92.7 | 3.8 | 2.5 | 5.6 |
|  |  | 1 | 666 | 5.5 | 1.9 | 9.3 | 90.7 | 88.2 | 92.7 | 3.8 | 2.5 | 5.5 |
|  |  | 2 | 1036 | 6.7 | 3.5 | 10.1 | 88.3 | 86.2 | 90.1 | 5.0 | 3.8 | 6.4 |
|  | 85-94 | 0 | 115 | 3.7 | -4.6 | 13.1 | 92.7 | 85.7 | 96.4 | 3.6 | 1.2 | 8.3 |
|  |  | 1 | 178 | 2.3 | -5.6 | 10.9 | 90.8 | 85.4 | 94.3 | 6.8 | 3.7 | 11.3 |
|  |  | 2 | 280 | 3.2 | -3.3 | 10.1 | 89.3 | 85.1 | 92.4 | 7.5 | 4.8 | 10.9 |

## Table S14-3: Number of patients and **five**-year probabilities of dying from cancer, dying from other-causes, and survival among **males** who were diagnosed with squamous cell carcinoma lung cancer **(SCC)** by stage, age and comorbidity level (n>100)

| Stage | Age | Comorbidity | N | Survival (%) | | | Cancer deaths (%) | | | Other-Cause Deaths (%) | | |
| --- | --- | --- | --- | --- | --- | --- | --- | --- | --- | --- | --- | --- |
|  |  |  |  | Estimate | 95%CI. | | Estimate | 95%CI. | | Estimate | 95%CI. | |
| Localized | 65-74 | 0 | 156 | 44.1 | 30.3 | 56.7 | 48.3 | 39.5 | 56.7 | 7.6 | 3.8 | 13.0 |
|  |  | 1 | 226 | 51.3 | 39.3 | 62.3 | 37.0 | 30.2 | 43.8 | 11.8 | 7.6 | 16.9 |
|  |  | 2 | 303 | 36.4 | 26.7 | 45.8 | 54.6 | 48.3 | 60.5 | 9.0 | 5.9 | 12.8 |
|  | 75-84 | 0 | 135 | 29.9 | 16.5 | 43.0 | 61.7 | 52.6 | 69.6 | 8.4 | 4.4 | 13.9 |
|  |  | 1 | 188 | 29.9 | 16.2 | 43.3 | 54.1 | 46.0 | 61.5 | 16.0 | 10.7 | 22.2 |
|  |  | 2 | 387 | 22.1 | 13.4 | 30.8 | 66.2 | 60.6 | 71.1 | 11.8 | 8.6 | 15.6 |
|  | 85-94 | 0 | 0 | 0.0 | 0.0 | 0.0 | 0.0 | 0.0 | 0.0 | 0.0 | 0.0 | 0.0 |
|  |  | 1 | 0 | 0.0 | 0.0 | 0.0 | 0.0 | 0.0 | 0.0 | 0.0 | 0.0 | 0.0 |
|  |  | 2 | 0 | 0.0 | 0.0 | 0.0 | 0.0 | 0.0 | 0.0 | 0.0 | 0.0 | 0.0 |
| Regional | 65-74 | 0 | 567 | 10.4 | 5.5 | 15.4 | 84.8 | 81.3 | 87.6 | 4.8 | 3.2 | 6.9 |
|  |  | 1 | 494 | 13.0 | 7.6 | 18.6 | 82.7 | 78.8 | 86.0 | 4.2 | 2.6 | 6.4 |
|  |  | 2 | 581 | 11.9 | 6.7 | 17.3 | 82.0 | 78.4 | 85.0 | 6.1 | 4.3 | 8.3 |
|  | 75-84 | 0 | 410 | 6.4 | 1.6 | 11.4 | 90.0 | 86.5 | 92.7 | 3.6 | 2.0 | 5.8 |
|  |  | 1 | 490 | 6.7 | 1.6 | 12.0 | 87.5 | 84.1 | 90.2 | 5.8 | 3.9 | 8.2 |
|  |  | 2 | 855 | 3.8 | 0.0 | 7.6 | 89.6 | 87.3 | 91.6 | 6.6 | 5.1 | 8.4 |
|  | 85-94 | 0 | 0 | 0.0 | 0.0 | 0.0 | 0.0 | 0.0 | 0.0 | 0.0 | 0.0 | 0.0 |
|  |  | 1 | 0 | 0.0 | 0.0 | 0.0 | 0.0 | 0.0 | 0.0 | 0.0 | 0.0 | 0.0 |
|  |  | 2 | 207 | 0.9 | -7.7 | 10.2 | 88.6 | 83.0 | 92.4 | 10.5 | 6.8 | 15.3 |
| Distant | 65-74 | 0 | 707 | 1.6 | -1.4 | 4.8 | 94.8 | 92.8 | 96.3 | 3.6 | 2.4 | 5.2 |
|  |  | 1 | 611 | 1.8 | -1.2 | 5.1 | 95.8 | 93.5 | 97.3 | 2.5 | 1.4 | 3.9 |
|  |  | 2 | 644 | 0.8 | -2.4 | 4.3 | 95.1 | 93.1 | 96.6 | 4.0 | 2.7 | 5.8 |
|  | 75-84 | 0 | 606 | 0.9 | -2.4 | 4.6 | 94.9 | 92.7 | 96.4 | 4.2 | 2.8 | 6.0 |
|  |  | 1 | 666 | 1.4 | -2.0 | 4.9 | 94.1 | 92.0 | 95.7 | 4.5 | 3.1 | 6.3 |
|  |  | 2 | 1036 | 0.4 | -2.5 | 3.3 | 93.9 | 92.3 | 95.2 | 5.7 | 4.4 | 7.2 |
|  | 85-94 | 0 | 115 | 1.4 | -7.2 | 11.9 | 94.1 | 86.5 | 97.5 | 4.5 | 1.6 | 9.7 |
|  |  | 1 | 178 | 2.3 | -5.6 | 10.9 | 90.8 | 85.4 | 94.3 | 6.8 | 3.7 | 11.3 |
|  |  | 2 | 280 | 3.2 | -3.3 | 10.1 | 89.3 | 85.1 | 92.4 | 7.5 | 4.8 | 10.9 |

## Table S14-4: Number of patients and **one**-year probabilities of dying from cancer, dying from other-causes, and survival among **males** who were diagnosed with lung adenocarcinoma **(ADC)** by stage, age and comorbidity level (n>100)

| Stage | Age | Comorbidity | N | Survival (%) | | | Cancer deaths (%) | | | Other-Cause Deaths (%) | | |
| --- | --- | --- | --- | --- | --- | --- | --- | --- | --- | --- | --- | --- |
|  |  |  |  | Estimate | 95CI. | | Estimate | 95CI. | | Estimate | 95CI. | |
| Localized | 65-74 | 0 | 336 | 92.5 | 87.9 | 95.7 | 6.0 | 3.8 | 8.9 | 1.5 | 0.6 | 3.3 |
|  |  | 1 | 508 | 92.9 | 89.4 | 95.3 | 6.3 | 4.4 | 8.6 | 0.8 | 0.3 | 1.9 |
|  |  | 2 | 314 | 91.4 | 86.4 | 94.8 | 7.3 | 4.8 | 10.6 | 1.3 | 0.4 | 3.1 |
|  | 75-84 | 0 | 168 | 82.1 | 72.9 | 88.7 | 15.5 | 10.5 | 21.5 | 2.4 | 0.8 | 5.6 |
|  |  | 1 | 293 | 87.0 | 81.1 | 91.3 | 11.6 | 8.3 | 15.6 | 1.4 | 0.5 | 3.3 |
|  |  | 2 | 406 | 80.3 | 74.5 | 85.1 | 16.8 | 13.3 | 20.6 | 3.0 | 1.6 | 5.0 |
|  | 85-94 | 0 | 0 | 0.0 | 0.0 | 0.0 | 0.0 | 0.0 | 0.0 | 0.0 | 0.0 | 0.0 |
|  |  | 1 | 0 | 0.0 | 0.0 | 0.0 | 0.0 | 0.0 | 0.0 | 0.0 | 0.0 | 0.0 |
|  |  | 2 | 0 | 0.0 | 0.0 | 0.0 | 0.0 | 0.0 | 0.0 | 0.0 | 0.0 | 0.0 |
| Regional | 65-74 | 0 | 431 | 62.1 | 55.9 | 67.7 | 35.8 | 31.3 | 40.3 | 2.1 | 1.0 | 3.8 |
|  |  | 1 | 431 | 70.3 | 64.3 | 75.6 | 27.4 | 23.3 | 31.7 | 2.3 | 1.2 | 4.1 |
|  |  | 2 | 338 | 55.9 | 48.5 | 62.6 | 41.1 | 35.8 | 46.3 | 3.0 | 1.5 | 5.2 |
|  | 75-84 | 0 | 285 | 45.3 | 37.3 | 52.8 | 51.9 | 45.9 | 57.5 | 2.8 | 1.3 | 5.2 |
|  |  | 1 | 432 | 54.5 | 48.4 | 60.2 | 43.8 | 39.1 | 48.5 | 1.6 | 0.7 | 3.2 |
|  |  | 2 | 513 | 44.8 | 38.7 | 50.5 | 51.7 | 47.3 | 56.0 | 3.5 | 2.2 | 5.4 |
|  | 85-94 | 0 | 0 | 0.0 | 0.0 | 0.0 | 0.0 | 0.0 | 0.0 | 0.0 | 0.0 | 0.0 |
|  |  | 1 | 0 | 0.0 | 0.0 | 0.0 | 0.0 | 0.0 | 0.0 | 0.0 | 0.0 | 0.0 |
|  |  | 2 | 127 | 34.3 | 20.9 | 47.1 | 58.6 | 49.4 | 66.6 | 7.1 | 3.5 | 12.5 |
| Distant | 65-74 | 0 | 1669 | 40.4 | 37.3 | 43.4 | 57.5 | 55.1 | 59.9 | 2.0 | 1.4 | 2.8 |
|  |  | 1 | 1714 | 44.5 | 41.4 | 47.5 | 53.2 | 50.8 | 55.6 | 2.3 | 1.6 | 3.1 |
|  |  | 2 | 1064 | 34.7 | 30.9 | 38.5 | 62.9 | 59.9 | 65.7 | 2.4 | 1.6 | 3.4 |
|  | 75-84 | 0 | 1074 | 28.3 | 24.6 | 32.0 | 69.1 | 66.3 | 71.8 | 2.5 | 1.7 | 3.6 |
|  |  | 1 | 1574 | 32.7 | 29.5 | 35.8 | 64.8 | 62.4 | 67.1 | 2.5 | 1.8 | 3.3 |
|  |  | 2 | 1729 | 26.8 | 23.7 | 29.9 | 69.1 | 66.9 | 71.2 | 4.1 | 3.2 | 5.1 |
|  | 85-94 | 0 | 229 | 21.7 | 13.6 | 29.7 | 74.8 | 68.7 | 79.9 | 3.5 | 1.6 | 6.5 |
|  |  | 1 | 406 | 25.1 | 18.4 | 31.7 | 69.5 | 64.8 | 73.7 | 5.4 | 3.5 | 7.9 |
|  |  | 2 | 573 | 21.1 | 15.8 | 26.3 | 74.4 | 70.6 | 77.7 | 4.6 | 3.1 | 6.5 |

## Table S14-5: Number of patients and **two**-year probabilities of dying from cancer, dying from other-causes, and survival among **males** who were diagnosed with lung adenocarcinoma **(ADC)** by stage, age and comorbidity level (n>100)

| Stage | Age | Comorbidity | N | Survival (%) | | | Cancer deaths (%) | | | Other-Cause Deaths (%) | | |
| --- | --- | --- | --- | --- | --- | --- | --- | --- | --- | --- | --- | --- |
|  |  |  |  | Estimate | 95%CI. | | Estimate | 95%CI. | | Estimate | 95%CI. | |
| Localized | 65-74 | 0 | 336 | 84.3 | 78.1 | 89.1 | 13.0 | 9.6 | 16.9 | 2.7 | 1.3 | 4.9 |
|  |  | 1 | 508 | 85.9 | 81.3 | 89.6 | 12.0 | 9.3 | 15.1 | 2.0 | 1.0 | 3.6 |
|  |  | 2 | 314 | 82.5 | 75.8 | 87.8 | 14.2 | 10.5 | 18.4 | 3.4 | 1.7 | 5.9 |
|  | 75-84 | 0 | 168 | 69.2 | 58.6 | 77.8 | 27.8 | 21.0 | 34.8 | 3.1 | 1.1 | 6.6 |
|  |  | 1 | 293 | 81.1 | 74.4 | 86.4 | 16.8 | 12.7 | 21.4 | 2.1 | 0.9 | 4.3 |
|  |  | 2 | 406 | 63.8 | 56.7 | 70.2 | 31.1 | 26.6 | 35.8 | 5.1 | 3.2 | 7.6 |
|  | 85-94 | 0 | 0 | 0.0 | 0.0 | 0.0 | 0.0 | 0.0 | 0.0 | 0.0 | 0.0 | 0.0 |
|  |  | 1 | 0 | 0.0 | 0.0 | 0.0 | 0.0 | 0.0 | 0.0 | 0.0 | 0.0 | 0.0 |
|  |  | 2 | 0 | 0.0 | 0.0 | 0.0 | 0.0 | 0.0 | 0.0 | 0.0 | 0.0 | 0.0 |
| Regional | 65-74 | 0 | 431 | 43.2 | 36.6 | 49.5 | 53.5 | 48.6 | 58.1 | 3.3 | 1.9 | 5.3 |
|  |  | 1 | 431 | 48.8 | 42.1 | 55.0 | 48.1 | 43.3 | 52.8 | 3.0 | 1.7 | 5.0 |
|  |  | 2 | 338 | 38.3 | 30.4 | 45.7 | 56.9 | 51.4 | 62.1 | 4.8 | 2.9 | 7.5 |
|  | 75-84 | 0 | 285 | 28.5 | 20.6 | 36.2 | 67.6 | 61.7 | 72.7 | 3.9 | 2.1 | 6.7 |
|  |  | 1 | 432 | 31.5 | 25.0 | 37.7 | 65.0 | 60.2 | 69.3 | 3.5 | 2.1 | 5.6 |
|  |  | 2 | 513 | 27.1 | 21.1 | 33.0 | 68.0 | 63.7 | 71.9 | 4.9 | 3.3 | 7.0 |
|  | 85-94 | 0 | 0 | 0.0 | 0.0 | 0.0 | 0.0 | 0.0 | 0.0 | 0.0 | 0.0 | 0.0 |
|  |  | 1 | 0 | 0.0 | 0.0 | 0.0 | 0.0 | 0.0 | 0.0 | 0.0 | 0.0 | 0.0 |
|  |  | 2 | 127 | 12.9 | 0.9 | 25.4 | 79.1 | 70.5 | 85.4 | 8.0 | 4.1 | 13.7 |
| Distant | 65-74 | 0 | 1669 | 19.5 | 16.7 | 22.3 | 77.8 | 75.7 | 79.8 | 2.7 | 2.0 | 3.6 |
|  |  | 1 | 1714 | 23.1 | 20.2 | 26.0 | 74.0 | 71.9 | 76.1 | 2.9 | 2.2 | 3.8 |
|  |  | 2 | 1064 | 17.7 | 14.3 | 21.2 | 79.4 | 76.8 | 81.8 | 2.8 | 2.0 | 4.0 |
|  | 75-84 | 0 | 1074 | 11.4 | 8.2 | 14.6 | 85.5 | 83.2 | 87.5 | 3.1 | 2.2 | 4.3 |
|  |  | 1 | 1574 | 13.2 | 10.4 | 16.0 | 83.0 | 81.0 | 84.7 | 3.9 | 3.0 | 4.9 |
|  |  | 2 | 1729 | 11.4 | 8.6 | 14.3 | 83.0 | 81.1 | 84.6 | 5.6 | 4.6 | 6.8 |
|  | 85-94 | 0 | 229 | 6.7 | 0.1 | 13.7 | 89.3 | 84.4 | 92.7 | 4.0 | 2.0 | 7.1 |
|  |  | 1 | 406 | 8.6 | 2.7 | 14.8 | 84.4 | 80.5 | 87.6 | 6.9 | 4.7 | 9.7 |
|  |  | 2 | 573 | 8.4 | 3.8 | 13.2 | 86.1 | 83.0 | 88.7 | 5.5 | 3.8 | 7.5 |

## Table S14-6: Number of patients and **five**-year probabilities of dying from cancer, dying from other-causes, and survival among **males** who were diagnosed with lung adenocarcinoma **(ADC)** by stage, age and comorbidity level (n>100)

| Stage | Age | Comorbidity | N | Survival (%) | | | Cancer deaths (%) | | | Other-Cause Deaths (%) | | |
| --- | --- | --- | --- | --- | --- | --- | --- | --- | --- | --- | --- | --- |
|  |  |  |  | Estimate | 95%CI. | | Estimate | 95%CI. | | Estimate | 95%CI. | |
| Localized | 65-74 | 0 | 336 | 58.9 | 50.1 | 66.9 | 36.8 | 30.7 | 42.8 | 4.3 | 2.4 | 7.1 |
|  |  | 1 | 508 | 65.8 | 58.3 | 72.4 | 29.4 | 24.6 | 34.2 | 4.9 | 3.0 | 7.4 |
|  |  | 2 | 314 | 55.0 | 44.8 | 64.4 | 36.4 | 30.1 | 42.7 | 8.6 | 5.5 | 12.6 |
|  | 75-84 | 0 | 168 | 44.2 | 30.0 | 57.3 | 46.0 | 37.4 | 54.3 | 9.7 | 5.3 | 15.7 |
|  |  | 1 | 293 | 49.2 | 38.9 | 58.8 | 44.8 | 38.0 | 51.4 | 5.9 | 3.2 | 9.7 |
|  |  | 2 | 406 | 33.0 | 23.8 | 42.0 | 54.0 | 48.4 | 59.3 | 13.0 | 9.6 | 16.9 |
|  | 85-94 | 0 | 0 | 0.0 | 0.0 | 0.0 | 0.0 | 0.0 | 0.0 | 0.0 | 0.0 | 0.0 |
|  |  | 1 | 0 | 0.0 | 0.0 | 0.0 | 0.0 | 0.0 | 0.0 | 0.0 | 0.0 | 0.0 |
|  |  | 2 | 0 | 0.0 | 0.0 | 0.0 | 0.0 | 0.0 | 0.0 | 0.0 | 0.0 | 0.0 |
| Regional | 65-74 | 0 | 431 | 16.4 | 10.4 | 22.6 | 79.2 | 74.7 | 83.0 | 4.3 | 2.7 | 6.6 |
|  |  | 1 | 431 | 17.6 | 11.2 | 24.2 | 77.3 | 72.7 | 81.3 | 5.0 | 3.2 | 7.5 |
|  |  | 2 | 338 | 13.2 | 5.8 | 20.8 | 80.5 | 75.2 | 84.7 | 6.4 | 4.0 | 9.5 |
|  | 75-84 | 0 | 285 | 6.1 | -0.3 | 12.8 | 88.8 | 84.2 | 92.1 | 5.2 | 2.9 | 8.3 |
|  |  | 1 | 432 | 10.3 | 4.1 | 16.7 | 83.3 | 79.2 | 86.7 | 6.4 | 4.2 | 9.2 |
|  |  | 2 | 513 | 8.9 | 3.2 | 14.8 | 83.5 | 79.8 | 86.6 | 7.5 | 5.4 | 10.2 |
|  | 85-94 | 0 | 0 | 0.0 | 0.0 | 0.0 | 0.0 | 0.0 | 0.0 | 0.0 | 0.0 | 0.0 |
|  |  | 1 | 0 | 0.0 | 0.0 | 0.0 | 0.0 | 0.0 | 0.0 | 0.0 | 0.0 | 0.0 |
|  |  | 2 | 127 | 2.8 | -7.5 | 14.5 | 89.2 | 81.4 | 93.8 | 8.0 | 4.1 | 13.7 |
| Distant | 65-74 | 0 | 1669 | 2.8 | 0.7 | 5.0 | 93.7 | 92.3 | 94.8 | 3.5 | 2.7 | 4.5 |
|  |  | 1 | 1714 | 4.2 | 1.9 | 6.5 | 92.0 | 90.5 | 93.3 | 3.8 | 3.0 | 4.8 |
|  |  | 2 | 1064 | 3.6 | 0.9 | 6.4 | 93.1 | 91.3 | 94.6 | 3.3 | 2.3 | 4.5 |
|  | 75-84 | 0 | 1074 | 1.4 | -1.0 | 3.9 | 95.2 | 93.6 | 96.3 | 3.4 | 2.4 | 4.6 |
|  |  | 1 | 1574 | 2.1 | -0.2 | 4.5 | 93.2 | 91.7 | 94.4 | 4.7 | 3.7 | 5.8 |
|  |  | 2 | 1729 | 1.5 | -0.9 | 4.1 | 92.1 | 90.7 | 93.3 | 6.4 | 5.3 | 7.6 |
|  | 85-94 | 0 | 229 | 0.5 | -5.1 | 6.7 | 95.0 | 91.0 | 97.2 | 4.5 | 2.3 | 7.8 |
|  |  | 1 | 406 | 1.1 | -4.2 | 6.7 | 91.4 | 88.0 | 93.8 | 7.6 | 5.2 | 10.4 |
|  |  | 2 | 573 | 1.7 | -2.4 | 6.0 | 92.5 | 89.9 | 94.4 | 5.9 | 4.1 | 8.0 |

## Table S14-7. Number of patients and **one**-year probabilities of dying from cancer, dying from other-causes, and survival among **females** who were diagnosed with lung Adenocarcinoma **(ADC)** by stage, age and comorbidity level (n>100)

| Stage | Age | Comorbidity | N | Survival (%) | | | Cancer deaths (%) | | | Other-Cause Deaths (%) | | |
| --- | --- | --- | --- | --- | --- | --- | --- | --- | --- | --- | --- | --- |
|  |  |  |  | Estimate | 95%CI. | | Estimate | 95%CI. | | Estimate | 95%CI. | |
| Localized | 65-74 | 0 | 429 | 98.1 | 95.5 | 99.3 | 1.4 | 0.6 | 2.9 | 0.5 | 0.1 | 1.6 |
|  |  | 1 | 720 | 97.8 | 95.9 | 98.8 | 1.9 | 1.1 | 3.2 | 0.3 | 0.1 | 1.0 |
|  |  | 2 | 381 | 94.7 | 91.0 | 97.1 | 4.5 | 2.7 | 6.9 | 0.8 | 0.2 | 2.2 |
|  | 75-84 | 0 | 127 | 90.6 | 84.7 | 94.9 | 9.4 | 5.1 | 15.3 | 0.0 | NA | NA |
|  |  | 1 | 378 | 95.2 | 91.6 | 97.4 | 4.2 | 2.5 | 6.6 | 0.5 | 0.1 | 1.8 |
|  |  | 2 | 270 | 86.3 | 79.5 | 91.4 | 8.9 | 5.9 | 12.7 | 4.8 | 2.7 | 7.8 |
|  | 85-94 | 0 | 0 | 0.0 | 0.0 | 0.0 | 0.0 | 0.0 | 0.0 | 0.0 | 0.0 | 0.0 |
|  |  | 1 | 0 | 0.0 | 0.0 | 0.0 | 0.0 | 0.0 | 0.0 | 0.0 | 0.0 | 0.0 |
|  |  | 2 | 0 | 0.0 | 0.0 | 0.0 | 0.0 | 0.0 | 0.0 | 0.0 | 0.0 | 0.0 |
| Regional | 65-74 | 0 | 327 | 80.7 | 74.7 | 85.3 | 18.4 | 14.4 | 22.8 | 0.9 | 0.3 | 2.5 |
|  |  | 1 | 503 | 80.3 | 75.4 | 84.3 | 18.3 | 15.1 | 21.8 | 1.4 | 0.6 | 2.7 |
|  |  | 2 | 265 | 72.1 | 63.9 | 79.0 | 23.8 | 18.8 | 29.1 | 4.2 | 2.2 | 7.1 |
|  | 75-84 | 0 | 151 | 61.6 | 53.9 | 69.4 | 38.4 | 30.6 | 46.1 | 0.0 | NA | NA |
|  |  | 1 | 375 | 66.7 | 60.1 | 72.4 | 31.2 | 26.6 | 35.9 | 2.1 | 1.0 | 4.0 |
|  |  | 2 | 262 | 58.0 | 49.6 | 65.4 | 39.3 | 33.4 | 45.2 | 2.7 | 1.2 | 5.2 |
|  | 85-94 | 0 | 0 | 0.0 | 0.0 | 0.0 | 0.0 | 0.0 | 0.0 | 0.0 | 0.0 | 0.0 |
|  |  | 1 | 0 | 0.0 | 0.0 | 0.0 | 0.0 | 0.0 | 0.0 | 0.0 | 0.0 | 0.0 |
|  |  | 2 | 107 | 38.3 | 24.7 | 50.8 | 57.9 | 48.0 | 66.7 | 3.7 | 1.2 | 8.6 |
| Distant | 65-74 | 0 | 1489 | 59.0 | 55.8 | 62.1 | 39.4 | 36.9 | 41.8 | 1.6 | 1.1 | 2.4 |
|  |  | 1 | 1970 | 60.4 | 57.7 | 62.9 | 38.6 | 36.5 | 40.8 | 1.0 | 0.6 | 1.5 |
|  |  | 2 | 871 | 51.7 | 47.3 | 55.8 | 46.2 | 42.8 | 49.4 | 2.2 | 1.4 | 3.3 |
|  | 75-84 | 0 | 779 | 43.5 | 38.8 | 47.9 | 54.2 | 50.7 | 57.6 | 2.3 | 1.4 | 3.6 |
|  |  | 1 | 1757 | 48.3 | 45.1 | 51.3 | 49.1 | 46.7 | 51.4 | 2.7 | 2.0 | 3.5 |
|  |  | 2 | 1099 | 39.3 | 35.1 | 43.4 | 56.4 | 53.4 | 59.3 | 4.3 | 3.2 | 5.6 |
|  | 85-94 | 0 | 201 | 25.9 | 16.2 | 35.3 | 68.7 | 61.8 | 74.6 | 5.5 | 2.9 | 9.2 |
|  |  | 1 | 477 | 31.6 | 25.5 | 37.5 | 64.6 | 60.1 | 68.7 | 3.8 | 2.3 | 5.8 |
|  |  | 2 | 401 | 23.4 | 17.0 | 29.8 | 72.1 | 67.4 | 76.2 | 4.5 | 2.8 | 6.8 |

## Table S14-8: Number of patients and **two**-year probabilities of dying from cancer, dying from other-causes, and survival among **females** who were diagnosed with lung Adenocarcinoma **(ADC)** by stage, age and comorbidity level (n>100)

| Stage | Age | Comorbidity | N | Survival (%) | | | Cancer deaths (%) | | | Other-Cause Deaths (%) | | |
| --- | --- | --- | --- | --- | --- | --- | --- | --- | --- | --- | --- | --- |
|  |  |  |  | Estimate | 95%CI. | | Estimate | 95%CI. | | Estimate | 95%CI. | |
| Localized | 65-74 | 0 | 429 | 93.6 | 89.8 | 96.0 | 6.0 | 3.9 | 8.6 | 0.5 | 0.1 | 1.6 |
|  |  | 1 | 720 | 95.2 | 92.7 | 97.0 | 4.1 | 2.8 | 5.7 | 0.7 | 0.3 | 1.6 |
|  |  | 2 | 381 | 89.6 | 84.6 | 93.3 | 8.2 | 5.7 | 11.4 | 2.1 | 1.0 | 4.0 |
|  | 75-84 | 0 | 127 | 82.5 | 71.6 | 89.4 | 16.6 | 10.6 | 23.9 | 0.9 | 0.1 | 4.6 |
|  |  | 1 | 378 | 87.0 | 81.7 | 91.0 | 11.3 | 8.3 | 14.9 | 1.7 | 0.7 | 3.5 |
|  |  | 2 | 270 | 74.9 | 66.6 | 81.8 | 19.9 | 15.2 | 25.0 | 5.2 | 3.0 | 8.3 |
|  | 85-94 | 0 | 0 | 0.0 | 0.0 | 0.0 | 0.0 | 0.0 | 0.0 | 0.0 | 0.0 | 0.0 |
|  |  | 1 | 0 | 0.0 | 0.0 | 0.0 | 0.0 | 0.0 | 0.0 | 0.0 | 0.0 | 0.0 |
|  |  | 2 | 0 | 0.0 | 0.0 | 0.0 | 0.0 | 0.0 | 0.0 | 0.0 | 0.0 | 0.0 |
| Regional | 65-74 | 0 | 327 | 66.4 | 59.2 | 72.6 | 31.7 | 26.6 | 36.9 | 1.9 | 0.8 | 3.9 |
|  |  | 1 | 503 | 66.0 | 60.3 | 71.1 | 32.0 | 27.9 | 36.2 | 2.0 | 1.0 | 3.6 |
|  |  | 2 | 265 | 56.8 | 47.7 | 65.0 | 37.8 | 31.9 | 43.8 | 5.3 | 3.1 | 8.5 |
|  | 75-84 | 0 | 151 | 39.8 | 29.0 | 49.7 | 58.2 | 49.7 | 65.6 | 2.0 | 0.5 | 5.4 |
|  |  | 1 | 375 | 43.7 | 36.5 | 50.5 | 52.8 | 47.6 | 57.8 | 3.5 | 2.0 | 5.7 |
|  |  | 2 | 262 | 34.6 | 25.9 | 42.9 | 61.1 | 54.8 | 66.9 | 4.3 | 2.3 | 7.2 |
|  | 85-94 | 0 | 0 | 0.0 | 0.0 | 0.0 | 0.0 | 0.0 | 0.0 | 0.0 | 0.0 | 0.0 |
|  |  | 1 | 0 | 0.0 | 0.0 | 0.0 | 0.0 | 0.0 | 0.0 | 0.0 | 0.0 | 0.0 |
|  |  | 2 | 107 | 13.4 | 1.0 | 26.2 | 80.0 | 70.9 | 86.5 | 6.6 | 2.9 | 12.5 |
| Distant | 65-74 | 0 | 1489 | 32.7 | 29.4 | 35.9 | 64.8 | 62.3 | 67.3 | 2.5 | 1.8 | 3.4 |
|  |  | 1 | 1970 | 35.5 | 32.6 | 38.2 | 62.5 | 60.3 | 64.7 | 2.0 | 1.5 | 2.7 |
|  |  | 2 | 871 | 27.1 | 22.8 | 31.3 | 69.7 | 66.5 | 72.6 | 3.2 | 2.2 | 4.6 |
|  | 75-84 | 0 | 779 | 20.8 | 16.5 | 25.1 | 76.0 | 72.8 | 78.9 | 3.1 | 2.1 | 4.5 |
|  |  | 1 | 1757 | 24.4 | 21.3 | 27.5 | 71.5 | 69.3 | 73.6 | 4.1 | 3.3 | 5.1 |
|  |  | 2 | 1099 | 20.0 | 16.1 | 23.9 | 75.0 | 72.3 | 77.4 | 5.0 | 3.9 | 6.5 |
|  | 85-94 | 0 | 201 | 9.8 | 1.4 | 18.6 | 83.7 | 77.8 | 88.2 | 6.5 | 3.6 | 10.5 |
|  |  | 1 | 477 | 11.5 | 6.1 | 17.0 | 83.2 | 79.5 | 86.3 | 5.3 | 3.5 | 7.6 |
|  |  | 2 | 401 | 10.1 | 4.3 | 16.0 | 84.4 | 80.4 | 87.6 | 5.5 | 3.6 | 8.1 |

## Table S14-9. Number of patients and **five**-year probabilities of dying from cancer, dying from other-causes, and survival among **females** who were diagnosed with lung Adenocarcinoma **(ADC)** by stage, age and comorbidity level (n>100)

| Stage | Age | Comorbidity | N | Survival (%) | | | Cancer deaths (%) | | | Other-Cause Deaths (%) | | |
| --- | --- | --- | --- | --- | --- | --- | --- | --- | --- | --- | --- | --- |
|  |  |  |  | Estimate | 95%CI. | | Estimate | 95%CI. | | Estimate | 95%CI. | |
| Localized | 65-74 | 0 | 429 | 78.2 | 71.5 | 83.7 | 19.9 | 15.6 | 24.6 | 1.9 | 0.8 | 3.9 |
|  |  | 1 | 720 | 79.4 | 74.0 | 83.9 | 18.2 | 14.7 | 21.9 | 2.4 | 1.3 | 4.1 |
|  |  | 2 | 381 | 72.0 | 63.8 | 79.1 | 22.5 | 17.8 | 27.7 | 5.4 | 3.2 | 8.5 |
|  | 75-84 | 0 | 127 | 55.9 | 38.5 | 70.3 | 36.4 | 26.6 | 46.3 | 7.7 | 3.1 | 15.2 |
|  |  | 1 | 378 | 64.6 | 55.9 | 72.2 | 31.1 | 25.5 | 36.8 | 4.3 | 2.3 | 7.3 |
|  |  | 2 | 270 | 49.6 | 38.1 | 60.3 | 38.3 | 31.6 | 45.0 | 12.0 | 8.1 | 16.8 |
|  | 85-94 | 0 | 0 | 0.0 | 0.0 | 0.0 | 0.0 | 0.0 | 0.0 | 0.0 | 0.0 | 0.0 |
|  |  | 1 | 0 | 0.0 | 0.0 | 0.0 | 0.0 | 0.0 | 0.0 | 0.0 | 0.0 | 0.0 |
|  |  | 2 | 0 | 0.0 | 0.0 | 0.0 | 0.0 | 0.0 | 0.0 | 0.0 | 0.0 | 0.0 |
| Regional | 65-74 | 0 | 327 | 34.6 | 26.8 | 42.1 | 63.1 | 56.9 | 68.7 | 2.3 | 1.0 | 4.5 |
|  |  | 1 | 503 | 26.1 | 19.4 | 32.7 | 69.2 | 64.3 | 73.5 | 4.7 | 3.0 | 7.1 |
|  |  | 2 | 265 | 22.6 | 13.0 | 32.4 | 70.5 | 63.5 | 76.4 | 6.9 | 4.2 | 10.6 |
|  | 75-84 | 0 | 151 | 17.9 | 8.1 | 27.9 | 79.3 | 71.2 | 85.3 | 2.8 | 0.9 | 6.6 |
|  |  | 1 | 375 | 16.7 | 9.2 | 24.4 | 76.2 | 71.0 | 80.6 | 7.1 | 4.6 | 10.3 |
|  |  | 2 | 262 | 11.5 | 2.7 | 20.7 | 81.9 | 75.5 | 86.7 | 6.6 | 3.8 | 10.6 |
|  | 85-94 | 0 | 0 | 0.0 | 0.0 | 0.0 | 0.0 | 0.0 | 0.0 | 0.0 | 0.0 | 0.0 |
|  |  | 1 | 0 | 0.0 | 0.0 | 0.0 | 0.0 | 0.0 | 0.0 | 0.0 | 0.0 | 0.0 |
|  |  | 2 | 107 | 1.7 | -9.1 | 14.8 | 90.7 | 81.7 | 95.4 | 7.6 | 3.5 | 13.7 |
| Distant | 65-74 | 0 | 1489 | 7.2 | 4.5 | 9.9 | 89.7 | 87.8 | 91.3 | 3.1 | 2.3 | 4.1 |
|  |  | 1 | 1970 | 7.3 | 5.0 | 9.7 | 89.7 | 88.0 | 91.1 | 3.0 | 2.3 | 3.9 |
|  |  | 2 | 871 | 6.3 | 2.6 | 10.1 | 89.1 | 86.5 | 91.1 | 4.7 | 3.3 | 6.3 |
|  | 75-84 | 0 | 779 | 3.3 | -0.1 | 6.9 | 92.6 | 90.3 | 94.4 | 4.1 | 2.8 | 5.7 |
|  |  | 1 | 1757 | 4.1 | 1.5 | 6.7 | 90.5 | 88.9 | 91.9 | 5.4 | 4.4 | 6.6 |
|  |  | 2 | 1099 | 3.0 | -0.2 | 6.3 | 91.0 | 89.0 | 92.7 | 6.0 | 4.7 | 7.6 |
|  | 85-94 | 0 | 201 | 2.9 | -4.8 | 11.2 | 90.1 | 84.8 | 93.7 | 7.0 | 4.0 | 11.1 |
|  |  | 1 | 477 | 1.1 | -3.7 | 6.2 | 92.2 | 89.2 | 94.4 | 6.7 | 4.6 | 9.3 |
|  |  | 2 | 401 | 1.5 | -3.9 | 7.2 | 91.5 | 88.2 | 94.0 | 7.0 | 4.7 | 9.9 |

## Figure S1-1. Prevalence of cancer survivors by calendar year and years from diagnosis: **All cancer**


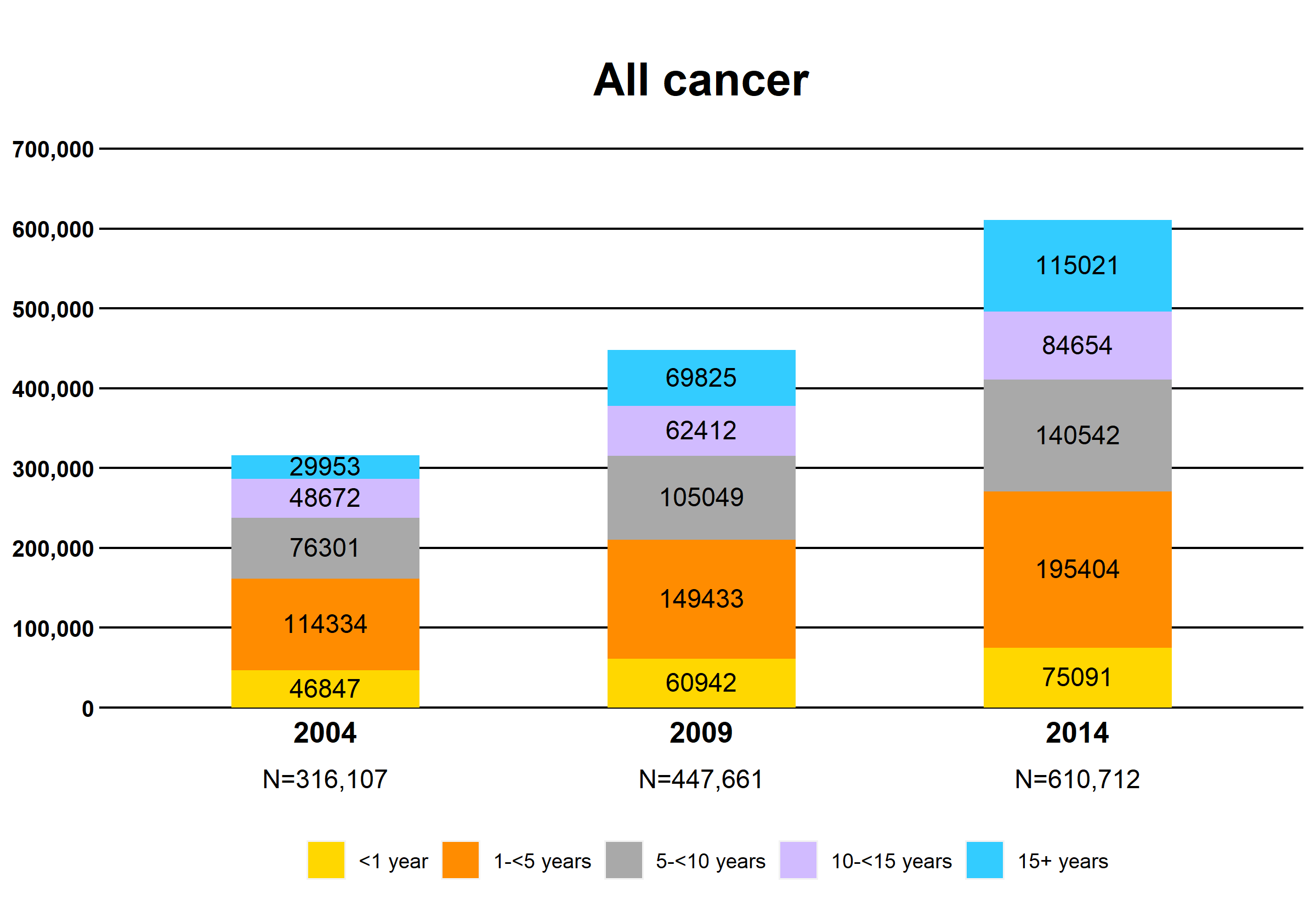


## Figure S1-2. Prevalence of cancer survivors by calendar year and years from diagnosis: **Breast Cancer**


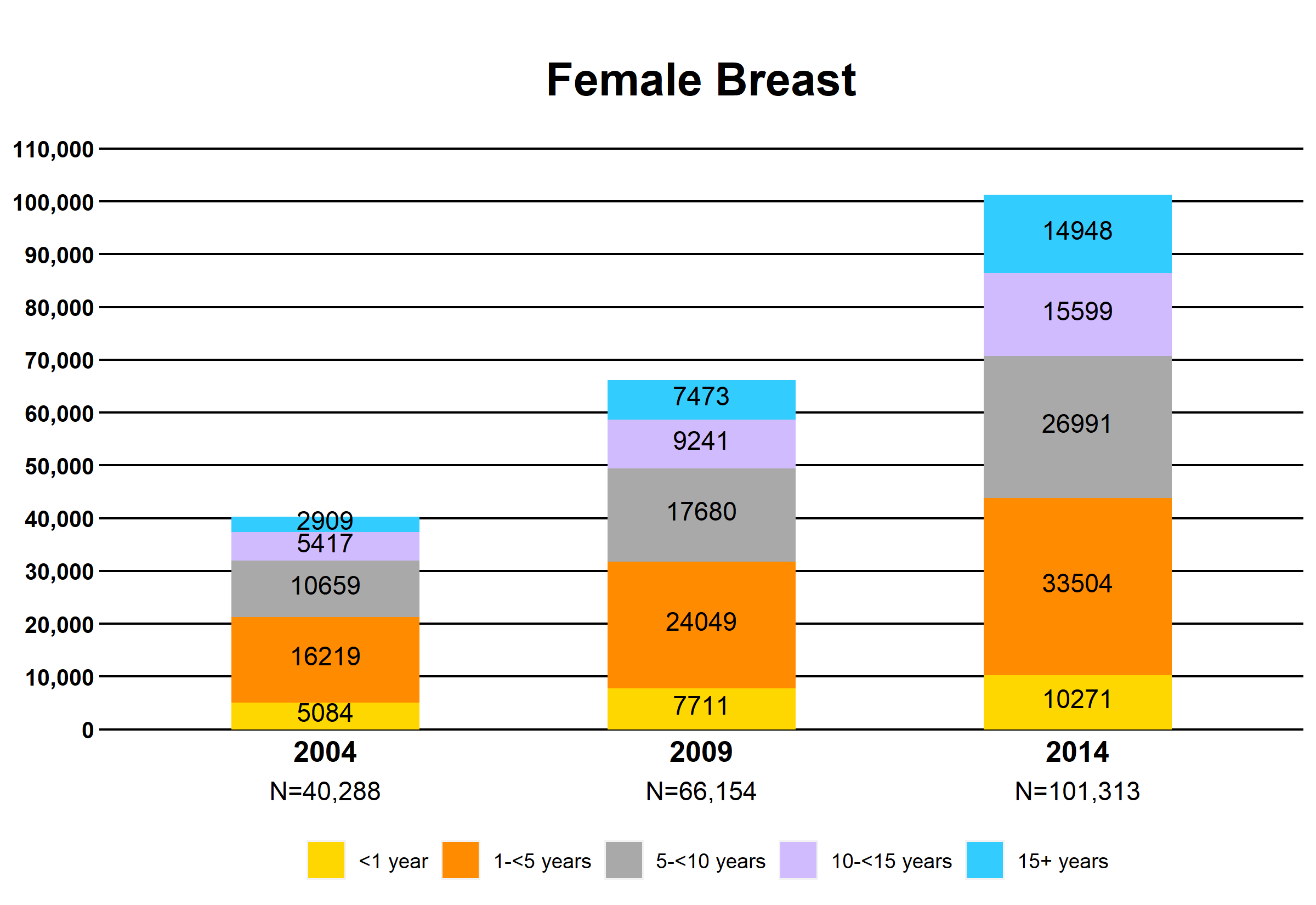


## Figure S1-3. Prevalence of cancer survivors by calendar year and years from diagnosis: **Colorectal Cancer**


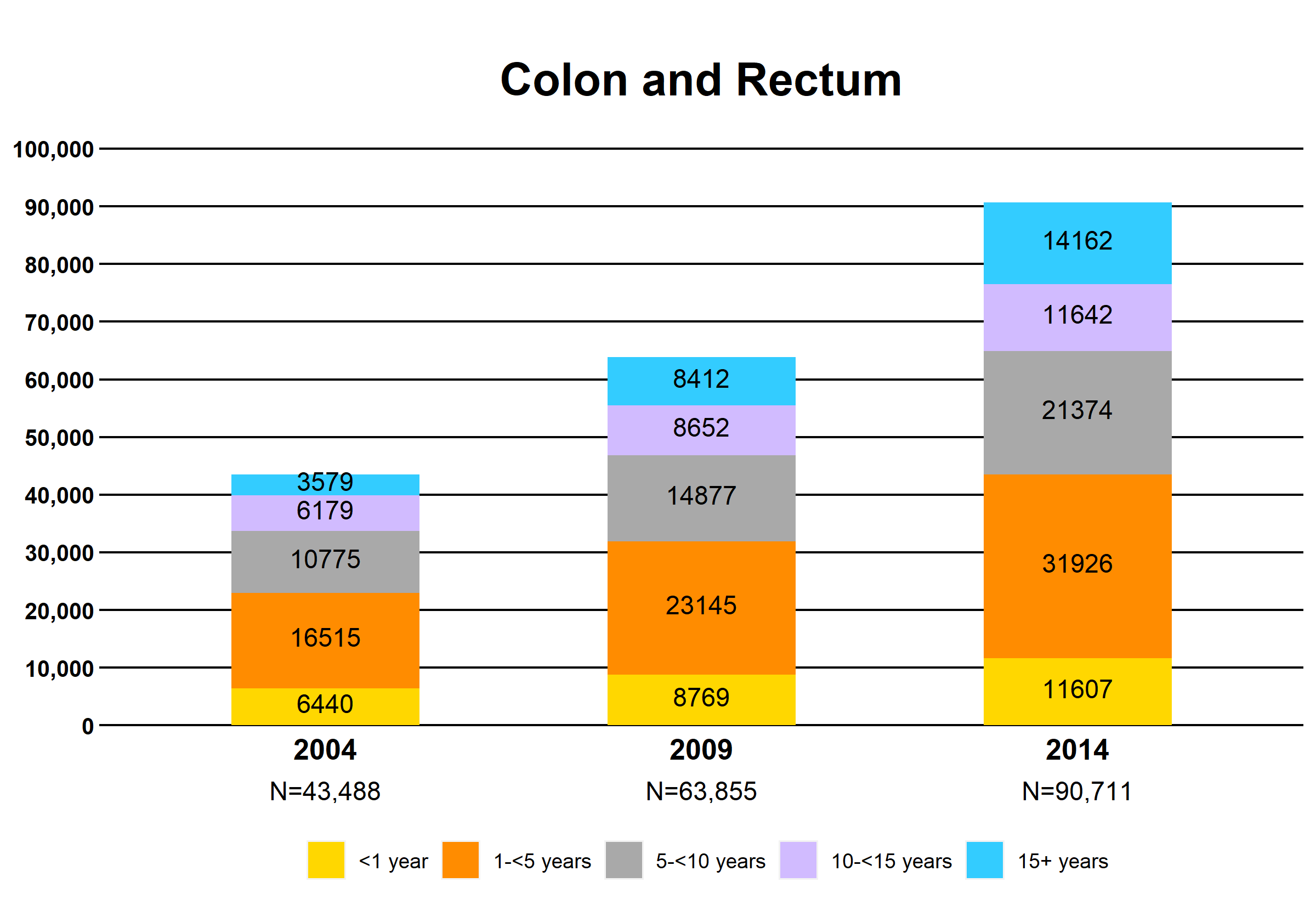


## Figure S1-4. Prevalence of cancer survivors by calendar year and years from diagnosis: **Liver Cancer**


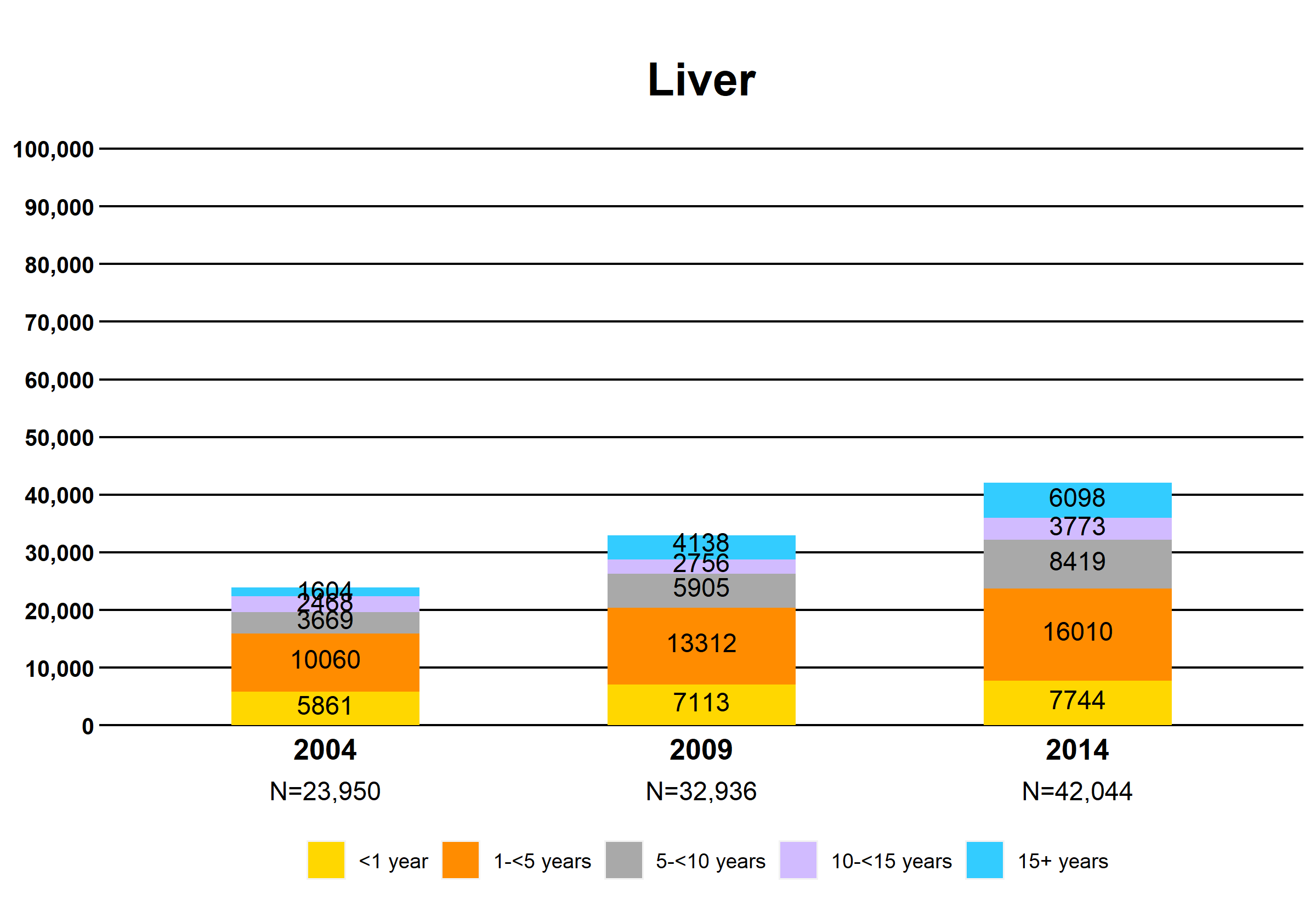


## Figure S1-5. Prevalence of cancer survivors by calendar year and years from diagnosis: **Lung Cancer**


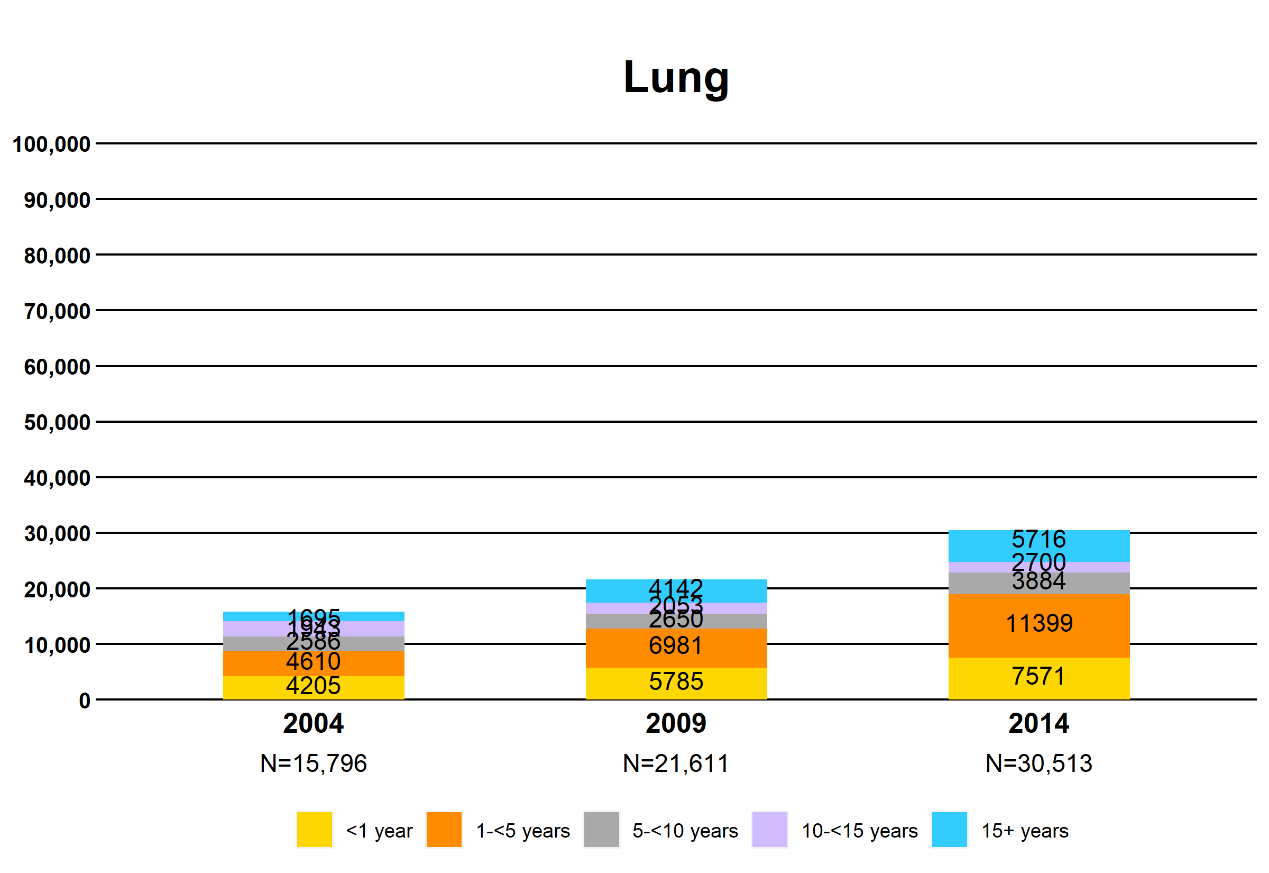


## Figure S1-6. Prevalence of cancer survivors by calendar year and years from diagnosis: **Oral Cancer**


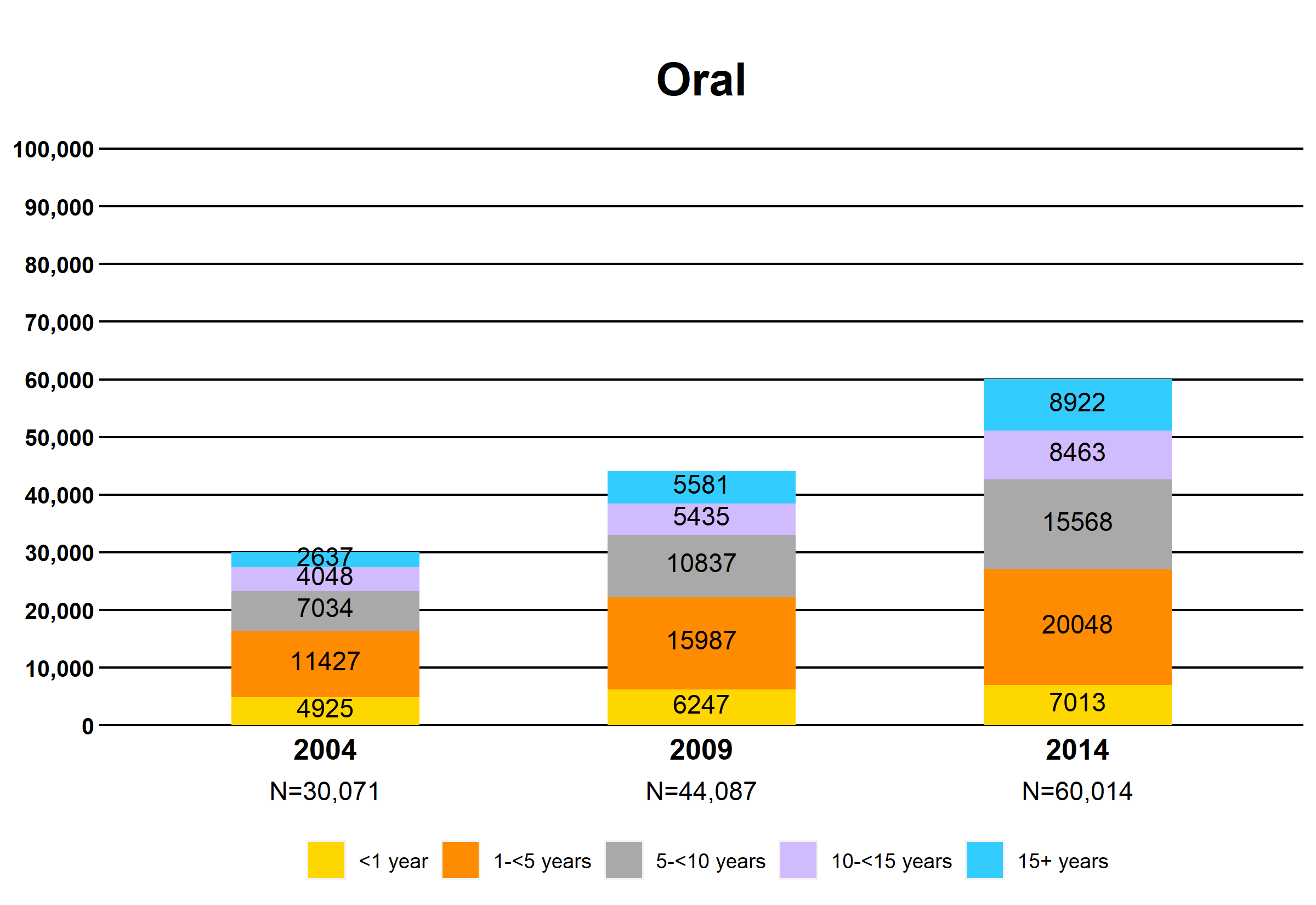


## Figure S2-1. Probabilities of dying from cancer, dying from other causes, and survival stratified by stage, comorbidity level, and age for **breast cancer**


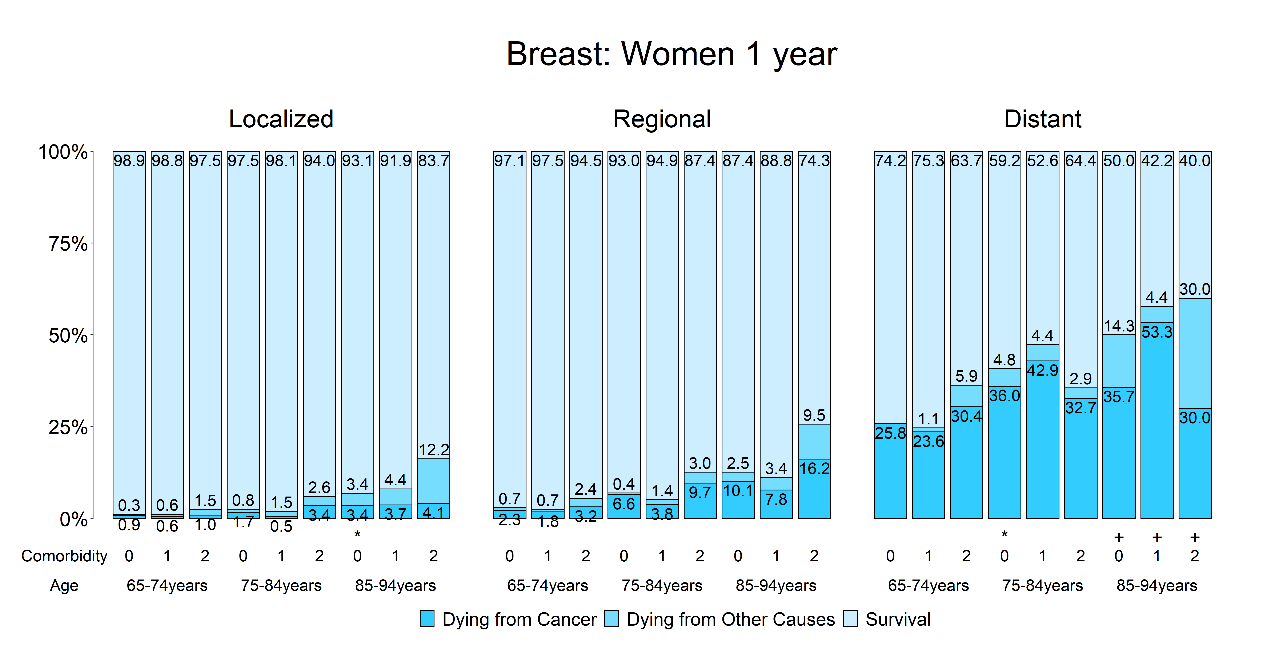


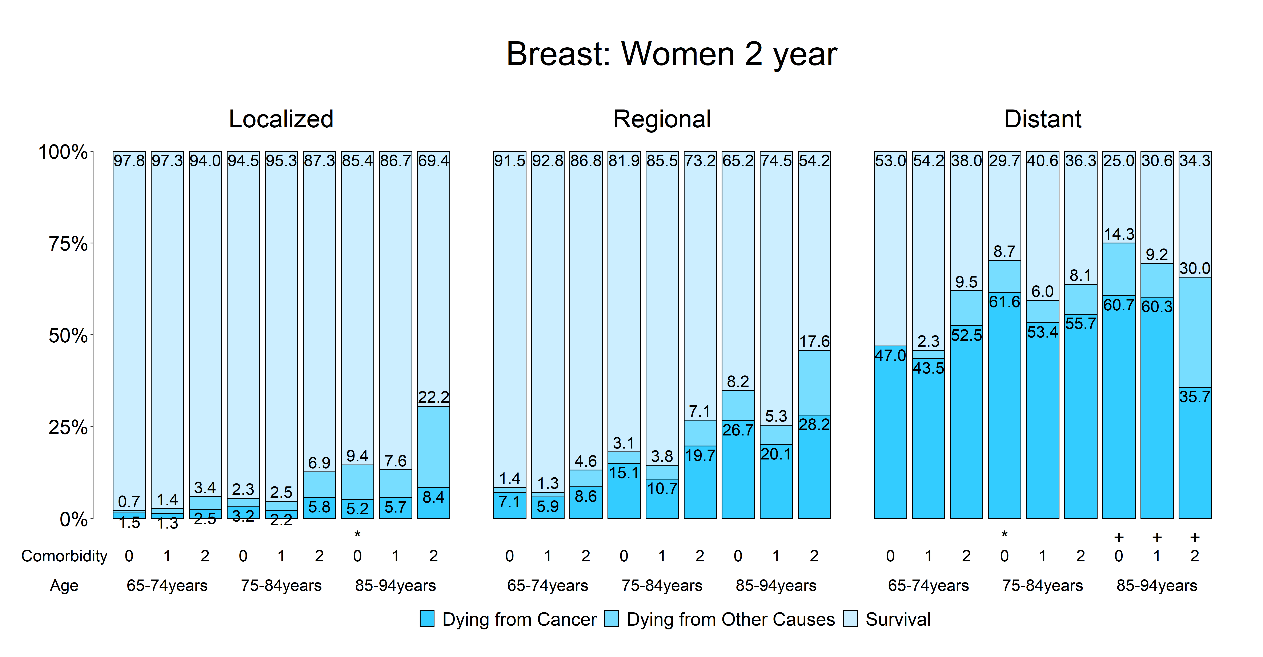

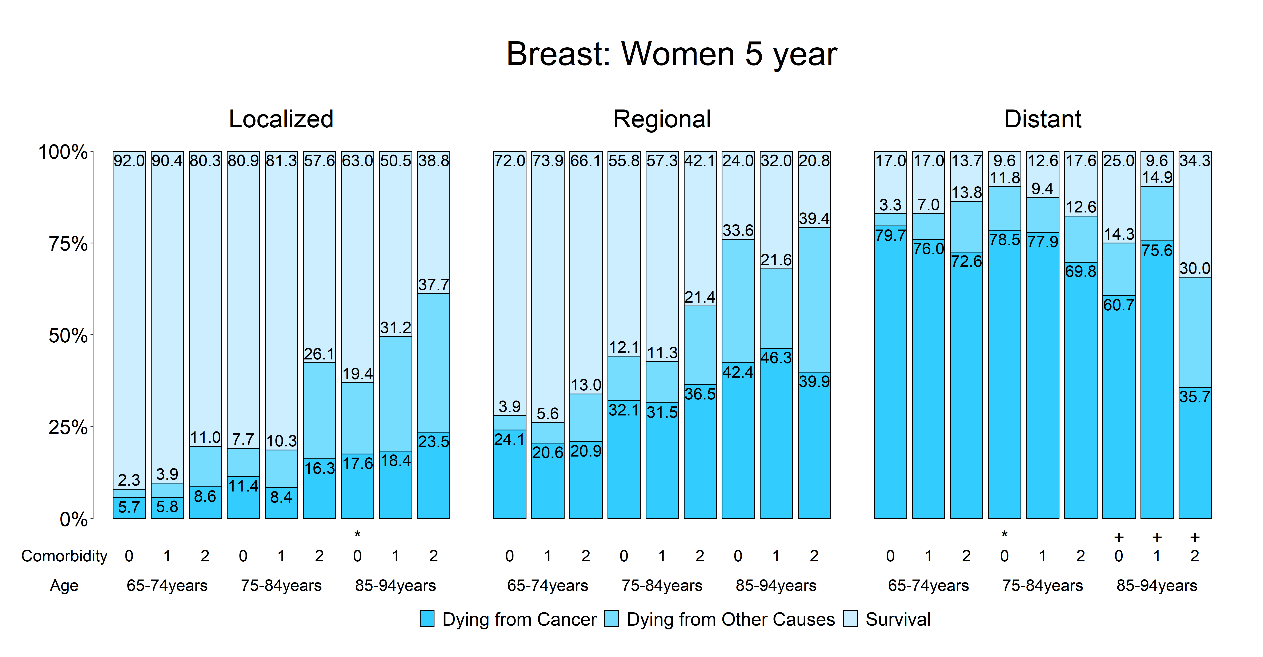


## Figure S2-2. Probabilities of dying from cancer, dying from other causes, and survival stratified by stage, comorbidity level, and age for **colorectal cancer**

**
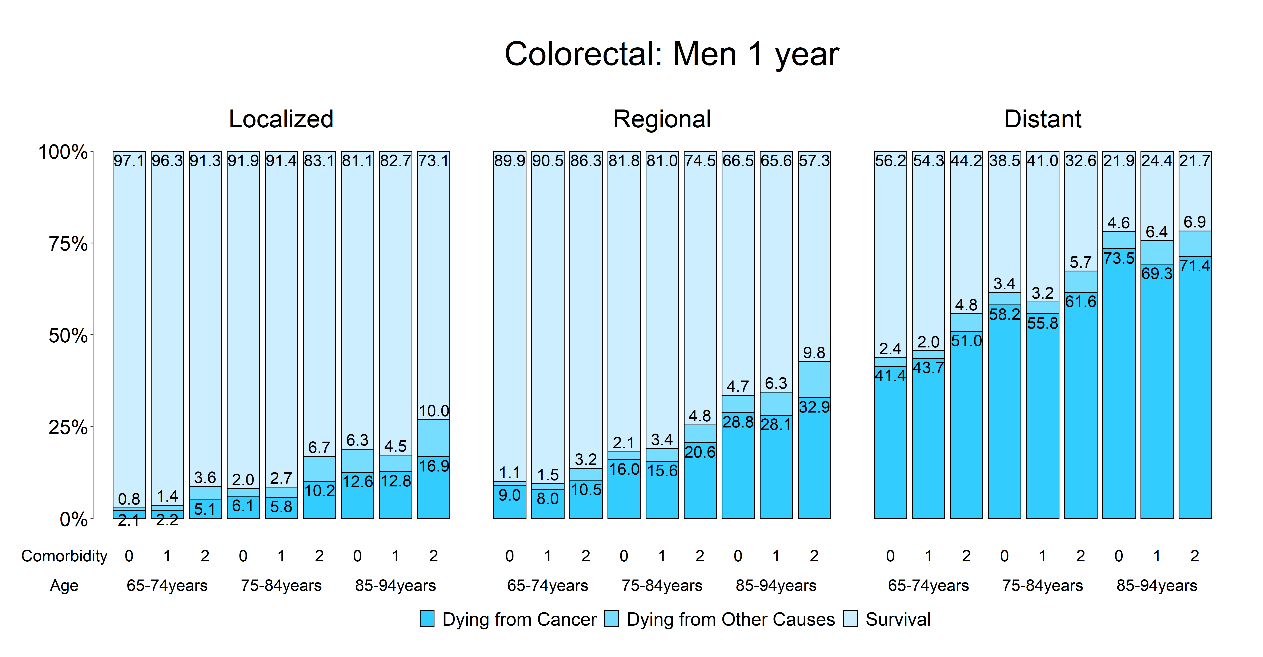

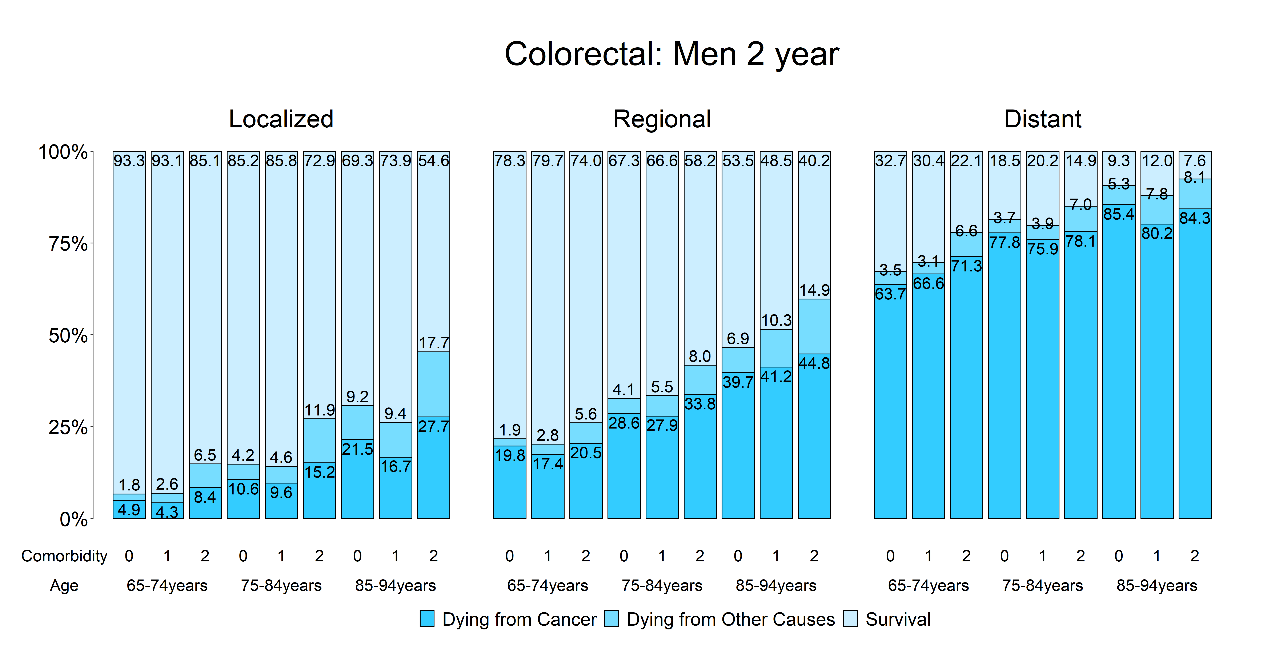

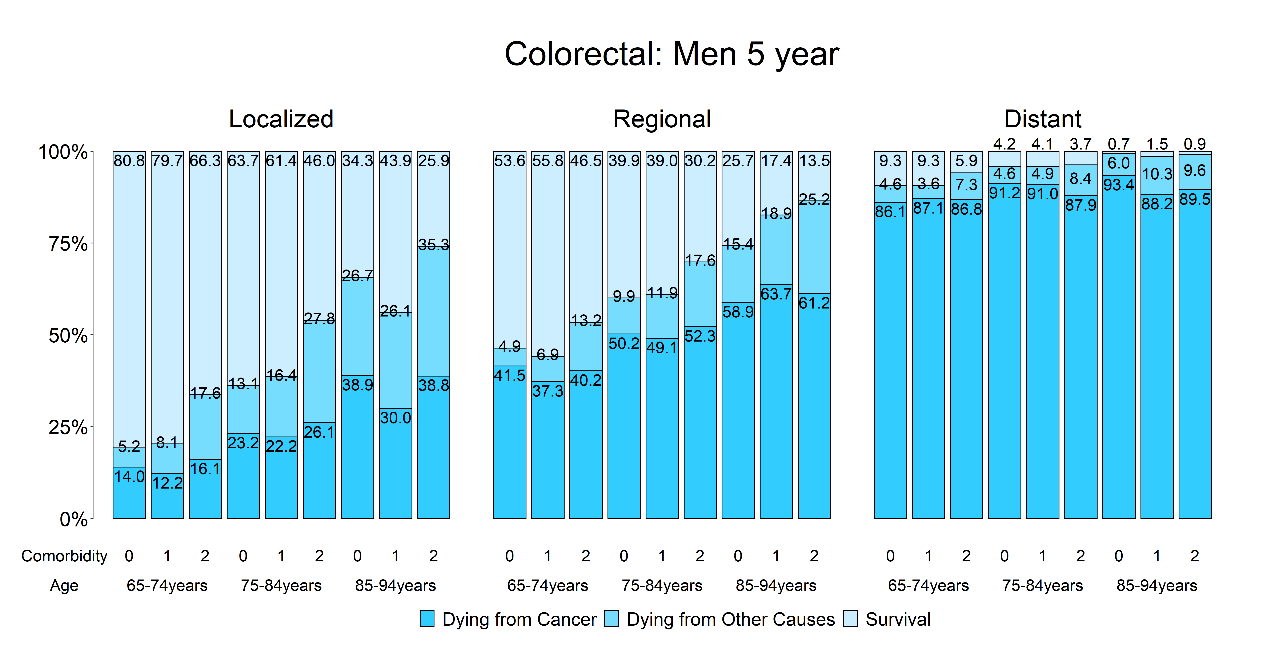
**

**
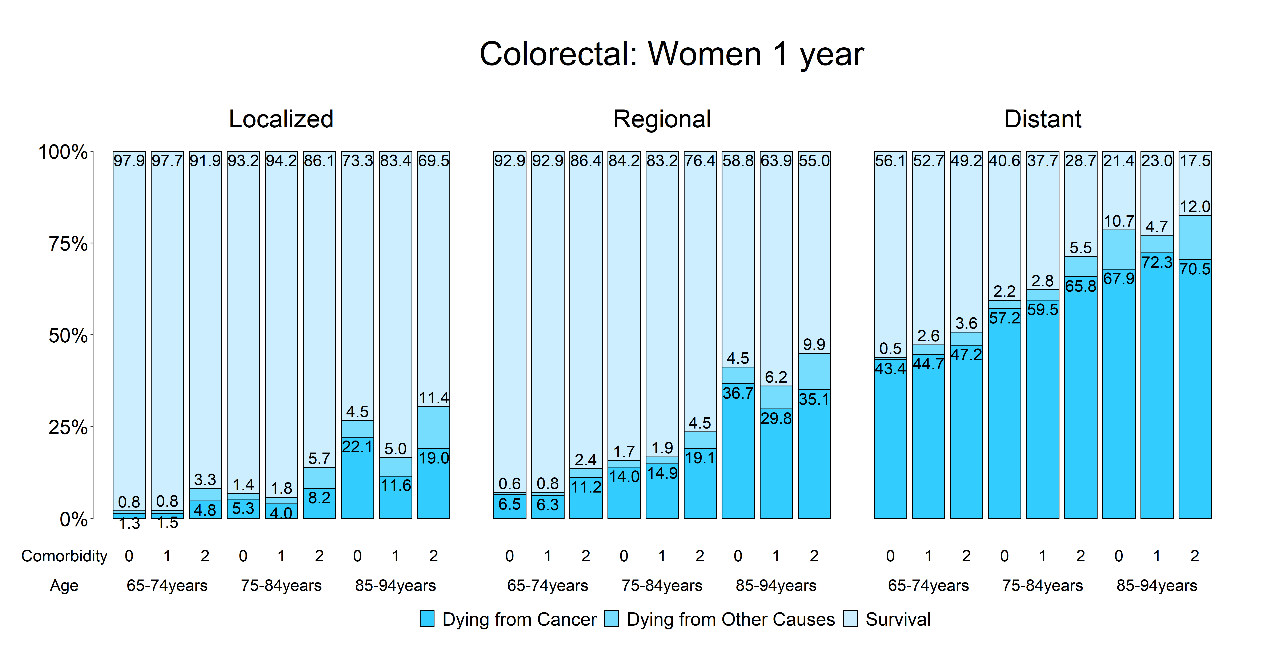

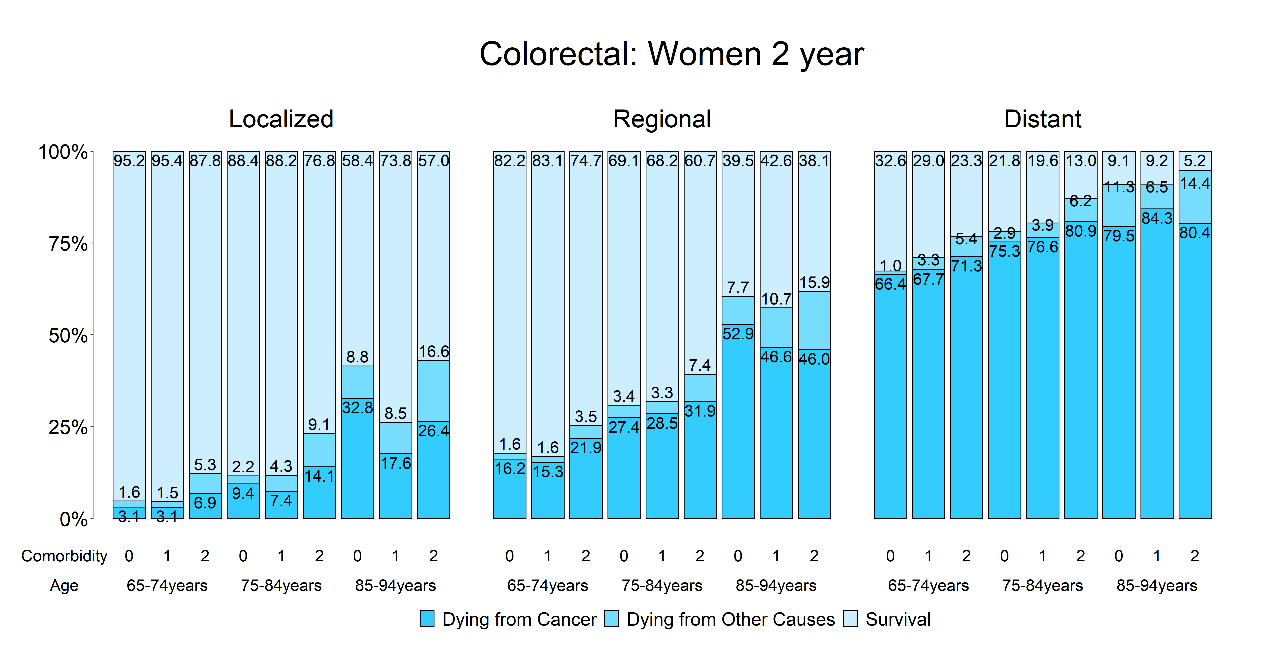

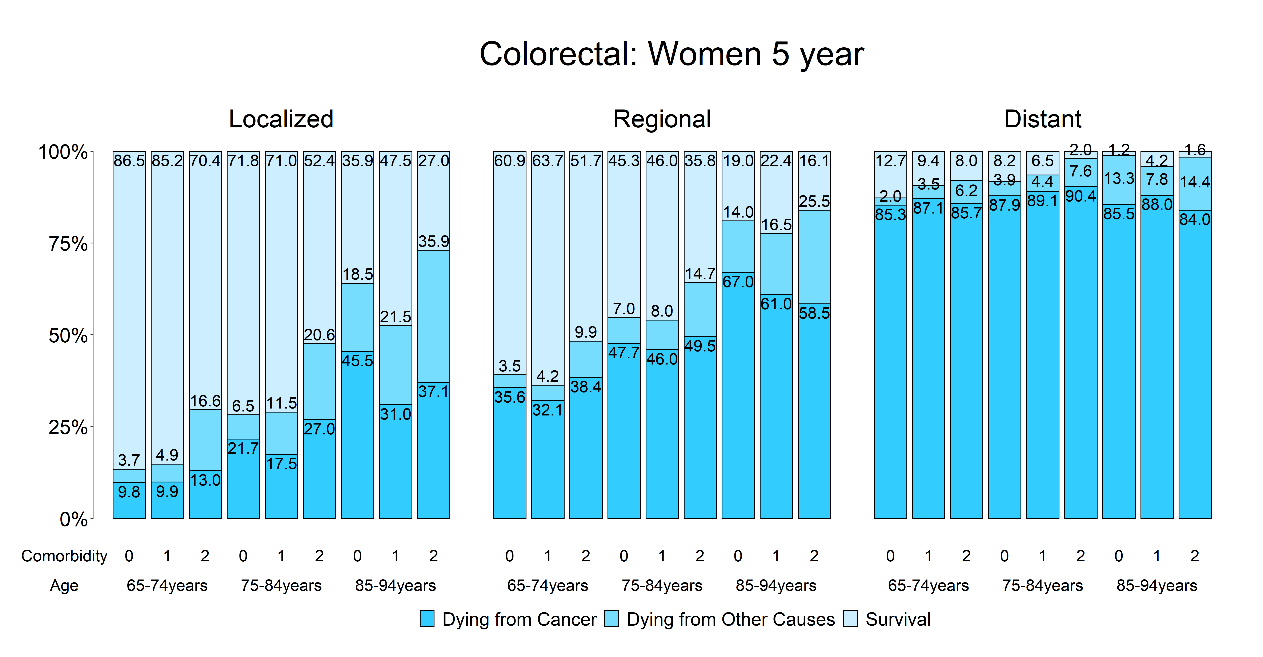
**

## Figure S2-3. Probabilities of dying from cancer, dying from other causes, and survival stratified by stage, comorbidity level, and age for **liver cancer**

**
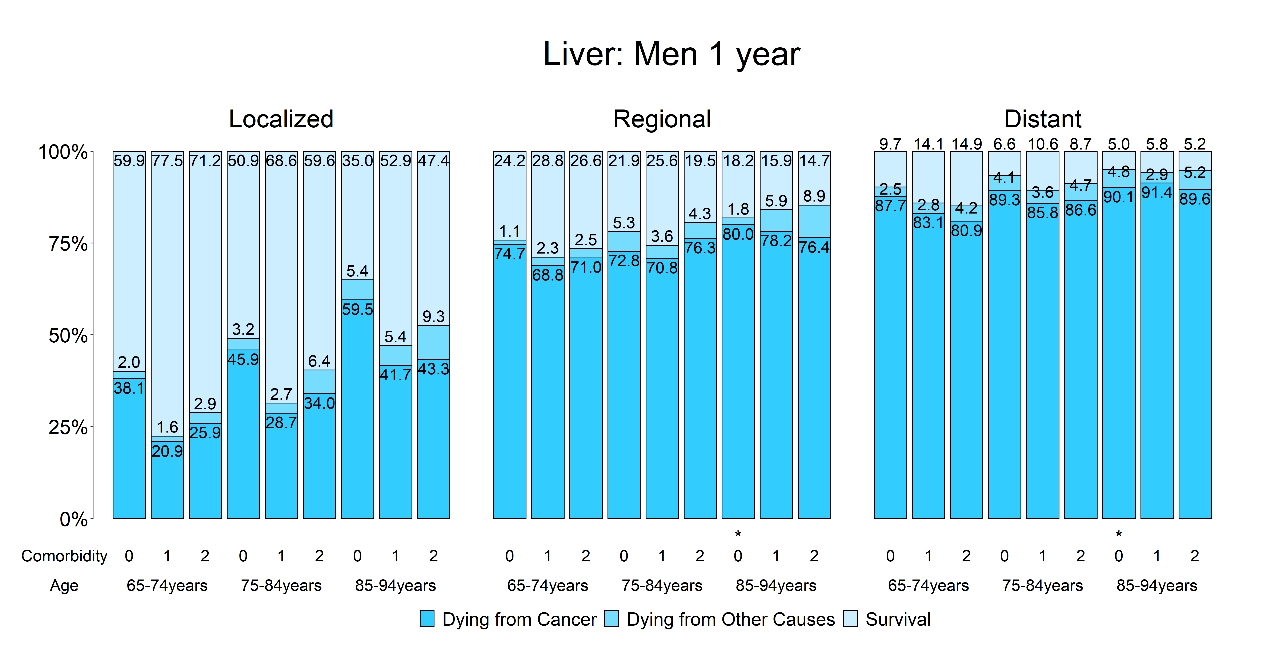

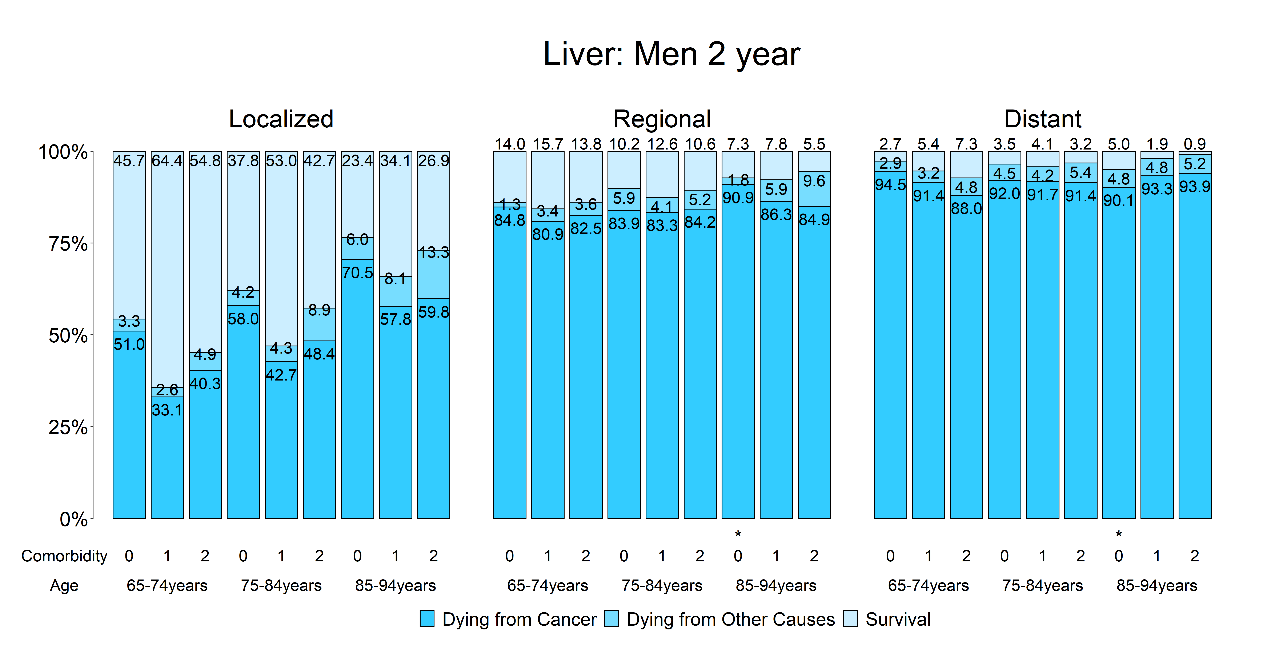

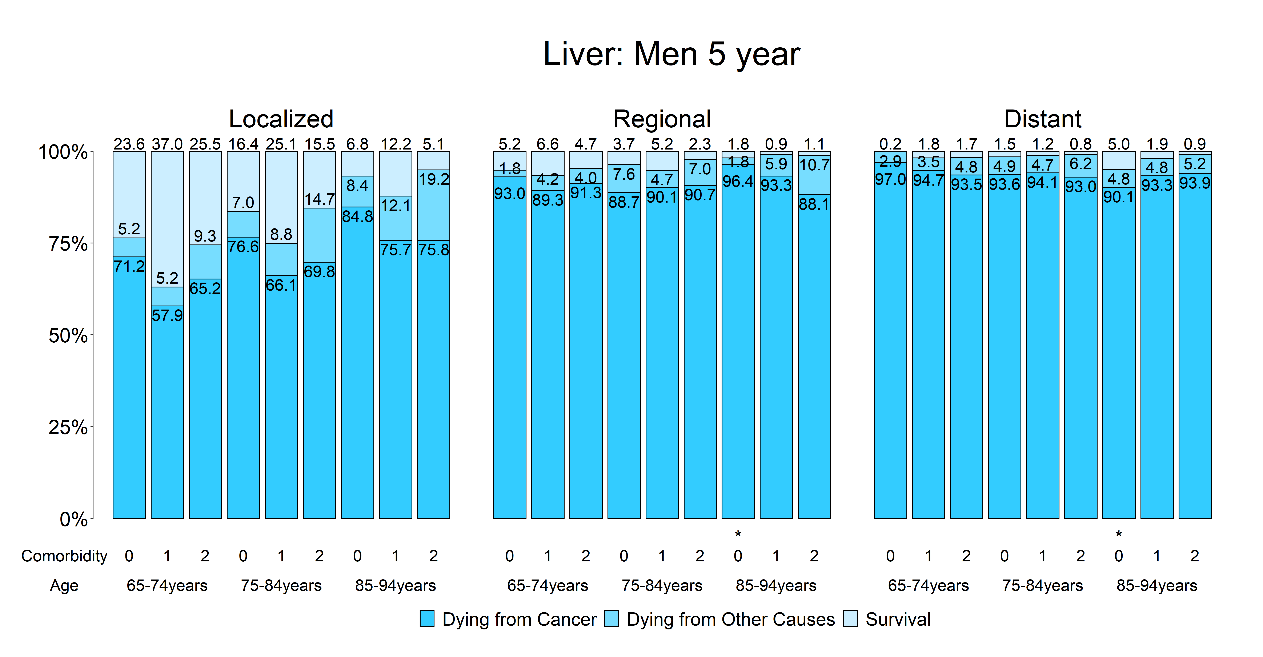
**

**
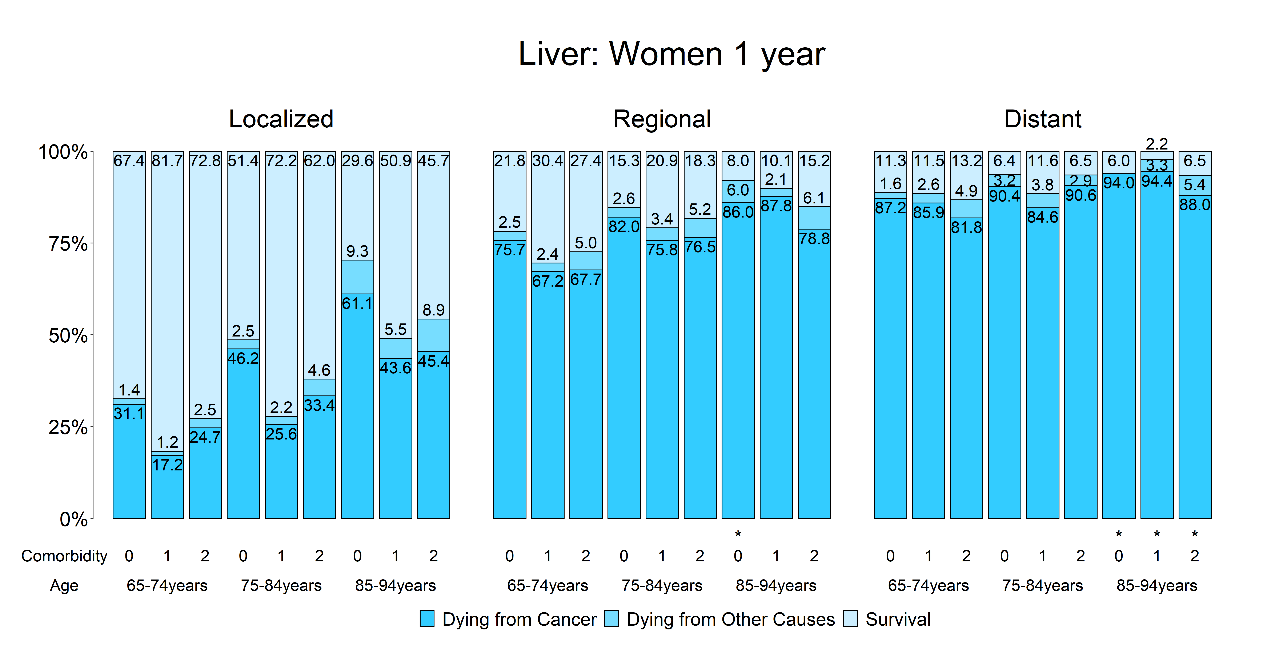

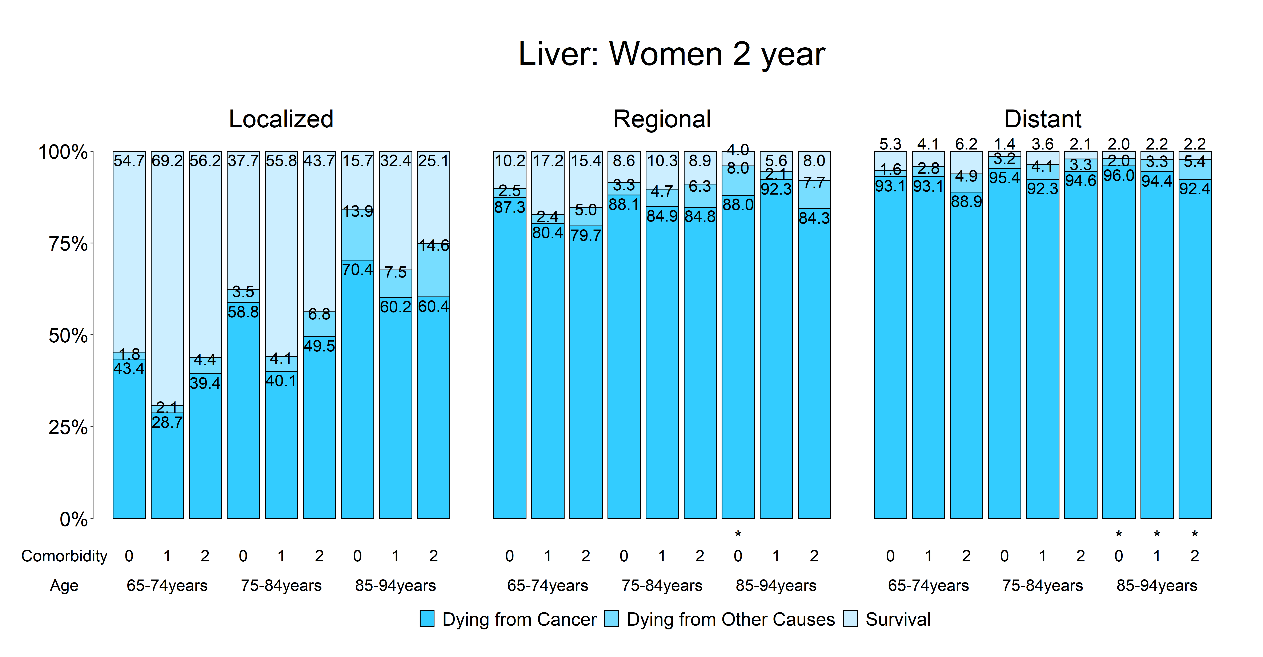

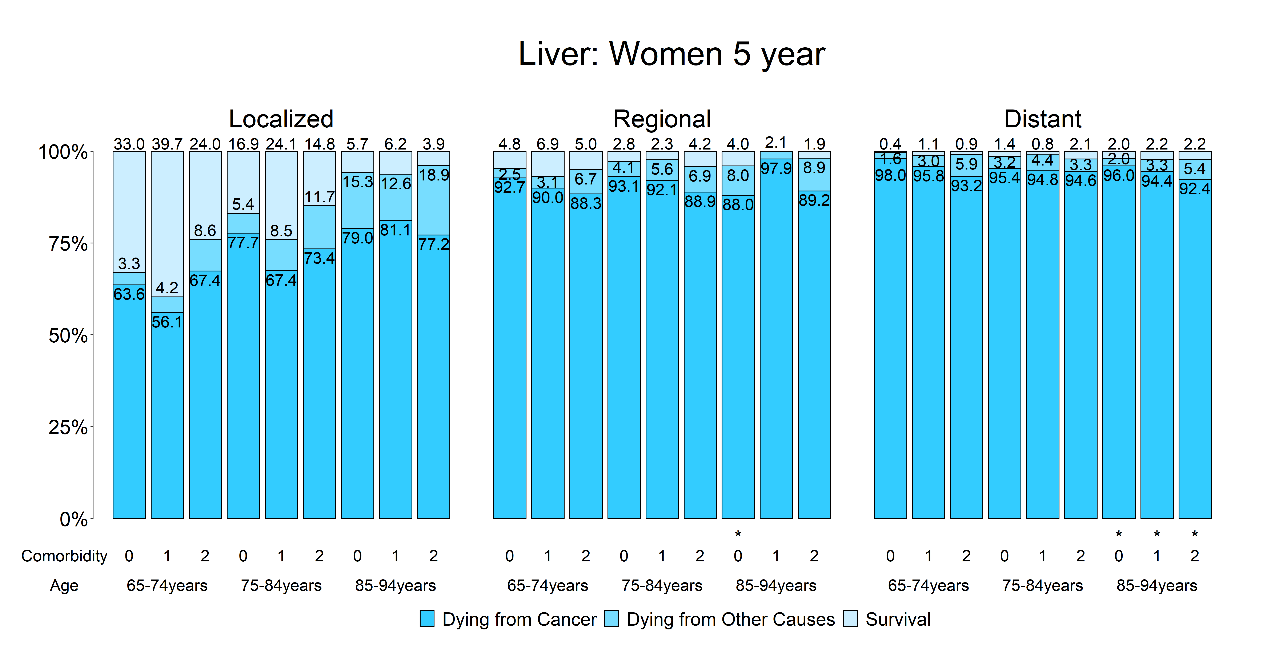
**

## Figure S2-4. Probabilities of dying from cancer, dying from other causes, and survival stratified by stage, comorbidity level, and age for **lung cancer**

**
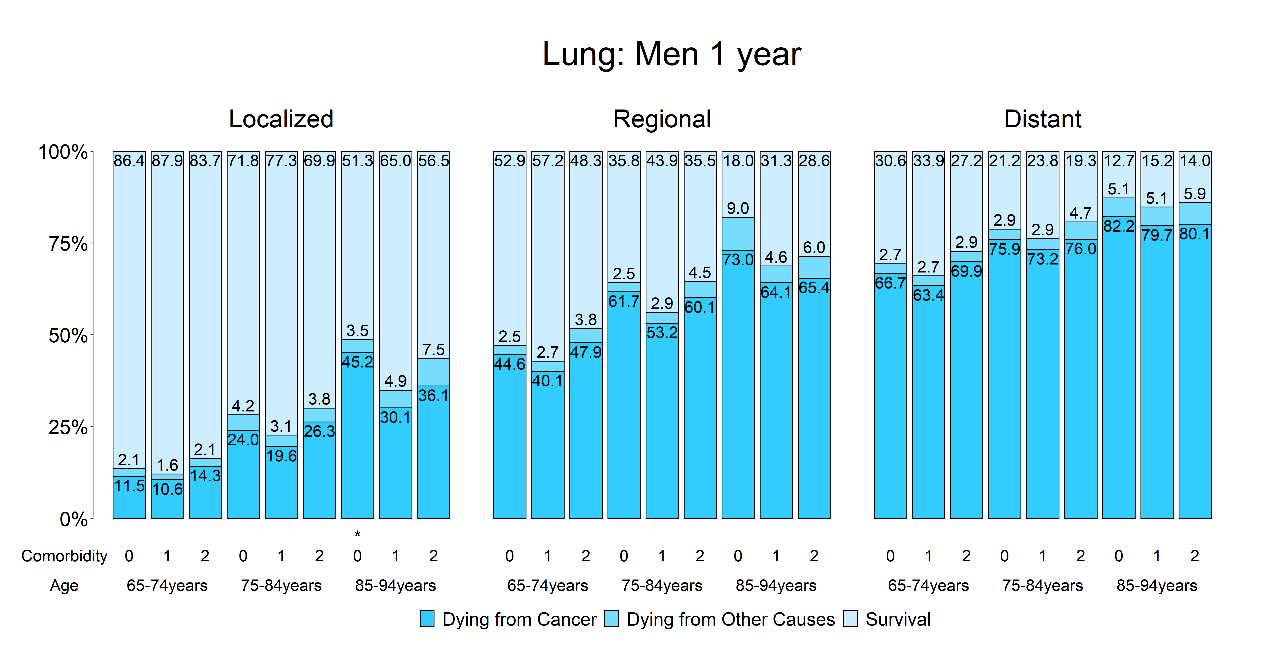

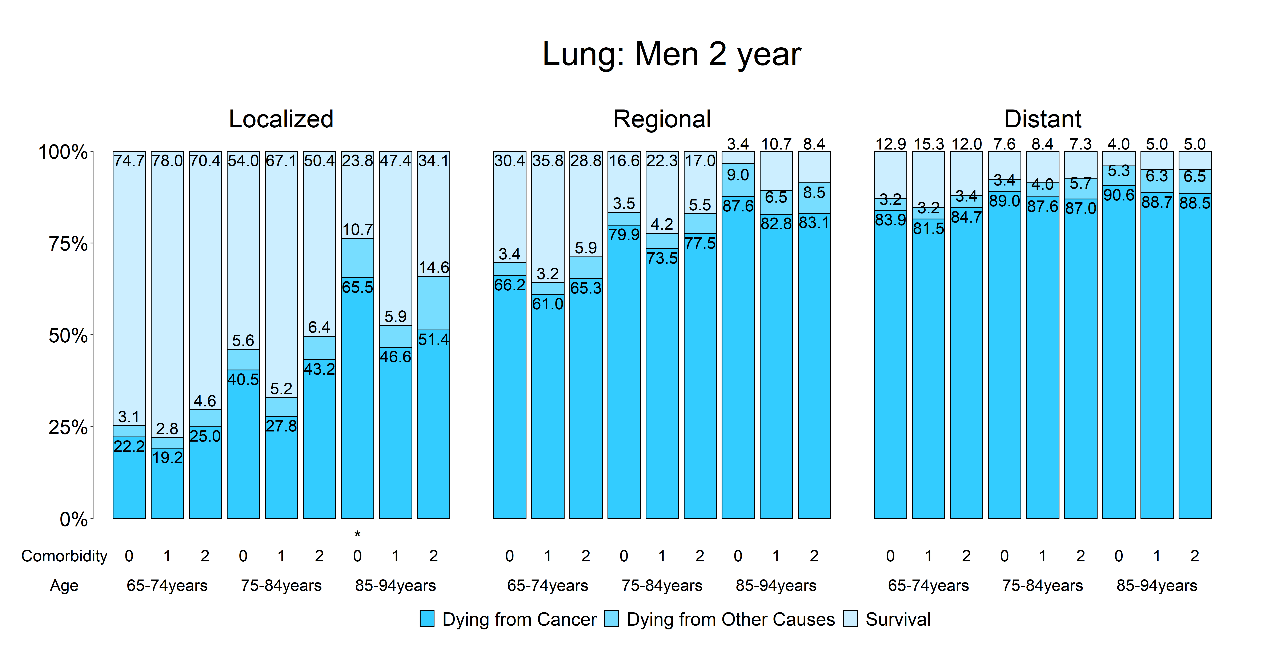

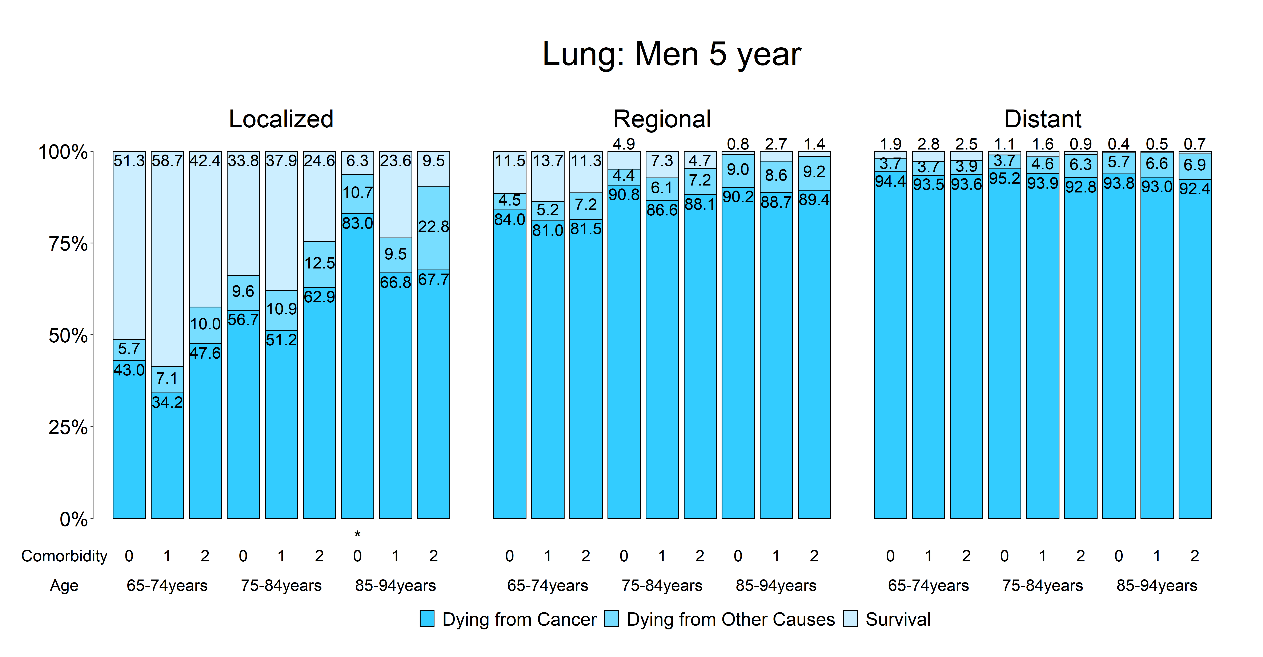
**

**
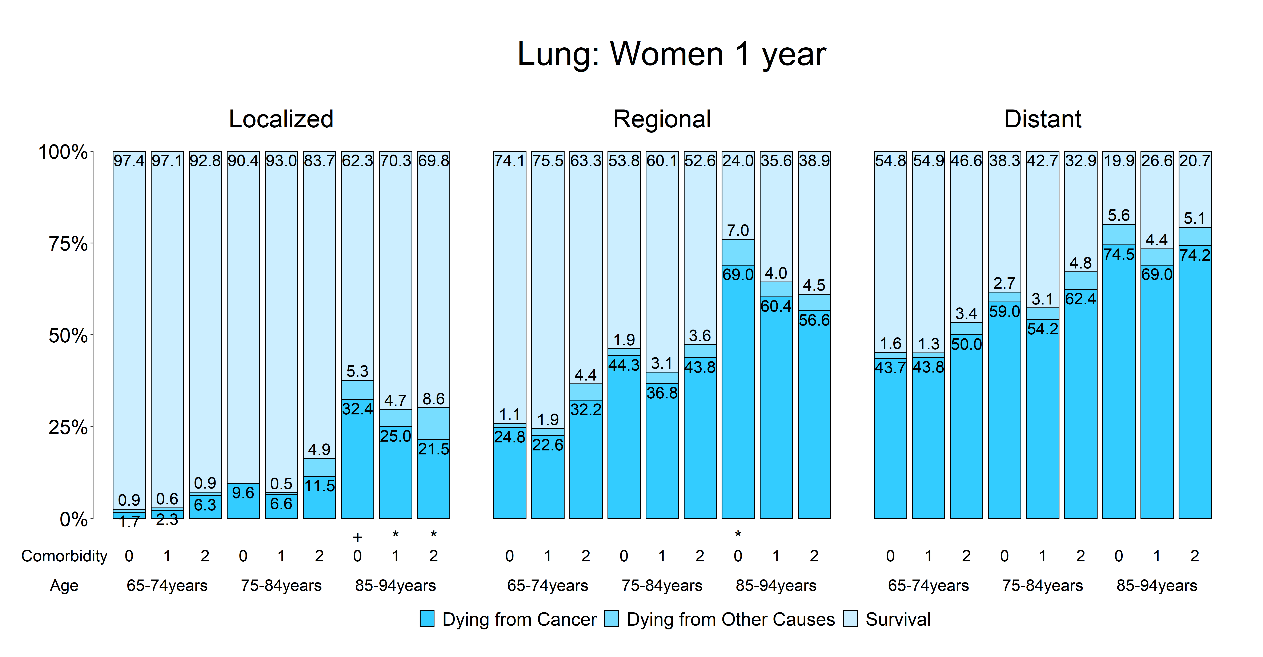

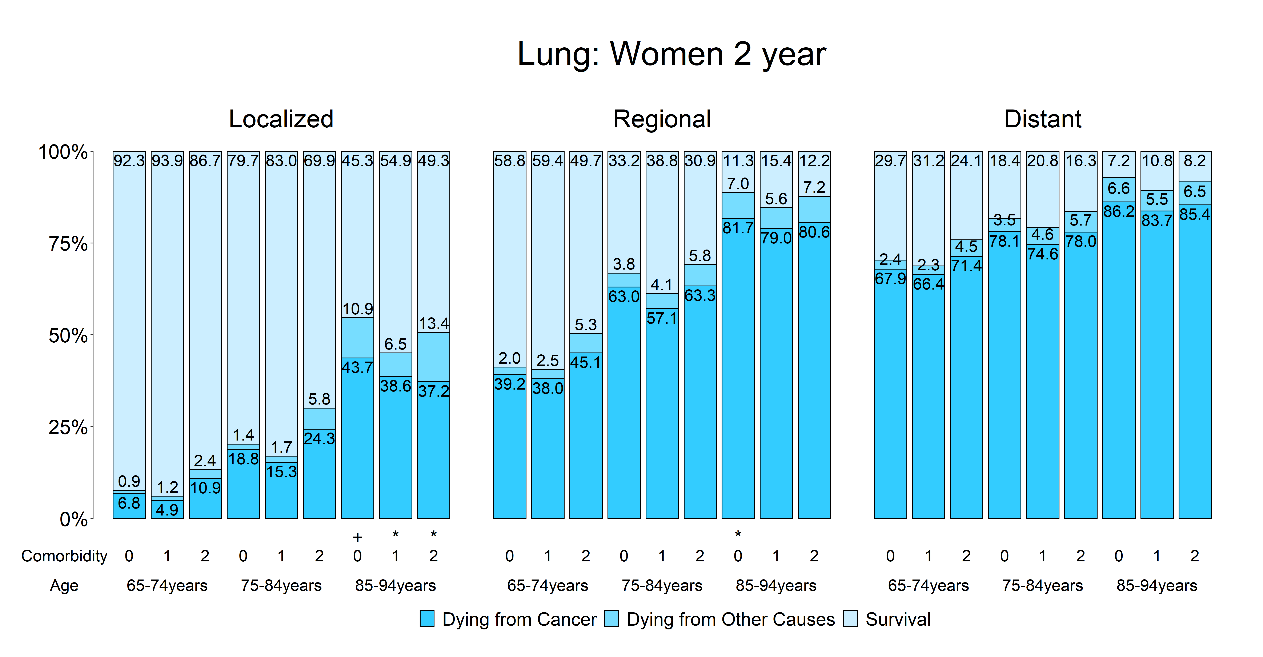

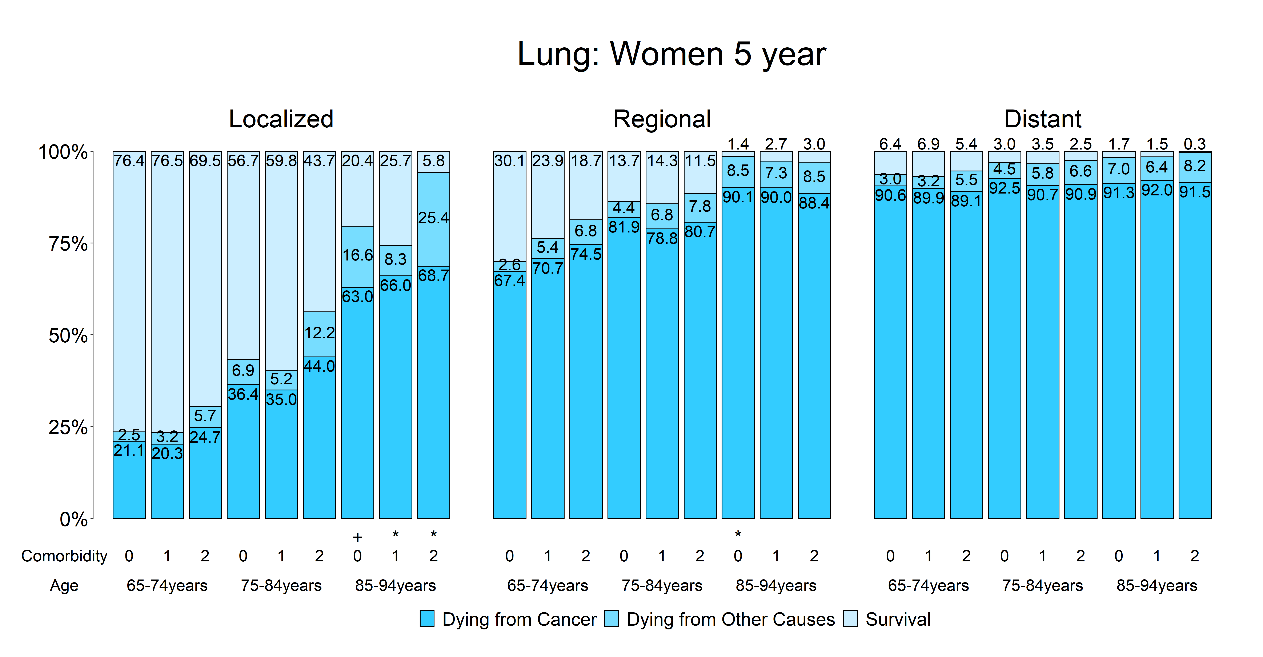
**

## Figure S2-5. Probabilities of dying from cancer, dying from other causes, and survival stratified by stage, comorbidity level, and age for **oral cancer**

**
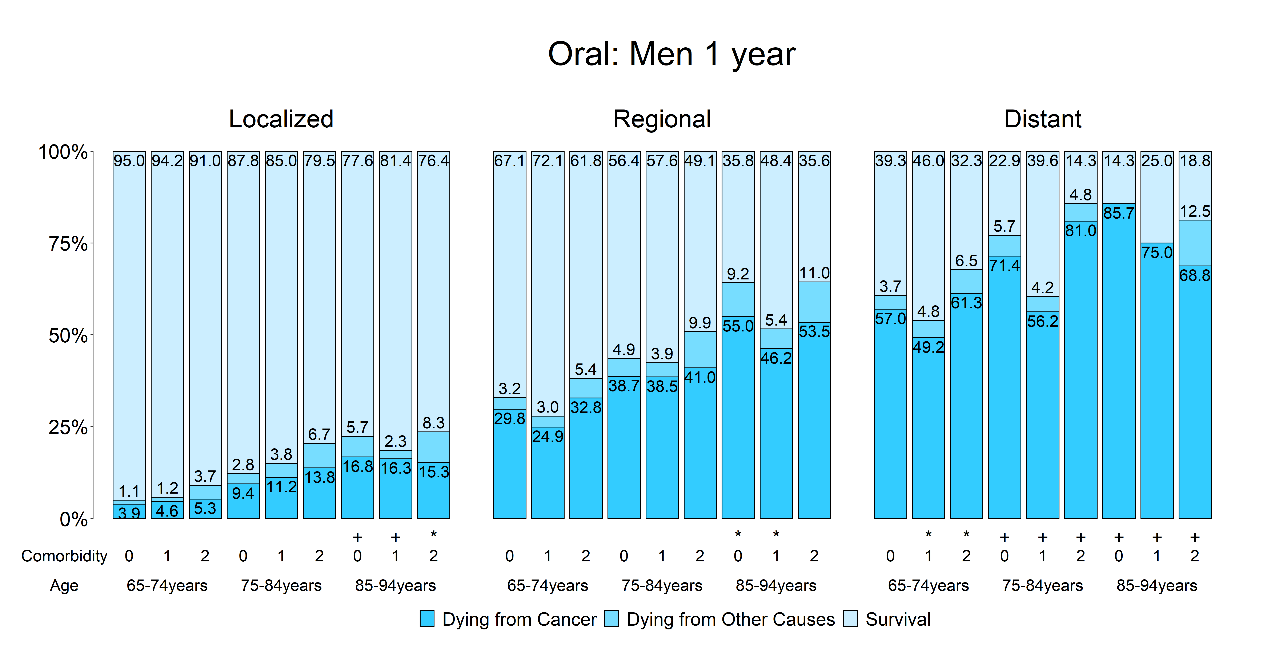

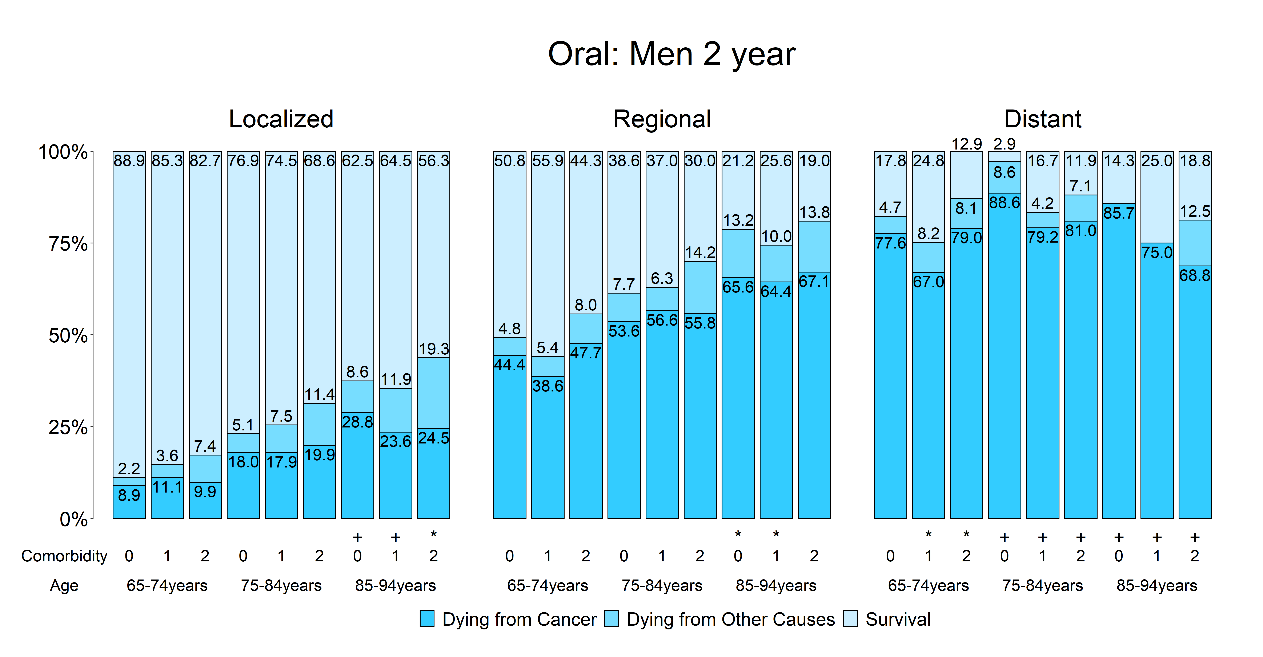

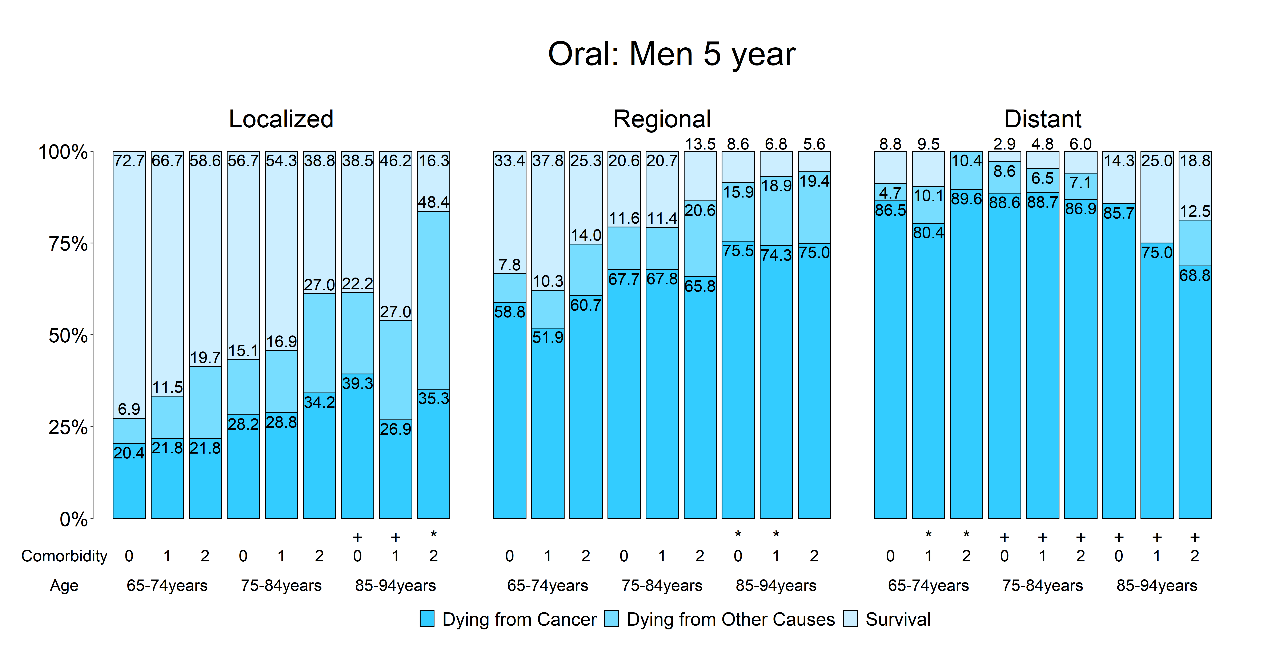
**

**
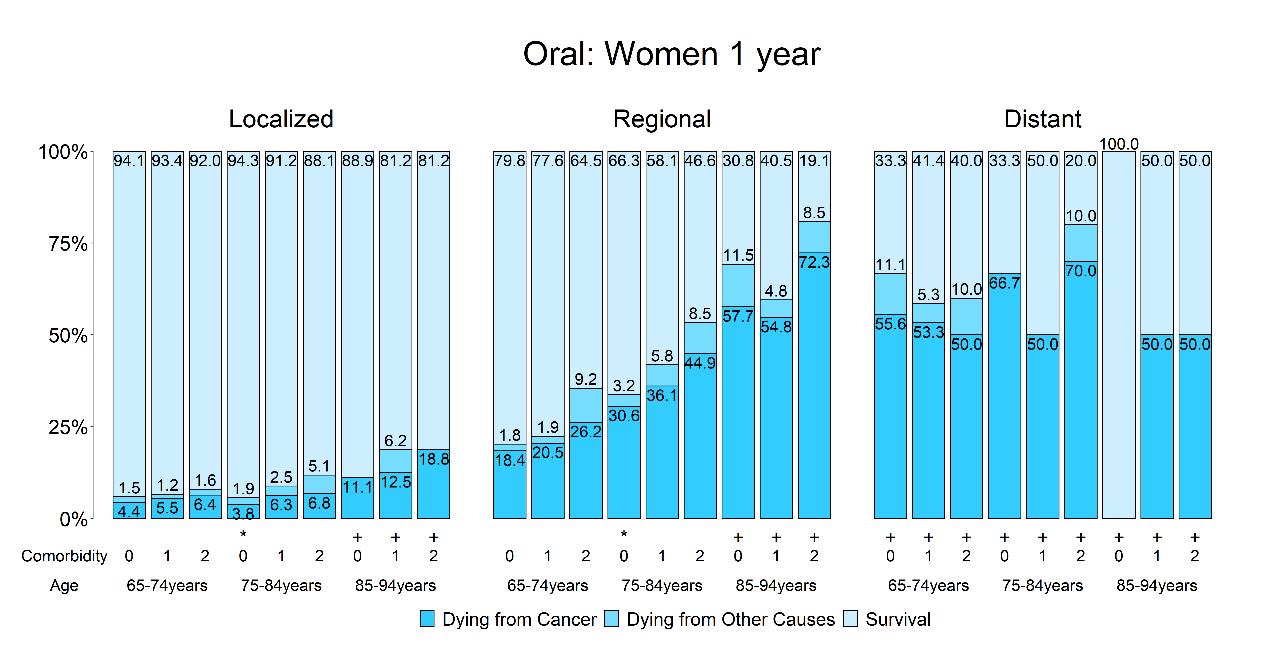

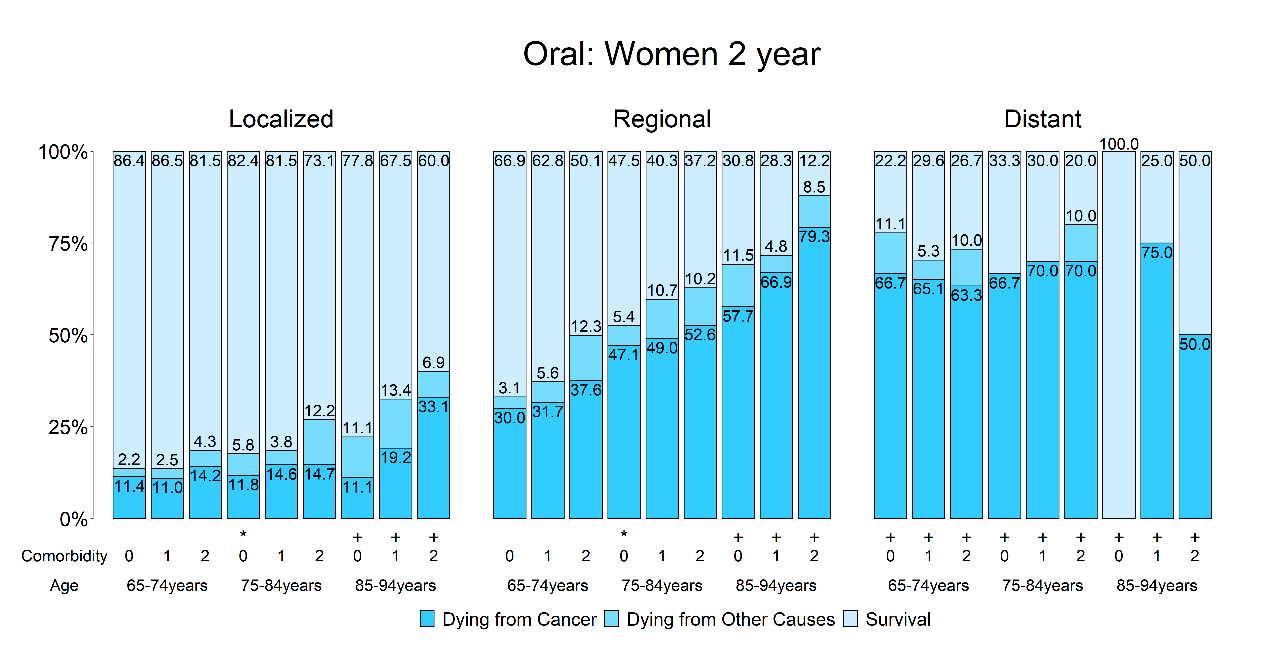

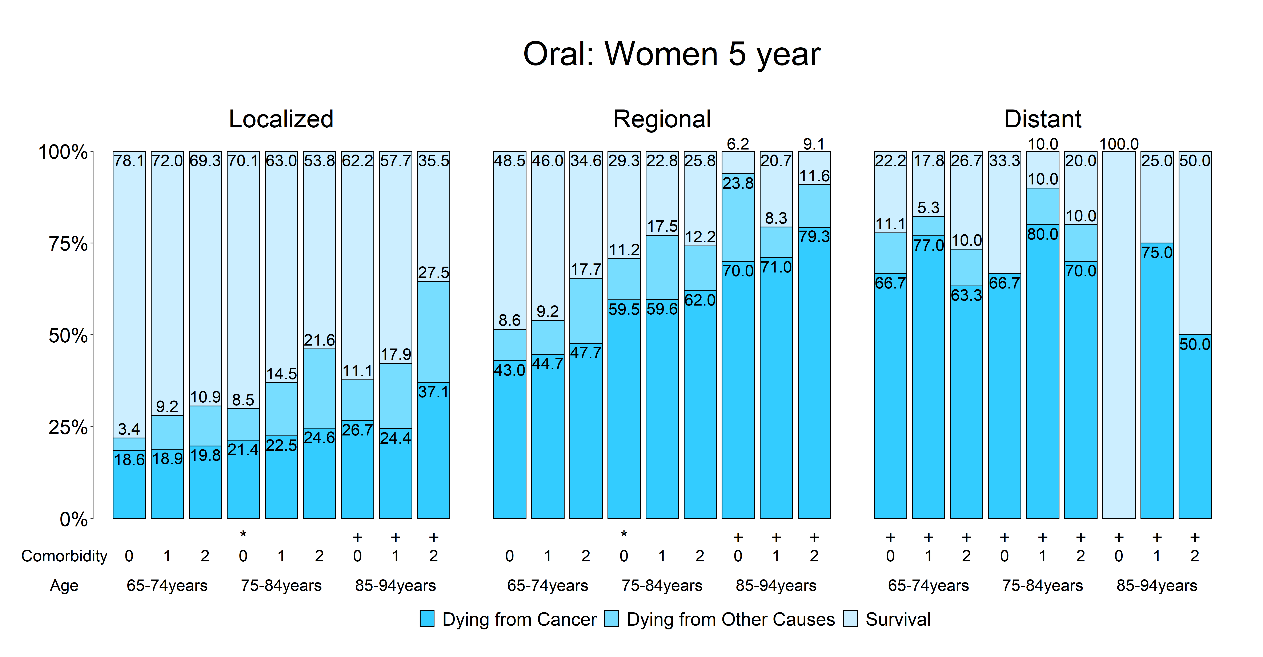
**

## Figure S3-1. One-year probabilities of dying from cancer, dying from other causes, and survival stratified by comorbidity level and subtype for distant **lung cancer** patients ages 30—94.


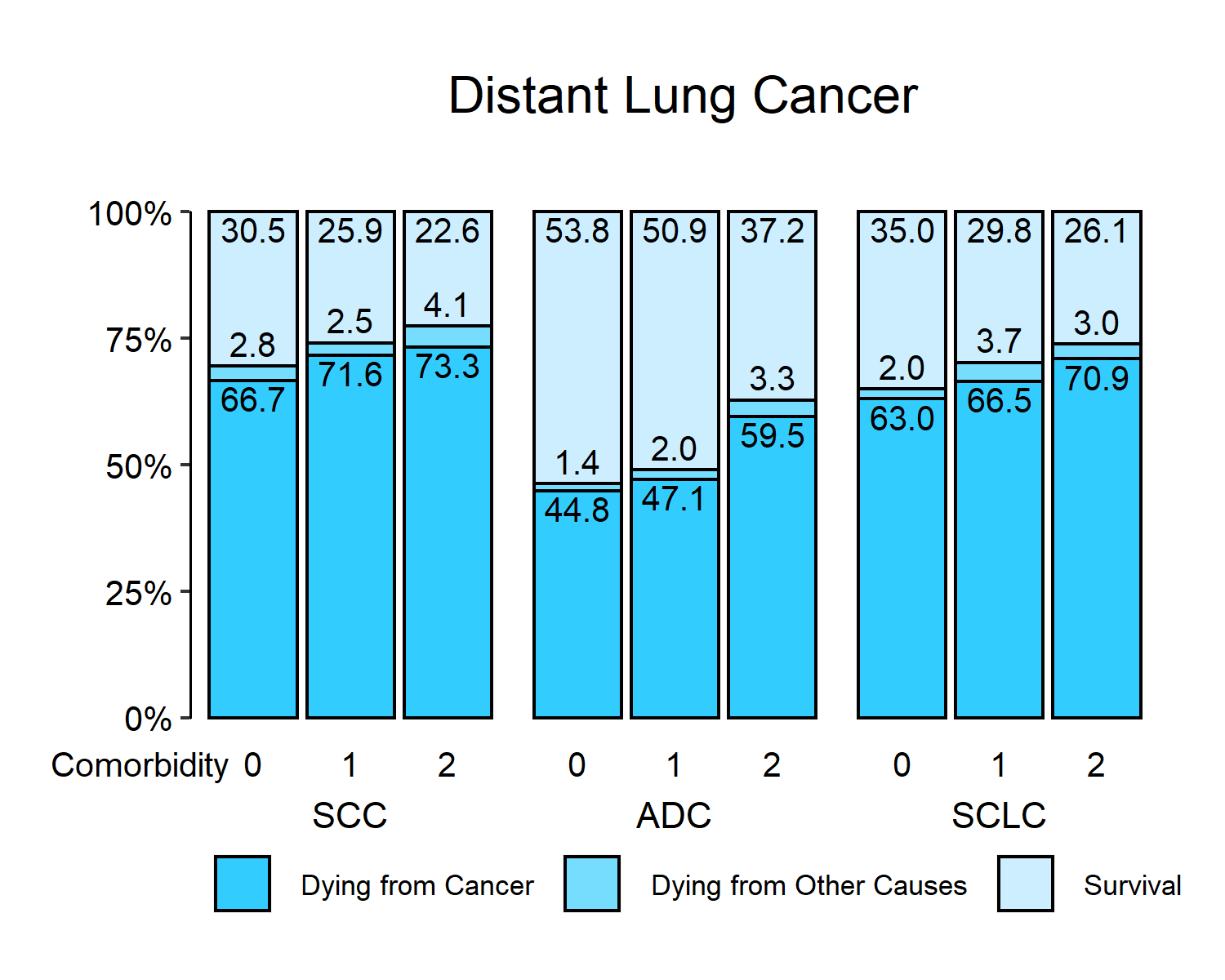


## Figure S3-2. Two-year probabilities of dying from cancer, dying from other causes, and survival stratified by comorbidity level and subtype for distant **lung** **cancer** patients ages 30—94.


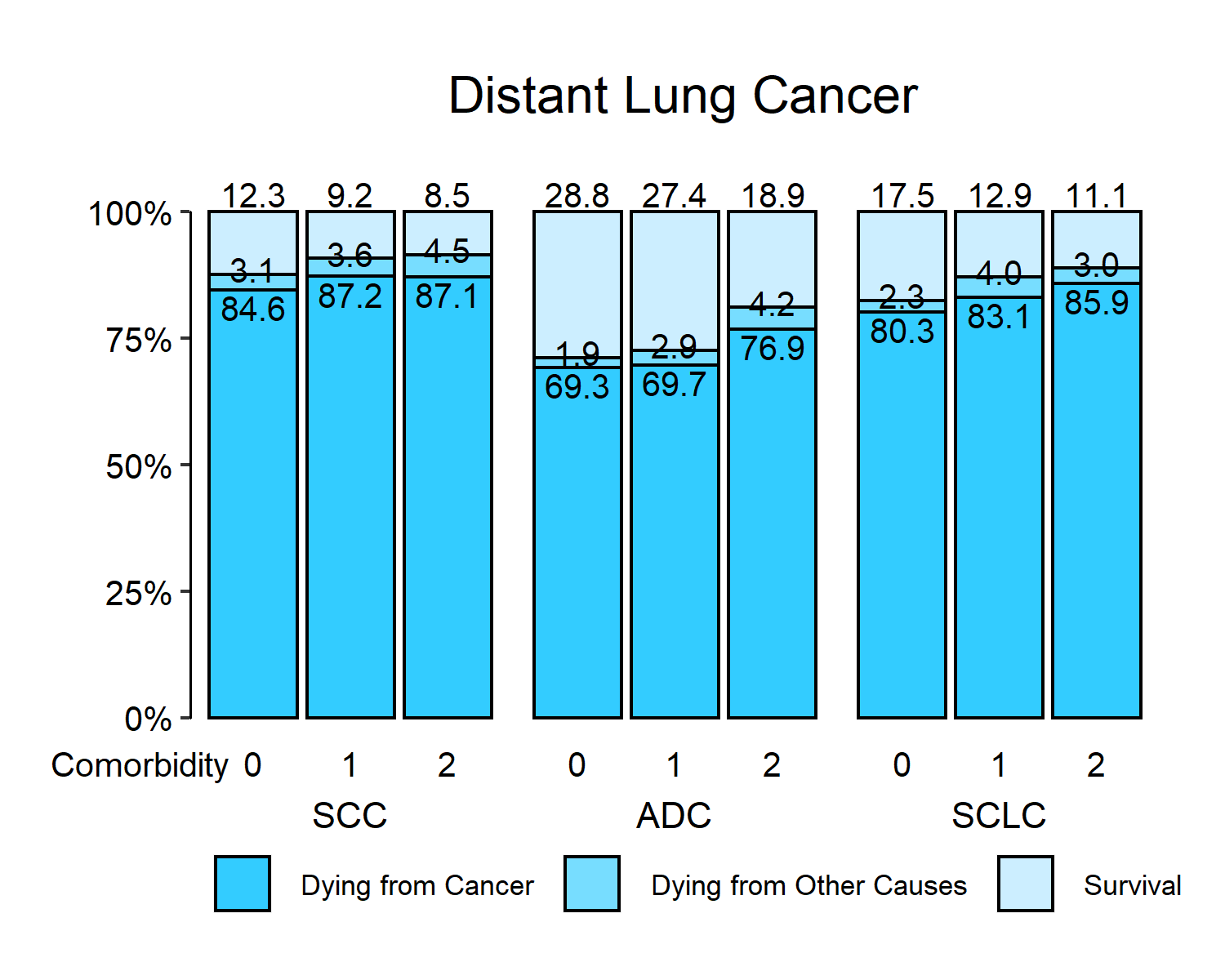


## Figure S3-3. Five-year probabilities of dying from cancer, dying from other causes, and survival stratified by comorbidity level and subtype for distant **lung cancer** patients ages 30—94.


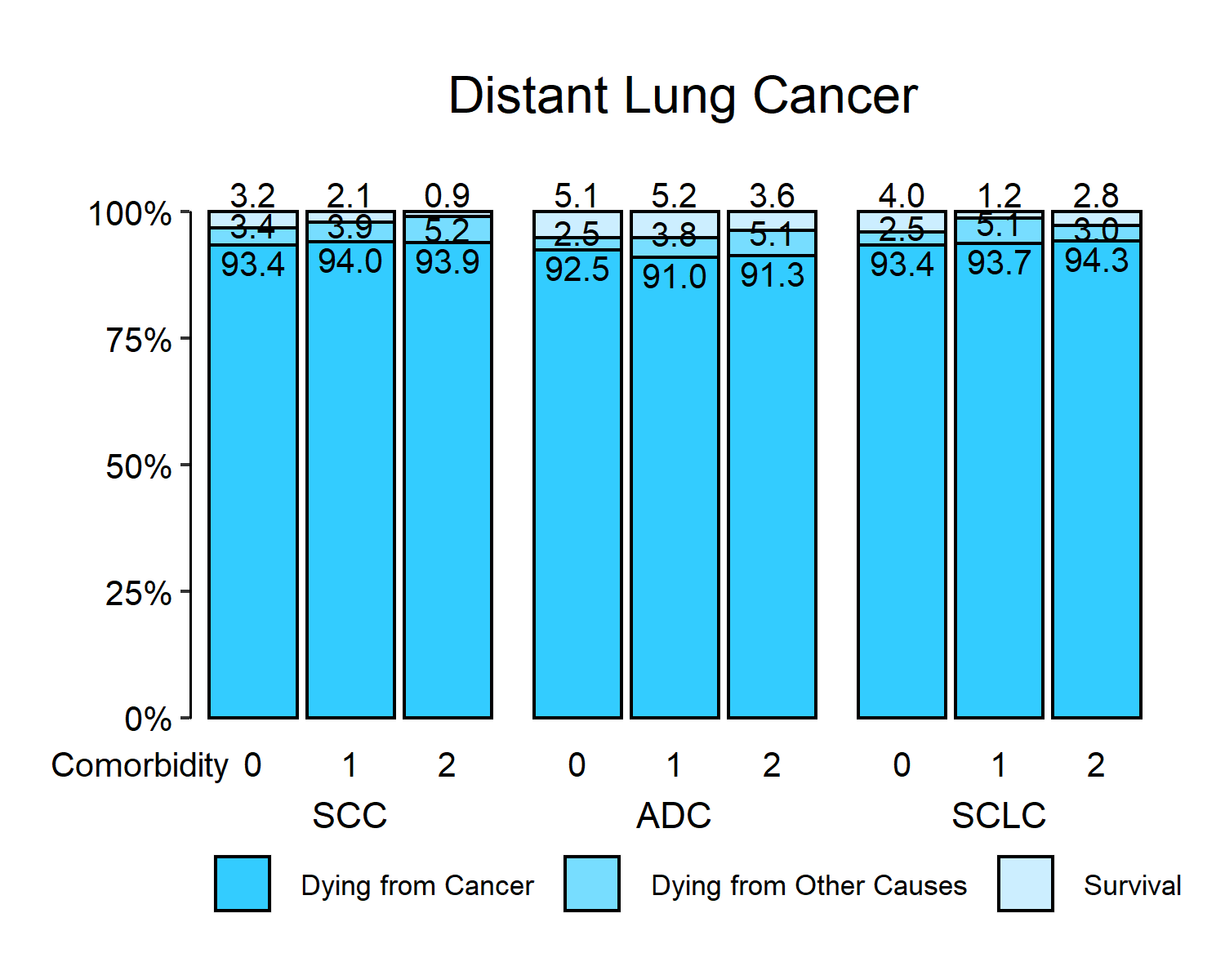


## Figure S4-1. One-year probabilities of dying from cancer, dying from other causes, and survival stratified by comorbidity level and year of diagnosis for distant **lung ADC** patients ages 30—94.


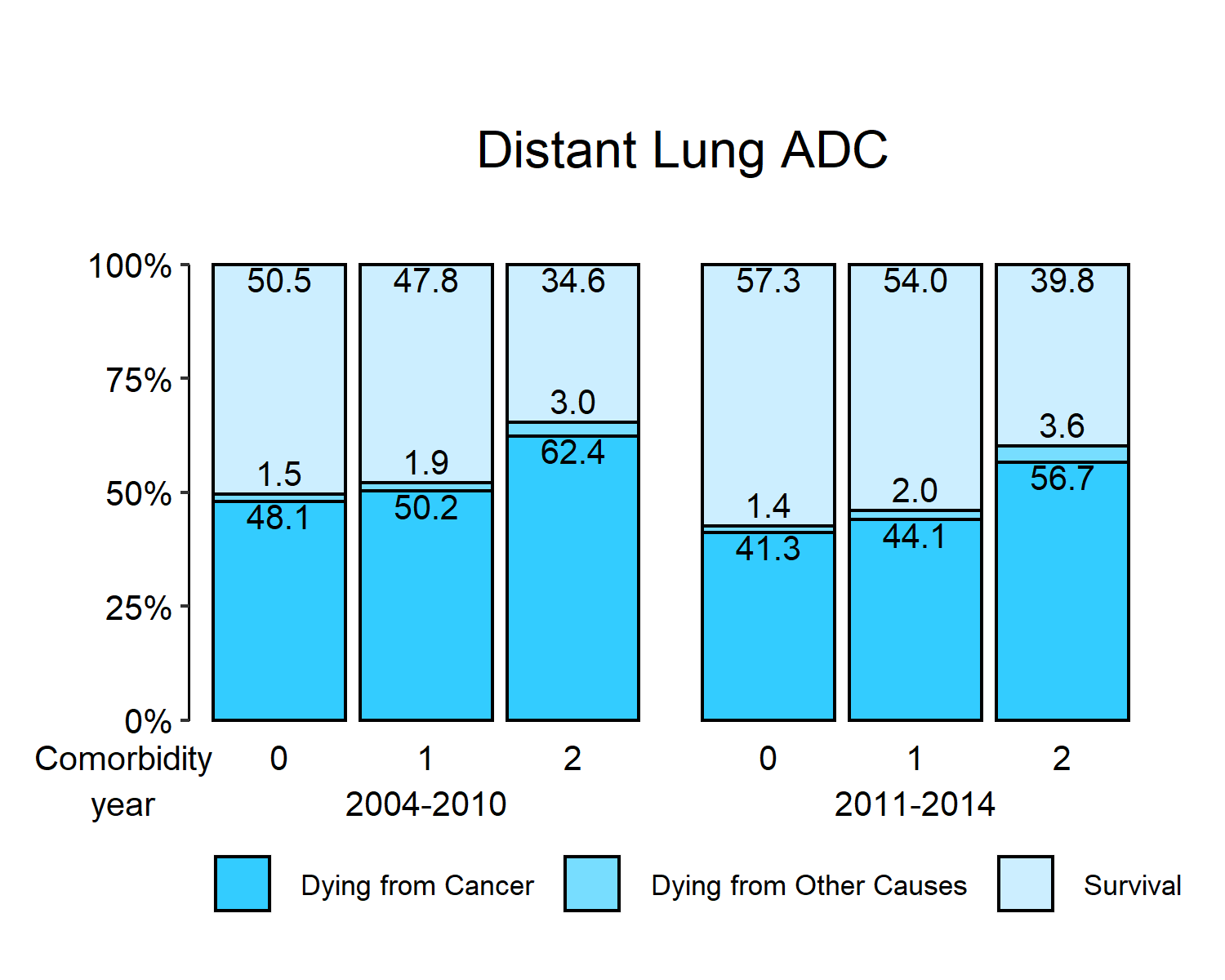


## Figure S4-2. Two-year probabilities of dying from cancer, dying from other causes, and survival stratified by comorbidity level and year of diagnosis for distant **lung ADC** patients ages 30—94.


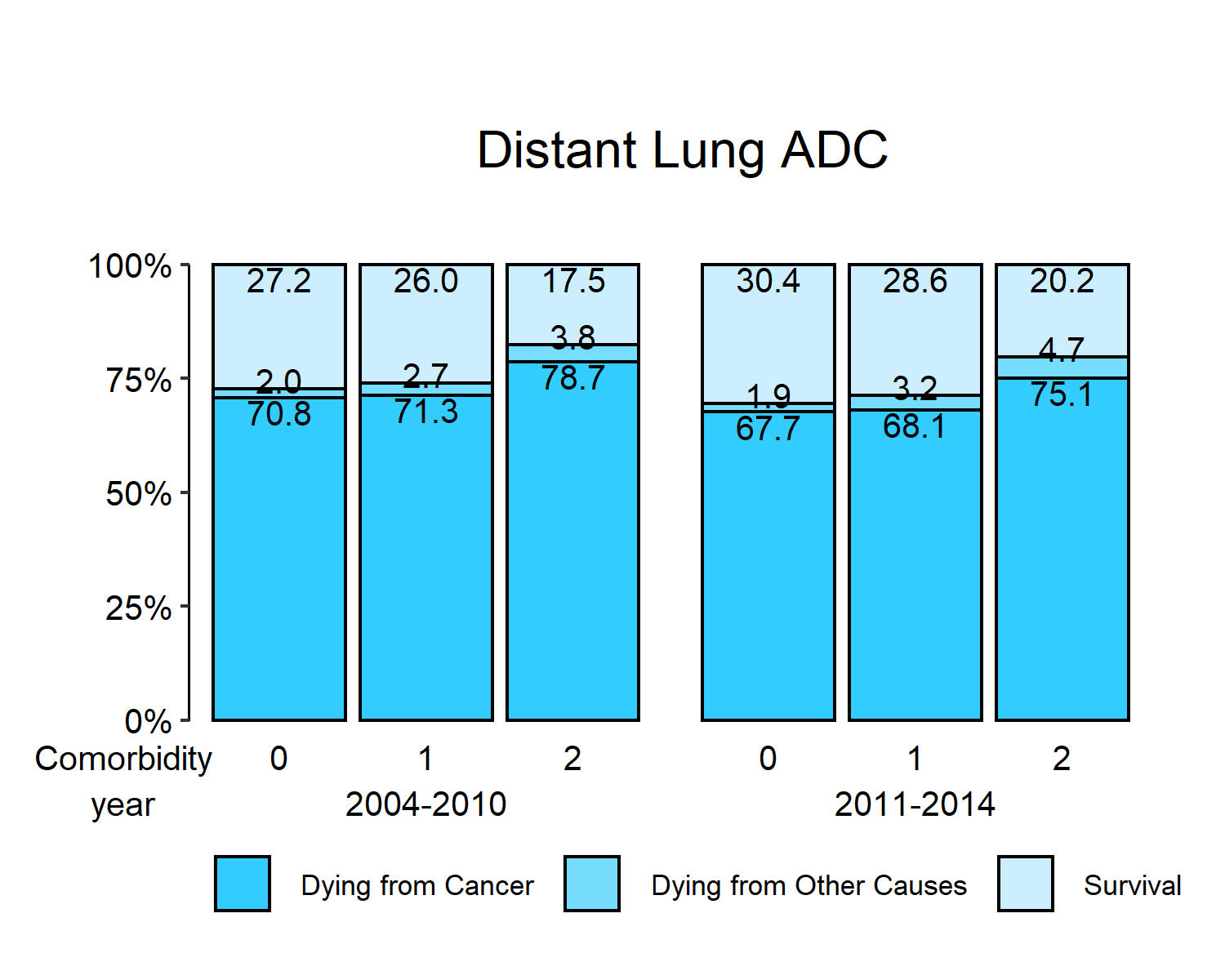


## Figure S4-3. Five-year probabilities of dying from cancer, dying from other causes, and survival are stratified by comorbidity level and year of diagnosis for distant **lung ADC** patients ages 30—94.


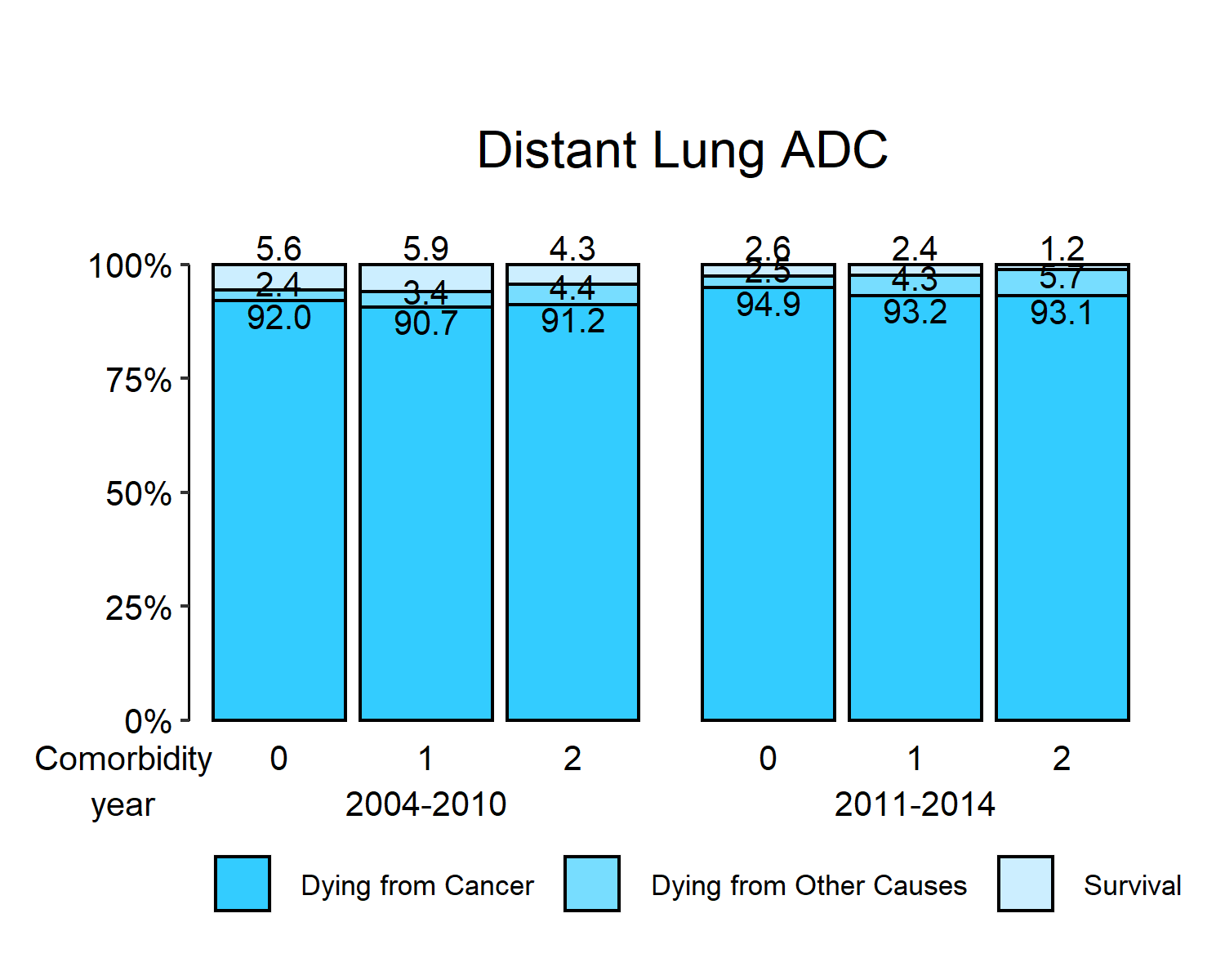


## Figure S5. Probabilities of dying from cancer, dying from other causes and survival stratified by stage, comorbidity level, age, and certain specific sex-subtypes for **lung cancer**


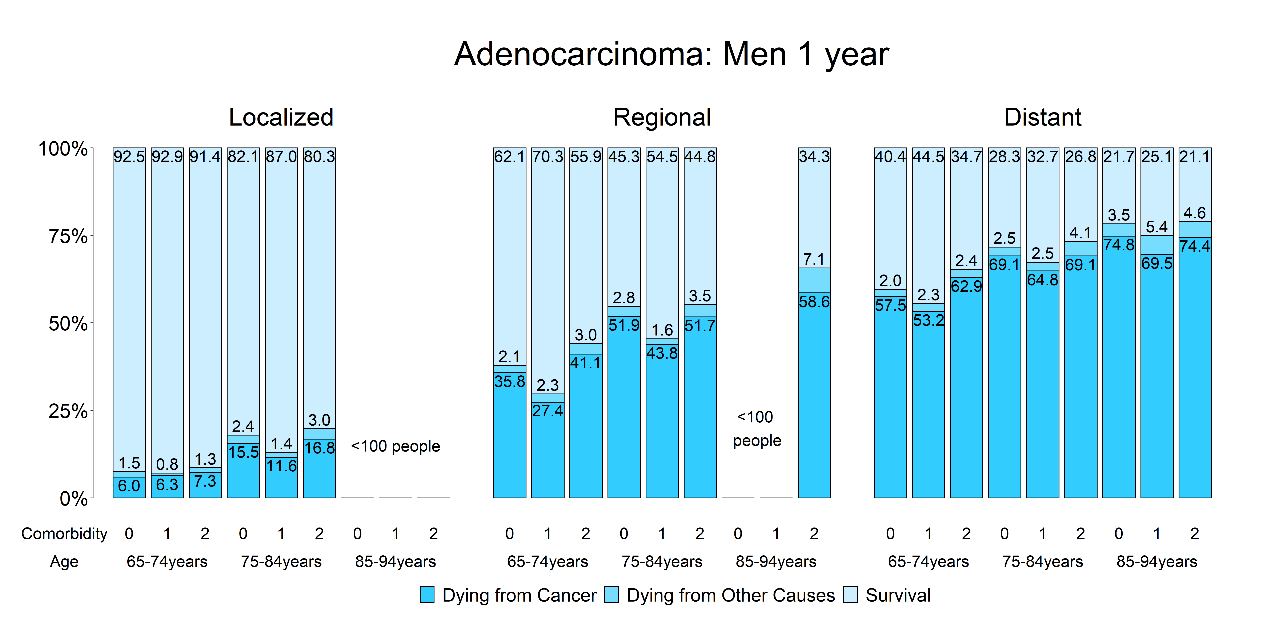

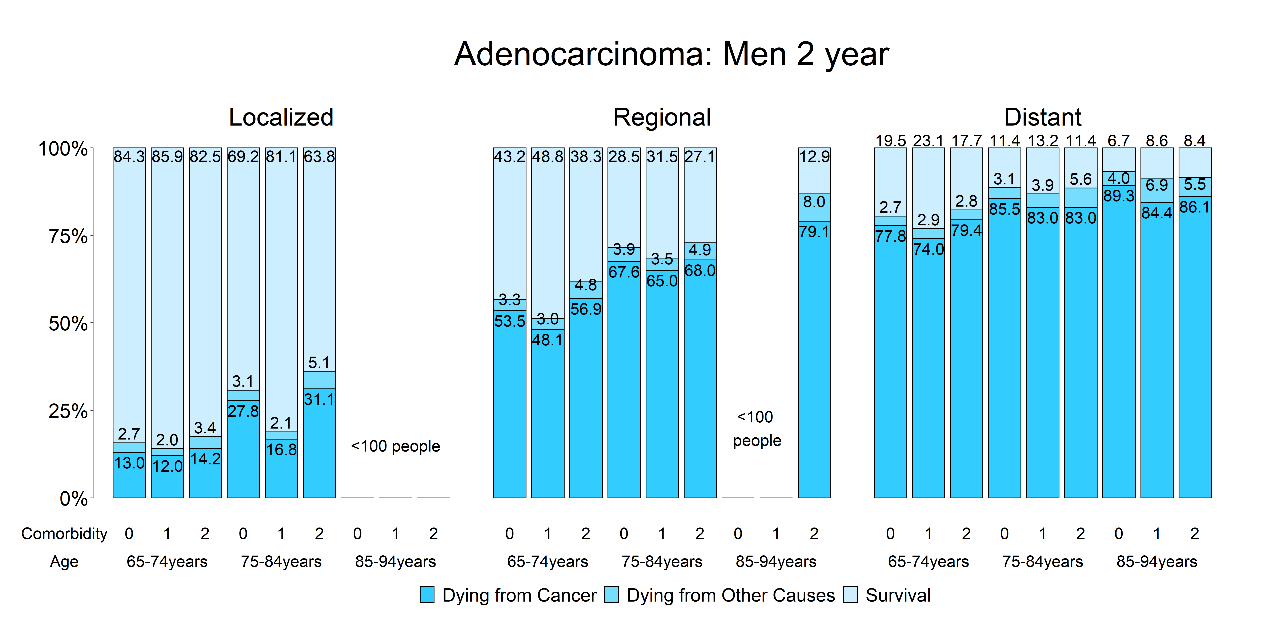

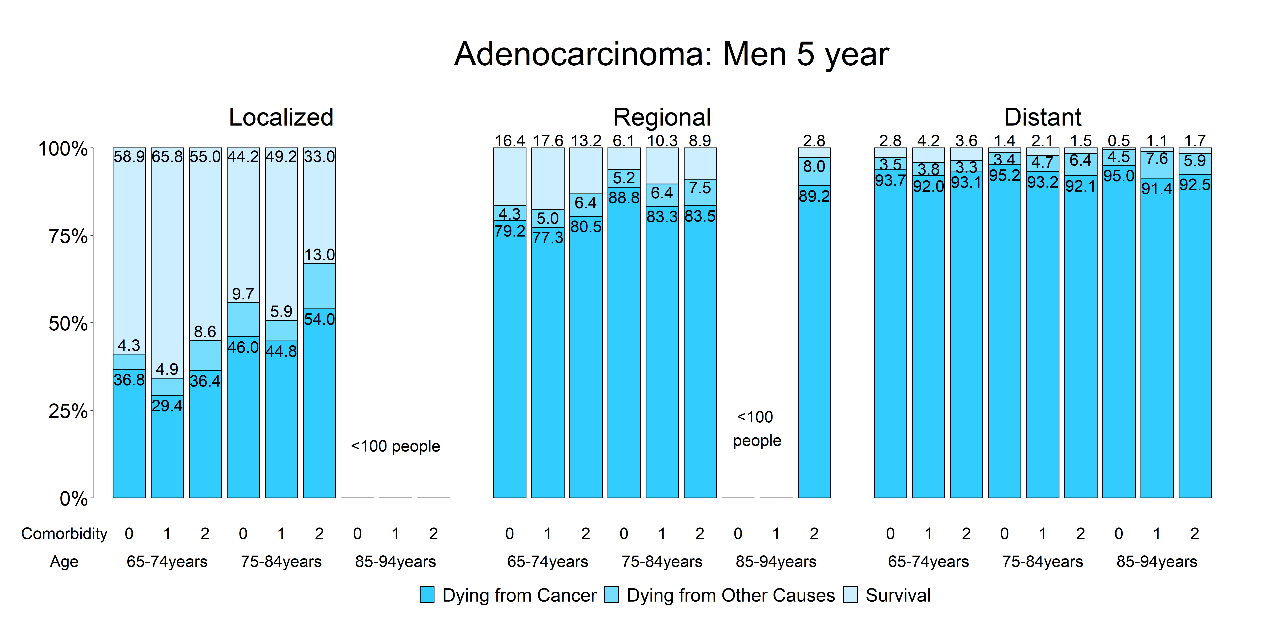


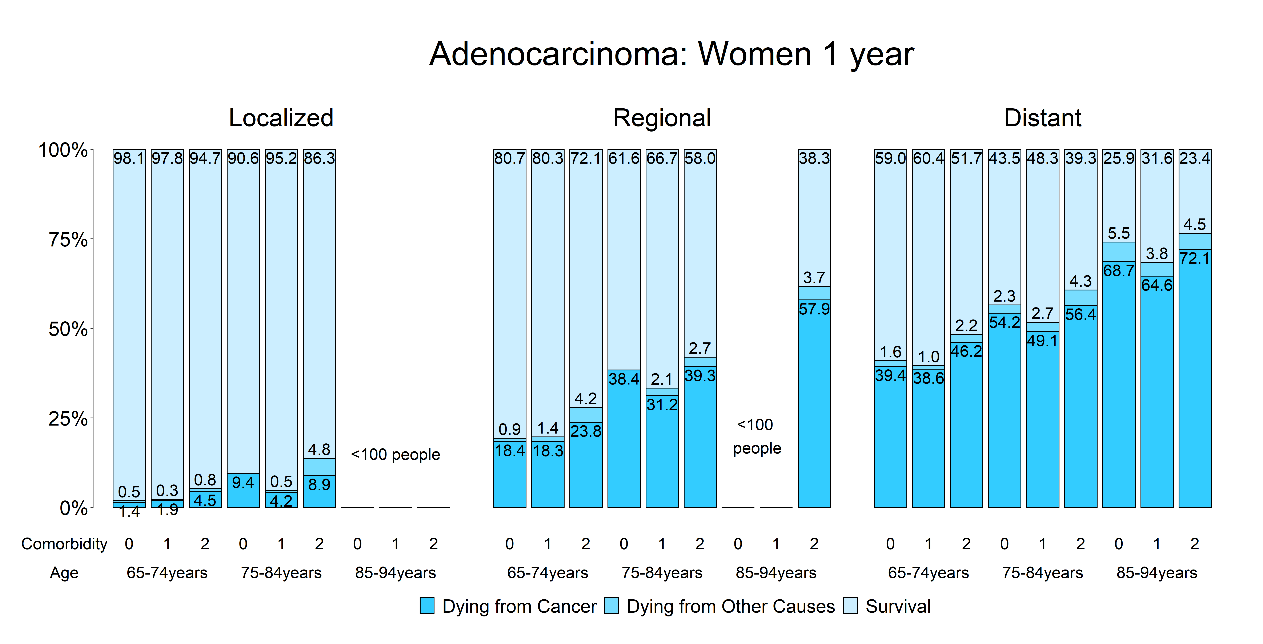


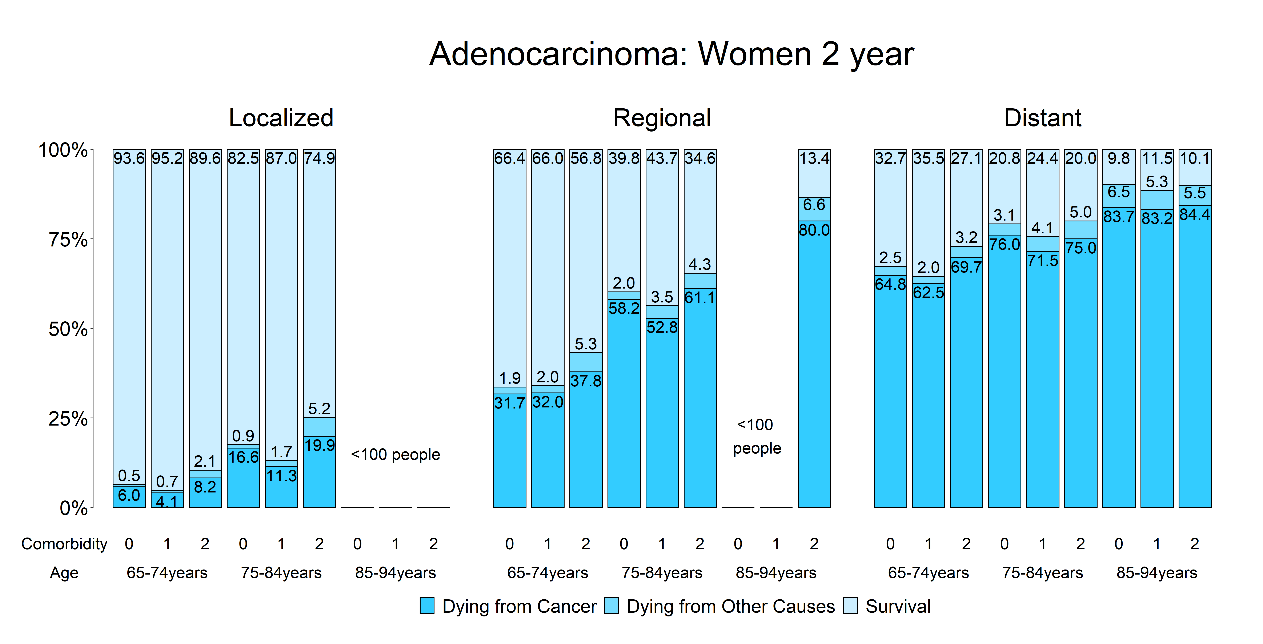


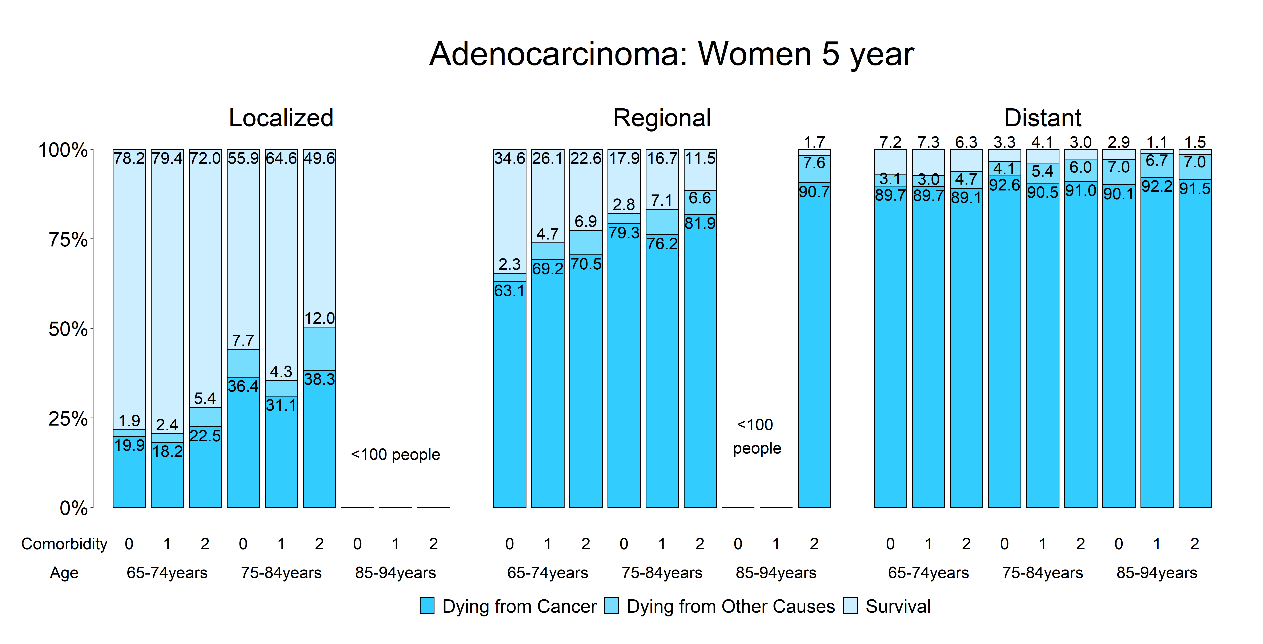


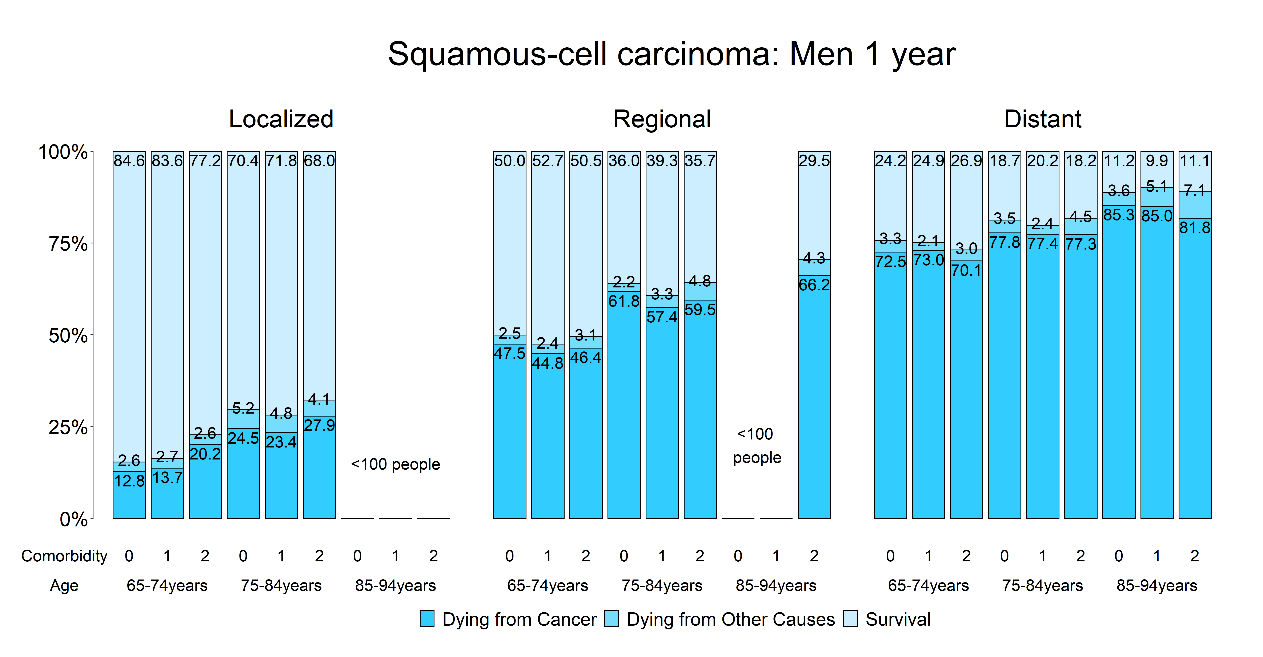


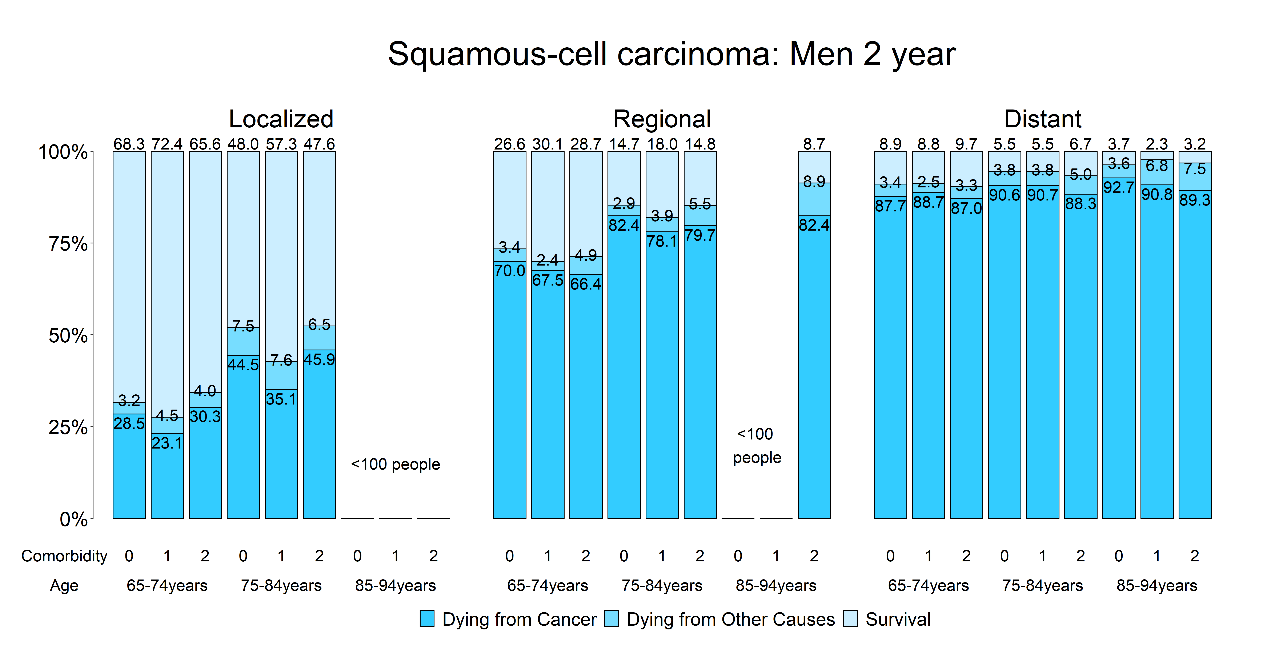


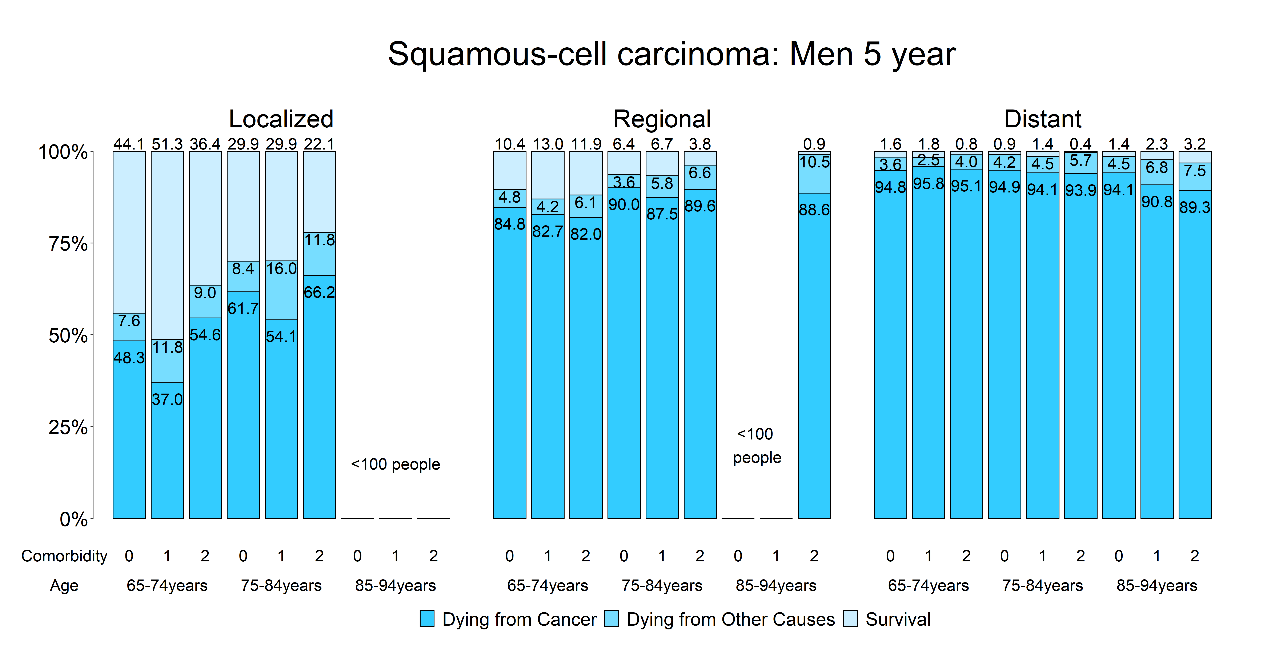

Supplement: Supplementary file 2 — Supplementary Information 2. [file 41598_2023_29582_MOESM2_ESM.docx]
